# Structural Tuning of Vidofludimus for High-Efficacy NR4A Agonism

**DOI:** 10.1021/acs.jmedchem.5c03217

**Published:** 2026-01-26

**Authors:** Jan Vietor, Romy Busch, Úrsula López-García, Tanja Stiller, Anna Maria Thommes, Christian Gege, Daniel Merk

**Affiliations:** 1 Ludwig-Maximilians-Universität München, Department of Pharmacy, 81377 Munich, Germany; 2 Immunic AG, 82166 Gräfelfing, Germany

## Abstract

The nerve growth factor IB-like receptors (NR4A) are (neuro)protective transcription factors as part of the immediate early response and hold potential in various pathologies including neurodegeneration. Despite recent progress in NR4A ligand development, high-quality chemical tools to probe phenotypic effects of NR4A modulation are still rare and a selective agonist with strong efficacy is lacking. Here, we developed a potent and selective NR4A activator equipped with distinctly high agonist efficacy building on the scaffold of the DHODH inhibitor and Nurr1 agonist vidofludimus. We identified structural modifications conveying potency, selectivity and efficacy via systematic SAR elucidation and fusion of favored motifs eventually enabled multiparameter optimization to a chemical tool meeting highest quality criteria for biological studies.

## Introduction

**Figure 1:**
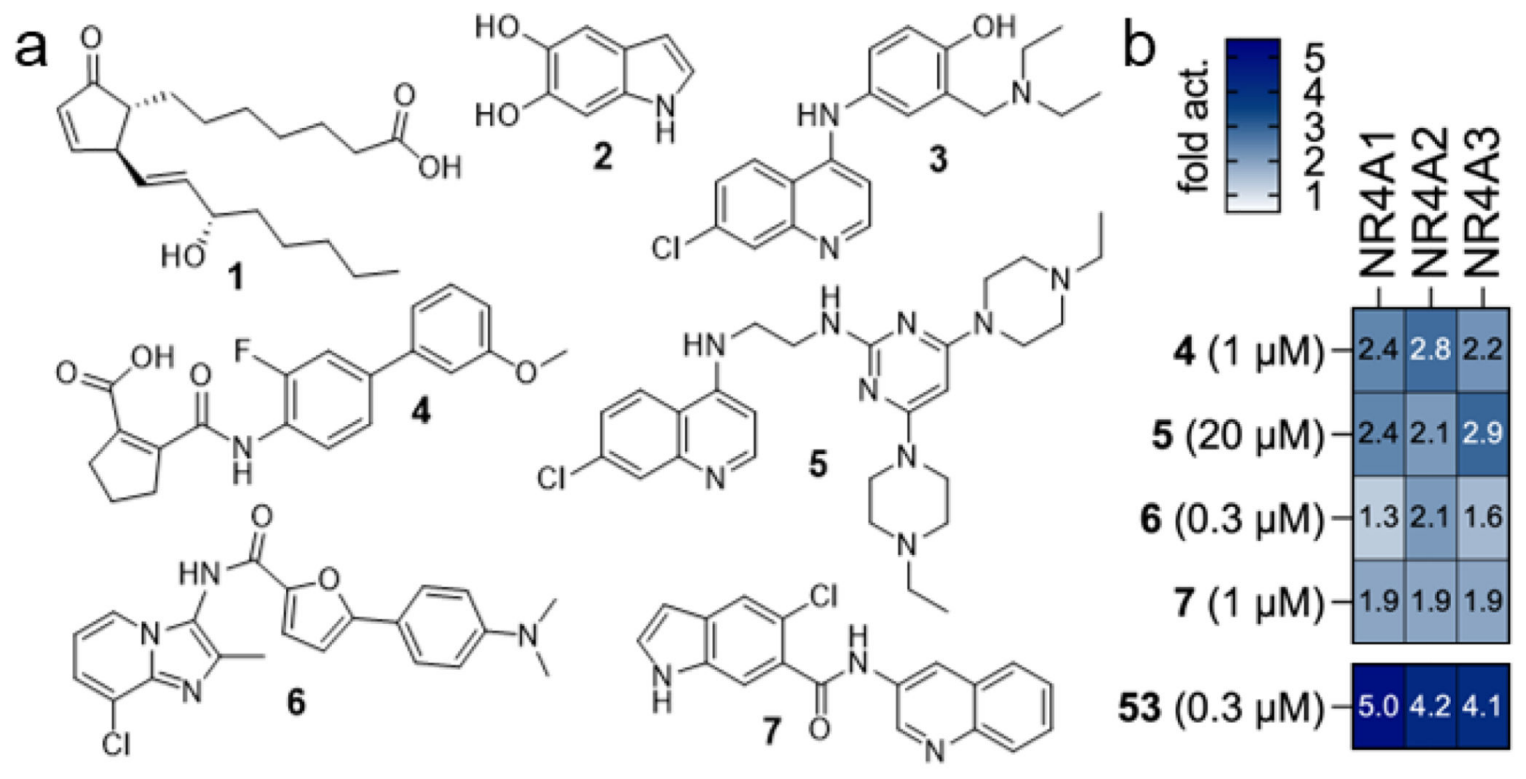
NR4A agonists. (a) Chemical structures of natural (**1, 2**) and synthetic (**3**-**7**) NR4A agonists. (b) Effects of **4**-**7** at ~EC_90_ on NR4A activity in uniform Gal4-hybrid reporter gene assays (data from ref.^8^; see also Table S1). Data for the optimized agonist **53** for comparison.

The nerve growth factor IB-like receptors (NR4A) are a small family of three ligand-sensing transcription factors with (neuro)protective properties as part of the immediate early response.^1–3^ The individual receptors Nur77 (NR4A1), Nurr1 (NR4A2) and NOR-1 (NR4A3) exhibit high structural similarity^3–5^ and act as constitutive transcriptional inducers also in absence of ligands^6,7^ but differ in expression patterns with Nurr1 displaying the strongest expression especially in brain tissue.^8,9^ Evidence for a protective role of Nurr1 in neurodegenerative pathologies^3,10–17^ such as Parkinson’s disease, dementia and multiple sclerosis has recently boosted interest in the receptor as a therapeutic target and sparked Nurr1 modulator development. Prostaglandins A1 (**1**; Figure 1a) and E1^18^ as well as the oxidized dopamine metabolite 5,6-dihydroxyindole (**2**)^19^ have been reported as natural ligands, forming covalent Michael adducts with Cys566 located behind helix 12 of the Nurr1 ligand binding domain (LBD). The antimalarial amodiaquine (**3**)^20^, several statins^21^ and the clinically studied dihydroorotate dehydrogenase (DHODH) inhibitor vidofludimus (**4**)^22^ were discovered as (experimental) drugs activating Nurr1 and dedicated Nurr1 ligand design efforts have yielded advanced synthetic modulators such as **5**-**7**.^23,24^ In addition to these validated ligands, literature also contains putative NR4A ligands whose activity has been questioned^8,25^ but which were used in previous biological studies on Nurr1. High-quality chemical tools are indispensable for probing protein function and validating therapeutic potential of new pharmacologies but reliance on weak or nonselective compounds of insufficient quality has led to misleading biological interpretations.^26^ To avoid deceptive results from application of improper chemical tools, further validated high-quality and highly annotated NR4A agonists are needed to advance this promising target family. Considering the available modulators^8^, there is a particular need for a potent and selective NR4A activator with higher activation efficacy than the established agonists to achieve strong responses in phenotypic models.

The Nurr1 agonist **4** exhibits favorably high activation efficacy compared to other potent agonists (Figure 1b) and emerges as a promising lead scaffold to develop a highly efficient NR4A activator also complying with highest quality criteria regarding potency and selectivity^27,28^. However, its activity on the original target DHODH had to be designed out and while our previous SAR studies on this chemotype^5,22,29^ indicated that potency and selectivity could be improved by structural modifications, conserving or even enhancing the 3.1-fold Nurr1 activation efficacy of **4** was challenging. Here, we report the broad and systematic exploration of all substructures of **4** for potency, selectivity and efficacy driving features. We obtained a high-quality chemical tool NR4A agonist with ~4.5-fold activation efficacy, nanomolar potency, ~40-fold selectivity over DHODH and a structurally matched negative control compound.

## Results & Discussion

We engaged on the systematic optimization of **4** with the objective to develop an NR4A activator exhibiting strong agonist efficacy and complying with community-agreed quality criteria (in vitro potency <100 nM, cellular on-target activity <1 µM and ≥30-fold selectivity, negative control available)^30,31^. For SAR profiling, we focused initially on the most abundant representative Nurr1 as the three NR4A receptor LBDs display strong homology and exhibit very similar ligand binding.^8^ We commenced SAR elucidation by stepwise deconstruction of **4** to identify key structural elements and promising regions for improvement (Table 1). First, we examined the importance of the sterically constrained cyclopentene in **4**. Both its reduction to the *cis*-substituted cyclopentane analogue **8** and simplification to the acyclic maleic acid analogue **9** resulted in a drastic loss in Nurr1 agonist potency and efficacy suggesting that the rigid cyclopentene was critical - possibly to correctly orient the carboxylic acid for binding.

Simplification of the trisubstituted biphenyl scaffold of **4** was more productive. Removal of the methoxy group on the terminal phenyl ring (**10**) or the fluorine atom on the central ring (**11**) were both tolerated with slight improvements in Nurr1 agonism, and even the unsubstituted analogue **12** lacking both the fluoro and the methoxy substituent displayed higher potency and efficacy than the lead **4**. Removal of the substituents additionally provided slight advances in selectivity over DHODH. Further simplification via complete elimination of the terminal anisol motif (**13**) disrupted Nurr1 agonism and mimicking the terminal ring by a bromine atom (**14**) displaying comparable size was insufficient to reinstate similar potency to the lead **4**. These SAR results thus indicated that the biphenyl scaffold was favored and that optimization of its substitution pattern offered potential to improve the Nurr1 agonist profile.

Hence, we focused our attention on the terminal anisol motif and broadly explored alternative substituent groups and positions (Tables 2&3). Preliminary SAR insights from our previous study^22^ had highlighted a propinyloxy motif (**15**) as valuable improvement of the methoxy group in **4** (Table 2) boosting both Nurr1 agonist potency and efficacy. However, selectivity of **15** over DHODH was insufficient. Extension of this residue to a 2-butin-1-yl group (**16**) hardly affected Nurr1 agonism and DHODH inhibition, while its reduction to the allyl ether **17** diminished agonist efficacy and preference over DHODH. Isosteric replacement of the alkyne (**15**) by a nitrile (**18**) disrupted Nurr1 agonism.

Dealkylation of the anisol **4** to the phenol **19** was tolerated with respect to potency, but in contrast to the alkynyl groups diminished efficacy. A chlorine atom (**20**) and especially a trifluoromethyl (**21**) substituent replacing the original methoxy group were favored in terms of potency but also caused a marked drop in efficacy. Fluorinated ethers (**22**-**26**) retained generally higher activation efficacy with the difluoromethoxy (**23**) and trifluoroethoxymethyl (**26**) exhibiting >6-fold Nurr1 activation. Deuteration of the most efficient difluoromethoxy motif in **24** enhanced Nurr1 agonist potency while reducing activation efficacy, consistent with our previous study^22^.

A benzyl ether (**27**) was favored to replace the methoxy group (**4**) and enhanced potency by tenfold while efficacy dropped slightly. Phenoxy (**28**) and phenyl motifs (**29**) were also tolerated but inferior to the benzyl ether (**27**) suggesting preference for flexible groups. Inactivity of the rigid *N*-phenylcarboxamide analogue **30** on Nurr1 supported this hypothesis. A more polar isoxazolylmethylether (**31**) to replace the favored benzyloxy group (**27**) markedly reduced potency on Nurr1 despite increased efficacy indicating that polarity was not preferred in this region.

Overall, extended hydrophobic ethers like the alkynyloxy (**15, 16**) and fluoroalkoxy (**22**-**26**) motifs thus emerged as favored to achieve both strong potency and efficacy in Nurr1 activation. However, these alternative ethers were also tolerated by DHODH and no efficacy enhancing group simultaneously offered sufficient selectivity. Nevertheless several motifs appeared promising for combination with selectivity driving features in fused derivatives.

As modifications of the methoxy motif retaining the original regiochemistry of **4** were insufficient to obtain the desired Nurr1 agonist profile, we moved our attention to alternative substituent positions and double substitution on the terminal ring (Table 3). The 3-methoxy group of **4** was also tolerated in the 4-position (**33**) and preferred in the 2-position (**32**) with improvements in potency, efficacy and selectivity of **32** over **4**. The trifluoromethoxy derivatives (**22, 34** and **35**) displayed a similar preference for 2- and 3-substitution but with overall lower activation efficacy. In line with these results, systematic evaluation of double substitution patterns revealed improved activation efficacy for all dimethoxy derivatives (**36**-**40**) over **4** and highlighted the 2,3- (**36**) and 2,4- (**38**) dimethoxy analogues as potent and highly efficient (4.7-to 4.9-fold activation) Nurr1 agonists. Double substitution in 2,5-positions (**37**) resulted in lower efficacy and the 3,4- (**39**) and 3,5- (**40**) dimethoxy analogues displayed lower potency.

Building on 2,3-disubstitution (**36**) as most favored to achieve potent Nurr1 agonism with strong efficacy, we evaluated alternative patterns in these positions. The 2,3-dichloro analogue **41** displayed remarkable 11 nM potency but efficacy dropped substantially. The 2-trifluoromethoxy-3-chloro derivative **42** revealed a similar potency with improved selectivity over DHODH (45-fold) but failed to exhibit enhanced efficacy. Fusing the 2,3-substituents in a ring in the difluorobenzodioxole **43**, however, provided the desired activity profile with high potency (EC_50_ 100 nM) and strong efficacy (5-fold activation) and thus emerged as most promising modification of the anisol motifin **4**.

Next, we assessed the central ring in the amidobiphenyl scaffold and its substitution pattern for modifications that could improve selectivity over DHODH and enhance Nurr1 agonist potency and efficacy (Table 4) using the original anisol of **4** as terminal motif. Removal of the fluorine of **4** in **11** had been tolerated with little impact on Nurr1 agonism. Its replacement by a methyl group (**44**), in contrast, increased potency by threefold and improved preference over DHODH. A methoxy group (**45**) was even more favored while the trifluoromethyl derivative **46** displayed similar activity as the unsubstituted analogue **11**. 3-Chloro substitution (**47**) achieved the strongest improvement in potency and was sixfold more active than the lead **4**. Simultaneously, selectivity of **47** over DHODH improved markedly compared to **4** highlighting the 3-chloro substituent as promising for combination with optimized terminal motifs.

Moving the 3-fluoro substituent of **4** to the 2-position (**48**) was also strongly favored in terms of potency and additionally improved preference over DHODH. Combination of the potency-driving fluorine atom in 2-position with the favored 3-chloro substituent in **49** did not provide improvement over the 3-chlorophenyl analogue **47**, however. Eventually, we explored the replacement of the central aromatic ring but even the conservative change to the bioisosteric thiophene (**50**) mediated an almost tenfold drop in potency compared to the corresponding phenyl analogue **11**. Despite a considerable improvement in activation efficacy of **50**, alternative aromatic systems hence did not seem promising to retain potent Nurr1 agonism.

The systematic exploration of all three substructures of **4** for potency, efficacy and selectivity driving features revealed promising modifications in the central and terminal phenyl ring while retaining the original cyclopentene carboxylic acid motif appeared critical. Notably, the SAR indicated minor changes that conserved the drug-like properties of the lead as sufficient to achieve potent, efficient and selective Nurr1 agonism. Building on these results, we next followed a combinatorial design approach to explore whether the effects of favored modifications were additive (Table 5).

While 3-chloro substitution clearly emerged as most preferred in the amidobenzene residue, several promising modifications were discovered in the terminal anisol moiety. Fluoroalkoxy motifs (**22**-**26**) and especially a trifluoromethoxy group (**22**) achieved potent Nurr1 agonism with high efficacy. Combination of the 3’-trifluoromethoxyphenyl group with the central 3-chlorobenzene (**47**) was not productive, however. The resulting compound **51** displayed potent Nurr1 agonism (EC_50_ 120 nM) and sufficient selectivity over DHODH (44-fold) but lacked efficacy (2.2-fold). The 2,3-dimethoxyphenyl derivative **36** had emerged as another potent Nurr1 agonist with particularly strong efficacy. Its fusion with the central 3-chlorobenzene (**52**) was tolerated in terms of potency (EC_50_ 110 nM) and adequately conserved activation efficacy (3.3-fold) but selectivity over DHODH was insufficient (15-fold). Only combination of the central 3-chlorobenzene and the terminal difluorobenzodioxole (**43**) in **53** evolved as additive in terms of selectivity over DHODH (47-fold) while conserving high potency (EC_50_ 92 nM) and strong efficacy (4.4-fold).

**Figure 2:**
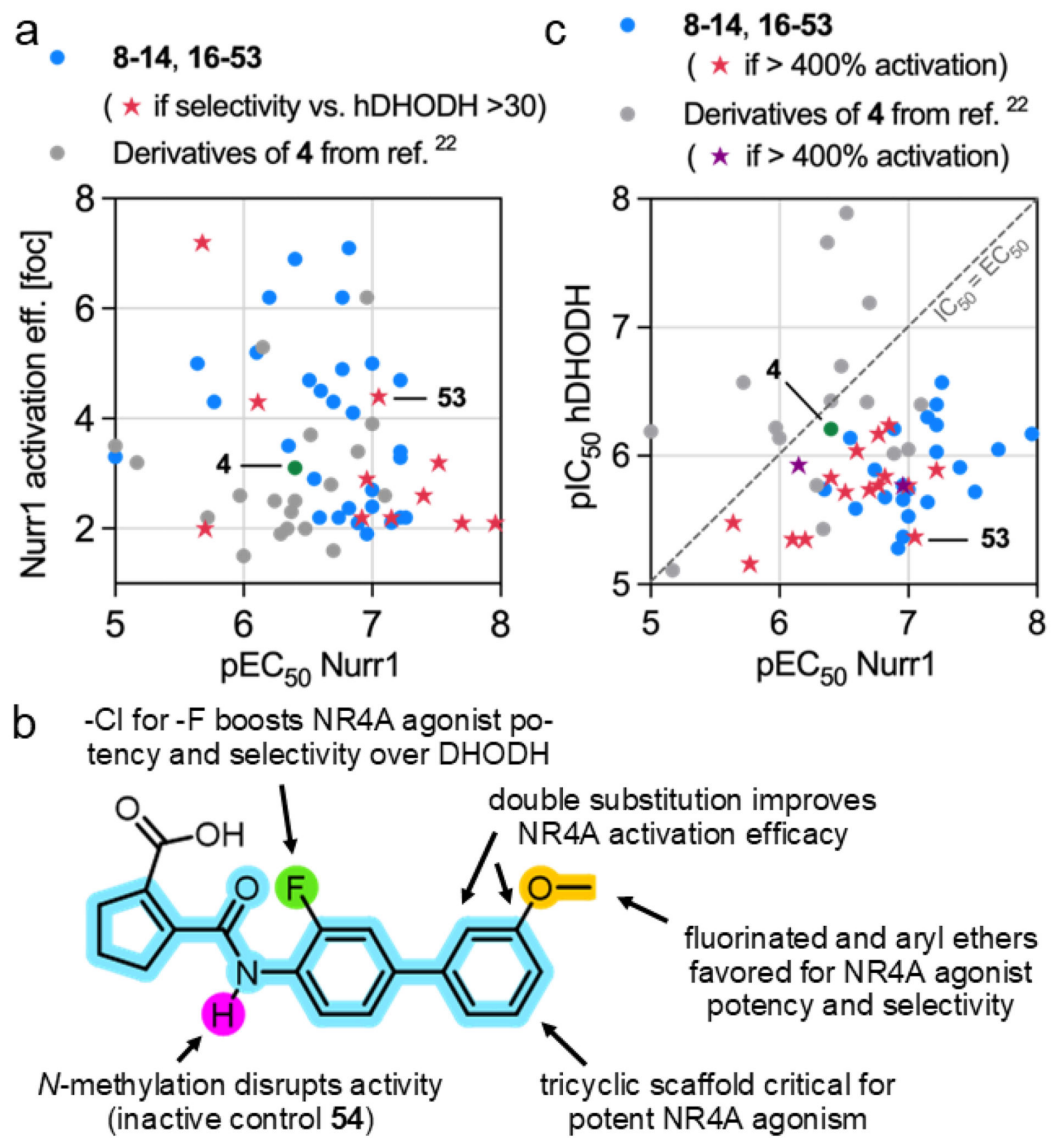
SAR summary of NR4A agonists derived from **4**. (a) Correlation of potency and activation efficacy on Nurr1 (NR4A2). (b) Summary of key structural modifications driving potency, efficacy and selectivity. (c) Correlation of Nurr1 agonist potency and DHODH inhibitor potency. Plots in (a) and (c) show compounds from this study and from ref.^22^.

The multiparameter optimization of the NR4A agonist **4** for potency, efficacy and selectivity presented as considerable challenge especially with respect to achieving strong activation efficacy. The latter did not correlate with potency (Figure 2a), tended to drop with minor structural modifications, and could only be conserved with few potency and selectivity enhancing motifs (Figure 2b). Nevertheless, **53** eventually emerged as Nurr1 agonist complying with the desired potency, efficacy and selectivity profile (Figure 2c).

**53** displayed similar potency and activation efficacy on Nur77 and NOR-1 as on Nurr1 in line with the envisioned pan-NR4A agonist profile (Table 6, comparison with other NR4A ligands in Table S1). Moreover, full-length Nurr1 reporter gene assays demonstrated activation of the human response elements for the monomer (NBRE), homodimer (NurRE) and RXR-heterodimer (DR5) by **53** with consistent potency and high efficacy (Table 6) corroborating NR4A agonism on the native receptors. Binding of **53** was validated by isothermal titration calorimetry (ITC) using recombinant NR4A1 LBD protein (Table 6, Figure S1).

To equip **53** with a negative control compound for more confident application in phenotypic studies, we capitalized on our previous SAR insights^22^ for the scaffold which had revealed a loss of Nurr1 agonism for the *N*-methyl amide of **4**.

**Figure 3:**
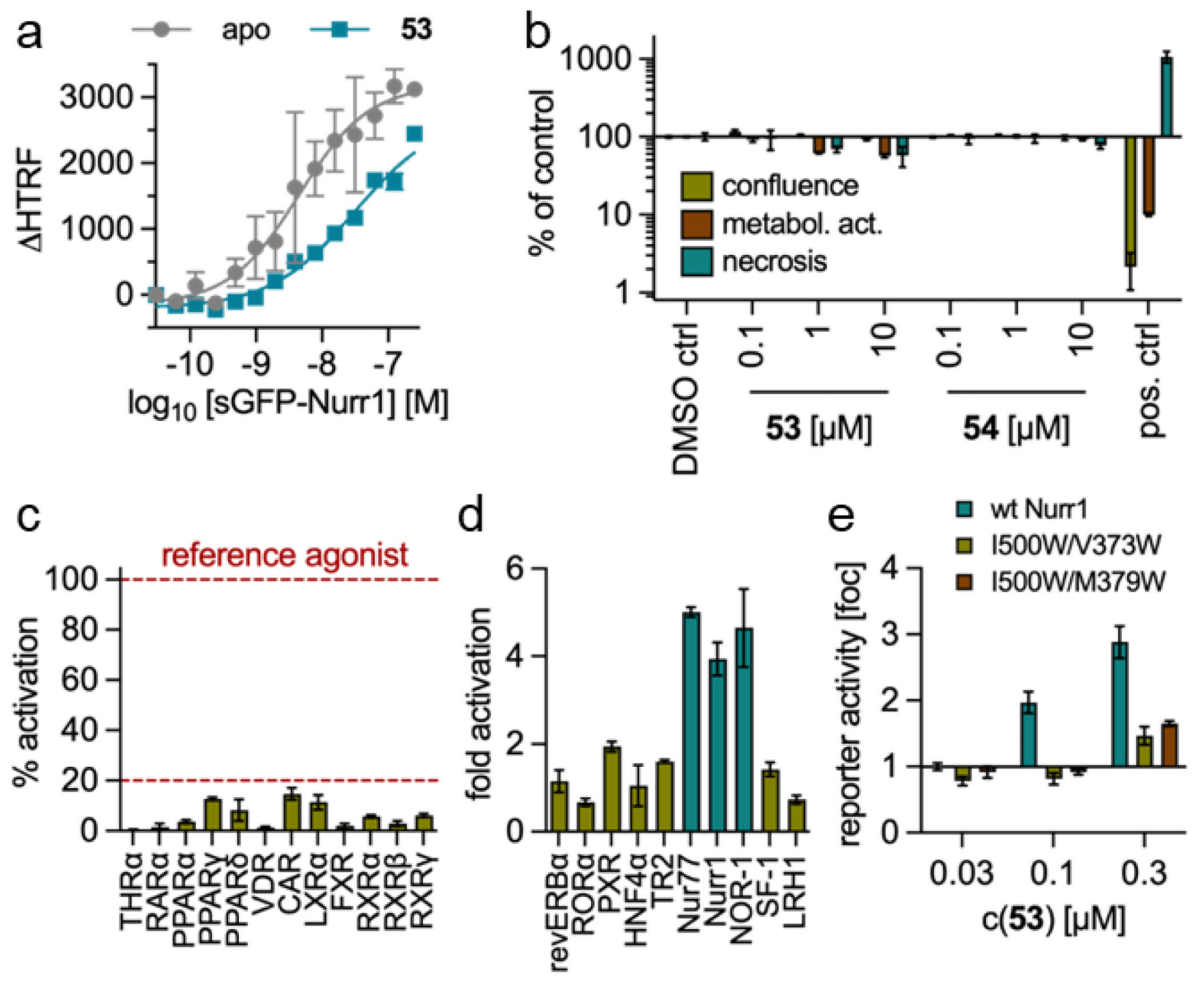
In vitro characterization of the NR4A agonist **53** and the inactive reference **54**. (a) **53** diminished Nurr1 homodimer formation in an HTRF based assay by ~9-fold. Tb^3+^-cryptate (donor) labeled Nurr1 LBD was titrated with sGFP (acceptor) labeled Nurr1 LBD in absence or presence of **53** (10 µM). Data are the mean±SD ΔHTRF; n=3. (b) **53** and **54** displayed no cytotoxic effects over 24 h in a multiplex toxicity assay monitoring confluence, metabolic activity and necrosis in HEK293T cells. Bexarotene (100 µM) was used as positive control. All data are normalized to the DMSO ctrl and are the mean±SD; n=4. (c, d) Selectivity profiling of **53** (3 µM) on nuclear receptors including classical lipid-activated (c) and constitutively active (d) receptors. Data are the mean±SD; n=3. (e) **53** displayed markedly reduced activation of the Nurr1 mutants I500W/V373W and I500W/M379W which also abolished the agonist activity of the lead **4**. Data are the mean±SD reporter activity normalized to DMSO ctrl, n=3.

Methylation of the amide linker of **53** in **54** also abolished activity on all NR4A receptors up to 100 µM corresponding to at least 1000-fold reduced potency. **54** thus qualified as structurally matched negative control for the NR4A agonist **53**. Mechanistic studies on Nurr1 have suggested that NR4A ligands mediate activation by releasing the transcriptionally active monomer from dimers^5,35^. Correspondingly, homogeneous time-resolved fluorescence resonance energy transfer (HTRF) based observation of Nurr1 homodimerization revealed markedly decreased dimer formation (~9-fold) in presence of **53** (Figure 3a) indicating that the compound followed the proposed activation mechanism.

In addition to orthogonally validated on-target potency, selectivity and lack of unspecific toxic effects are key features for high-quality chemical tools. Hence, we tested **53** and the negative reference **54** in a multiplex toxicity assay monitoring confluence, metabolic activity and necrosis in HEK293T cells, which revealed no relevant cytotoxic activity for both compounds up to 10 µM (Figure 3b). Moreover, evaluation of **53** (3 µM) in uniform Gal4 hybrid reporter gene assays for modulation of nuclear receptors demonstrated selective NR4A activation (Figure 3c,d). The cytotoxicity and selectivity profile thus supported suitability of **53** as chemical tool.

We have previously located the presumable binding site of the lead **4** in the Nurr1 LBD at a surface pocket formed by helices 1, 5, 7, and 8 using molecular dynamics simulation and mutagenesis.^5^ Blocking this pocket with bulky residues in the Nurr1 double mutants I500W/V373W and I500W/M379W led to a loss of activation by **4** while fluvastatin retained agonism also on the mutants supporting a specific effect. Evaluation of **53** on these mutants in the hybrid reporter gene assay setting revealed markedly diminished reporter activation (Figure 3e) indicating that the compound bound to the same site as **4**.

**Figure 4:**
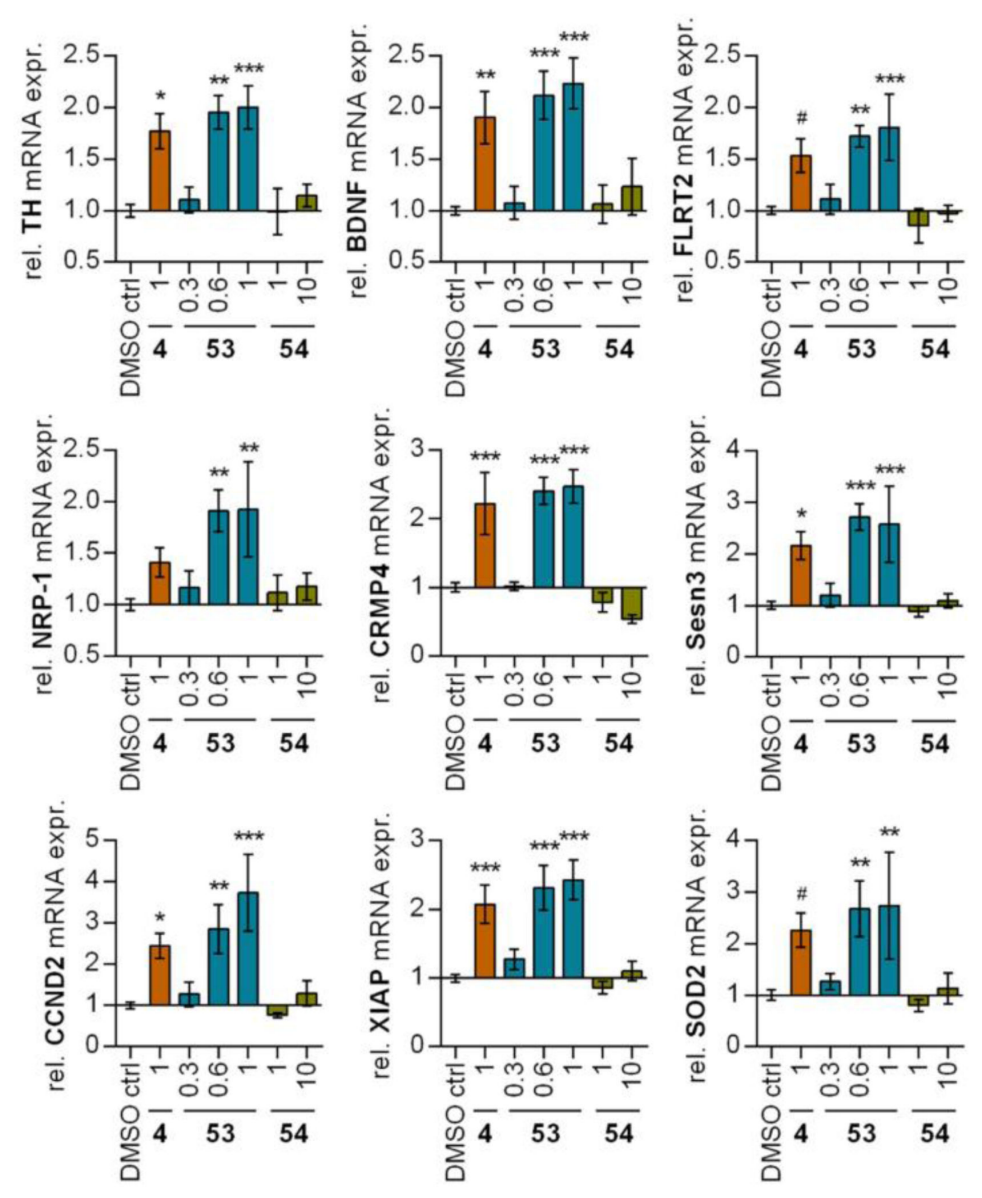
Effects of **4, 53** and **54** on gene expression in rat dopaminergic neurons (N27). The NR4A agonist **53** induced all studied NR4A-regulated genes in a dose-dependent manner and with higher efficacy than the lead **4** (p < 0.01 for **53** (1 μM) vs **4** (1 μM); ANOVA over all studied genes). The inactive reference **54** had no effect. Data are the mean±S.E.M. relative mRNA expression vs. DMSO ctrl analyzed by the ΔΔCt method with GAPDH as reference gene; n=5; ^#^ *p*<0.1, * *p*<0.05, ** *p*<0.01, *** *p*<0.001 (vs. DMSO ctrl, ANOVA with Dunnett’s test).

To validate cellular target engagement of **53** in a native setting and corroborate suitability as chemical tool, we studied the compound’s impact on gene expression in NR4A expressing rat dopaminergic neurons (N27, Figure 4)^36^. In addition to the dopaminergic neuron marker tyrosine hydroxylase (TH), NR4A2 activation in these cells has been shown to induce neuroprotective genes such as brain-derived neurotrophic factor (BDNF), fibronectin leucine rich transmembrane protein 2 (FLRT2), neuropilin-1 (NRP-1), collapsin response mediator protein 4 (CRMP4), sestrin 3 (Sesn3), cyclin D2 (CCND2), X-linked inhibitor of apoptosis protein (XIAP), and superoxide dismutase 2 (SOD2).^23,29^ Evaluation of these NR4A2 targets revealed strong induction after treatment with **53** in a dose-dependent manner. The effects of **53** were generally stronger compared to the lead **4** (*p* < 0.01 for **53** (1 µM) vs. **4** (1 µM); ANOVA over all studied genes) corroborating enhanced efficacy of **53**. The inactive reference **54** had no significant effect on any of the studied genes supporting NR4A-mediated effects of **53** and confirming **54** as suitable negative control compound.

## Conclusion

Despite strong evidence for protective properties of NR4A receptors, e.g., in the CNS^17^ and the liver^37^, as well as a role in T-cell exhaustion and immune escape^38^, high-quality chemical tools to study the pharmacological modulation of this transcription factor family are still rare.^8^ Here we aimed to expand this collection with a potent and selective agonist providing distinctly high activation efficacy for strong responses in phenotypic models. Using the DHODH inhibitor and Nurr1 agonist vidofludimus^22^ (**4**) as lead, systematic SAR elucidation revealed minor structural modifications conferring improved potency, selectivity and/or efficacy. Although the SAR was mostly not additive especially with respect to agonist efficacy, multiparameter optimization eventually succeeded by combining a terminal 2,2-difluorobenzodioxole (**43**) which was highly favored in terms of potency and efficacy with chlorination of the central benzene (**47**) to enhance selectivity over DHODH. The resulting NR4A agonist **53** retains the favorable drug-like properties of the lead, meets community agreed quality criteria^27^ and mediated robust induction of NR4A regulated genes in dopaminergic neurons supporting its suitability as chemical tool.

### Chemistry

**Scheme 1:**
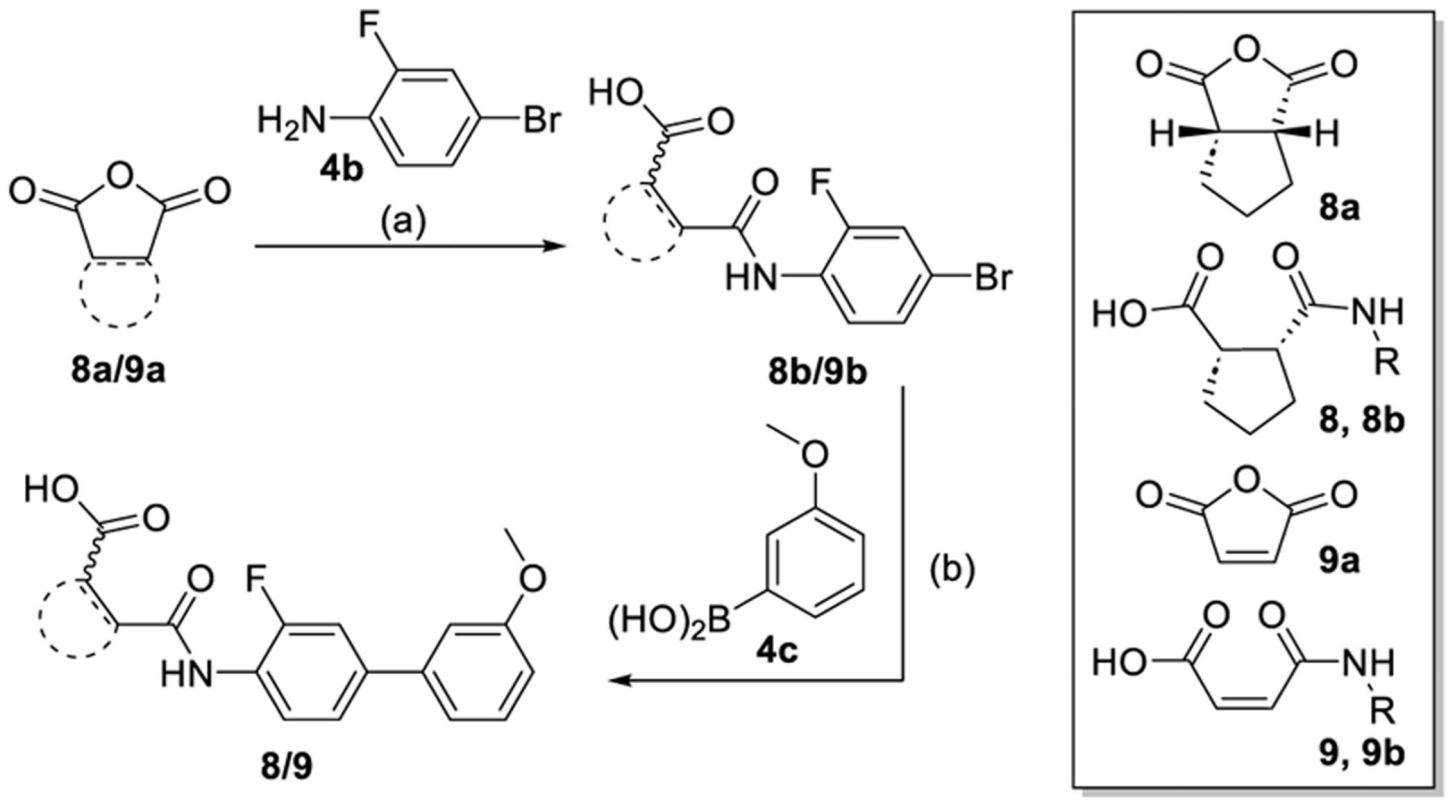
Synthesis of 8 and 9^a^ ^a^ Reagents and Conditions: (a) CH_2_Cl_2_, rt, 16 h, 61-97%; (b) XPhos-Pd-G2, Cs_2_CO_3_, toluene/EtOH/H_2_O (3:2:1), 90 °C, 16 h, 32-45%.

**Scheme 2:**
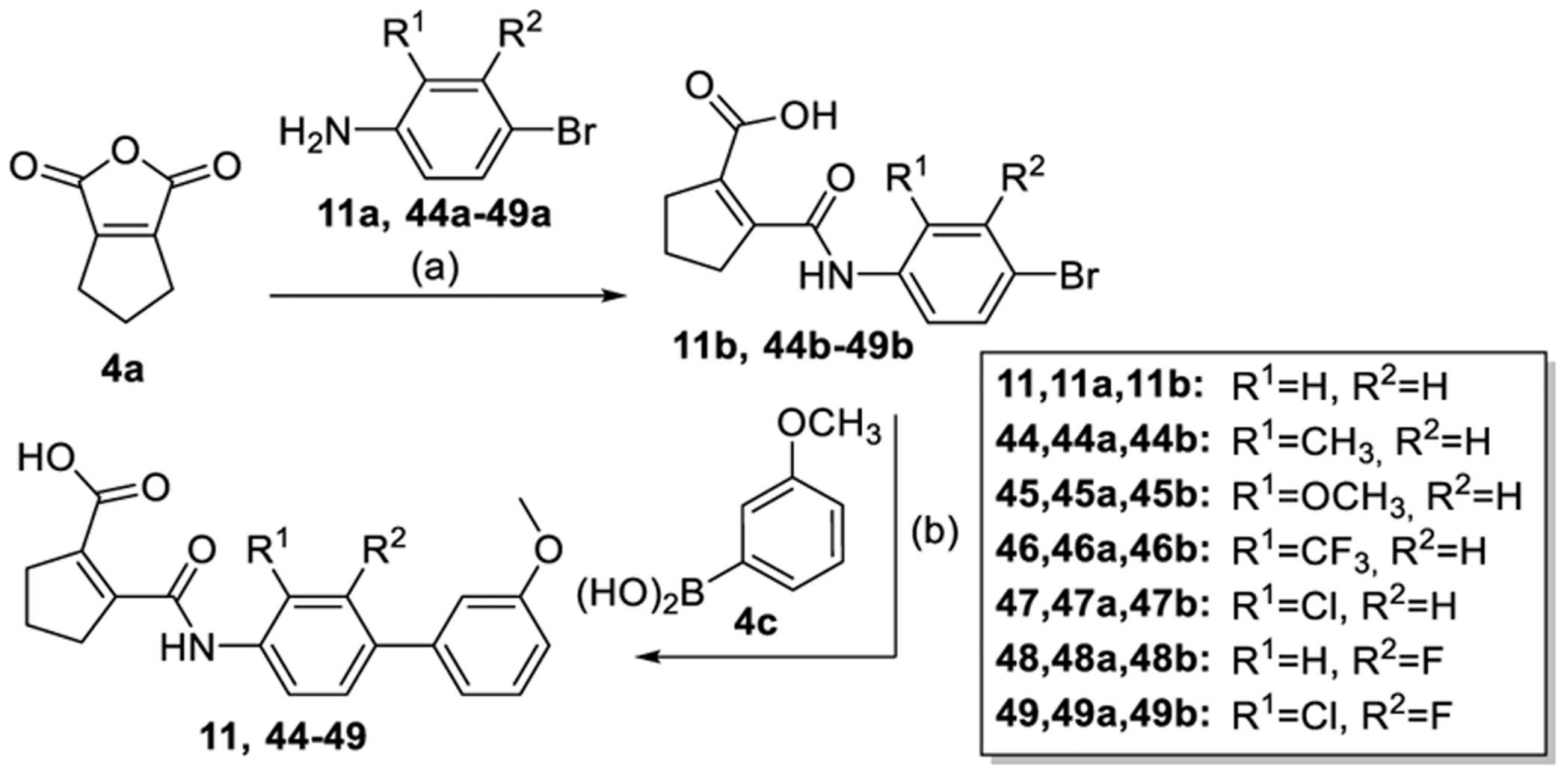
Synthesis of 11 and 44-49^a^ ^a^ Reagents and Conditions: (a) CH_2_Cl_2_, rt, 16 h, 49-100%; (b) XPhos-Pd-G2, Cs_2_CO_3_, toluene/EtOH/H_2_O (3:2:1), 90 °C, 16 h, 15-92%.

**Scheme 3:**
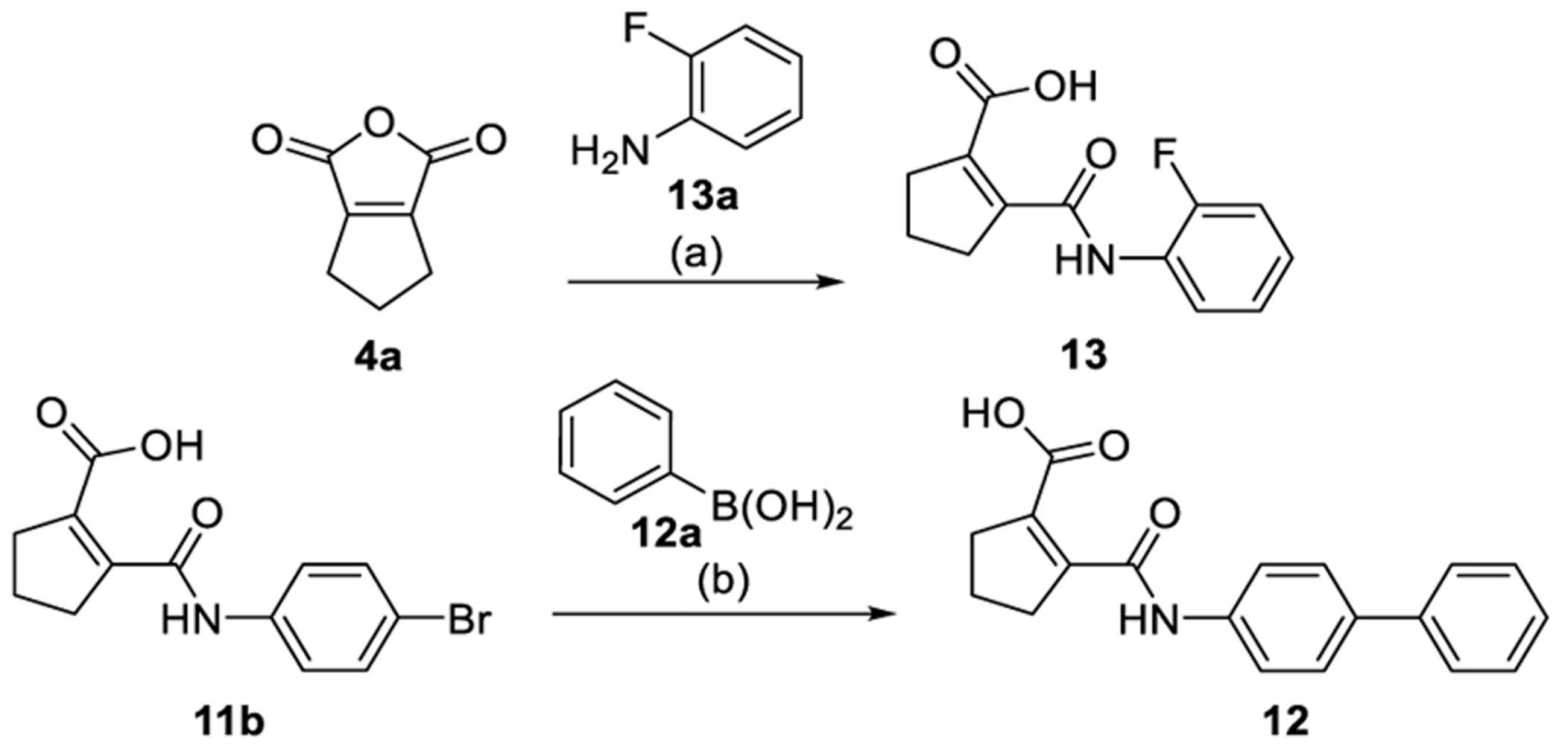
Synthesis of 12 and 13^a^ ^a^ Reagents and Conditions: (a) CH_2_Cl_2_, rt, 16 h, 78%; (b) XPhos-Pd-G2, Cs_2_CO_3_, toluene/EtOH/H_2_O (3:2:1), 90 °C, 16 h, 50%.

**Scheme 4:**
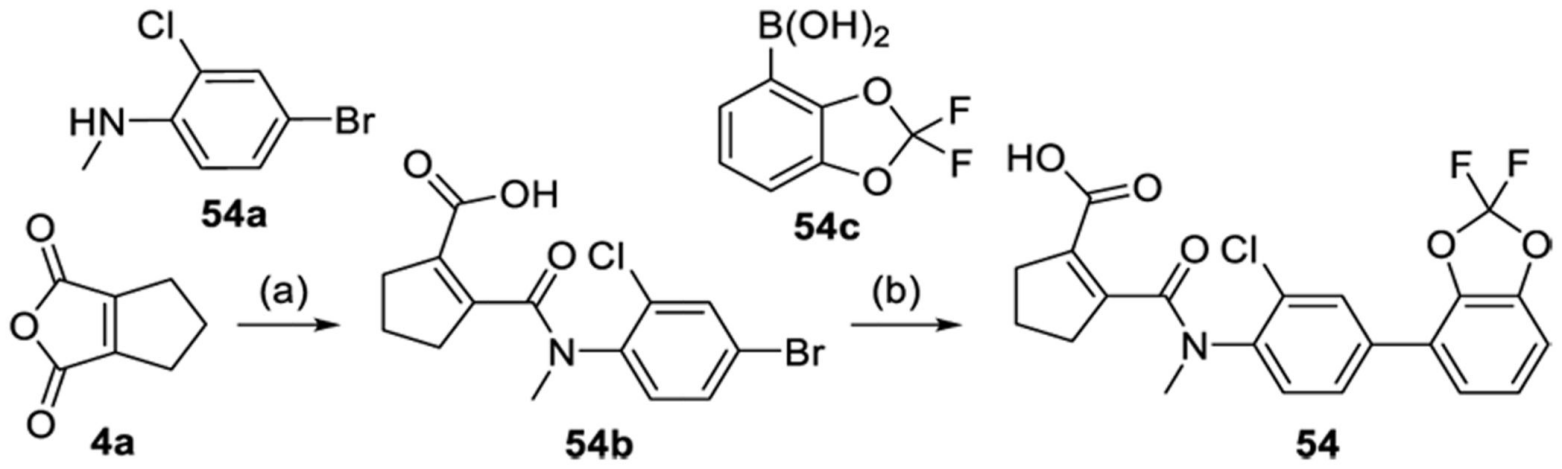
Synthesis of the inactive control 54^a^ ^a^ Reagents and Conditions: (a) CH_2_Cl_2_, rt, 16 h, 12%; (b) Pd(PPh_3_)_4_, Na_2_CO_3_, toluene/EtOH/H_2_O (3:2:1), 60 °C, 6 h, 32%.

**Scheme 5:**
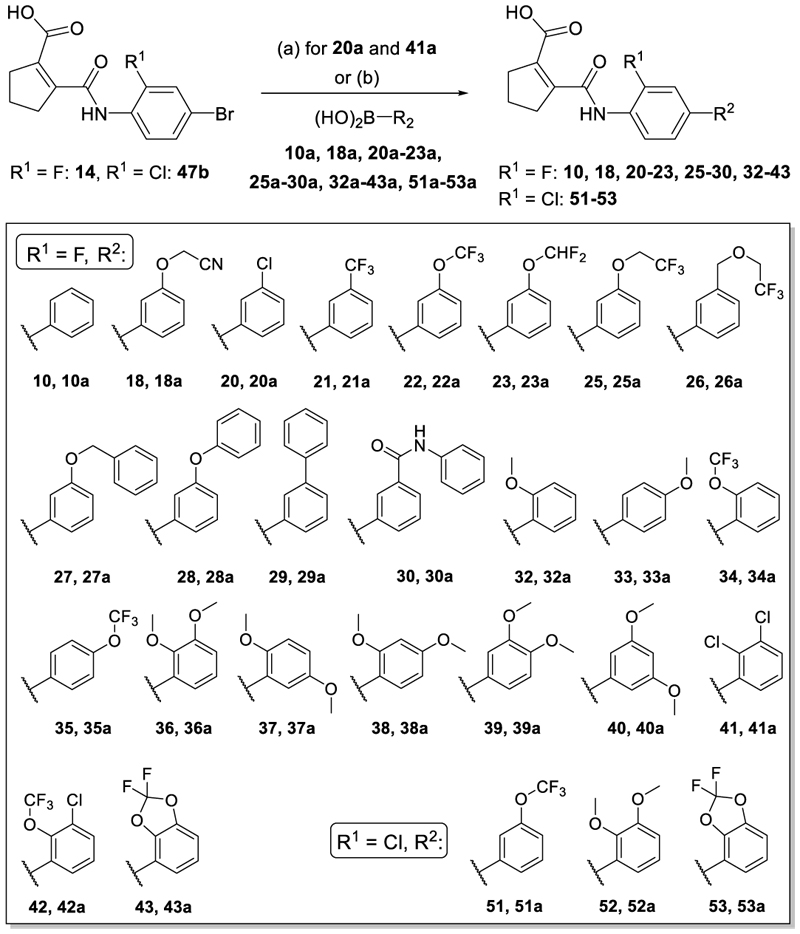
Synthesis of 10, 18, 20-23, 25-30, 32-43 and 51-53^a^ ^a^ Reagents and Conditions: (a) Pd(PPh_3_)_4_, Na_2_CO_3_, toluene/EtOH/H_2_O (3:2:1), 60 °C, 6 h, 36-82%; (b) XPhos-Pd-G2, Cs_2_CO_3_, toluene/EtOH/H_2_O (3:2:1), 90 °C, 16 h, 16-93%.

The synthesis of compounds **4, 14** and **15** has been described previously.^22^ The tricyclic scaffold of most compounds (**8**-**13, 18, 20-23, 25-30, 32**-**49, 51**-**54**) was assembled in a two-step sequence with variable order involving (i) amide formation by the reaction of an anhydride with an aniline and (ii) a Suzuki-Miyaura coupling of an aryl bromide with a phenylboronic acid. The corresponding building blocks, reaction sequences and conditions are presented in Schemes 1-5.

**Scheme 6:**
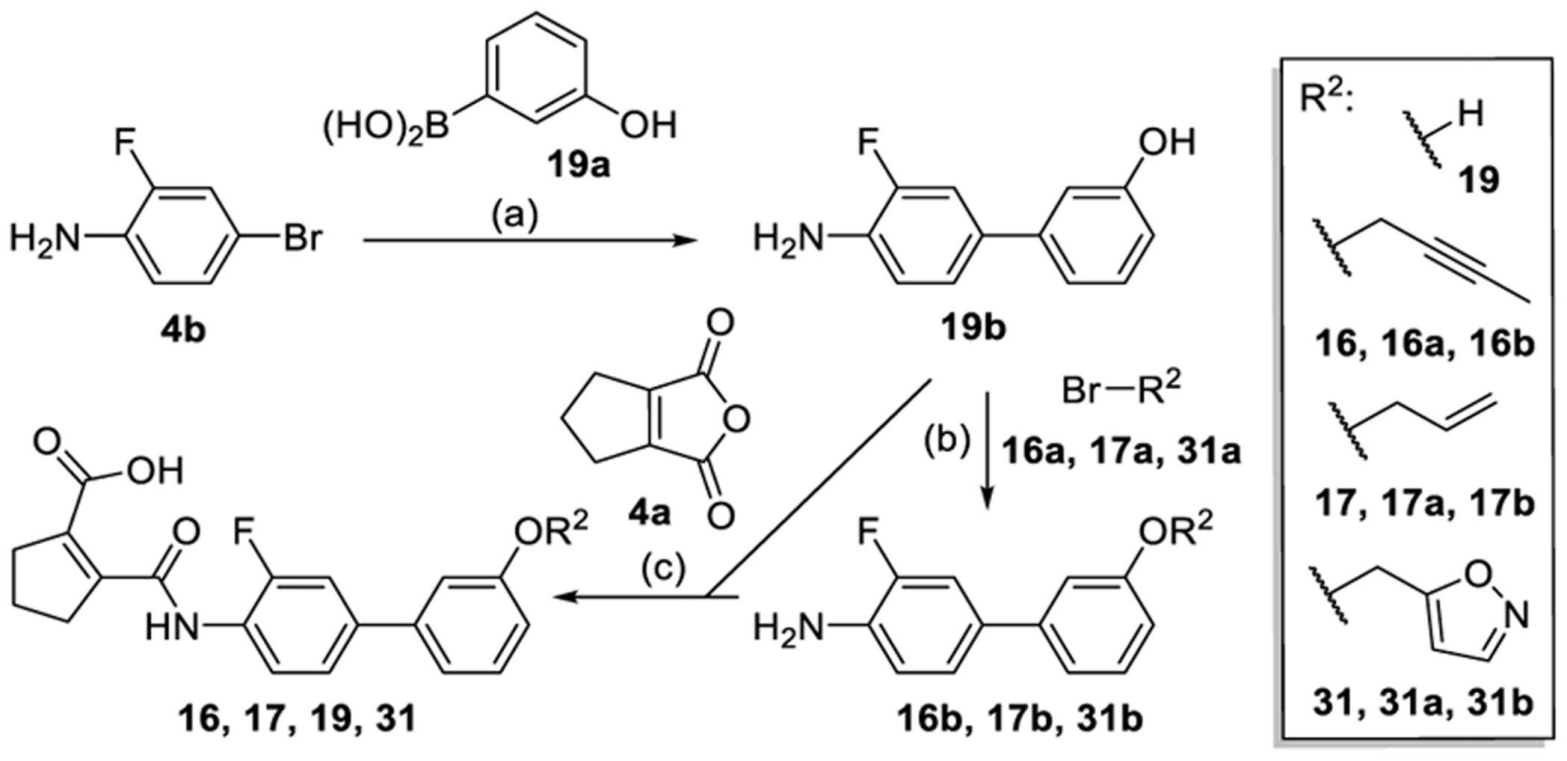
Synthesis of 16, 17, 19 and 31^a^ ^a^ Reagents and Conditions: (a) XPhos-Pd-G2, Cs_2_CO_3_, toluene/EtOH/H_2_O (3:2:1), 90 °C, 16 h, 90%; (b) *t*-BuOK, THF, rt, 16 h, 18-96%; (c) CH_2_Cl_2_, rt, 16 h, 30-61%.

**Scheme 7:**
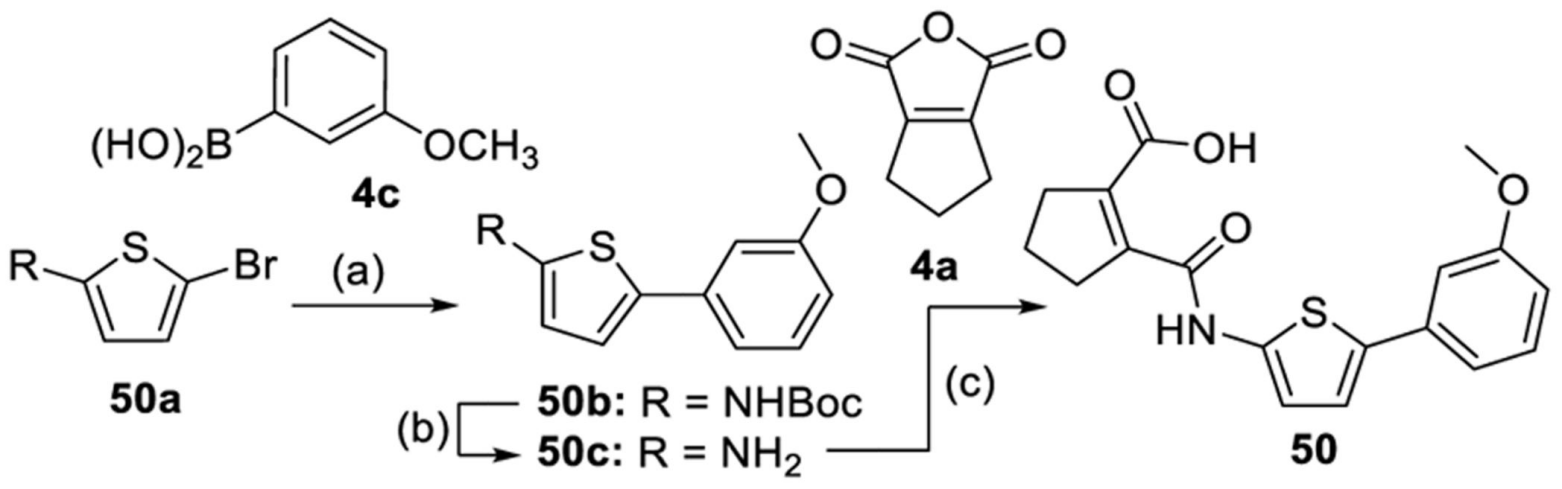
Synthesis of 50^a^ ^a^ Reagents and Conditions: (a) XPhos-Pd-G2, Cs_2_CO_3_, toluene/EtOH/H_2_O (3:2:1), 90 °C, 16 h, 82%; (b) TFA, CH_2_Cl_2_, rt, 4 h, 46%; (c) CH_2_Cl_2_, rt, 16 h, 46%.

Compounds **16, 17, 19** and **31** (Scheme 6) were prepared via Suzuki-coupling of 4-bromo-2-fluoroaniline (**4b**) with 3-hydroxyphenylboronic acid (**19a**) to the biphenyl **19b** followed by Williamson ether synthesis with allyl bromide (**16a**), 1-bromo-2-butyne (**17a**) or 5-bromomethyl-isoxazole (**31a**) to afford **16b, 17b** and **31b**. The anilines **16b, 17b, 19b** and **31b** were then reacted with 1-cyclopentene-1,2-dicarboxylic acid anhydride (**4a**) to yield the amides **16, 17, 19** and **31**. The thiophene derivative **50** (Scheme 7) was obtained via Suzuki-coupling of *tert*-butyl (5-bromothiophen-2-yl)carbamate (**50a**) with 3-methoxyphenylboronic acid (**4c**) to **50b**, cleavage of the *N*-Boc group (**50c**) and subsequent amide formation with 1-cyclopentene-1,2-dicarboxylic acid anhydride (**4a**).

**Scheme 8:**
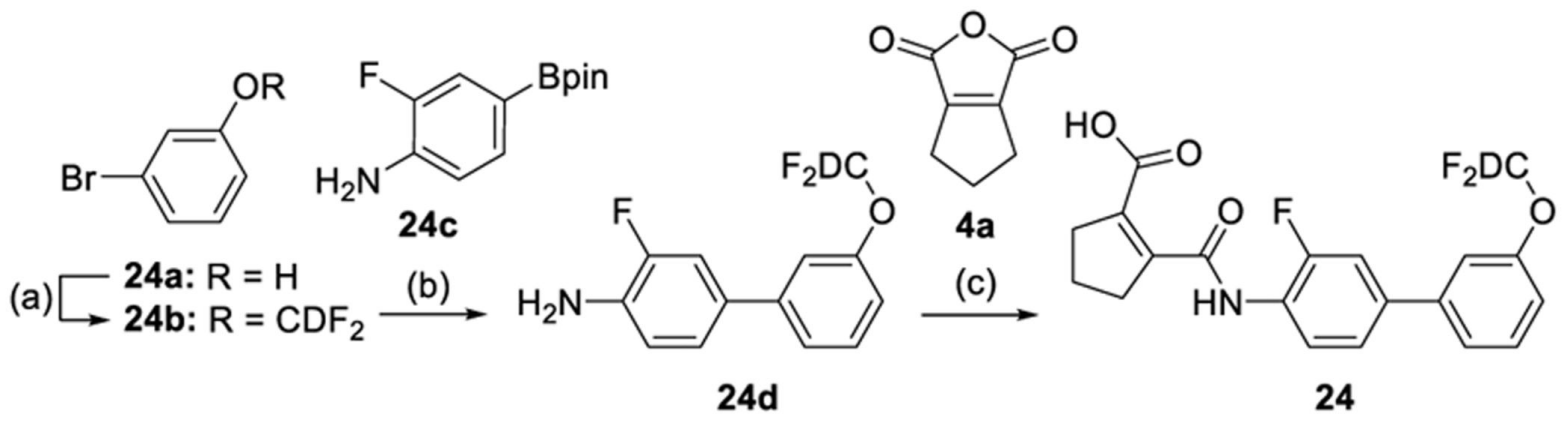
Synthesis of 24^a^ ^a^ Reagents and Conditions: (a) NaH, THF, 0 °C, 30 min, then D_2_O, 0 °C, 10 min, then diethyl (bromodifluoromethyl)phosphonate, rt, 30 min, 35%; (b) Pd(dppf)Cl_2_, Na_2_CO_3_, 1,4-dioxane/H_2_O (10:1), 90°C, 2 h, 88%; (c) CH_2_Cl_2_, 40°C, 4 h, 65%.

Compound **24** was prepared in three steps (Scheme 8) starting with difluoromethylation and deuteration of 3-bromophenol (**24a**) followed by Suzuki coupling with 4-amino-3-fluorophenylboronic acid pinacol ester (**24c**) and amide formation with 1-cyclopentene-1,2-dicarboxylic acid (**4a**).

## Experimental Procedures

### Chemistry

#### General

All chemicals and solvents were of reagent grade, purchased from commercial sources (e.g., Sigma-Aldrich, Enamine and BLDpharm) and used without further purification. All reactions were conducted in oven-dried glassware under argon atmosphere and in absolute solvents. Other solvents, especially for work-up procedures, were of reagent grade or purified by distillation (*iso*-hexane, cyclohexane, EtOAc, EtOH). Reactions were monitored by thin layer chromatography (TLC) on TLC Silica gel F_254_ aluminum sheets by Merck and visualized under ultraviolet light (254 nm) or by in-process LC/MS. Purification by column chromatography (CC) was performed on an Interchim puriFlash XS520Plus system (Advion, Ithaca, NY) using high-performance spherical silica columns (SIHP, 30 µM) by Interchim and a gradient of hexane, 10-100% EtOAc). Reversed-phase column chromatography (RP-CC) was performed on a puriFlash 5.250 system (Advion) using C18HP columns (SIHP, 15 µM) by Interchim and a gradient of H_2_O, 10-100% ACN or MeOH. Preparative HPLC was performed on a puriFlash 5.250 system using a utisphere strategy C18-HQ prep-LC column (5 µM, 150 × 30 mm) and a gradient of H_2_O, 10-100% ACN or MeOH (HPLC gradient grade). Mass spectra were obtained on a puriFlash-CMS system (Advion) using atmospheric pressure chemical ionization (APCI). High resolution MS (HRMS) spectra were obtained with a Finnigan LTQ FT instrument (Thermo Fisher Scientific) using electrospray ionization (ESI) or electron ionization (EI). ^1^H and ^13^C NMR spectra were recorded at 25 °C on Bruker Avance III HD 400, or Avance III HD 500 spectrometers (Bruker Corporation, Billerica, MA, USA). Chemical shifts are reported in δ values (ppm), coupling constants (J) in hertz (Hz). Compound purity was determined by quantitative ^1^H NMR (qH NMR) according to the method described by Pauli et al.^39^ with internal calibration. To ensure accurate determination of peak area ratio, the qH NMR measurements were conducted under conditions allowing for complete relaxation. Ethyl 4-(dimethylamino)benzoate (LOT#BCCC6657, purity 99.63%) was used as internal standard. All final compounds for biological evaluation had a purity of >95% according to qNMR.

#### General procedure for amide coupling of anilines with carboxylic acid anhydrides (GP1)

The respective cyclic anhydride (1.0 eq) and the respective aniline (1.0 eq) were dissolved in CH_2_Cl_2_ and stirred at rt for 16 h. The mixture was diluted with aqueous HCl (10%) and extracted with CH_2_Cl_2_ three times. The combined organic layer was dried over Na_2_SO_4_, filtered, concentrated under reduced pressure and purified as described in the respective example (if necessary).

#### General procedure for Suzuki Miyaura coupling using XPhos-Pd-G2 (GP2)

The respective phenylboronic acid derivative (1.1 eq), the respective arylbromide (1.0 eq) and Cs_2_CO_3_ (3.0 eq) were evacuated for 10 minutes. A solvent mixture of toluene/EtOH/H_2_O (3:2:1) was degassed by the freeze-pump-thaw method (3x) and added to the reactants under argon to afford a 40-50 mM reaction mixture. Then XPhos-Pd-G2 (0.1 eq) was added and the reaction was stirred at 90 °C for 16 h. After cooling to rt, EtOAc and H_2_O were added and the mixture was filtered through Celite. All solvents were removed under reduced pressure and aqueous HCl (10%) was added to the resulting residue. The aqueous layer was extracted with EtOAc (3x). The organic layers were combined, dried over Na_2_SO_4_, filtered and evaporated. The crude product was purified as described in the respective example.

#### General procedure for Williamson ether synthesis (GP3)

To a stirred solution of 4'-amino-3'-fluoro-[1,1'-biphenyl]-3-ol (**19b**, 1.0 eq) in THF at rt was added potassium *tert*-butoxide (1 M in THF, 1.0 eq). The mixture was stirred at rt for 30 min before addition of the respective alkyl bromide (**16a**/**17a**/**31a**, 1.0 eq). The reaction mixture was stirred at rt overnight. H_2_O was then added. The aqueous layer was extracted with EtOAc (3x). The organic layers were combined, dried over Na_2_SO_4_, filtered and evaporated. The crude product was purified as described in the respective example (if necessary).

#### General procedure for Suzuki Miyaura coupling using Pd(PPh_3_)_4_ (GP4)

The respective phenylboronic acid derivative (1.1 eq), the respective arylbromide (1.0 eq) and Na_2_CO_3_ (6.0 eq) were evacuated for 10 minutes. A solvent mixture of toluene/EtOH/H_2_O (3:2:1) was degassed by the freeze-pump-thaw method (3x) and added to the reactants under argon to afford a 40-50 mM reaction mixture. Then Pd(PPh_3_)_4_ (0.05 eq) was added, and the reaction was stirred at 60 °C for 6 h. After cooling to rt, EtOAc and H_2_O were added, and the mixture was filtered through Celite. All solvents were removed under reduced pressure and aqueous HCl (10%) was added to the resulting residue. The aqueous layer was extracted with EtOAc (3x). The organic layers were combined, dried over Na_2_SO_4_, filtered and evaporated. The crude product was purified as described in the respective example.

#### cis-2-[(3-Fluoro-3’-methoxy-[1,1’-biphenyl]-4-yl)carbamoyl]cyclopentane-1-carboxylic acid (**8**)

Preparation according to GP2, using arylbromide **8b** (83 mg, 0.25 mmol) and 3-methoxyphenylboronic acid (**4c**, 43 mg, 0.28 mmol). Further purification was performed by RP-CC (H_2_O/ACN) to obtain compound **8** (40 mg, yield: 45%) as a colorless solid. R_f_ (cyclohexane/EtOAc = 7:3 + 1% FA) = 0.48. ^1^H NMR (500 MHz, MeOD-*d*_*4*_): δ = 7.59–7.49 (m, 2H), 7.43–7.29 (m, 2H), 7.26–7.15 (m, 2H), 7.01–6.93 (m, 1H), 3.86 (s, 3H), 3.54–3.36 (m, 2H), 2.26–2.10 (m, 2H), 2.10–1.93 (m, 2H), 1.92–1.81 (m, 1H), 1.62–1.41 (m, 1H) ppm. ^13^C NMR (126 MHz, CD_2_Cl_2_): δ = 179.1, 160.6, 157.9 (d, *J* = 251.7 Hz), 144.7 (d, *J* = 7.8 Hz), 140.9 (d, *J* = 2.0 Hz), 130.4, 130.0, 123.7, 119.9, 119.8–119.4 (m), 115.5 (d, *J* = 20.4 Hz), 114.1, 113.2, 58.6, 55.7, 46.2, 31.3, 25.3, 18.7 ppm. qH NMR (400 MHz, DMSO-*d*_6_, ethyl 4-(dimethylamino)benzoate as reference): purity = 98.9%. MS (+APCI): *m*/*z* 358.4 ([M + H]^+^). HRMS (FIA/ESI): *m*/*z* calculated 380.1269 for C_20_H_20_FNO_4_Na, found 380.1269 ([M + Na]^+^).

#### cis-2-[(4-Bromo-2-fluorophenyl)carbamoyl]-cyclopentane-1-carboxylic acid (**8b**)

Preparation according to GP1, using *cis*-1,2-cyclopentandicarboxylic acid anhydride (**8a**, 0.14 g, 1.0 mmol) and 4-bromo-2-fluoroaniline (**4b**, 0.19 g, 1.0 mmol). Further purification was performed by CC (cyclohexane/EtOAc + 1% FA) to obtain compound **8b** (0.20 g, yield: 61%) as a colorless solid. R_f_ (cyclohexane/EtOAc = 7:3 + 1% FA) = 0.35. ^1^H NMR (500 MHz, D_2_O+NaOD): δ = 7.53 (t, *J* = 8.4 Hz, 1H), 7.45 (dd, *J* = 10.1, 2.2 Hz, 1H), 7.41–7.35 (m, 1H), 3.22–3.13 (m, 1H), 3.02 (q, *J* = 8.4 Hz, 1H), 2.09–1.81 (m, 5H), 1.70–1.58 (m, 1H) ppm. ^13^C NMR (126 MHz, D_2_O+NaOD): δ = 184.2, 178.5, 155.5 (d, *J* = 250.3 Hz), 128.0 (d, *J* = 3.7 Hz), 127.3 (d, *J* = 2.1 Hz), 124.7 (d, *J* = 12.2 Hz), 119.7 (d, *J* = 23.1 Hz), 118.7 (d, *J* = 9.4 Hz), 52.1, 31.7, 31.6, 30.7, 25.5 ppm. MS (+APCI): *m/z* 329.7 ([M + H]^+^).

#### (Z)-4-[(3-Fluoro-3’-methoxy-[1,1’-biphenyl]-4-yl)amino]-4-oxobut-2-enoic acid (**9**)

Preparation according to GP2, using arylbromide **9b** (86 mg, 0.30 mmol) and 3-methoxyphenylboronic acid (**4c**, 52 mg, 0.33 mmol). Further purification was performed by preparative HPLC (H_2_O/ACN + 0.1% FA) to obtain compound **9** (30 mg, yield: 32%) as a colorless solid. R_f_ (cyclohexane/EtOAc = 7:3 + 1% FA) = 0.13. ^1^H NMR (500 MHz, MeOD-*d*_*4*_): δ = 8.12 (t, *J* = 8.5 Hz, 1H), 7.49–7.42 (m, 2H), 7.35 (t, *J* = 8.0 Hz, 1H), 7.22–7.17 (m, 1H), 7.15 (t, *J* = 2.1 Hz, 1H), 6.96–6.90 (m, 1H), 6.64 (d, *J* = 12.4 Hz, 1H), 6.36 (d, *J* = 12.4 Hz, 1H), 3.85 (s, 3H) ppm. ^13^C NMR (126 MHz, MeOD-*d*_*4*_): δ = 168.6, 166.2, 161.7, 155.7 (d, *J* = 246.5 Hz), 141.9 (d, *J* = 2.0 Hz), 140.9 (d, *J* = 7.2 Hz), 134.5, 132.0, 131.1, 125.7 (d, *J* = 11.8 Hz), 125.5 (d, *J* = 1.8 Hz), 123.8 (d, *J* = 3.2 Hz), 120.2, 114.8 (d, *J* = 20.5 Hz), 114.4, 113.5, 55.8 ppm. qH NMR (400 MHz, DMSO-*d*_6_, ethyl 4-(dimethylamino)benzoate as reference): purity = 95.8%. MS (+APCI): *m*/*z* 315.8 ([M + H]^+^). HRMS (GC/EI): *m*/*z* calculated 315.0901 for C_17_H_14_FNO_4_, found 315.0900 ([M]^+^).

#### (Z)-4-[(4-Bromo-2-fluorophenyl)amino]-4-oxobut-2-enoic acid (**9b**)

Preparation according to GP1, using maleic acid anhydride (**9a**, 0.10 g, 1.0 mmol) and 4-bromo-2-fluoroaniline (**4b**, 0.19 g, 1.0 mmol) to give compound **9b** (0.28 g, yield: 97%) as a colorless solid. R_f_ (cyclohexane/EtOAc = 1:1 + 1% FA) = 0.53. ^1^H NMR (500 MHz, DMSO-*d*_*6*_): δ = 12.96 (s, 1H), 10.30 (s, 1H), 7.94 (t, *J* = 8.5 Hz, 1H), 7.62 (dd, *J* = 10.5, 2.2 Hz, 1H), 7.45–7.33 (m, 1H), 6.54 (d, *J* = 12.0 Hz, 1H), 6.36 (d, *J* = 12.0 Hz, 1H) ppm. ^13^C NMR (126 MHz, DMSO-*d*_*6*_): δ = 167.2, 163.4, 153.4 (d, *J* = 250.8 Hz), 131.1, 130.5, 127.5 (d, *J* = 3.4 Hz), 125.3 (d, *J* = 11.6 Hz), 125.2 (d, *J* = 2.3 Hz), 118.9 (d, *J* = 22.7 Hz), 116.1 (d, *J* = 9.0 Hz) ppm. MS (+APCI): *m/z* 387.5 ([M + H]^+^).

#### 2-[(3-Fluoro-[1,1’-biphenyl]-4-yl)carbamoyl]cyclopent-1-ene-1-carboxylic acid (**10**)

Preparation according to GP2, using arylbromide **14** (82 mg, 0.25 mmol) and phenylboronic acid (**10a**, 35 mg, 0.28 mmol). Further purification was performed by preparative HPLC (H_2_O/MeOH + 0.1% FA) to obtain compound **10** (22 mg, yield: 27%) as a yellow solid. R_f_ (cyclohexane/EtOAc = 7:3 + 1% FA) = 0.38. ^1^H NMR (400 MHz, acetone-*d*_*6*_): δ = 10.46 (s, 1H), 8.33–8.20 (m, 1H), 7.75–7.63 (m, 2H), 7.58–7.43 (m, 4H), 7.43–7.34 (m, 1H), 3.07–2.99 (m, 2H), 2.93–2.86 (m, 2H), 1.94 (p, *J* = 7.8 Hz, 2H) ppm. ^13^C NMR (126 MHz, acetone-*d*_*6*_): δ = 166.6, 164.8, 155.1 (d, J = 245.7 Hz), 146.2, 140.6, 140.0 (d, J = 1.9 Hz), 139.6 (d, J = 7.4 Hz), 129.9, 128.7, 127.6, 126.0 (d, J = 11.6 Hz), 124.9, 123.5 (d, J = 2.9 Hz), 114.3 (d, J = 20.4 Hz), 37.3, 37.2, 21.3 ppm. qH NMR (400 MHz, DMSO-*d*_6_, ethyl 4-(dimethylamino)benzoate as reference): purity = 96.3%. MS (+EI): *m*/*z* 325.2 ([M]^+^). HRMS (GC/EI): *m*/*z* calculated 325.1109 for C_19_H_16_FNO_3_, found 325.1119 ([M]^+^).

#### 2-[(3’-Methoxy-[1,1’-biphenyl]-4-yl)carbamoyl]cyclopent-1-ene-1-carboxylic acid (**11**)

Preparation according to GP2, using arylbromide **11b** (93 mg, 0.30 mmol) and 3-methoxyphenylboronic acid (**4c**, 52 mg, 0.33 mmol). Further purification was performed by reversed-phase CC (H_2_O/ACN) to obtain compound **11** (90 mg, yield: 89%) as a yellow solid. R_f_ (cyclohexane/EtOAc = 7:3 + 1% FA) = 0.19. ^1^H NMR (500 MHz, CD_2_Cl_2_) δ = 7.90 (s, 1H), 7.66 (s, 4H), 7.37 (t, *J* = 8.0 Hz, 1H), 7.23–7.16 (m, 1H), 7.16–7.11 (m, 1H), 6.91 (dd, *J* = 8.2, 2.5 Hz, 1H), 3.86 (s, 3H), 3.09–2.89 (m, 4H), 2.00 (p, *J* = 7.5 Hz, 2H) ppm. ^13^C NMR (126 MHz, CD_2_Cl_2_): δ = 165.3, 164.1, 160.6, 148.9, 141.8, 139.5, 139.1, 135.6, 130.3, 128.2, 122.2, 119.7, 113.3, 113.0, 55.7, 37.9, 36.3, 20.6 ppm. qH NMR (400 MHz, DMSO-*d*_6_, ethyl 4-(dimethylamino)benzoate as reference): purity = 95.5%. MS (+APCI): *m*/*z* 338.2 ([M + H]^+^). HRMS (FIA/ESI): *m*/*z* calculated 360.1206 for C_20_H_19_NO_4_Na, found 360.1207 ([M + Na]^+^).

#### 2-([4-Bromophenyl]carbamoyl)cyclopent-1-ene-1-carboxylic acid (**11b**)

Preparation according to GP1, using 1-cyclopentene-1,2-dicarboxylic acid anhydride (**4a**, 73 µl, 0.72 mmol) and 4-bromoaniline (**11a**, 78 µl, 0.72 mmol) to obtain compound **11b** (0.22 g, yield: 98%) as a colorless solid. R_f_ (cyclohexane/CH_2_Cl_2_ = 7:3 + 2% FA) = 0.41. ^1^H NMR (400 MHz, acetone-*d*_6_) δ = 10.06 (s, 1H), 7.69–7.63 (m, 2H), 7.55–7.50 (m, 2H), 3.03–2.94 (m, 2H), 2.87–2.79 (m, 2H), 1.92 (p, *J* = 7.7 Hz, 2H) ppm. ^13^C NMR (101 MHz, acetone-*d*_6_): δ = 165.8, 165.4, 145.8, 141.0, 138.4, 132.6, 123.5, 117.6, 37.0, 36.9, 21.5 ppm. MS (+APCI): *m*/*z* 310.2 ([M + H]^+^).

#### 2-([1,1’-Biphenyl]-4-ylcarbamoyl)cyclopent-1-ene-1-carboxylic acid (**12**)

Preparation according to GP2, using arylbromide **11b** (62 mg, 0.20 mmol) and phenylboronic acid (**12a**, 28 mg, 0.22 mmol). Further purification was performed by preparative HPLC (H_2_O/ACN) to obtain compound **12** (31 mg, yield: 50%) as a colorless solid. R_f_ (cyclohexane/EtOAc = 7:3 + 1% FA) = 0.31. ^1^H NMR (500 MHz, MeOD-*d*_*4*_): δ = 7.72–7.67 (m, 2H), 7.63–7.57 (m, 4H), 7.42 (t, *J* = 7.6 Hz, 2H), 7.31 (t, *J* = 7.4 Hz, 1H), 2.96–2.89 (m, 2H), 2.86–2.79 (m, 2H), 1.99 (p, *J* = 7.6 Hz, 2H) ppm. ^13^C NMR (126 MHz, MeOD-*d*_*4*_): δ = 168.2, 167.2, 148.6, 141.8, 138.8, 137.9–137.7 (m), 129.9, 128.3, 128.2, 127.7, 122.1, 37.6, 36.0, 22.5 ppm. qH NMR (400 MHz, DMSO-*d*_6_, ethyl 4-(dimethylamino)benzoate as reference): purity = 95.5%. MS (+APCI): *m*/*z* 308.5 ([M + H]^+^). HRMS (GC/EI): *m*/*z* calculated 307.1203 for C_19_H_17_NO_3_, found 307.1212 ([M]^+^).

#### 2-[(2-Fluorophenyl)carbamoyl]cyclopent-1-ene-1-carboxylic acid (**13**)

Preparation according to GP1, using 1-cyclopentene-1,2-dicarboxylic acid anhydride (**4a**, 15 µl, 0.15 mmol) and 2-fluoroaniline (**13a**, 15 µl, 0.15 mmol). Further purification was performed by RP-CC (H_2_O/ACN) to obtain compound **13** (29 mg, yield: 78%) as a colorless solid. R_f_ (cyclohexane/EtOAc = 7:3 + 1% FA) = 0.37. ^1^H NMR (500 MHz, acetone-*d*_*6*_): δ = 10.22 (s, 1H), 8.18–8.06 (m, 1H), 7.26–7.15 (m, 3H), 3.06–2.97 (m, 2H), 2.91–2.84 (m, 2H), 1.93 (p, *J* = 7.8 Hz, 2H) ppm. ^13^C NMR (126 MHz, acetone-*d*_*6*_): δ = 166.2, 165.0, 155.1 (d, *J* = 245.9 Hz), 145.8, 140.9, 127.0 (d, *J* = 7.6 Hz), 126.6 (d, *J* = 11.3 Hz), 125.3 (d, *J* = 3.7 Hz), 125.1, 116.2 (d, *J* = 19.4 Hz), 37.2, 37.1, 21.3 ppm. qH NMR (400 MHz, DMSO-*d*_6_, ethyl 4-(dimethylamino)benzoate as reference): purity = 96.9%. MS (+EI): *m*/*z* 249.1 ([M]^+^). HRMS (GC/EI): *m*/*z* calculated 249.0796 for C_13_H_12_FNO_3_, found 249.0797 ([M]^+^).

#### 2-{[3’-(But-2-yn-1-yloxy)-3-fluoro-[1,1’-biphenyl]-4-yl]carbamoyl}cyclopent-1-ene-1-carboxylic acid (**16**)

Preparation according to GP1, using 1-cyclopentene-1,2-dicarboxylic acid anhydride (**4a**, 31 µl, 0.31 mmol) and aniline **16b** (78 mg, 0.31 mmol). Further purification was performed by RP-CC (H_2_O/ACN) to obtain compound **16** (0.07 g, yield: 60%) as a yellow solid. R_f_ (*iso*-hexane/EtOAc = 7:3 + 1% FA) = 0.45. ^1^H NMR (400 MHz, CD_2_Cl_2_): δ = 8.29 (t, *J* = 8.3 Hz, 1H), 8.13 (s, 1H), 7.51–7.34 (m, 3H), 7.24–7.15 (m, 2H), 7.02–6.94 (m, 1H), 4.72 (q, *J* = 2.3 Hz, 2H), 3.10–2.88 (m, 4H), 2.01 (p, *J* = 7.5 Hz, 2H), 1.87 (t, *J* = 2.3 Hz, 3H) ppm. ^13^C NMR (126 MHz, CD_2_Cl_2_): δ = 165.2, 163.9, 158.8, 153.9 (d, *J* = 245.4 Hz), 149.0, 140.7 (d, *J* = 1.9 Hz), 140.2 (d, *J* = 7.6 Hz), 139.1, 130.4, 124.0 (d, *J* = 10.4 Hz), 123.7 (d, *J* = 3.2 Hz), 123.5, 120.2, 114.7, 114.1 (d, *J* = 20.0 Hz), 113.9, 84.2, 74.2, 56.9, 37.9, 36.2, 20.6, 3.7 ppm. qH NMR (400 MHz, CD_2_Cl_2_, ethyl 4-(dimethylamino)benzoate as reference): purity = 95.2%. MS (+APCI): *m*/*z* 393.9 ([M + H]^+^). HRMS (FIA/ESI): *m*/*z* calculated 394.1449 for C_23_H_21_FNO_4_, found 394.1446 ([M + H]^+^).

#### 3’-(But-2-yn-1-yloxy)-3-fluoro-[1,1’-biphenyl]-4-amine (**16b**)

Preparation according to GP3, using 4’-amino-3’-fluoro-[1,1’-biphenyl]-3-ol (**19b**, 70 mg, 0.34 mmol) and 1-bromo-2-butyne (**16a**, 30 µl, 0.34 mmol) to give compound **16b** (0.08 g, yield: 96%) as a brown oil. R_f_ (*iso*-hexane/EtOAc = 8:2 + 1% triethylamine) = 0.31. ^1^H NMR (126 MHz, CD_2_Cl_2_): δ = 7.32 (t, *J* = 8.0 Hz, 1H), 7.29–7.22 (m, 1H), 7.24–7.18 (m, 1H), 7.16–7.12 (m, 1H), 7.11–7.08 (m, 1H), 6.90–6.86 (m, 1H), 6.85–6.82 (m, 1H), 4.69 (q, *J* = 2.3 Hz, 2H), 3.85 (s, 2H), 1.86 (t, *J* = 2.3 Hz, 3H) ppm. ^13^C NMR (126 MHz, CD_2_Cl_2_): δ = 158.7, 152.1 (d, *J* = 238.0 Hz), 141.9 (d, *J* = 2.0 Hz), 134.7 (d, *J* = 13.1 Hz), 131.8 (d, *J* = 6.5 Hz), 130.1, 123.4 (d, *J* = 2.9 Hz), 119.6, 117.3 (d, *J* = 4.2 Hz), 114.1, 114.0, 113.3 (d, *J* = 4.7 Hz), 84.0, 74.4, 56.8, 3.7 ppm. MS (+APCI): *m*/*z* 255.7 ([M + H]^+^).

#### 2-[(3’-Allyloxy-3-fluoro-[1,1’-biphenyl]-4-yl)carbamoyl]cyclopent-1-ene-1-carboxylic acid (**17**)

Preparation according to GP1, using 1-cyclopentene-1,2-dicarboxylic acid anhydride (**4a**, 64 µl, 0.64 mmol) and aniline **17b** (0.16 g, 0.64 mmol). Further purification was performed by RP-CC (H_2_O/ACN) to obtain compound **17** (0.20 g, yield: 61%) as a beige solid. R_f_ (*iso*-hexane/EtOAc = 7:3 + 1% FA) = 0.76. ^1^H NMR (500 MHz, MeOD-*d*_4_): δ = 8.12 (t, *J* = 8.2 Hz, 1H), 7.47–7.39 (m, 2H), 7.34 (t, *J* = 7.9 Hz, 1H), 7.24–7.13 (m, 2H), 6.96–6.90 (m, 1H), 6.15–6.03 (m, 1H), 5.49–5.37 (m, 1H), 5.31–5.23 (m, 1H), 4.65–4.57 (m, 2H), 2.98–2.89 (m, 2H), 2.89–2.81 (m, 2H), 1.98 (p, *J* = 7.7 Hz, 2H) ppm. ^13^C NMR (126 MHz, MeOD-d4): δ = 168.3, 166.8, 160.6, 155.7 (d, *J* = 246.1 Hz), 148.4, 142.1 (d, *J* = 1.9 Hz), 140.3 (d, *J* = 7.2 Hz), 138.2, 135.0, 131.0, 126.1 (d, *J* = 11.8 Hz), 125.4 (d, *J* = 1.2 Hz), 123.7 (d, *J* = 3.1 Hz), 120.3, 117.5, 115.1, 114.7 (d, *J* = 20.7 Hz), 114.3, 69.8, 37.8, 36.4, 22.2 ppm. qH NMR (400 MHz, acetone-*d*_6_, ethyl 4-(dimethylamino)benzoate as reference): purity = 95.2%. MS (+APCI): *m*/*z* 382.4 ([M + H]^+^). HRMS (GC/EI): *m*/*z* calculated 363.1265 for C_22_H_18_FNO_3_, found 363.1265 ([M – H_2_O]^+^).

#### 3’-(Allyloxy)-3-fluoro-[1,1’-biphenyl]-4-amine (**17b**)

Preparation according to GP3, using 4’-amino-3’-fluoro-[1,1’-biphenyl]-3-ol (**19b**, 0.10 g, 0.49 mmol) and allyl bromide (**17a**, 43 µl, 0.49 mmol). Further purification was performed by CC (*iso*-hexane/EtOAc) to obtain compound **17b** (0.09 g, yield: 77%) as a brown oil. R_f_ (*iso*-hexane/EtOAc = 8:2 + 1% triethylamine) = 0.37. ^1^H NMR (400 MHz, acetone-*d*_6_): δ = 7.34–7.21 (m, 3H), 7.18–7.11 (m, 2H), 6.95–6.83 (m, 2H), 6.17–6.03 (m, 1H), 5.44 (dq, *J* = 17.3, 1.7 Hz, 1H), 5.25 (dq, *J* = 10.6, 1.6 Hz, 1H), 4.79 (s, 2H), 4.64 (dt, *J* = 5.2, 1.6 Hz, 2H) ppm. ^13^C NMR (101 MHz, acetone-*d*_6_): δ = 160.1, 152.4 (d, *J* = 236.5 Hz), 142.6 (d, *J* = 2.1 Hz), 136.7 (d, *J* = 13.0 Hz), 134.9, 130.7 (d, *J* = 6.3 Hz), 130.6, 123.7 (d, *J* = 2.9 Hz), 119.4, 117.5 (d, *J* = 4.6 Hz), 117.3, 114.1 (d, *J* = 19.4 Hz), 113.7, 113.2, 69.3 ppm. MS (+APCI): *m*/*z* 244.0 ([M + H]^+^).

#### 2-[(3’-Cyanomethoxy-3-fluoro-[1,1’-biphenyl]-4-yl)carbamoyl]cyclopent-1-ene-1-carboxylic acid (**18**)

Preparation according to GP2, using arylbromide **14** (99 mg, 0.30 mmol) and 3-cyanomethoxyphenylboronic acid (**18a**, 47 µl, 0.33 mmol). Further purification was performed by preparative HPLC (H_2_O/ACN + 0.1% FA) to obtain compound **18** (18 mg, yield: 16%) as a yellow solid. R_f_ (*iso*-hexane/EtOAc = 1:1 + 1% FA) = 0.42. ^1^H NMR (500 MHz, MeOD-*d*_4_): δ = 8.12 (t, *J* = 8.3 Hz, 1H), 7.49–7.42 (m, 2H), 7.37 (t, *J* = 8.0 Hz, 1H), 7.28–7.22 (m, 1H), 7.19 (s, 1H), 6.94 (d, *J* = 8.3 Hz, 1H), 4.73 (s, 2H), 2.98–2.89 (m, 2H), 2.89–2.79 (m, 2H), 1.98 (p, *J* = 7.8 Hz, 2H) ppm. ^13^C NMR (126 MHz, MeOD-*d*_4_): δ = 172.7, 168.3, 166.9, 160.0, 155.8 (d, *J* = 246.7 Hz), 148.4, 142.2 (d, *J* = 2.0 Hz), 140.1 (d, *J* = 7.2 Hz), 138.2, 131.1, 126.2 (d, *J* = 12.0 Hz), 125.4, 123.7 (d, *J* = 3.1 Hz), 121.0, 114.9, 114.7 (d, *J* = 20.6 Hz), 114.3, 66.0, 37.8, 36.4, 22.3 ppm. qH NMR (400 MHz, DMSO-*d*_6_, ethyl 4-(dimethylamino)benzoate as reference): purity = 95.5%. MS (-APCI): *m*/*z* 378.8 ([M – H]^-^). HRMS (FIA/ESI): *m*/*z* calculated 402.0996 for C_21_H_16_FN_2_O_4_Na, found 402.0994 ([M – H + Na]^-^).

#### 2-[(3-Fluoro-3’-hydroxy-[1,1’-biphenyl]-4-yl)carbamoyl]cyclopent-1-ene-1-carboxylic acid (**19**)

Preparation according to GP1, using 1-cyclopentene-1,2-dicarboxylic acid anhydride (**4a**, 37 µl, 0.36 mmol) and aniline **19b** (74 mg, 0.36 mmol). Further purification by crystallization from H_2_O/ACN afforded compound **19** (0.04 g, yield: 30%) as a yellow solid. R_f_ (*iso*-hexane/EtOAc = 7:3 + 1% FA) = 0.20. ^1^H NMR (400 MHz, acetone-*d*_6_): δ = 10.45 (s, 1H), 8.25 (t, *J* = 8.1 Hz, 1H), 7.53–7.40 (m, 2H), 7.33–7.24 (m, 1H), 7.21–7.09 (m, 2H), 6.91–6.79 (m, 1H), 3.06–2.97 (m, 2H), 2.97–2.83 (m, 2H), 1.94 (p, *J* = 7.8 Hz, 2H) ppm. ^13^C NMR (101 MHz, acetone-*d*_6_): δ = 166.4, 164.7, 158.8, 154.9 (d, *J* = 246.0 Hz), 146.4, 141.5 (d, *J* = 2.1 Hz), 140.2, 139.6 (d, *J* = 6.7 Hz), 130.9, 125.9 (d, *J* = 11.1 Hz), 124.7 (d, *J* = 1.2 Hz), 123.4 (d, *J* = 3.1 Hz), 118.8, 115.6, 114.3, 114.1, 37.3, 37.1, 21.4 ppm. qH NMR (400 MHz, DMSO-*d*_6_, ethyl 4-(dimethylamino)benzoate as reference): purity = 99.2%. MS (+APCI): *m*/*z* 341.6 ([M + H]^+^). HRMS (GC/EI): *m*/*z* calculated 323.0952 for C_19_H_14_FNO_3_, found 323.0950 ([M – H_2_O]^+^).

#### 4’-Amino-3’-fluoro-[1,1’-biphenyl]-3-ol (**19b**)

Preparation according to GP2, using 4-bromo-2-fluoroaniline (**4b**, 0.38 g, 2 mmol) and 3-hydroxyphenylboronic acid (**19a**, 0.30 g, 2.2 mmol). Further purification was performed by CC (*iso*-hexane/EtOAc) to obtain compound **19b** (0.37 g, yield: 90%) as a yellow solid. R_f_ (*iso*-hexane/EtOAc = 8:2) = 0.16. ^1^H NMR (400 MHz, acetone-*d*_*6*_): δ = 8.31 (s, 1H), 7.28–7.17 (m, 3H), 7.07–7.00 (m, 2H), 6.94–6.87 (m, 1H), 6.78–6.72 (m, 1H), 4.77 (s, 2H) ppm. ^13^C NMR (126 MHz, acetone-*d*_6_): δ = 158.7, 152.4 (d, *J* = 236.5 Hz), 142.6 (d, *J* = 1.9 Hz), 136.5 (d, *J* = 12.9 Hz), 131.0 (d, *J* = 6.3 Hz), 130.6, 123.6 (d, *J* = 2.8 Hz), 118.2, 117.5 (d, *J* = 4.7 Hz), 114.3, 113.9 (d, *J* = 19.5 Hz), 113.7 ppm. MS (-APCI): *m*/*z* 203.9 ([M + H]^+^).

#### 2-[(3’-Chloro-3-fluoro-[1,1’-biphenyl]-4-yl)carbamoyl]cyclopent-1-ene-1-carboxylic acid (**20**)

Preparation according to GP4, using arylbromide **14** (0.16 g, 0.50 mmol) and 3-chlorophenylboronic acid (**20a**, 86 mg, 0.55 mmol). Further purification was performed by preparative HPLC (H_2_O/ACN) to obtain compound **20** (0.15 g, yield: 82%) as a yellow solid. R_f_ (cyclohexane/EtOAc = 7:3 + 1% FA) = 0.39. ^1^H NMR (500 MHz, DMSO-*d*_6_): δ = 10.63 (s, 1H), 8.11 (t, *J* = 8.3 Hz, 1H), 7.80–7.76 (m, 1H), 7.71–7.65 (m, 2H), 7.59–7.54 (m, 1H), 7.49 (t, *J* = 7.8 Hz, 1H), 7.46–7.40 (m, 1H), 2.83–2.76 (m, 2H), 2.73–2.66 (m, 2H), 1.89 (p, *J* = 7.7 Hz, 2H) ppm. ^13^C NMR (126 MHz, DMSO-*d*_6_): δ = 166.2, 164.8, 153.6 (d, *J* = 245.8 Hz), 147.1, 140.5 (d, *J* = 1.6 Hz), 135.5 (d, *J* = 7.5 Hz), 134.8, 133.8, 130.8, 127.6, 126.3, 125.9 (d, *J* = 11.8 Hz), 125.2, 123.8, 122.7 (d, *J* = 2.9 Hz), 113.8 (d, *J* = 20.6 Hz), 36.4, 34.3, 21.1 ppm. qH NMR (400 MHz, DMSO-*d*_6_, ethyl 4-(dimethylamino)benzoate as reference): purity = 98.2%. MS (+EI): *m*/*z* 359.1 ([M]^+^). HRMS (GC/EI): *m*/*z* calculated 359.0719 for C_19_H_15_ClFNO_3_, found 359.0710 ([M]^+^).

#### 2-[(3-Fluoro-3’-trifluoromethyl-[1,1’-biphenyl]-4-yl)carbamoyl]cyclopent-1-ene-1-carboxylic acid (**21**)

Preparation according to GP2, using arylbromide **14** (99 mg, 0.30 mmol) and 3-(trifluoromethyl)phenylboronic acid (**21a**, 63 mg, 0.33 mmol). Further purification was performed by reversed-phase CC (H_2_O/ACN) to obtain compound **21** (93 mg, yield: 78%) as a colorless solid. R_f_ (*iso*-hexane/EtOAc = 7:3 + 1% FA) = 0.27. ^1^H NMR (500 MHz, CD_2_Cl_2_): δ = 8.36 (t, *J* = 8.3 Hz, 1H), 7.86–7.77 (m, 2H), 7.68–7.58 (m, 2H), 7.52–7.43 (m, 2H), 3.08–2.94 (m, 4H), 2.01 (p, *J* = 7.7 Hz, 2H) ppm. ^13^C NMR (126 MHz, CD_2_Cl_2_): δ = 165.1, 164.1, 154.0 (d, *J* = 245.9 Hz), 148.6, 140.2 (d, *J* = 2.1 Hz), 139.6, 138.7 (d, *J* = 7.6 Hz), 131.6 (q, *J* = 32.2 Hz), 130.7, 130.1, 125.1 (q, *J* = 3.9 Hz), 124.8 (d, *J* = 10.3 Hz), 124.6 (q, *J* = 272.5 Hz), 124.0 (q, *J* = 3.9 Hz), 123.8 (d, *J* = 3.1 Hz), 123.7, 114.2 (d, *J* = 20.4 Hz), 37.9, 36.3, 20.6 ppm. qH NMR (400 MHz, CD_2_Cl_2_, ethyl 4-(dimethylamino)benzoate as reference): purity = 97.9%. MS (+APCI): *m/z* 394.0 ([M + H]^+^). HRMS (GC/EI): *m/z* calculated 375.0877 for C_20_H_13_F_4_NO_2_, found 375.0875 ([M – H_2_O]^+^).

#### 2-[(3-Fluoro-3’-trifluoromethoxy-[1,1’-biphenyl]-4-yl)carbamoyl]cyclopent-1-ene-1-carboxylic acid (**22**)

Preparation according to GP2, using arylbromide **14** (20 mg, 60 µmol) and 3-(trifluoromethoxy)phenylboronic acid (**22a**, 9.6 µL, 66 µmol). Further purification was performed by reversed-phase CC (H_2_O/ACN) to obtain compound **22** (16 mg, yield: 65%) as a yellow solid. R_f_ (*iso*-hexane/EtOAc = 8:2 + 1% FA) = 0.38. ^1^H NMR (500 MHz, acetone-*d*_6_): δ = 10.96 (s, 1H), 8.38 (t, *J* = 8.2 Hz, 1H), 7.77–7.70 (m, 1H), 7.67–7.53 (m, 4H), 7.38–7.32 (m, 1H), 3.05–2.95 (m, 2H), 2.95–2.85 (m, 2H), 1.91 (p, *J* = 7.7 Hz, 2H) ppm. ^13^C NMR (126 MHz, acetone-*d*_6_) δ = 167.0, 164.6, 154.8 (d, *J* = 245.9 Hz), 150.6 (q, *J* = 1.9 Hz), 146.3, 142.4 (d, *J* = 1.9 Hz), 140.2, 137.1 (d, *J* = 7.3 Hz), 131.6, 127.2 (d, *J* = 11.4 Hz), 126.5, 124.5, 123.7 (d, *J* = 3.2 Hz), 121.5 (q, *J* = 255.5 Hz), 120.9, 120.2, 114.5 (d, *J* = 20.8 Hz), 37.4, 37.3, 21.4 ppm. qH NMR (400 MHz, acetone-*d*_*6*_, ethyl 4-(dimethylamino)benzoate as reference): purity = 95.7%. MS (+APCI): *m*/*z* 409.5 ([M + H]^+^). HRMS (GC/EI): *m*/*z* calculated 391.0826 for C_20_H_13_F_4_NO_3_, found 391.0828 ([M – H_2_O]^+^).

#### 2-[(3-Fluoro-3’-difluoromethoxy-[1,1’-biphenyl]-4-yl)carbamoyl]cyclopent-1-ene-1-carboxylic acid (**23**)

Preparation according to GP2, using arylbromide **14** (99 mg, 0.30 mmol) and 3-(difluoromethoxy)phenylboronic acid (**23a**, 47 µL, 0.33 mmol). Further purification was performed by reversed-phase CC (H_2_O/ACN) to obtain compound **23** (106 mg, yield: 89%) as a yellow solid. R_f_ (*iso*-hexane/EtOAc = 7:3 + 1% FA) = 0.28. ^1^H NMR (500 MHz, CD_2_Cl_2_): δ = 8.32 (t, *J* = 8.2 Hz, 1H), 8.28 (s, 1H), 7.54–7.39 (m, 4H), 7.39–7.28 (m, 1H), 7.15 (s, 1H), 6.62 (t, *J* = 74.0 Hz, 1H), 3.09–2.91 (m, 4H), 2.01 (p, *J* = 7.8 Hz, 2H) ppm. ^13^C NMR (126 MHz, CD_2_Cl_2_): δ = 165.1, 164.1, 153.9 (d, *J* = 245.7 Hz), 152.1 (t, *J* = 2.7 Hz), 148.7, 141.4 (d, *J* = 2.0 Hz), 139.4 (t, *J* = 4.4 Hz), 139.0 (d, *J* = 7.5 Hz), 130.9, 124.5 (d, *J* = 10.4 Hz), 124.4, 123.7 (d, *J* = 3.2 Hz), 123.6, 119.3, 118.5, 116.5 (t, *J* = 259.4 Hz), 114.2 (d, *J* = 20.2 Hz), 37.9, 36.2, 20.6 ppm. qH NMR (400 MHz, CD_2_Cl_2_, ethyl 4-(dimethylamino)benzoate as reference): purity = 96.0%. MS (+APCI): *m*/*z* 392.1 ([M + H]^+^). HRMS (GC/EI): *m*/*z* calculated 373.0920 for C_20_H_14_F_3_NO_3_, found 373.0920 ([M – H_2_O]^+^).

#### 2-[(3-Fluoro-3’-(difluoromethoxy-d)-[1,1’-biphenyl]-4-yl)carbamoyl]cyclopent-1-ene-1-carboxylic acid (**24**)

To a solution of compound **24d** (70 mg, 0.28 mmol) in CH_2_Cl_2_ (2.5 mL) was added 1-cyclopentene-1,2-dicarboxylic acid anhydride (**4a**, 0.44 g, 0.28 mmol) and the mixture was heated at 40 °C for 4 h. The mixture was cooled to rt, filtered and the filter cake washed with ACN (2x2 mL). The solid was dried in vacuum to afford compound **24** (70 mg, yield: 65%) as a light yellow solid. R_f_ (*iso*-hexane/EtOAc = 7:3 + 1% FA) = 0.29. ^1^H NMR (500 MHz, CD_2_Cl_2_): δ = 8.33 (t, *J* = 8.2 Hz, 1H), 8.25 (s, 1H), 7.50–7.41 (m, 4H), 7.34 (s, 1H), 7.19–7.12 (m, 1H), 3.06–2.96 (m, 4H), 2.01 (p, *J* = 7.9 Hz, 2H) ppm. ^13^C NMR (126 MHz, CD_2_Cl_2_): δ = 165.1, 164.1, 153.9 (d, *J* = 245.8 Hz), 152.1 (t, *J* = 2.8 Hz), 148.8, 141.4 (d, *J* = 2.0 Hz), 139.3, 139.0 (d, *J* = 7.3 Hz), 130.9, 124.5 (d, *J* = 10.1 Hz), 124.3, 123.7 (d, *J* = 3.1 Hz), 123.6, 119.3, 118.5, 116.2 (t, *J* = 34.2 Hz), 114.2 (d, *J* = 20.3 Hz), 37.9, 36.2, 20.6 ppm. qH NMR (400 MHz, CD_2_Cl_2_, ethyl 4-(dimethylamino)benzoate as reference): purity = 97.7%. LCMS (ESI): m/z 393.3 (M + H)^+^. HRMS (GC/EI): *m*/*z* calculated 392.1094 for C_20_H_15_DF_3_NO_4_, found 392.1088 ([M]^+^).

#### 1-Bromo-3-(difluoromethoxy-d)benzene (**24b**)

To a solution of 3-bromophenol (**24a**,0.56 g, 3.2 mmol) in dry THF (10 mL) was added NaH (1.3 g, 60% w/w, 33 mmol) at 0 °C and the mixture was stirred at 0 °C for 30 min, then D_2_O (6.5 mL) was added dropwise at 0 °C for 10 min. After addition of diethyl (bromodifluoromethyl)phosphonate (1.7 g, 6.4 mmol), the mixture was stirred at rt for 30 min. The mixture was extracted with EtOAc (3x20 mL). The combined organic layer was washed with brine (100 mL), dried over Na_2_SO_4_, concentrated and purified by CC (PE/EtOAc = 40:1) to give compound **24b** (0.25 g, yield: 35%) as a colorless oil. ^1^H NMR (500 MHz, DMSO-*d*_6_): δ = 7.48–7.38 (m, 3H), 7.22 (dd, *J* = 2.0, 8.0 Hz, 1H) ppm.

#### 3’-(Difluoromethoxy-d)-3-fluoro-[1,1’-biphenyl]-4-amine (**24d**)

To a solution of compound **24b** (0.25 g, 1.1 mmol) in 1,4-dioxane (6 mL) and H_2_O (0.6 mL) was added 2-fluoro-4-(4,4,5,5-tetramethyl-1,3,2-dioxaborolan-2-yl)aniline (**24c**, 0.27 g, 1.1 mmol), Na_2_CO_3_ (0.36 g, 3.4 mmol) and Pd(dppf)Cl_2_ (41 mg, 56 µmol). The mixture was heated at 90 °C for 2 h and cooled to rt. The organic layer was separated, concentrated and purified by CC (PE/EtOAc = 10:1) to give compound **24d** (0.25 g, yield: 88%) as a colorless oil. ^1^H NMR (500 MHz, DMSO-*d*_6_): δ = 7.47–7.39 (m, 3H), 7.37 (s, 1H), 7.28 (dd, *J* = 1.8, 8.3 Hz, 1H), 7.05 (d, *J* = 8.0 Hz, 1H), 6.84 (t, *J* = 9.0, 1H), 5.38 (s, 2H) ppm. LCMS (ESI): m/z 255.3 (M + H)^+^.

#### 2-{[3-Fluoro-3’-(2,2,2-trifluoroethoxy)-[1,1’-biphenyl]-4-yl]carbamoyl}cyclopent-1-ene-1-carboxylic acid (**25**)

Preparation according to GP2, using arylbromide **14** (49 mg, 0.15 mmol) and 3-(2,2,2-trifluoroethoxy)phenylboronic acid (**25a**, 27 µL, 0.17 mmol). Further purification was performed by reversed-phase CC (H_2_O/ACN) to obtain compound **25** (58 mg, yield: 91%) as a yellow solid. R_f_ (*iso*-hexane/EtOAc = 8:2 + 1% FA) = 0.24. ^1^H NMR (500 MHz, acetone-*d*_6_): δ = 10.48 (s, 1H), 8.28 (t, *J* = 8.2 Hz, 1H), 7.60–7.53 (m, 2H), 7.47–7.42 (m, 1H), 7.41–7.37 (m, 2H), 7.11–7.06 (m, 1H), 4.78 (q, *J* = 8.6 Hz, 2H), 3.05–2.98 (m, 2H), 2.93–2.86 (m, 2H), 1.94 (p, *J* = 7.7 Hz, 2H) ppm. ^13^C NMR (126 MHz, acetone-*d*_6_): δ = 166.5, 164.7, 159.0, 154.9 (d, *J* = 245.4 Hz), 146.5, 141.8 (d, *J* = 2.0 Hz), 140.0, 138.8 (d, *J* = 7.3 Hz), 131.2, 126.3 (d, *J* = 11.3 Hz), 125.0 (q, *J* = 277.0 Hz), 124.7, 123.6 (d, *J* = 3.1 Hz), 121.5, 115.4, 114.5 (d, *J* = 20.5 Hz), 113.8, 66.2 (q, *J* = 35.0 Hz), 37.3, 37.1, 21.4 ppm. qH NMR (400 MHz, DMSO-*d*_*6*_, ethyl 4-(dimethylamino)benzoate as reference): purity = 95.3%. MS (+APCI): *m*/*z* 423.5 ([M + H]^+^). HRMS (GC/EI): *m*/*z* calculated 405.0983 for C_21_H_15_F_4_NO_3_, found 405.0984 ([M – H_2_O]^+^).

#### 2-({3-Fluoro-3’-[(2,2,2-trifluoroethoxy)methyl]-[1,1’-iphenyl]-4-yl}carbamoyl)cyclopent-1-ene-1-carboxylic acid (**26**)

Preparation according to GP2, using arylbromide **14** (99 mg, 0.30 mmol) and 3-[(2,2,2-trifluoroethoxy)methyl]phenylboronic acid (**26a**, 60 µL, 0.33 mmol). Further purification was performed by reversed-phase CC (H_2_O/ACN) to obtain compound **26** (91 mg, yield: 68%) as a colorless solid. R_f_ (*iso*-hexane/EtOAc = 8:2 + 1% FA) = 0.18. ^1^H NMR (500 MHz, CD_2_Cl_2_): δ = 8.30 (t, *J* = 8.3 Hz, 1H), 8.25 (s, 1H), 7.60–7.53 (m, 2H), 7.51–7.43 (m, 3H), 7.40–7.35 (m, 1H), 4.74 (s, 2H), 3.91 (q, *J* = 8.8 Hz, 2H), 3.07–3.01 (m, 2H), 3.01–2.95 (m, 2H), 2.01 (p, *J* = 7.8 Hz, 2H) ppm. ^13^C NMR (126 MHz, CD_2_Cl_2_): δ = 165.1, 164.2, 154.0 (d, *J* = 245.4 Hz), 148.6, 140.1 (d, *J* = 7.6 Hz), 139.7 (d, *J* = 1.9 Hz), 139.4, 138.1, 129.7, 127.9, 127.1, 126.6, 125.7 (q, *J* = 279.3 Hz), 124.1 (d, *J* = 10.4 Hz), 123.63, 123.59 (d, *J* = 2.8 Hz), 114.1 (d, *J* = 20.2 Hz), 74.3, 67.8 (q, *J* = 33.9 Hz), 37.9, 36.2, 20.6 ppm. qH NMR (400 MHz, CD_2_Cl_2_, ethyl 4-(dimethylamino)benzoate as reference): purity = 97.6%. MS (+APCI): *m*/*z* 438.4 ([M + H]^+^). HRMS (FIA/ESI): *m*/*z* calculated 438.1323 for C_22_H_20_F_4_NO_4_, found 438.1320 ([M + H]^+^).

#### 2-[(3’-Benzyloxy-3-fluoro-[1,1’-biphenyl]-4-yl)carbamoyl]cyclopent-1-ene-1-carboxylic acid (**27**)

Preparation according to GP2, using arylbromide **14** (49 mg, 0.15 mmol) and 3-benzyloxyphenylboronic acid (**27a**, 31 µL, 0.17 mmol). Further purification was performed by reversed-phase CC (H_2_O/ACN) to obtain compound **27** (14 mg, yield: 22%) as a yellow solid. R_f_ (*iso*-hexane/EtOAc = 8:2 + 1% FA) = 0.27. ^1^H NMR (500 MHz, acetone-*d*_6_): δ = 10.67 (s, 1H), 8.29 (t, *J* = 8.3 Hz, 1H), 7.59–7.49 (m, 4H), 7.48–7.31 (m, 5H), 7.31–7.27 (m, 1H), 7.05 (dd, *J* = 8.2, 2.5 Hz, 1H), 5.24 (s, 2H), 3.08–2.97 (m, 2H), 2.97–2.84 (m, 2H), 1.94 (p, *J* = 7.7 Hz, 2H) ppm. ^13^C NMR (126 MHz, acetone-*d*_6_): δ = 164.6, 160.4, 154.9 (d, *J* = 245.5 Hz), 146.0, 141.5 (d, *J* = 1.9 Hz), 140.5, 139.2 (d, *J* = 7.4 Hz), 138.4, 130.9, 129.3, 128.7, 128.5, 126.3 (d, *J* = 11.6 Hz), 124.6, 123.5 (d, *J* = 3.1 Hz), 120.1, 115.2, 114.4 (d, *J* = 20.5 Hz), 114.0, 70.5, 37.29, 37.25, 21.4 ppm. qH NMR (400 MHz, DMSO-*d*_6_, ethyl 4-(dimethylamino)benzoate as reference): purity = 95.7%. MS (+APCI): *m*/*z* 431.5 ([M + H]^+^). HRMS (GC/EI): *m*/*z* calculated 431.1527 for C_26_H_22_FNO_4_, found 431.1538 ([M]^+^).

#### 2-[(3-Fluoro-3’-phenoxy-[1,1’-biphenyl]-4-yl)carbamoyl]cyclopent-1-ene-1-carboxylic acid (**28**)

Preparation according to GP2, using arylbromide **14** (66 mg, 0.20 mmol) and 3-phenoxyphenylboronic acid (**28a**, 38 µL, 0.22 mmol). Further purification was performed by reversed-phase CC (H_2_O/ACN) to obtain compound **28** (42 mg, yield: 50%) as a yellow solid. R_f_ (cyclohexane/EtOAc = 7:3 + 1% FA) = 0.35. ^1^H NMR (500 MHz, CD_2_Cl_2_): δ = 8.28 (t, *J* = 8.3 Hz, 1H), 8.23 (s, 1H), 7.48–7.30 (m, 6H), 7.24 (t, *J* = 2.1 Hz, 1H), 7.18–7.10 (m, 1H), 7.09–7.03 (m, 2H), 7.03–6.98 (m, 1H), 3.05–2.94 (m, 4H), 2.00 (p, *J* = 7.8 Hz, 2H) ppm. ^13^C NMR (126 MHz, CD_2_Cl_2_): δ = 165.1, 164.2, 158.4, 157.4, 153.9 (d, *J* = 245.5 Hz), 148.5, 141.2 (d, *J* = 2.0 Hz), 139.7 (d, *J* = 7.5 Hz), 139.5, 130.8, 130.3, 124.2 (d, *J* = 10.6 Hz), 124.0, 123.6 (d, *J* = 3.2 Hz), 123.5, 122.1, 119.4, 118.6, 117.5, 114.1 (d, *J* = 20.1 Hz), 37.9, 36.2, 20.6 ppm. qH NMR (400 MHz, DMSO-*d*_6_, ethyl 4-(dimethylamino)benzoate as reference): purity = 99.9%. MS (+APCI): *m*/*z* 418.4 ([M + H]^+^). HRMS (FIA/ESI): *m*/*z* calculated 440.1268 for C_25_H_20_FNO_4_Na, found 440.1265 ([M + Na]^+^).

#### 2-{[3-Fluoro-(1,1’:3’,1’-terphenyl)-4-yl]carbamoyl}cyclopent-1-ene-1-carboxylic acid (**29**)

Preparation according to GP2, using arylbromide **14** (66 mg, 0.20 mmol) and 3-biphenylboronic acid (**29a**, 44 mg, 0.22 mmol). Further purification was performed by reversed-phase CC (H_2_O/ACN) to obtain compound **29** (75 mg, yield: 93%) as a yellow solid. R_f_ (cyclohexane/EtOAc = 7:3 + 1% FA) = 0.49. ^1^H NMR (500 MHz, CD_2_Cl_2_) δ = 8.33 (t, *J* = 8.3 Hz, 1H), 8.14 (s, 1H), 7.85–7.78 (m, 1H), 7.71–7.61 (m, 3H), 7.62–7.43 (m, 6H), 7.42–7.35 (m, 1H), 3.10–2.92 (m, 4H), 2.02 (p, *J* = 7.7 Hz, 2H) ppm. ^13^C NMR (126 MHz, CD_2_Cl_2_): δ = 165.2, 163.9, 154.0 (d, *J* = 245.4 Hz), 149.1, 142.4, 141.1, 140.5 (d, *J* = 7.6 Hz), 139.9 (d, *J* = 1.9 Hz), 139.0, 129.9, 129.3, 128.0, 127.6, 127.3, 126.2, 126.1, 124.0 (d, *J* = 10.4 Hz), 123.7 (d, *J* = 3.2 Hz), 123.6, 114.2 (d, *J* = 20.0 Hz), 37.9, 36.2, 20.6 ppm. qH NMR (400 MHz, DMSO-*d*_6_, ethyl 4-(dimethylamino)benzoate as reference): purity = 99.2%. MS (+APCI): *m*/*z* 402.2 ([M + H]^+^). HRMS (FIA/ESI): *m*/*z* calculated 424.1319 for C_25_H_20_FNO_3_Na, found 424.1317 ([M + Na]^+^).

#### 2-[(3-Fluoro-3’-phenylcarbamoyl-[1,1’-biphenyl]-4-yl)carbamoyl]cyclopent-1-ene-1-carboxylic acid (**30**)

Preparation according to GP2, using arylbromide **14** (66 mg, 0.20 mmol) and 3-phenylcarbamoylbenzeneboronic acid (**30a**, 41 µl, 0.22 mmol). Further purification was performed by preparative HPLC (H_2_O/ACN) to obtain compound **30** (59 mg, yield: 66%) as a yellow solid. R_f_ (cyclohexane/EtOAc = 7:3 + 1% FA) = 0.19. ^1^H NMR (500 MHz, DMSO-*d*_6_) δ = 10.81 (s, 1H), 10.33 (s, 1H), 8.24 (s, 1H), 8.15 (t, *J* = 8.3 Hz, 1H), 7.98–7.87 (m, 2H), 7.84–7.71 (m, 3H), 7.71–7.53 (m, 2H), 7.37 (t, *J* = 7.8 Hz, 2H), 7.12 (t, *J* = 7.4 Hz, 1H), 2.86–2.75 (m, 2H), 2.75–2.65 (m, 2H), 1.89 (p, *J* = 7.6 Hz, 2H) ppm. ^13^C NMR (126 MHz, DMSO-*d*_6_): δ = 166.3, 165.4, 164.8, 153.8 (d, *J* = 245.8 Hz), 146.7, 139.1, 138.5 (d, *J* = 1.3 Hz), 136.5 (d, *J* = 7.1 Hz), 135.7, 135.4, 129.6, 129.3, 128.7, 127.2, 125.7 (d, *J* = 11.7 Hz), 125.5, 124.0, 123.9, 122.7 (d, *J* = 2.9 Hz), 120.6, 113.8 (d, *J* = 20.6 Hz), 36.5, 34.5, 21.1 ppm. qH NMR (400 MHz, DMSO-*d*_6_, ethyl 4-(dimethylamino)benzoate as reference): purity = 95.1%. MS (+APCI): *m*/*z* 444.6 ([M + H]^+^). HRMS (FIA/ESI): *m*/*z* calculated 467.1377 for C_26_H_21_FN_2_O_4_Na, found 467.1375 ([M + Na]^+^).

#### 2-{[3-Fluoro-3’-(isoxazol-5-ylmethoxy)-[1,1’-biphenyl]-4-yl]carbamoyl}cyclopent-1-ene-1-carboxylic acid (**31**)

Preparation according to GP1, using 1-cyclopentene-1,2-dicarboxylic acid anhydride (**4a**, 3.5 µl, 35 µmol) and aniline **31b** (10 mg, 35 µmol). Further purification was performed by preparative HPLC (H_2_O/ACN) to obtain compound **31** (8 mg, yield: 54%) as a colorless solid. R_f_ (cyclohexane/EtOAc = 7:3 + 1% FA) = 0.22. ^1^H NMR (500 MHz, MeOD-*d*_4_): δ = 8.39 (d, *J* = 1.8 Hz, 1H), 8.23 (t, *J* = 8.5 Hz, 1H), 7.47–7.32 (m, 3H), 7.29–7.19 (m, 2H), 7.04–6.96 (m, 1H), 6.57–6.46 (m, 1H), 5.32 (s, 2H), 2.94–2.78 (m, 4H), 1.82 (p, *J* = 7.7 Hz, 2H) ppm. ^13^C NMR (126 MHz, MeOD-*d*_4_): δ = 174.8, 169.1, 165.9, 159.9, 155.4 (d, *J* = 246.3 Hz), 151.6, 147.4, 142.4 (d, *J* = 1.3 Hz), 139.3, 138.9 (d, *J* = 7.3 Hz), 131.2, 127.4 (d, *J* = 11.7 Hz), 124.6 (d, *J* = 2.0 Hz), 123.6 (d, *J* = 3.2 Hz), 121.2, 115.1, 114.5 (d, *J* = 20.7 Hz), 114.3, 104.1, 62.0, 39.2, 36.9, 21.6 ppm. qH NMR (400 MHz, DMSO-*d*_6_, ethyl 4-(dimethylamino)benzoate as reference): purity = 95.3%. MS (+APCI): *m*/*z* 422.7 ([M]^+^). HRMS (FIA/ESI): *m*/*z* calculated 445.1170 for C_23_H_19_FN_2_O_5_Na, found 445.1167 ([M + Na]^+^).

#### 3-Fluoro-3’-(isoxazol-5-ylmethoxy)-[1,1’-biphenyl]-4-amine (**31b**)

Preparation according to GP3, using 4’-amino-3’-fluoro-[1,1’-biphenyl]-3-ol (**19b**, 0.04 g, 0.02 mmol) and 5-bromomethyl-isoxazole (**31a**, 0.03 g, 0.02 mmol) to give compound **31b** (0.01 g, yield: 18%) as a yellow solid. R_f_ (cyclohexane/EtOAc = 7:2 + 1% TEA) = 0.31. ^1^H NMR (500 MHz, acetone-*d*_*6*_): δ = 8.42 (d, *J* = 1.7 Hz, 1H), 7.37–7.30 (m, 2H), 7.29–7.19 (m, 3H), 6.99–6.88 (m, 2H), 6.60–6.55 (m, 1H), 5.38 (s, 2H), 4.82 (s, 2H) ppm. ^13^C NMR (126 MHz, acetone-*d*_*6*_): δ = 168.4, 159.5, 152.4 (d, *J* = 236.6 Hz), 151.3, 142.7 (d, *J* = 1.9 Hz), 136.8 (d, *J* = 13.1 Hz), 130.8, 130.4 (d, *J* = 6.3 Hz), 123.8 (d, *J* = 2.8 Hz), 120.2, 117.5 (d, *J* = 4.6 Hz), 114.1 (d, *J* = 19.4 Hz), 113.8, 113.2, 103.9, 61.5 ppm. MS (+APCI): *m*/*z* 284.7 ([M]^+^).

#### 2-[(3-Fluoro-2’-methoxy-[1,1’-biphenyl]-4-yl)carbamoyl]cyclopent-1-ene-1-carboxylic acid (**32**)

Preparation according to GP2, using arylbromide **14** (99 mg, 0.30 mmol) and 2-methoxyphenylboronic acid (**32a**, 52 mg, 0.33 mmol). Further purification was performed by reversed-phase CC (H_2_O/ACN) to obtain compound **32** (88 mg, yield: 81%) as a colorless solid. R_f_ (*iso*-hexane/EtOAc = 7:3 + 1% FA) = 0.28. ^1^H NMR (400 MHz, acetone-*d*_6_): δ = 10.32 (s, 1H), 8.23–8.10 (m, 1H), 7.43–7.38 (m, 1H), 7.38–7.31 (m, 3H), 7.14–7.09 (m, 1H), 7.07–7.01 (m, 1H), 3.84 (s, 3H), 3.09–2.97 (m, 2H), 2.94–2.82 (m, 2H), 1.94 (p, *J* = 7.7 Hz, 2H) ppm. ^13^C NMR (126 MHz, acetone-*d*_6_): δ = 166.3, 164.8, 157.5, 154.3 (d, *J* = 244.7 Hz), 146.1, 140.5, 137.5 (d, *J* = 7.8 Hz), 131.2, 130.1, 129.4 (d, *J* = 1.9 Hz), 126.1 (d, *J* = 3.2 Hz), 125.2 (d, *J* = 11.5 Hz), 124.1, 121.7, 117.1 (d, *J* = 20.4 Hz), 112.5, 55.9, 37.3, 37.1, 21.4 ppm. qH NMR (400 MHz, acetone-*d*_6_, ethyl 4-(dimethylamino)benzoate as reference): purity = 96.2%. MS (+APCI): *m*/*z* 355.8 ([M + H]^+^). HRMS (GC/EI): *m*/*z* calculated 337.1109 for C_20_H_16_FNO_3_, found 337.1109 ([M – H_2_O]^+^).

#### 2-[(3-Fluoro-4’-methoxy-[1,1’-biphenyl]-4-yl)carbamoyl]cyclopent-1-ene-1-carboxylic acid (**33**)

Preparation according to GP2, using arylbromide **14** (99 mg, 0.30 mmol) and 4-methoxyphenylboronic acid (**33a**, 52 mg, 0.33 mmol). Further purification by crystallization from ACN afforded compound **33** (81 mg, yield: 75%) as a yellow solid. R_f_ (*iso*-hexane/EtOAc = 7:3 + 1% FA) = 0.24. ^1^H NMR (400 MHz, DMSO-*d*_6_) δ = 10.56 (s, 1H), 8.02 (t, *J* = 8.2 Hz, 1H), 7.76–7.33 (m, 4H), 7.13–6.92 (m, 2H), 3.80 (s, 3H), 2.87–2.61 (m, 4H), 1.96–1.81 (m, 2H) ppm. ^13^C NMR (126 MHz, DMSO-*d*_6_): δ = 166.2, 164.7, 159.1, 153.9 (d, *J* = 245.1 Hz), 147.0, 137.2 (d, *J* = 7.3 Hz), 135.0, 130.8, 127.7, 124.4 (d, *J* = 11.8 Hz), 124.1, 121.8 (d, *J* = 3.0 Hz), 114.4, 112.9 (d, *J* = 20.3 Hz), 55.2, 36.4, 34.4, 21.1 ppm. qH NMR (400 MHz, acetone-*d*_6_, ethyl 4-(dimethylamino)benzoate as reference): purity = 96.7%. MS (-APCI): *m*/*z* 355.8 ([M + H]^+^). HRMS (GC/EI): *m*/*z* calculated 337.1109 for C_20_H_16_FNO_3_, found 337.1107 ([M – H_2_O]^+^).

#### 2-[(3-Fluoro-2’-trifluoromethoxy-[1,1’-biphenyl]-4-yl)carbamoyl]cyclopent-1-ene-1-carboxylic acid (**34**)

Preparation according to GP2, using arylbromide **14** (62 mg, 0.19 mmol) and 3-trifluoromethoxyphenylboronic acid (**34a**, 31 µl, 0.21 mmol). Further purification was performed by reversed-phase CC (H_2_O/ACN) to obtain compound **34** (38 mg, yield: 49%) as a yellow solid. R_f_ (cyclohexane/EtOAc = 7:3 + 1% FA) = 0.33. ^1^H NMR (400 MHz, CD_2_Cl_2_): δ = 8.34 (t, *J* = 8.3 Hz, 1H), 8.25 (s, 1H), 7.59–7.38 (m, 5H), 7.30–7.21 (m, 1H), 3.10–2.91 (m, 4H), 2.01 (p, *J* = 7.7 Hz, 2H) ppm. ^13^C NMR (126 MHz, CD_2_Cl_2_): δ = 165.2, 164.0, 153.9 (d, *J* = 245.8 Hz), 150.1 (q, *J* = 1.7 Hz), 148.8, 141.5 (d, *J* = 1.9 Hz), 139.3, 138.67 (d, *J* = 7.6 Hz), 130.9, 125.8, 124.7 (d, *J* = 10.4 Hz), 123.7 (d, *J* = 3.1 Hz), 123.6, 120.9 (q, *J* = 257.0 Hz), 120.8, 119.9, 114.2 (d, *J* = 20.3 Hz), 37.9, 36.2, 20.6 ppm. qH NMR (400 MHz, DMSO-*d*_6_, ethyl 4-(dimethylamino)benzoate as reference): purity = 96.1%. MS (+APCI): *m*/*z* 409.3 ([M + H]^+^). HRMS (DEP/EI): *m*/*z* calculated 409.0932 for C_20_H_15_F_4_NO_4_, found 409.0930 ([M]^+^).

#### 2-[(3-Fluoro-4’-trifluoromethoxy-[1,1’-biphenyl]-4-yl)carbamoyl]cyclopent-1-ene-1-carboxylic acid (**35**)

Preparation according to GP2, using arylbromide **14** (49 mg, 0.15 mmol) and 4-trifluoromethoxyphenylboronic acid (**35a**, 34 mg, 0.17 mmol). Further purification was performed by CC (cyclohexane/EtOAc) to obtain compound **35** (26 mg, yield: 42%) as a yellow solid. R_f_ (cyclohexane/EtOAc = 7:3 + 1% FA) = 0.39. ^1^H NMR (500 MHz, CD_2_Cl_2_): δ = 8.32 (t, *J* = 8.3 Hz, 1H), 8.20 (s, 1H), 7.66–7.60 (m, 2H), 7.48–7.39 (m, 2H), 7.36–7.30 (m, 2H), 3.07–2.94 (m, 4H), 2.01 (p, *J* = 7.8 Hz, 2H) ppm. ^13^C NMR (126 MHz, CD_2_Cl_2_): δ = 165.2, 164.0, 153.9 (d, *J* = 245.5 Hz), 149.5 (q, *J* = 1.7 Hz), 148.8, 139.3, 139.0 (d, *J* = 7.6 Hz), 138.2 (d, *J* = 2.2 Hz), 128.8, 124.3 (d, *J* = 10.3 Hz), 123.7 (d, *J* = 3.3 Hz), 123.6, 121.8, 120.9 (q, *J* = 256.9 Hz), 114.1 (d, *J* = 20.3 Hz), 37.9, 36.2, 20.6 ppm. qH NMR (400 MHz, DMSO-*d*_6_, ethyl 4-(dimethylamino)benzoate as reference): purity = 95.2%. MS (+APCI): *m*/*z* 409.6 ([M]^+^). HRMS (DEP/EI): *m*/*z* calculated 409.0932 for C_20_H_15_F_4_NO_4_, found 409.0946 ([M]^+^).

#### 2-[(3-Fluoro-2’,3’-dimethoxy-[1,1’-biphenyl]-4-yl)carbamoyl]cyclopent-1-ene-1-carboxylic acid (**36**)

Preparation according to GP2, using arylbromide **14** (99 mg, 0.30 mmol) and 2,3-dimethoxyphenylboronic acid (**36a**, 61 mg, 0.33 mmol). Further purification was performed by reversed-phase CC (H_2_O/ACN) to obtain compound **36** (61 mg, yield: 52%) as a yellow solid. R_f_ (*iso*-hexane/EtOAc = 8:2 + 1% FA) = 0.49. ^1^H NMR (500 MHz, acetone-*d*_6_): δ = 10.37 (s, 1H), 8.29–8.16 (m, 1H), 7.44–7.32 (m, 2H), 7.14 (t, *J* = 7.9 Hz, 1H), 7.10–7.05 (m, 1H), 7.01–6.94 (m, 1H), 3.90 (s, 3H), 3.63 (s, 3H), 3.09–2.97 (m, 2H), 2.94–2.83 (m, 2H), 1.94 (p, *J* = 7.8 Hz, 2H) ppm. ^13^C NMR (126 MHz, acetone-*d*_6_): δ = 166.3, 164.8, 154.4, 154.3 (d, *J* = 244.9 Hz), 147.5, 146.3, 140.3, 137.1 (d, *J* = 7.5 Hz), 134.7 (d, *J* = 1.8 Hz), 126.0 (d, *J* = 3.2 Hz), 125.5 (d, *J* = 11.3 Hz), 125.1, 124.1, 122.8, 116.8 (d, *J* = 20.5 Hz), 113.4, 60.6, 56.3, 37.3, 37.1, 21.4 ppm. qH NMR (400 MHz, DMSO-*d*_6_, ethyl 4-(dimethylamino)benzoate as reference): purity = 96.8%. MS (+APCI): *m*/*z* 386.1 ([M + H]^+^). HRMS (GC/EI): *m*/*z* calculated 367.1214 for C_21_H_18_FNO_4_, found 367.1213 ([M – H_2_O]^+^).

#### 2-[(3-Fluoro-2’,5’-dimethoxy-[1,1’-biphenyl]-4-yl)carbamoyl]cyclopent-1-ene-1-carboxylic acid (**37**)

Preparation according to GP2, using arylbromide **14** (99 mg, 0.30 mmol) and 2,5-dimethoxyphenylboronic acid (**37a**, 61 mg, 0.33 mmol). Further purification was performed by reversed-phase CC (H_2_O/ACN) to obtain compound **37** (95 mg, yield: 81%) as a yellow solid. R_f_ (*iso*-hexane/EtOAc = 8:2 + 1% FA) = 0.51. ^1^H NMR (500 MHz, acetone-*d*_6_): δ = 10.33 (s, 1H), 8.24–8.13 (m, 1H), 7.46–7.33 (m, 2H), 7.05 (d, *J* = 8.9 Hz, 1H), 6.98–6.88 (m, 2H), 3.80 (s, 3H), 3.77 (s, 3H), 3.08–2.97 (m, 2H), 2.95–2.84 (m, 2H), 1.94 (p, *J* = 7.7 Hz, 2H) ppm. ^13^C NMR (126 MHz, acetone-*d*_6_): δ = 166.3, 164.8, 154.9, 154.3 (d, *J* = 245.0 Hz), 151.6, 146.1, 140.6, 137.4 (d, *J* = 7.9 Hz), 130.3 (d, *J* = 1.6 Hz), 126.1 (d, *J* = 3.2 Hz), 125.3, 124.2 (d, *J* = 13.1 Hz), 117.1 (d, *J* = 20.5 Hz), 116.9, 114.6, 113.9, 56.6, 56.0, 37.2, 37.1, 21.4 ppm. qH NMR (400 MHz, DMSO-*d*_6_, ethyl 4-(dimethylamino)benzoate as reference): purity = 95.1%. MS (+APCI): *m*/*z* 386.1 ([M + H]^+^). HRMS (GC/EI): *m*/*z* calculated 367.1214 for C_21_H_18_FNO_4_, found 367.1215 ([M – H_2_O]^+^).

#### 2-[(3-Fluoro-2’,4’-dimethoxy-[1,1’-biphenyl]-4-yl)carbamoyl]cyclopent-1-ene-1-carboxylic acid (**38**)

Preparation according to GP2, using arylbromide **14** (82 mg, 0.25 mmol) and 2,4-dimethoxyphenylboronic acid (**38a**, 50 mg, 0.28 mmol). Further purification was performed by reversed-phase CC (H_2_O/ACN) to obtain compound **38** (38 mg, yield: 39%) as a yellow solid. R_f_ (cyclohexane/EtOAc = 7:3 + 1% FA) = 0.29. ^1^H NMR (500 MHz, CD_2_Cl_2_): δ = 8.26 (s, 1H), 8.20 (t, *J* = 8.3 Hz, 1H), 7.43–7.37 (m, 1H), 7.37–7.31 (m, 1H), 7.28–7.23 (m, 1H), 6.62–6.55 (m, 2H), 3.84 (s, 3H), 3.81 (s, 3H), 3.05–2.94 (m, 4H), 2.00 (p, *J* = 7.7 Hz, 2H) ppm. ^13^C NMR (126 MHz, CD_2_Cl_2_): δ = 165.1, 164.3, 161.4, 157.9, 153.3 (d, *J* = 244.3 Hz), 148.9, 139.1, 138.0 (d, *J* = 7.7 Hz), 131.4, 125.8 (d, *J* = 2.8 Hz), 122.9 (d, *J* = 10.6 Hz), 122.7, 121.4 (d, *J* = 1.5 Hz), 116.5 (d, *J* = 19.8 Hz), 105.4, 99.3, 55.9, 55.8, 38.0, 36.2, 20.6 ppm. qH NMR (400 MHz, DMSO-*d*_6_, ethyl 4-(dimethylamino)benzoate as reference): purity = 95.3%. MS (+APCI): *m*/*z* 385.5 ([M]^+^). HRMS (DEP/EI): *m*/*z* calculated 385.1320 for C_21_H_20_FNO_5_, found 385.1327 ([M]^+^).

#### 2-[(3-Fluoro-3’,4’-dimethoxy-[1,1’-biphenyl]-4-yl)carbamoyl]cyclopent-1-ene-1-carboxylic acid (**39**)

Preparation according to GP2, using arylbromide **14** (82 mg, 0.25 mmol) and 3,4-dimethoxyphenylboronic acid (**39a**, 42 µl, 0.28 mmol). Further purification was performed by CC (cyclohexane/EtOAc) to obtain compound **39** (53 mg, yield: 55%) as a yellow solid. R_f_ (cyclohexane/EtOAc = 7:3 + 1% FA) = 0.27. ^1^H NMR (500 MHz, DMSO-*d*_*6*_): δ = 10.59 (s, 1H), 8.02 (t, *J* = 8.4 Hz, 1H), 7.60 (dd, *J* = 12.5, 2.1 Hz, 1H), 7.49 (dd, *J* = 8.4, 2.1 Hz, 1H), 7.27–7.20 (m, 2H), 7.02 (d, *J* = 8.2 Hz, 1H), 3.85 (s, 3H), 3.79 (s, 3H), 2.84–2.76 (m, 2H), 2.72–2.66 (m, 2H), 1.89 (p, *J* = 7.8 Hz, 2H) ppm. ^13^C NMR (126 MHz, DMSO-*d*_*6*_): δ = 166.2, 164.7, 153.8 (d, *J* = 244.8 Hz), 149.1, 148.8, 147.0, 137.4 (d, *J* = 7.2 Hz), 135.0, 131.1, 124.5 (d, *J* = 11.9 Hz), 124.0, 121.9 (d, *J* = 2.8 Hz), 118.7, 113.1 (d, *J* = 20.3 Hz), 112.1, 110.2, 55.6, 55.6, 36.4, 34.4, 21.1 ppm. qH NMR (400 MHz, DMSO-*d*_6_, ethyl 4-(dimethylamino)benzoate as reference): purity = 95.2%. MS (+APCI): *m*/*z* 385.7 ([M + H]^+^). HRMS (GC/EI): *m*/*z* calculated 385.1320 for C_21_H_20_FNO_5_, found 385.1316 ([M]^+^).

#### 2-[(3-Fluoro-3’,5’-dimethoxy-[1,1’-biphenyl]-4-yl)carbamoyl]cyclopent-1-ene-1-carboxylic acid (**40**)

Preparation according to GP2, using arylbromide **14** (82 mg, 0.25 mmol) and 3,5-dimethoxyphenylboronic acid (**40a**, 50 mg, 0.28 mmol). Further purification was performed by CC (cyclohexane/EtOAc) to obtain compound **40** (73 mg, yield: 76%) as a yellow solid. R_f_ (cyclohexane/EtOAc = 7:3 + 1% FA) = 0.40. ^1^H NMR (500 MHz, acetone-*d*_*6*_): δ = 10.44 (s, 1H), 8.29–8.18 (m, 1H), 7.57–7.47 (m, 2H), 6.83 (d, *J* = 2.2 Hz, 2H), 6.51 (t, *J* = 2.2 Hz, 1H), 3.86 (s, 6H), 3.05–2.99 (m, 2H), 2.92–2.87 (m, 2H), 1.94 (p, *J* = 7.7 Hz, 2H) ppm. ^13^C NMR (126 MHz, acetone-*d*_*6*_): δ = 166.5, 164.8, 162.4, 155.0 (d, *J* = 245.8 Hz), 146.3, 142.1 (d, *J* = 1.9 Hz), 140.5, 139.7 (d, *J* = 7.5 Hz), 126.1 (d, *J* = 11.4 Hz), 124.8, 123.6 (d, *J* = 3.2 Hz), 114.5 (d, *J* = 20.6 Hz), 105.7, 100.6, 55.8, 37.3, 37.2, 21.3 ppm. qH NMR (400 MHz, DMSO-*d*_6_, ethyl 4-(dimethylamino)benzoate as reference): purity = 96.7%. MS (+APCI): *m*/*z* 385.7 ([M + H]^+^). HRMS (GC/EI): *m*/*z* calculated 385.1320 for C_21_H_20_FNO_5_, found 385.1302 ([M]^+^).

#### 2-[(2’,3’-Dichloro-3-fluoro-[1,1’-biphenyl]-4-yl)carbamoyl]cyclopentane-1-carboxylic acid (**41**)

Preparation according to GP4, using arylbromide **14** (66 mg, 0.20 mmol) and 2,3-dichlorophenylboronic acid (**41a**, 42 mg, 0.22 mmol). Further purification was performed by preparative HPLC (H_2_O/ACN) to obtain compound **41** (28 mg, yield: 36%) as a colorless solid. R_f_ (cyclohexane/EtOAc = 7:3 + 1% FA) = 0.45. ^1^H NMR (500 MHz, MeOD-*d*_4_): δ = 8.25 (t, *J* = 8.2 Hz, 1H), 7.54 (dd, *J* = 7.8, 1.8 Hz, 1H), 7.34 (t, *J* = 7.7 Hz, 1H), 7.30 (dd, *J* = 7.6, 1.8 Hz, 1H), 7.21 (dd, *J* = 11.7, 2.0 Hz, 1H), 7.19 – 7.13 (m, 1H), 2.93 – 2.80 (m, 4H), 1.82 (p, *J* = 7.7 Hz, 2H) ppm. ^13^C NMR (126 MHz, MeOD-*d*_4_): δ = 174.8, 165.9, 154.6 (d, *J* = 246.7 Hz), 147.2, 142.8 (d, *J* = 1.8 Hz), 139.7, 137.1 (d, *J* = 7.5 Hz), 134.6, 131.8, 130.9, 130.9, 128.9, 127.9 (d, *J* = 11.3 Hz), 126.3 (d, *J* = 3.3 Hz), 124.0 (d, *J* = 2.3 Hz), 117.3 (d, *J* = 20.8 Hz), 39.2, 36.9, 21.6 ppm. qH NMR (400 MHz, DMSO-*d*_6_, ethyl 4-(dimethylamino)benzoate as reference): purity = 97.1%. MS (+APCI): *m*/*z* 393.4 ([M]^+^). HRMS (FIA/ESI): *m*/*z* calculated 416.0227 for C_19_H_14_Cl_2_FNO_3_Na, found 416.0224 ([M + Na]^+^).

#### 2-[(3’-Chloro-3-fluoro-2’-trifluoromethoxy-[1,1’-biphenyl]-4-yl)carbamoyl]cyclopent-1-ene-1-carboxylic acid (**42**)

Preparation according to GP2, using arylbromide **14** (49 mg, 0.15 mmol) and 3,5-dimethoxyphenylboronic acid (**42a**, 40 mg, 0.17 mmol). Further purification was performed by CC (cyclohexane/EtOAc + 2% FA) to obtain compound **42** (47 mg, yield: 71%) as a colorless solid. R_f_ (cyclohexane/EtOAc = 7:3 + 1% FA) = 0.36. ^1^H NMR (500 MHz, CD_2_Cl_2_): δ = 8.33 (t, *J* = 8.5 Hz, 1H), 8.26 (s, 1H), 7.46–7.37 (m, 2H), 7.37–7.18 (m, 3H), 3.08–2.94 (m, 4H), 2.02 (p, *J* = 7.7 Hz, 2H) ppm. ^13^C NMR (126 MHz, CD_2_Cl_2_): δ = 165.2, 164.1, 153.1 (d, *J* = 246.0 Hz), 148.7, 146.2 (q, *J* = 1.7 Hz), 141.3 (d, *J* = 2.0 Hz), 139.4, 137.3 (d, *J* = 8.0 Hz), 129.9, 128.1, 126.4, 126.4 (d, *J* = 3.2 Hz), 124.8 (d, *J* = 10.3 Hz), 123.0, 122.4, 121.0 (q, *J* = 258.8 Hz), 116.9 (d, *J* = 20.3 Hz), 37.9, 36.2, 20.6 ppm. qH NMR (400 MHz, DMSO-*d*_6_, ethyl 4-(dimethylamino)benzoate as reference): purity = 95.1%. MS (+APCI): *m*/*z* 443.3 ([M]^+^). HRMS (DEP/EI): *m*/*z* calculated 443.0542 for C_20_H_14_ClF_4_NO_4_, found 443.0550 ([M]^+^).

#### 2-{[4-(2,2-Difluorobenzo[d][1,3]dioxol-4-yl)-2-fluorophenyl]carbamoyl}cyclopent-1-ene-1-carboxylic acid (**43**)

Preparation according to GP2, using arylbromide **14** (66 mg, 0.20 mmol) and 2,2-difluoro[1,3]dioxole-4-boronic acid (**43a**, 44 mg, 0.22 mmol). Further purification was performed by reversed-phase CC (H_2_O/ACN) to obtain compound **43** (63 mg, yield: 78%) as a yellow solid. R_f_ (cyclohexane/EtOAc = 7:3 + 1% FA) = 0.39. ^1^H NMR (500 MHz, CD_2_Cl_2_): δ = 8.39 (t, *J* = 8.2 Hz, 1H), 8.25 (s, 1H), 7.66–7.56 (m, 2H), 7.32 (dd, *J* = 8.1, 1.2 Hz, 1H), 7.21 (t, *J* = 8.0 Hz, 1H), 7.11 (dd, *J* = 8.0, 1.2 Hz, 1H), 3.08–2.95 (m, 4H), 2.01 (p, *J* = 7.8 Hz, 2H) ppm. ^13^C NMR (126 MHz, CD_2_Cl_2_): δ = 165.2, 163.9, 153.6 (d, *J* = 245.7 Hz), 148.9, 144.6, 141.2, 139.3, 132.9 (d, *J* = 8.0 Hz), 131.9 (t, *J* = 254.8 Hz), 125.0 (d, *J* = 10.4 Hz), 124.7, 124.6 (d, *J* = 3.3 Hz), 123.4, 123.1, 122.3, 115.1 (d, *J* = 20.8 Hz), 109.6, 37.9, 36.2, 20.6 ppm. qH NMR (400 MHz, DMSO-*d*_6_, ethyl 4-(dimethylamino)benzoate as reference): purity = 99.4%. MS (+APCI): *m*/*z* 406.5 ([M + H]^+^). HRMS (GC/EI): *m*/*z* calculated 405.0819 for C_20_H_14_F_3_NO_5_, found 405.0816 ([M]^+^).

#### 2-[(3’-Methoxy-3-methyl-[1,1’-biphenyl]-4-yl)carbamoyl]cyclopent-1-ene-1-carboxylic acid (**44**)

Preparation according to GP2, using arylbromide **44b** (81 mg, 0.25 mmol) and 3-methoxyphenylboronic acid (**4c**, 43 mg, 0.28 mmol). Further purification was performed by preparative HPLC (H_2_O/ACN) to obtain compound **44** (44 mg, yield: 50%) as a yellow solid. R_f_ (cyclohexane/EtOAc = 7:3 + 1% FA) = 0.28. ^1^H NMR (500 MHz, CD_2_Cl_2_): δ = 7.86–7.70 (m, 2H), 7.55–7.47 (m, 2H), 7.36 (t, *J* = 8.0 Hz, 1H), 7.24–7.15 (m, 1H), 7.15–7.09 (m, 1H), 6.96–6.88 (m, 1H), 3.86 (s, 3H), 3.09–2.90 (m, 4H), 2.36 (s, 3H), 2.01 (p, *J* = 7.7 Hz, 2H) ppm. ^13^C NMR (126 MHz, CD_2_Cl_2_): δ = 165.5, 164.0, 160.5, 149.0, 141.9, 140.3, 138.8, 133.5, 131.9, 130.3, 129.8, 125.9, 124.9, 119.7, 113.3, 113.1, 55.7, 37.8, 36.2, 20.6, 18.1 ppm. qH NMR (400 MHz, DMSO-*d*_6_, ethyl 4-(dimethylamino)benzoate as reference): purity = 99.6%. MS (+EI): *m*/*z* 351.21 ([M]^+^). HRMS (GC/EI): *m*/*z* calculated 351.1465 for C_21_H_21_NO_4_, found 351.1460 ([M]^+^).

#### 2-[(4-Bromo-2-methylphenyl)carbamoyl]cyclopent-1-ene-1-carboxylic acid (**44b**)

Preparation according to GP1, using 1-cyclopentene-1,2-dicarboxylic acid anhydride (**4a**, 73 µl, 0.72 mmol) and 4-bromo-2-methylaniline **44a** (90 µl, 0.72 mmol) to obtain compound **44b** (0.23 g, yield: 99%) as a colorless solid. R_f_ (cyclohexane/EtOAc = 1:1 + 2% FA) = 0.57. ^1^H NMR (500 MHz, DMSO-*d*_6_): δ = 12.90 (s, 1H), 9.90 (s, 1H), 7.46–7.40 (m, 2H), 7.39–7.33 (m, 1H), 2.83–2.75 (m, 2H), 2.71–2.63 (m, 2H), 2.22 (s, 3H), 1.90 (p, *J* = 7.7 Hz, 2H) ppm. ^13^C NMR (126 MHz, DMSO-*d*_6_): δ = 165.8, 165.0, 147.2, 135.3, 134.6, 134.5, 132.7, 128.7, 126.8, 117.4, 36.4, 34.1, 21.2, 17.5 ppm. MS (+APCI): *m*/*z* 223.5 ([M + H]^+^).

#### 2-[(3,3’-Dimethoxy-[1,1’-biphenyl]-4-yl)carbamoyl]cyclopent-1-ene-1-carboxylic acid (**45**)

Preparation according to GP2, using arylbromide **45b** (85 mg, 0.25 mmol) and 3-methoxyphenylboronic acid (**4c**, 43 mg, 0.28 mmol). Further purification was performed by preparative HPLC (H_2_O/ACN) to obtain compound **45** (36 mg, yield: 39%) as a yellow solid. R_f_ (cyclohexane/EtOAc = 7:3 + 1% FA) = 0.27. ^1^H NMR (500 MHz, CD_2_Cl_2_): δ = 8.53 (s, 1H), 8.39 (d, *J* = 8.4 Hz, 1H), 7.37 (t, *J* = 8.0 Hz, 1H), 7.26 (dd, *J* = 8.4, 2.0 Hz, 1H), 7.24–7.17 (m, 2H), 7.17–7.11 (m, 1H), 6.95–6.87 (m, 1H), 4.01 (s, 3H), 3.86 (s, 3H), 3.06–3.00 (m, 2H), 3.00–2.93 (m, 2H), 2.00 (p, *J* = 7.7 Hz, 2H) ppm. ^13^C NMR (126 MHz, CD_2_Cl_2_): δ = 164.7, 164.0, 160.5, 149.5, 148.9, 142.2, 139.5, 139.2, 130.3, 125.5, 121.2, 120.1, 119.7, 113.2, 113.2, 109.6, 56.7, 55.7, 37.9, 36.0, 20.5 ppm. qH NMR (400 MHz, DMSO-*d*_6_, ethyl 4-(dimethylamino)benzoate as reference): purity = 99.9%. MS (+APCI): *m*/*z* 367.6 ([M + H]^+^). HRMS (GC/EI): *m*/*z* calculated 367.1414 for C_21_H_21_NO_5_, found 367.1419 ([M]^+^).

#### 2-[(4-Bromo-2-methoxyphenyl)carbamoyl]cyclopent-1-ene-1-carboxylic acid (**45b**)

Preparation according to GP1, using 1-cyclopentene-1,2-dicarboxylic acid anhydride (**4a**, 73 µl, 0.72 mmol) and 4-bromo-2-methoxyaniline **45a** (98 µl, 0.72 mmol) to obtain compound **45b** (0.24 g, yield: 98%) as a yellow solid. R_f_ (cyclohexane/EtOAc = 1:1 + 1% FA) = 0.60. ^1^H NMR (500 MHz, DMSO-*d*_6_): δ = 13.01 (s, 1H), 10.17 (s, 1H), 8.06 (d, *J* = 8.6 Hz, 1H), 7.24 (d, *J* = 2.2 Hz, 1H), 7.12 (dd, *J* = 8.7, 2.2 Hz, 1H), 3.85 (s, 3H), 2.81–2.74 (m, 2H), 2.73–2.65 (m, 2H), 1.85 (p, *J* = 7.7 Hz, 2H) ppm. ^13^C NMR (126 MHz, DMSO-*d*_6_): δ = 166.4, 164.0, 150.4, 146.7, 135.2, 126.7, 123.0, 122.6, 116.0, 114.4, 56.3, 36.4, 34.8, 20.9 ppm. MS (+APCI): *m*/*z* 339.5 ([M + H]^+^).

#### 2-[(3’-Methoxy-3-trifluoromethyl-[1,1’-biphenyl]-4-yl)carbamoyl]cyclopent-1-ene-1-carboxylic acid (**46**)

Preparation according to GP2, using arylbromide **46b** (64 mg, 0.17 mmol) and 3-methoxyphenylboronic acid (**4c**, 29 mg, 0.19 mmol). Further purification was performed by preparative HPLC (H_2_O/ACN) to obtain compound **46** (30 mg, yield: 44%) as a colorless solid. R_f_ (cyclohexane/EtOAc = 7:3 + 1% FA) = 0.31. ^1^H NMR (500 MHz, acetone-*d*_6_) δ = 8.02–7.91 (m, 3H), 7.42 (t, *J* = 7.9 Hz, 1H), 7.33–7.25 (m, 2H), 7.03–6.97 (m, 1H), 3.08–2.99 (m, 2H), 2.93–2.85 (m, 2H), 1.97 (p, *J* = 7.7 Hz, 2H) ppm. ^13^C NMR (126 MHz, acetone-*d*_6_): δ = 166.0, 165.7, 161.3, 144.7, 142.3, 141.2, 140.4, 134.5, 132.1, 131.0, 130.09, 130.06, 125.6 (q, *J* = 5.2 Hz), 124.7 (q, *J* = 272.8 Hz), 120.2, 114.7, 113.4, 55.7, 37.1, 37.0, 21.3 ppm. qH NMR (400 MHz, DMSO-*d*_6_, ethyl 4-(dimethylamino)benzoate as reference): purity = 96.3%. MS (+APCI): *m*/*z* 406.3 ([M + H]^+^). HRMS (FIA/ESI): *m*/*z* calculated 428.1080 for C_21_H_18_F_3_NO_4_Na, found 428.1077 ([M + Na]^+^).

#### 2-{[4-Bromo-2-(trifluoromethyl)phenyl]carbamoyl}cyclopent-1-ene-1-carboxylic acid (**46b**)

Preparation according to GP1, using 1-cyclopentene-1,2-dicarboxylic acid anhydride (**4a**, 73 µl, 0.72 mmol) and 4-bromo-2-trifluoromethylaniline **46a** (0.10 mL, 0.72 mmol). Further purification was performed by reversed-phase CC (H_2_O/ACN) to obtain compound **46b** (0.13 g, yield: 49%) as a yellow solid. R_f_ (cyclohexane/DCM = 7:3 + 2% FA) = 0.37. ^1^H NMR (500 MHz, CD_2_Cl_2_): δ = 8.12 (s, 1H), 8.05–7.99 (m, 1H), 7.88–7.84 (m, 1H), 7.82–7.76 (m, 1H), 3.02–2.94 (m, 4H), 2.01 (p, *J* = 7.8 Hz, 2H) ppm. ^13^C NMR (126 MHz, CD_2_Cl_2_): δ = 165.6, 163.8, 149.5, 138.7, 136.6, 132.7 (q, *J* = 1.3 Hz), 130.1 (q, *J* = 5.3 Hz), 127.8, 123.9 (q, *J* = 30.7 Hz), 123.3 (q, *J* = 273.7 Hz), 120.2, 38.0, 35.9, 20.5 ppm. MS (+APCI): *m*/*z* 378.1 ([M + H]^+^).

#### 2-[(3-Chloro-3’-methoxy-[1,1’-biphenyl]-4-yl)carbamoyl]cyclopent-1-ene-1-carboxylic acid (**47**)

Preparation according to GP2, using arylbromide **47b** (99 mg, 0.29 mmol) and 3-methoxyphenylboronic acid (**4c**, 50 mg, 0.32 mmol). Further purification was performed by reversed-phase CC (H_2_O/ACN) to obtain compound **47** (38 mg, yield: 35%) as a yellow solid. R_f_ (*iso*-hexane/EtOAc = 7:3 + 1% FA) = 0.29. ^1^H NMR (500 MHz, acetone-*d*_6_): δ = 10.02 (s, 1H), 8.22 (d, *J* = 8.5 Hz, 1H), 7.79 (d, *J* = 2.1 Hz, 1H), 7.67 (dd, *J* = 8.5, 2.1 Hz, 1H), 7.39 (t, *J* = 7.9 Hz, 1H), 7.31–7.20 (m, 2H), 7.01–6.92 (m, 1H), 3.88 (s, 3H), 3.10–3.00 (m, 2H), 2.94–2.85 (m, 2H), 1.96 (p, *J* = 7.7 Hz, 2H) ppm. ^13^C NMR (126 MHz, CDCl_3_): δ = 164.6, 163.7, 160.3, 149.6, 140.3, 140.0, 138.5, 131.9, 130.3, 127.9, 126.8, 124.5, 122.7, 119.5, 113.6, 112.9, 55.5, 37.8, 35.8, 20.27 ppm. qH NMR (400 MHz, acetone-*d*_6_, ethyl 4-(dimethylamino)benzoate as reference): purity = 99.7%. MS (+APCI): *m*/*z* 371.7 ([M + H]^+^). HRMS (GC/EI): *m*/*z* calculated 353.0813 for C_20_H_16_ClNO_3_, found 353.0811 ([M – H_2_O]^+^).

#### 2-[(4-Bromo-2-chlorophenyl)carbamoyl]cyclopent-1-ene-1-carboxylic acid (**47b**)

Preparation according to GP1, using 1-cyclopentene-1,2-dicarboxylic acid anhydride (**4a**, 73 µl, 0.72 mmol) and 4-bromo-2-chloroaniline **47a** (0.15 g, 0.72 mmol) to obtain compound **47b** (0.25 g, yield: 100%) as a yellow solid. R_f_ (*iso*-hexane/EtOAc = 7:3 + 1% FA) = 0.29. ^1^H NMR (400 MHz, acetone-*d*_6_): δ = 10.16 (s, 1H), 8.14 (d, *J* = 8.8 Hz, 1H), 7.70 (d, *J* = 2.3 Hz, 1H), 7.55 (dd, *J* = 8.8, 2.3 Hz, 1H), 3.05–2.95 (m, 2H), 2.93–2.84 (m, 2H), 1.94 (p, *J* = 7.8 Hz, 2H) ppm. ^13^C NMR (101 MHz, acetone-*d*_6_): δ = 166.2, 164.9, 146.1, 140.3, 135.1, 132.6, 131.5, 127.3, 126.6, 118.1, 37.2, 37.0, 21.4 ppm. MS (+APCI): *m*/*z* 343.4 ([M + H]^+^).

#### 2-[(2-Fluoro-3’-methoxy-[1,1’-biphenyl]-4-yl)carbamoyl]cyclopent-1-ene-1-carboxylic acid (**48**)

Preparation according to GP2, using arylbromide **48b** (82 mg, 0.25 mmol) and 3-methoxyphenylboronic acid (**4c**, 42 mg, 0.28 mmol). Further purification was performed by reversed-phase CC (H_2_O/MeOH) to obtain compound **48** (82 mg, yield: 92%) as a colorless solid. R_f_ (cyclohexane/EtOAc = 7:3 + 1% FA) = 0.32. ^1^H NMR (400 MHz, MeOD-*d*_*4*_): δ = 7.66 (dd, *J* = 13.0, 2.0 Hz, 1H), 7.48–7.29 (m, 3H), 7.13–7.03 (m, 2H), 6.95–6.89 (m, 1H), 3.83 (s, 3H), 2.95–2.86 (m, 2H), 2.86–2.76 (m, 2H), 2.00 (p, *J* = 7.7 Hz, 2H) ppm. ^13^C NMR (126 MHz, MeOD-*d*_*4*_): δ = 168.0, 167.5, 161.2, 160.9 (d, *J* = 245.5 Hz), 149.0, 140.5 (d, *J* = 11.2 Hz), 138.1 (d, *J* = 0.9 Hz), 137.2, 131.8 (d, *J* = 4.7 Hz), 130.5, 125.9 (d, *J* = 13.7 Hz), 122.3 (d, *J* = 3.1 Hz), 117.2 (d, *J* = 3.3 Hz), 115.6 (d, *J* = 3.2 Hz), 114.0, 109.0 (d, *J* = 28.3 Hz), 55.7, 37.7, 35.8, 22.6 ppm. qH NMR (400 MHz, DMSO-*d*_6_, ethyl 4-(dimethylamino)benzoate as reference): purity = 98.3%. MS (+APCI): *m*/*z* 355.9 ([M + H]^+^). HRMS (GC/EI): *m*/*z* calculated 355.1214 for C_20_H_18_FNO_4_, found 355.1213 ([M]^+^).

#### 2-[(4-Bromo-3-fluorophenyl)carbamoyl]cyclopent-1-ene-1-carboxylic acid (**48b**)

Preparation according to GP1, using 1-cyclopentene-1,2-dicarboxylic acid anhydride (**4a**, 73 µl, 0.72 mmol) and 4-bromo-3-fluoroaniline **48a** (81 µl, 0.72 mmol) to obtain compound **48b** (0.22 g, yield: 93%) as a colorless solid. R_f_ (cyclohexane/EtOAc = 1:1 + 1% FA) = 0.64. ^1^H NMR (500 MHz, DMSO-*d*_6_): δ = 12.68 (s, 1H), 10.54 (s, 1H), 7.76 (dd, *J* = 11.4, 2.3 Hz, 1H), 7.63 (t, *J* = 8.4 Hz, 1H), 7.33 (dd, *J* = 8.8, 2.3 Hz, 1H), 2.79–2.71 (m, 2H), 2.68–2.60 (m, 2H), 1.92 (p, *J* = 7.7 Hz, 2H) ppm. ^13^C NMR (126 MHz, DMSO-*d*_6_): δ = 165.8, 165.3, 158.0 (d, *J* = 242.2 Hz), 147.4, 140.2 (d, *J* = 10.1 Hz), 133.8, 133.4, 116.7 (d, *J* = 3.2 Hz), 107.2 (d, *J* = 27.2 Hz), 100.9 (d, *J* = 20.9 Hz), 36.2, 33.4, 21.5 ppm. MS (+APCI): *m*/*z* 327.5 ([M + H]^+^).

#### 2-[(3-Chloro-2-fluoro-3’-methoxy-[1,1’-biphenyl]-4-yl)carbamoyl]cyclopent-1-ene-1-carboxylic acid (**49**)

Preparation according to GP2, using arylbromide **49b** (54 mg, 0.15 mmol) and 3-methoxyphenylboronic acid (**4c**, 26 mg, 0.17 mmol). Further purification was performed by preparative HPLC (H_2_O/ACN) to obtain compound **49** (9 mg, yield: 15%) as a colorless solid. R_f_ (cyclohexane/EtOAc = 7:3 + 1% FA) = 0.27. ^1^H NMR (500 MHz, MeOD-*d*_*4*_): δ = 7.89 (d, *J* = 8.7 Hz, 1H), 7.43 (t, *J* = 8.4 Hz, 1H), 7.37 (t, *J* = 8.0 Hz, 1H), 7.13–7.05 (m, 2H), 6.97 (dd, *J* = 8.2, 2.6 Hz, 1H), 3.84 (s, 3H), 2.97–2.90 (m, 2H), 2.90–2.83 (m, 2H), 1.99 (p, *J* = 7.6 Hz, 2H) ppm. ^13^C NMR (126 MHz, MeOD-*d*_*4*_): δ = 167.0, 161.3, 156.6 (d, *J* = 248.0 Hz), 146.9–146.7 (m), 139.9–139.6 (m), 137.3, 136.8, 130.7, 129.4 (d, *J* = 4.2 Hz), 128.1 (d, *J* = 10.9 Hz), 122.3 (d, *J* = 3.0 Hz), 121.5, 115.6 (d, *J* = 3.0 Hz), 114.7, 55.8, 37.6, 36.7, 22.3 ppm. qH NMR (400 MHz, DMSO-*d*_6_, ethyl 4-(dimethylamino)benzoate as reference): purity = 99.6%. MS (+APCI): *m*/*z* 389.5 ([M]^+^). HRMS (DEP/EI): *m*/*z* calculated 389.0825 for C_20_H_17_ClFNO_4_, found 389.0818 ([M]^+^).

#### 2-[(4-Bromo-2-chloro-3-fluorophenyl)carbamoyl]cyclopent-1-ene-1-carboxylic acid (**49b**)

Preparation according to GP1, using 1-cyclopentene-1,2-dicarboxylic acid anhydride (**4a**, 73 µl, 0.72 mmol) and 4-bromo-2-chloro-3-fluoroaniline **49a** (90 µl, 0.72 mmol) to obtain compound **49b** (0.16 g, yield: 59%) as a colorless solid. R_f_ (cyclohexane/EtOAc = 1:1 + 1% FA) = 0.50. ^1^H NMR (500 MHz, DMSO-*d*_6_): δ = 13.07 (s, 1H), 10.51 (s, 1H), 7.79–7.56 (m, 2H), 2.83–2.74 (m, 2H), 2.73–2.65 (m, 2H), 1.90 (p, *J* = 7.7 Hz, 2H) ppm. ^13^C NMR (126 MHz, DMSO-*d*_6_): δ = 166.1, 165.0, 154.3 (d, *J* = 243.9 Hz), 146.6, 136.3, 135.3, 131.1, 121.6, 114.6 (d, *J* = 18.3 Hz), 104.1 (d, *J* = 21.2 Hz), 36.3, 34.2, 21.1 ppm. MS (+APCI): *m*/*z* 361.1 ([M + H]^+^).

### 2-{[5-(3-Methoxyphenyl)thiophen-2-yl]carbamoyl}cyclopent-1-ene-1-carboxylic acid (**50**)

Preparation according to GP1, using 1-cyclopentene-1,2-dicarboxylic acid anhydride (**4a**, 16 µl, 0.16 mmol) and amine **50c** (33 mg, 0.16 mmol). Further purification was performed by preparative HPLC (H_2_O/ACN) to obtain compound **50** (25 mg, yield: 46%) as a yellow solid. R_f_ (cyclohexane/EtOAc = 7:3 + 1% FA) = 0.26. ^1^H NMR (500 MHz, DMSO-*d*_6_): δ = 7.31–7.24 (m, 2H), 7.15–7.10 (m, 1H), 7.08 (t, *J* = 2.1 Hz, 1H), 6.81–6.75 (m, 1H), 6.60–6.55 (m, 1H), 3.79 (s, 3H), 2.79–2.70 (m, 4H), 1.66 (p, *J* = 7.7 Hz, 2H) ppm. ^13^C NMR (126 MHz, DMSO-*d*_6_): δ = 168.8, 161.0, 159.7, 146.9, 141.0, 137.0, 136.2, 132.3, 130.1, 121.2, 116.9, 112.1, 111.1, 109.7, 55.1, 38.4, 35.7, 19.8 ppm. qH NMR (400 MHz, DMSO-*d*_6_, ethyl 4-(dimethylamino)benzoate as reference): purity = 95.9%. MS (+APCI): *m*/*z* 343.6 ([M]^+^). HRMS (FIA/EI): *m*/*z* calculated 342.0795 for C_18_H_16_NO_4_S, found 342.0803 ([M – H]^-^).

#### tert-Butyl [5-(3-methoxyphenyl)thiophen-2-yl]carbamate (**50b**)

Preparation according to GP2, using *tert*-butyl (5-bromothiophen-2-yl)carbamate **50a** (0.28 g, 1.0 mmol) and 3-methoxyphenylboronic acid (**4c**, 0.17 g, 1.1 mmol). Further purification was performed by reversed-phase CC (H_2_O/ACN) to obtain compound **50b** (0.25 g, yield: 82%) as a colorless solid. R_f_ (cyclohexane/EtOAc = 7:3 + 1% TEA) = 0.62. ^1^H NMR (500 MHz, acetone-*d*_*6*_): δ = 9.43 (s, 1H), 7.27 (t, *J* = 7.9 Hz, 1H), 7.19–7.10 (m, 3H), 6.83–6.77 (m, 1H), 6.61 (d, *J* = 3.9 Hz, 1H), 3.84 (s, 3H), 1.51 (s, 9H) ppm. ^13^C NMR (126 MHz, acetone-*d*_*6*_): δ = 161.2, 153.3, 142.2, 137.3, 134.4, 130.8, 121.9, 118.0, 113.1, 111.6, 110.9, 81.1, 55.5, 28.4 ppm. MS (+APCI): *m*/*z* 305.5 ([M]^+^).

#### 5-(3-Methoxyphenyl)thiophen-2-amine (**50c**)

The Boc-protected amine **50a** (0.25 g, 0.81 mmol) was dissolved in methylenchloride (3 mL) and TFA (0.31 mL, 4.0 mmol) was added. The mixture was stirred at rt for 4 h and aqueous saturated sodium carbonate solution (5 mL) was added. The aqueous layer was extracted with methylenchloride (3x10 mL). The organic layers were combined, dried over Na_2_SO_4_, filtered and evaporated. The crude product was purified by CC (cyclohexane/EtOAc + 2% TEA) to afford compound **50c** (76 mg, yield: 46%) as a brown oil. R_f_ (cyclohexane/EtOAc = 7:3 + 1% TEA) = 0.38. ^1^H NMR (400 MHz, acetone-*d*_*6*_): δ = 7.23–7.15 (m, 1H), 7.06–6.93 (m, 3H), 6.71 (ddd, *J* = 8.2, 2.5, 0.9 Hz, 1H), 6.04–5.97 (m, 1H), 5.17 (s, 2H), 3.80 (s, 3H) ppm. ^13^C NMR (101 MHz, acetone-*d*_*6*_): δ = 161.1, 154.8, 137.7, 130.6, 128.1, 123.8, 117.4, 111.8, 110.3, 107.1, 55.4 ppm. MS (+APCI): *m*/*z* 205.8 ([M]^+^).

#### 2-[(3-Chloro-3’-trifluoromethoxy-[1,1’-biphenyl]-4-yl)carbamoyl]cyclopent-1-ene-1-carboxylic acid (**51**)

Preparation according to GP2, using arylbromide **47b** (72 mg, 0.21 mmol) and 3-trifluoromethoxyphenylboronic acid (**51a**, 34 µl, 0.23 mmol). Further purification was performed by reversed-phase CC (H_2_O/ACN) to obtain compound **51** (45 mg, yield: 50%) as a yellow solid. R_f_ (*iso*-hexane/EtOAc = 7:3 + 1% FA) = 0.29. ^1^H NMR (500 MHz, CD_2_Cl_2_): δ = 8.47–8.41 (m, 2H), 7.71 (d, *J* = 2.1 Hz, 1H), 7.60 (dd, *J* = 8.6, 2.1 Hz, 1H), 7.57–7.48 (m, 2H), 7.47–7.43 (m, 1H), 7.30–7.24 (m, 1H), 3.12–3.04 (m, 2H), 3.03–2.95 (m, 2H), 2.03 (p, *J* = 7.7 Hz, 2H) ppm. ^13^C NMR (126 MHz, CD_2_Cl_2_): δ = 165.2, 163.8, 150.1 (q, *J* = 1.7 Hz), 149.6, 141.3, 139.0, 138.6, 132.9, 130.9, 128.2, 127.0, 125.8, 125.4, 123.3, 121.3 (q, *J* = 257.1 Hz), 120.8, 119.9, 38.0, 36.1, 20.6 ppm. qH NMR (400 MHz, CD_2_Cl_2_, ethyl 4-(dimethylamino)benzoate as reference): purity = 95.9%. MS (+APCI): *m*/*z* 426.1 ([M + H]^+^). HRMS (GC/EI): *m*/*z* calculated 425.0636 for C_20_H_15_ClF_3_NO_4_, found 425.0648 ([M]^+^).

#### 2-[(3-Chloro-2’,3’-diethoxy-[1,1’-biphenyl]-4-yl)carbamoyl]cyclopentane-1-carboxylic acid (**52**)

Preparation according to GP2, using arylbromide **47b** (69 mg, 0.20 mmol) and 2,3-dimethoxyphenylboronic acid (**52a**, 34 µl, 0.22 mmol). Further purification was performed by preparative HPLC (H_2_O/ACN) to obtain compound **52** (40 mg, yield: 50%) as a colorless solid. R_f_ (cyclohexane/EtOAc = 7:3 + 1% FA) = 0.41. ^1^H NMR (500 MHz, CD_2_Cl_2_): δ = 8.40 (s, 1H), 8.37 (d, *J* = 8.6 Hz, 1H), 7.69 (d, *J* = 2.0 Hz, 1H), 7.54 (dd, *J* = 8.5, 2.0 Hz, 1H), 7.12 (t, *J* = 8.0 Hz, 1H), 6.97 (dd, *J* = 8.2, 1.5 Hz, 1H), 6.93 (dd, *J* = 7.7, 1.5 Hz, 1H), 3.89 (s, 3H), 3.63 (s, 3H), 3.12–3.04 (m, 2H), 3.03–2.95 (m, 2H), 2.02 (p, *J* = 7.8 Hz, 2H) ppm. ^13^C NMR (126 MHz, CD_2_Cl_2_): δ = 165.2, 163.8, 153.7, 149.6, 147.0, 138.8, 137.7, 133.8, 132.0, 130.4, 129.3, 124.6, 124.4, 122.5, 122.3, 112.8, 60.9, 56.3, 38.0, 36.1, 20.5 ppm. qH NMR (400 MHz, DMSO-*d*_6_, ethyl 4-(dimethylamino)benzoate as reference): purity = 96.0%. MS (+APCI): *m*/*z* 401.4 ([M]^+^). HRMS (DEP/EI): *m*/*z* calculated 401.1031 for C_21_H_20_ClNO_5_, found 401.1031 ([M]^+^).

*<H32>2-{[4-(2,2-Difluorobenzo[d][1,3]dioxol-4-yl)-2-chlorophenyl]carbamoyl}cyclopent-1-ene-1-carboxylic acid (* ***53*** *)*

Preparation according to GP2, using arylbromide **47b** (69 mg, 0.20 mmol) and 2,2-difluoro[1,3]dioxole-4-boronic acid (**53a**, 44 mg, 0.22 mmol). Further purification was performed by reversed-phase CC (H_2_O/ACN) to obtain compound **53** (22 mg, yield: 26%) as a yellow solid. R_f_ (cyclohexane/EtOAc = 7:3 + 1% FA) = 0.40. ^1^H NMR (500 MHz, CD_2_Cl_2_): δ = 8.54–8.38 (m, 2H), 7.85 (d, *J* = 2.0 Hz, 1H), 7.72 (dd, *J* = 8.7, 2.1 Hz, 1H), 7.31 (dd, *J* = 8.1, 1.2 Hz, 1H), 7.21 (t, *J* = 8.0 Hz, 1H), 7.12 (dd, *J* = 7.9, 1.2 Hz, 1H), 3.13–3.05 (m, 2H), 3.05–2.94 (m, 2H), 2.03 (p, *J* = 8.2 Hz, 2H) ppm. ^13^C NMR (126 MHz, CD_2_Cl_2_): δ = 165.3, 163.8, 149.7, 144.6, 141.2, 138.9, 133.2, 132.9, 131.9 (t, *J* = 254.6 Hz), 129.0, 127.8, 125.1, 124.7, 123.13, 123.07, 122.1, 109.6, 38.0, 36.1, 20.6 ppm. qH NMR (400 MHz, DMSO-*d*_6_, ethyl 4-(dimethylamino)benzoate as reference): purity = 97.1%. MS (+APCI): *m*/*z* 421.5 ([M]^+^). HRMS (FIA/ESI): *m*/*z* calculated 444.0421 for C_20_H_14_ClF_2_NO_5_Na, found 444.0417 ([M + Na]^+^).

#### 2-{[4-(2,2-Difluorobenzo[d][1,3]dioxol-4-yl)-2-hlorophenyl]methylcarbamoyl}cyclopent-1-ene-1-carboxylic acid (**54**)

– mixture of rotamers. Preparation according to GP4, using arylbromide **54b** (49 mg, 0.14 mmol) and 2,2-difluoro[1,3]dioxole-4-boronic acid (**54c**, 30 mg, 0.15 mmol). Further purification was performed by preparative HPLC (H_2_O/ACN) to obtain compound **54** (19 mg, yield: 32%) as a colorless solid. R_f_ (cyclohexane/EtOAc = 1:1 + 1% FA) = 0.36. ^1^H NMR (500 MHz, MeOD-*d*_4_): δ = 7.96–7.86 (m, 1H), 7.81–7.54 (m, 2H), 7.49–7.38 (m, 1H), 7.34–7.19 (m, 2H), 3.31–3.27 (m, 3H), 3.00–2.71 (m, 1.2 H), 2.71–2.24 (m, 3.0 H), 2.18–2.07 (m, 0.6H), 1.92–1.74 (m, 0.6 H), 1.64–1.48 (m, 0.6 H) ppm. ^13^C NMR (126 MHz, MeOD-*d*_4_): δ = 172.0, 171.2, 167.2, 167.1, 149.9, 149.3, 145.5, 145.5, 142.0, 140.99, 140.95, 137.4, 136.7, 136.3, 135.5, 134.2, 133.8, 132.9 (t, *J* = 253.4 Hz), 132.9 (t, *J* = 253.4 Hz), 131.8, 131.1, 130.6, 130.6, 128.9, 128.6, 125.9, 125.9, 124.5, 124.5, 123.3, 122.8, 110.8, 110.6, 38.6, 37.2, 37.1, 36.0, 33.9, 33.7, 23.7, 23.5 ppm. qH NMR (400 MHz, DMSO-*d*_6_, ethyl 4-(dimethylamino)benzoate as reference): purity = 96.6%. MS (+APCI): *m*/*z* 435.8 ([M]^+^). HRMS (FIA/ESI): *m*/*z* calculated 458.0577 for C_21_H_16_ClF_2_NO_5_Na, found 458.0576 ([M + Na]^+^).

#### 2-[(4-Bromo-2-chlorophenyl)methylcarbamoyl]cyclopent-1-ene-1-carboxylic acid (**54b**)

– mixture of rotamers. Preparation according to GP1, using 1-cyclopentene-1,2-dicarboxylic acid anhydride (**4a**, 71 µl, 0.70 mmol) and 4-bromo-2-chloro-*N*-methylaniline (**54a**, 0.15 g, 0.70 mmol). Further purification was performed by reversed-phase CC (H_2_O/ACN) to obtain compound **54b** (29 mg, yield: 12%) as a colorless solid. R_f_ (cyclohexane/EtOAc = 7:3 + 1% FA) = 0.14. ^1^H NMR (500 MHz, MeOD-*d*_4_) δ = 7.79–7.77 (m, 0.7H), 7.77–7.75 (m, 0.3H), 7.60–7.56 (m, 0.3H), 7.53–7.49 (m, 0.7H), 7.44 (d, *J* = 8.5 Hz, 0.7H), 7.35 (d, *J* = 8.4 Hz, 0.3H), 3.25 (s, 2H), 3.23 (s, 1H), 2.92–2.82 (m, 0.7H), 2.79–2.71 (m, 0.7H), 2.66–2.46 (m, 1.3H), 2.41–2.28 (m, 1.2H), 2.11 (p, *J* = 7.6 Hz, 0.7H), 1.90–1.77 (m, 0.7H), 1.63–1.52 (m, 0.7H) ppm. ^13^C NMR (126 MHz, MeOD-*d*_4_): δ = 171.8, 171.0, 167.0, 166.9, 149.9, 149.4, 140.4, 140.2, 136.2, 135.3, 134.8, 134.5, 134.1, 134.0, 132.7, 132.6, 132.6, 132.0, 124.1, 123.1, 38.4, 37.2, 37.0, 35.8, 33.8, 33.6, 23.6, 23.5 ppm. MS (+APCI): *m*/*z* 357.4 ([M + H]^+^).

### In vitro Characterization

*Hybrid reporter gene assays*. NR modulation was determined in Gal4 hybrid reporter gene assays in HEK293T cells (German Collection of Microorganisms and Cell Culture GmbH, DSMZ) using pFR-Luc (Stratagene, La Jolla, CA, USA; reporter), pRL-SV40 (Promega, Madison, WI, USA; internal control) and pFA-CMV-hNR-LBD^40–42^ plasmids coding for the hinge region and ligand binding domain of the canonical isoform of the respective NR. HEK293T cells were cultured in Dulbecco’s modified Eagle’s medium (DMEM), high glucose supplemented with 10% fetal calf serum (FCS), sodium pyruvate (1 mM), penicillin (100 U/mL), and streptomycin (100 μg/mL) at 37 °C and 5% CO_2_ and seeded in 96-well plates (3×10^4^ cells/well). After 24 h, medium was changed to Opti-MEM without supplements and cells were transiently transfected using Lipofectamine LTX reagent (Invitrogen, Carlsbad, CA, USA) according to the manufacturer’s protocol. Five hours after transfection, cells were incubated with the test compounds in Opti-MEM supplemented with penicillin (100 U/mL), streptomycin (100 μg/mL) and 0.1% DMSO for 14 h before luciferase activity was measured using the Dual-Glo Luciferase Assay System (Promega) according to the manufacturer’s protocol on a Tecan Spark luminometer (Tecan Deutschland GmbH, Crailsheim, Germany). Firefly luminescence was divided by Renilla luminescence and multiplied by 1000 resulting in relative light units (RLU) to normalize for transfection efficiency and cell growth. Fold activation was obtained by dividing the mean RLU of test compound by the mean RLU of the untreated control and relative activation was calculated by dividing the fold activation of a test sample by the fold activation of the respective reference agonist (1 µM T3 for THRα, 1 µM tretinoin for RARα, 1 µM GW7647 for PPARα, 1 µM pioglitazone for PPARγ, 1 µM L165,041 for PPARδ, 1 µM calcitriol for VDR, 1 µM CITCO for CAR, 1 µM T0901317 for LXRα, 1 µM GW4064 for FXR, 1 µM bexarotene for RXRα, RXRβ and RXRγ). All samples were tested in at least three biologically independent experiments in duplicates. For dose-response curve fitting and calculation of EC_50_ values, the equation “[Agonist] vs. response -- Variable slope (four parameters)” was used in GraphPad Prism (version 7.00, GraphPad Software, La Jolla, CA, USA). Activity of **53** on the Nurr1 mutants I500W/V373W and I500W/M379W was performed with the plasmids described in ref. ^5^.

#### Reporter gene assays for full-length human Nurr1

Activation of full length human Nurr1 was studied in transiently transfected HEK293T cells using the reporter plasmids pFR-Luc-NBRE, pFR-LUC-POMC or pFR-Luc-DR5 each containing one copy of the respective human Nurr1 response element NBRE Nl3, NurRE or DR5^32^. The full length human nuclear receptor Nurr1 (pcDNA3.1-hNurr1-NE; Addgene plasmid #102363; gift from Shu Leong Ho) and, for DR5, RXRα (pSG5-hRXR) were overexpressed. pRL-SV40 (Promega) was used for normalization of transfection efficacy and to observe test compound toxicity. Cell culture, seeding, transient transfection, incubation with test compounds, luciferase activity measurement and data analysis were performed as described for hybrid reporter gene assays. All samples were tested in at least three biologically independent experiments in duplicates.

#### DHODH inhibition assay

Inhibition of DHODH was measured in vitro using an *N-*terminally truncated recombinant DHODH enzyme as described previously^33^. The final assay mixture contained 60 *μ*M 2,6-dichloroindophenol, 50 *μ*M decylubiquinone, 100 *μ*M dihydroorotate, and the DHODH protein whose concentration was adjusted in a way that an average slope of approx. 0.2 AU/min served as the positive control (no inhibitor). Measurements were performed in 50 mM TrisHCl, 150 mM KCl, and 0.1% Triton X-100 at pH 8.0 and at 30 °C with at least six different concentrations of a test compound. The reaction was started by adding dihydroorotate and measuring the absorption at 600 nm for 2 min. Each test compound concentration used for IC_50_ calculation was tested in at least three independent experiments.

#### Evaluation of NR4A-Regulated Gene Expression

N27 rat dopaminergic neural cells (SCC048, Sigma-Aldrich, Darmstadt, Germany) were cultured in RPMI 1640 medium (Gibco, Thermo Fisher Scientific, Waltham, MA, USA) supplemented with 10% FCS, penicillin (100 U/mL), and streptomycin (100 μg/mL) at 37 °C and 5% (v/v) CO_2_ and seeded in 12-well plates (3×10^5^ cells/well). After 8 h, the medium was changed to RPMI 1640 medium supplemented with 0.2% FCS, penicillin (100 U/mL), and streptomycin (100 μg/mL), and the cells were incubated for another 22 h, before the medium was changed again to RPMI 1640 medium supplemented with 0.2% FCS, penicillin (100 U/mL), and streptomycin (100 μg/mL), additionally containing either **4** (1 µM), **53** (0.3, 0.6, 1 µM) or **54** (1, 10 µM) in 0.1% DMSO or 0.1% DMSO alone. After 20 h of incubation, the medium was removed, cells were washed with phosphate-buffered saline (PBS), and after full aspiration of residual liquids immediately frozen at -80 °C until further procession. Each sample was prepared in 6 biologically independent replicates. Total RNA was isolated using peqGOLD Total RNA Kit (VWR International, Darmstadt, Germany) following the manufacturer’s instructions. RNA concentration and purity were assessed using a NanoDrop One UV-vis spectrophotometer (Thermo Fisher Scientific) at 260/280 nm. Right before reverse transcription (RT), RNA was linearized at a concentration of 133 ng/μL at 65 °C for 10 min and then immediately incubated on ice for at least 1 min. Reverse transcription was performed using 2 μg of total RNA, 20 U Recombinant RNasin Ribonuclease Inhibitor (Promega, Mannheim, Germany), 100 U SuperScript IV Reverse Transcriptase including 5× First Strand Buffer and 0.1 M dithiothreitol (Thermo Fisher Scientific), 3.75 ng of linear acrylamide, 625 ng of random hexamer primers (Merck, Darmstadt, Germany), and 11.25 nmol of deoxynucleoside triphosphate mix (2.8 nmol each ATP, TTP, CTP, GTP; Thermo Fisher Scientific) at a volume of 22.45 μL at 50 °C for 10 min and 80 °C for 10 min using a Thermal cycler XT96 (VWR International). A quantitative polymerase chain reaction (qPCR) was conducted using a qTOWERiris (Analytik Jena, Jena, Germany) and a SYBR green-based detection method. 0.2 μL of prepared cDNA was added to 6 pmol each of forward and reverse primer, 0.8 U Taq DNA Polymerase (New England Biolabs, Ipswich, MA, USA), 40 ppm SYBR Green I (Sigma-Aldrich), 15 nmol of deoxynucleoside triphosphate mix (as indicated above), 60 nmol of MgCl_2_, 4 μg of bovine serum albumin (Thermo Fisher Scientific), 20% BioStab PCR Optimizer II (Merck, Darmstadt, Germany), and 10% Taq buffer without detergents (Thermo Fisher Scientific), topped up to a final volume of 20 μL with ddH_2_O. Samples underwent 40 cycles of 15 s denaturation at 95 °C, 15 s of primer annealing at primer-specific temperatures and 20 s of elongation at 68 °C. PCR product specificity was evaluated using a melting curve analysis ranging from 65 to 95 °C. Gene expression was normalized to rGAPDH mRNA expression per sample using the ΔCt-method. The following primers and annealing temperatures were used: rGAPDH (59.4 °C): 5’-CAG CCG CAT CTT CTT GTG C-3’ (fwd), 5’-AAC TTG CCG TGG GTA GAG TC-3’ (rev); rTH (59.4 °C): 5’-TGG GGA GCT GAA GGC TTA TG-3’ (fwd), 5’-AGA GAA TGG GCG CTG GAT AC-3’ (rev); rFLRT2 (59.0 °C): 5’-AAG GAG ACA AGG CTA CCA GAT TAC-3’ (fwd), 5’-GCA AAG CGT GAT GCC AAG TA-3’ (rev); rBDNF (58.0 °C): 5’-AGT CTA GAA CCT TGG GGA CC-3’ (fwd), 5’-GCC TTC ATG CAA CCG AAG TA-3’ (rev); rCRMP4 (58.0 °C): 5’-TGT CCT ACC AGG GCA AGA A-3’ (fwd), 5’-ATC AGA TTG TCT CCA ATT TGC TTT A-3’ (rev); rNRP-1 (62.4 °C): 5’-GGT GAT GAC TTC CAG CTC ACA G-3’ (fwd), 5’-CCG TAT GTC GGG AAC TCT GAT TG-3’ (rev); rSesn3 (62.4 °C): 5’-TCG GCC AAC TAC CTG CTC TG-3’ (fwd), 5’-CGT GTT TGC TTG GAC AAC TTC CT-3’ (rev); rCCND2 (58.9 °C): 5’-CAA GTT TGC CAT GTA CCC GC-3’ (fwd), 5’-GCT TTG AGA CAA TCC ACA TCG G-3’ (rev); rXIAP (61.1 °C): 5’-TCA CTT GGG GAA TCT GTG GTA AG-3’ (fwd), 5’-TCC CAG ATG TTT GGA GCT TTT CT-3’ (rev); rSOD2 (59.4 °C): 5’-CGG GGG CCA TAT CAA TCA CA-3’ (fwd), 5’-TCC AGC AAC TCT CCT TTG GG-3’ (rev).

#### Nurr1 homodimerization assay

Modulation of Nurr1 LBD homodimerization by **53** was studied in a homogenous time-resolved fluorescence resonance energy transfer (HTRF) based assay. Biotinylated recombinant Nurr1 LBD protein and sGFP-Nurr1 LBD protein (FRET acceptor) were expressed and purified as described previously^32^. Terbium cryptate as streptavidin conjugate (Tb-SA; Cisbio Bioassays, Codolet, France) was used as FRET donor for stable coupling to biotinylated recombinant Nurr1 LBD protein. sGFP-Nurr1 LBD protein was titrated from 0.5 µM against a fixed concentration of Tb-SA (0.375 nM) conjugated Nurr1 LBD protein (0.188 nM). Free sGFP was added to keep the total GFP content stable at 0.5 μM. Assay solutions were prepared in HTRF assay buffer (25 mM HEPES pH 7.5, 150 mM KF, 10% (m/v) glycerol, 5 mM DTT) supplemented with 0.1% (w/v) CHAPS as well as 1% DMSO with **53** at 10 µM or DMSO alone as negative control (apo). Samples were equilibrated at RT for 2 h before fluorescence intensities (FI) after excitation at 340 nm were recorded at 520 nm for sGFP acceptor fluorescence and 620 nm for Tb-SA donor fluorescence on a Tecan SPARK plate reader (Tecan Group Ltd.). FI520 nm was divided by FI620 nm and multiplied with 10,000 to give a dimensionless HTRF signal. HTRF data were normalized to the DMSO control of the respective titration experiment to obtain ΔHTRF.

#### Isothermal titration calorimetry (ITC)

ITC experiments were conducted on an Affinity ITC instrument (TA Instruments, New Castle, DE) at 25 °C with a stirring rate of 75 rpm. NR4A1 was used as representative NR4A receptor. NR4A1 LBD protein (20 µM) in buffer (20 mM Tris pH 7.4, 100 mM NaCl, 0.2 mM TCEP) containing 3% DMSO was titrated with compound **53** (100 μM in the same buffer containing 3% DMSO) in 21 injections (1x 1 µL, 20x 4 μL) with an injection interval of 120 s. As control experiments, the compound was titrated to the buffer, and the buffer was titrated to the NR4A1 LBD protein under otherwise identical conditions. The heats of the compound-NR4A1 LBD titrations were corrected using the heats of the compound-buffer titrations. Results were analyzed using NanoAnalyze software (version 3.11.0, TA Instruments, New Castle, DE) with independent binding model.

#### Multiplex toxicity assay

HEK293T cells were grown in DMEM high glucose, supplemented with 10% FCS, sodium pyruvate (1 ×10^−3^ M), penicillin (100 U/mL), and streptomycin (100 μg/mL) at 37 °C and 5% CO_2_ and seeded in 96-well plates (2 × 10^4^ cells per well). The next day, medium with reduced serum content (0.2% FCS) was refreshed and additionally contained 0.1% DMSO with **53** or **54** (0.1, 1 or 10 µM), 0.1% DMSO with bexarotene (100 µM) as positive control, or 0.1% DMSO alone as untreated control. Each sample was prepared in four biologically independent replicates. After incubation for 24 h, the medium was changed to 90 μL culture medium without phenol red (0.2% FCS) and 10 μL Cell Counting Kit-8 solution (CCK-8, MedChem Express #HY-K0301), and absorbance was measured after 2 h incubation at 450 nm on a Tecan Spark Cyto (Tecan Group AG) to assess metabolic activity of the cells. Thereafter, Hoechst33342 (10 μM, #ab228551, Abcam Limited, Cambridge, UK) and Live-or-Dye Nuc-Fix Red (0.05×, Biotium, Inc., Fremont, CA, 1691 USA) were added and incubated for 30 min to detect necrosis. After incubation, a total of 3 fluorescence images per well at 10× magnification were taken to detect Hoechst33342-positive cell nuclei (Ex: 381−400 nm, Em: 414−450 nm) and Live-or-Dye positive cells (Ex: 543−566 nm, Em: 580−611 nm), respectively, using a Tecan Spark Cyto (Tecan Group AG). Necrotic cells were counted using CellProfiler (Version 4.2.6). Reference readings for background correction and detection of autofluorescence were taken at the same wavelengths prior to staining. Before drug administration, after the first medium exchange, 24 h after drug administration, and after fluorescence imaging cell confluence was assessed using the Tecan Spark Cyto, to account for changes in cell confluence due to drug administration and cell handling. Data were normalized to the untreated (DMSO) control for each biological replicate.

#### Determination of aqueous solubility

The aqueous solubility of **53** and **54** was assessed by mixing 1 mg of each test compound with an appropriate volume of water for a theoretical concentration of 2 mM to obtain an oversaturated mixture. The mixture was agitated in a VWR Thermal Shake lite (VWR International GmbH, Darmstadt, Germany) for 24 h at 600 rpm and constant temperature of 25 °C. The supersaturated mixtures were subsequently centrifuged at 15000x g for 15 min (25 °C). Part of the supernatant was taken off for quantification by UV absorbance at 254 nm (**53**) or 260 nm (**54**) with external calibration. The external calibration samples contained 1% DMSO and the test samples were spiked with DMSO to 1% concentration right before the measurement. Absorbance was measured with a Tecan Spark luminometer (Tecan Deutschland GmbH, Crailsheim, Germany). The solubility test was repeated in three independent experiments.

## Supplementary Material

Supporting Information (pdf) containing Figure S1, Table S1, and NMR spectra (1H, 13C, qH) of 4, 8-13 and 16-54.

Molecular formula strings (csv) containing chemical structures and activity data of 4 and 8-54.

Supporting info.

## Figures and Tables

**Table 1:** Deconstruction of the lead 4

ID	structure	EC_50_(Nurr1)^a^ (max. act.)	IC_50_(hDHODH)^b^
**4**	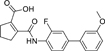	0.4±0.2 μM (3.1±0.4-fold) ^c^	0.61±0.07 gM ^c^
**8**	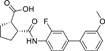	inactive (0.01-30 μM)	>100 μM
**9**	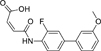	2±1 μM (2.0±0.2-fold)	>100 μM
**10**	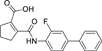	0.25±0.01 μM (4.5±0.2-fold)	0.91±0.03 μM
**11**	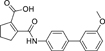	0.28±0.04 μM (2.9±0.2-fold)	0.72±0.02 μM
**12**	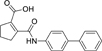	0.20±0.02 μM (4.3±0.2-fold)	1.8±0.3 μM
**13**	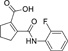	inactive (0.03-30 μM)	>100 μM
**14**	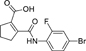	10.3±0.6 μM (6.2±0.4-fold)	13.7±0.5 μM

a Nurr1 agonism was determined in a Gal4 hybrid reporter gene assay.^32^ Data are the mean±SD, n≥3.

b hDHODH inhibition was determined in a colorimetric assay on recombinant human protein.^33^ Data are the mean±SD, n=3.

c Data from ref.^22^.

**Table 2:** Variation of the anisol motif I

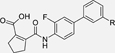			
ID	R	EC_50_(Nurr1)^a^ (max. act.)	IC_50_(hDHODH)^b^
**4**	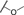	0.4±0.2 μM (3.1±0.4-fold) ^c^	0.61±0.07 μM ^c^
**15**	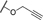	0.11±0.05 μM (6.2±0.4-fold) ^c^	1.7±0.4 μM ^c^
**16**	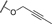	0.15±0.02 μM (7.1±0.5-fold)	1.43±0.01 μM
**17**	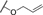	0.060±0.007 μM (3.29±0.05-fold)	0.57±0.02 μM
**18**	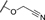	inactive (0.01-30 μM)	>100 μM
**19**	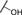	0.18±0.08 μM (2.2±0.3-fold)	1.3±0.1 μM
**20**	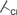	0.15±0.02 μM (2.37±0.06-fold)	2.09±0.08 μM
**21**	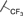	0.07±0.02 μM (2.2±0.2-fold)	2.3±0.1 μM
**22**	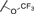	0.03±0.01 μM (3.2±0.1-fold)	1.9±0.1 μM
**23**	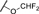	0.40±0.02 μM (6.9±0.2-fold)	1.47±0.06 μM
**24**	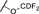	0.060±0.007 μM (4.7±0.1-fold)	1.3±0.3 μM
**25**	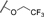	0.06±0.03 μM (3.4±0.2-fold)	0.40±0.03 μM
**26**	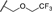	0.17±0.03 μM (6.2±0.2-fold)	1.7±0.1 μM
**27**	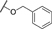	0.04±0.02 μM (2.6±0.2-fold)	1.22±0.09 μM
**28**	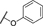	0.10±0.03 μM (2.4±0.3-fold)	2.96±0.03 μM
**29**	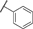	0.11±0.02 μM (2.9±0.3-fold)	4.23±0.09 μM
**30**	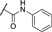	inactive (0.003-3 μM)	5.7±0.3 μM
**31**	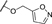	0.8±0.1 μM (5.2±0.3-fold)	4.5±0.2 μM

a Nurr1 agonism was determined in a Gal4 hybrid reporter gene assay.^32^ Data are the mean±SD, n≥3.

b hDHODH inhibition was determined in a colorimetric assay on recombinant human protein.^33^ Data are the mean±SD, n=3.

c Data from ref.^22^.

**Table 3:** Variation of the anisol motif II

	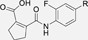		
ID	R	EC_50_(Nurr1)^a^ (max. act.)	IC_50_(hDHODH)^b^
**4**	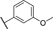	0.4±0.2 μM (3.1±0.4-fold)	0.61±0.07 μM
**32**	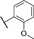	0.14±0.01 μM (4.1±0.1-fold)	0.58±0.04 μM
**33**	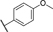	0.63±0.06 μM (6.2±0.3-fold)	4.5±0.3 μM
**22**	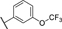	0.03±0.01 μM (3.2±0.1-fold)	1.9±0.1 μM
**34**	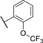	0.099±0.007 μM (2.7±0.2-fold)	1.8±0.1 μM
**35**	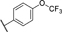	0.77±0.09 μM (4.3±0.2-fold)	>100 μM
**36**	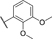	0.17±0.01 μM (4.9±0.1-fold)	0.68±0.02 μM
**37**	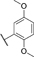	0.45±0.05 μM (3.5±0.1-fold)	1.84±0.08 μM
**38**	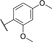	0.31±0.02 μM (4.7±0.3-fold)	1.9±0.2 μM
**39**	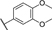	2.3±0.1 μM (5.0±0.2-fold)	3.3±0.2 μM
**40**	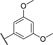	1.7±0.1 μM (4.3±0.2-fold)	6.9±0.6 μM
**41**	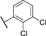	0.011±0.002 μM (2.1±0.1-fold)	0.68±0.07 μM
**42**	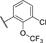	0.020±0.002 μM (2.1±0.1-fold)	0.9±0.2 μM
**43**	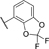	0.10±0.01 μM (5.0±0.4-fold)	1.70±0.03 μM

a Nurr1 agonism was determined in a Gal4 hybrid reporter gene assay.^32^ Data are the mean±SD, n≥3.

b hDHODH inhibition was determined in a colorimetric assay on recombinant human protein.^33^ Data are the mean±SD, n=3.

**Table 4:** Modification of the central amidobenzene

	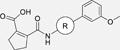		
ID	R	EC_50_(Nurr1)^a^ (max. act.)	IC_50_(hDHODH)^b^
**4**	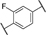	0.4±0.2 μM (3.1±0.4-fold)	0.61±0.07 μM
**11**	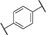	0.28±0.04 μM (3.0±0.2-fold)	0.72±0.02 μM
**44**	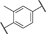	0.13±0.03 μM (2.1±0.1-fold)	0.61±0.06 μM
**45**	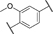	0.071±0.009 μM (2.11±0.09-fold)	0.5±0.1 μM
**46**	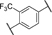	0.26±0.06 μM (2.2±0.1-fold)	2.6±0.1 μM
**47**	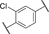	0.06±0.02 μM (2.2±0.1-fold)	0.93±0.04 μM
**48**	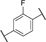	0.055±0.005 μM (2.2±0.1-fold)	0.27±0.02 μM
**49**	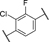	0.11±0.05 μM (1.9±0.2-fold)	2.2±0.2 μM
**50**	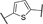	2.1±0.1 μM (7.2±0.4-fold)	>100 μM

a Nurr1 agonism was determined in a Gal4 hybrid reporter gene assay.^32^ Data are the mean±SD, n≥3.

b hDHODH inhibition was determined in a colorimetric assay on recombinant human protein.^33^ Data are the mean±SD, n=3.

**Table 5:** Combination of favored modifications

ID	structure	EC_50_(Nurr1)^a^ (max. act.)	IC_50_(hDHODH)^b^
**51**	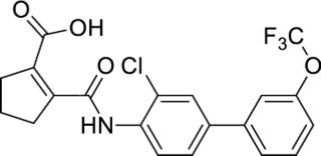	0.12±0.02 μM (2.2±0.2-fold)	5.3±0.7 μM
**52**	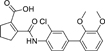	0.11±0.01 μM (3.3±0.1-fold)	1.7±0.1 μM
**53**	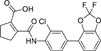	0.092±0.008 μM (4.4±0.3-fold)	4.3±0.4 μM

a Nurr1 agonism was determined in a Gal4 hybrid reporter gene assay.^32^ Data are the mean±SD, n≥3.

b hDHODH inhibition was determined in a colorimetric assay on recombinant human protein.^33^ Data are the mean±SD, n=3.

**Table 6:** Profiles of the NR4A agonist 53 and the negative reference 54.

	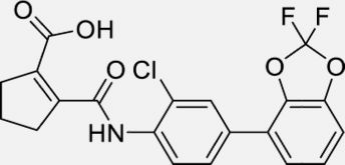	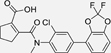
	53	54
EC_50_(Nur77) (max. act.) ^a^	0.098±0.007 μM (5.6±0.3-fold)	inactive (0.001-100 μM)
EC_50_(Nurr1) (max. act.) ^a^	0.092±0.008 μM (4.4±0.3-fold)	inactive (0.001-100 μM)
EC_50_(NOR-1) (max. act.) ^a^	0.09±0.02 μM (4.4±0.4-fold)	inactive (0.001-100 μM)
EC_50_(NBRE) (max. act.) ^b^	0.094±0.003 μM (4.8±0.2-fold)	inactive (0.001-100 μM)
EC_50_(NurRE) (max. act.) ^b^	0.099±0.005 μM (5.2±0.3-fold)	inactive (0.001-100 μM)
EC_50_(DR5) (max. act.) ^b^	0.098±0.003 μM (8.6±0.4-fold)	inactive (0.001-100 μM)
K_d_(NR4A1) ^c^	0.10±0.01 μM	n.d.
IC_50_(DHODH) ^d^	4.3±0.4 μM	inactive (100 μM)
cytotoxicity ^e^	>10 μM	>10 μM
aq. solubility ^f^	23±7 μM (10±3 mg/L)	69±2 μM (30.1±0.9 mg/L)

a NR4A agonism was determined in a Gal4 hybrid reporter gene assay.^32^ Data are the mean±SD, n≥3.

b NR4A RE activation was determined using reporters with a single repeat of the respective RE. Nurr1 and RXRα (only for DR5) were overexpressed. Data are the mean±SD, n≥3.

c From isothermal titration calorimetry (ITC; Figure S1; mean±SD, n=2; n.d. - not determined).

d hDHODH inhibition was determined in a colorimetric assay on recombinant human protein.^33^ Data are the mean±SD, n=3.

e Cytotoxicity was evaluated in a multiplex assay monitoring confluence, metabolic activity and necrosis in HEK293T cells (Figure 3b).

f Thermodynamic aq. solubility. The higher solubility of **54** compared to **53** may be due to steric repulsion between the chloro substituent and the *N*-methyl group potentially leading to altered crystal packing and lower lattice energy.^34^

## Abbreviations

BDNF: brain-derived neurotrophic factor
CCND2: cyclin D2
CNS: central nervous system
CRMP4: collapsing response mediator protein 4
DHODH: dihydroorotate dehydrogenase
FLRT2: fibronectin leucine rich transmembrane protein 2
HTRF: homogeneous time-resolved fluorescence resonance energy transfer
LBD: ligand binding domain
NOR1: neuron derived orphan receptor 1
NRP-1: neuropilin-1
Nur77: nerve growth factor IB
Nurr1: nuclear receptor related 1
RE: response element
RXR: retinoid X receptor
SAR: structure activity relationship
Sesn3: sestrin 3
SOD2: superoxide dismutase 2
TH: tyrosine hydroxylase
XIAP: X-linked inhibitor of apoptosis protein
