# Supplementary material for: Structural Tuning of Vidofludimus for High-Efficacy NR4A Agonism
Source: J Med Chem. Author manuscript; Available in PMC 2026 Mar 5. (PMC7618816; doi:10.1021/acs.jmedchem.5c03217)
Supplement: Supporting info. [file EMS212278-supplement-Supporting_info_.pdf]

- Supporting Information -

Structural Tuning of Vidofludimus for High-Efficacy NR4A Agonism

Jan Vietor[1], Romy Busch[1], Úrsula López-García[1], Tanja Stiller[1], Anna Maria Thommes[1], Christian Gege[2], Daniel Merk[1]*

[1] Ludwig-Maximilians-Universität München, Department of Pharmacy, 81377 Munich, Germany; [2] Immunic AG, 82166 Gräfelfing, Germany; * daniel.merk@cup.lmu.de

Table of Contents

Supplementary Figures & Tables S2

NMR spectra of **8-13** and **16-53** S3

Supplementary References S71

Supplementary Figures & Tables

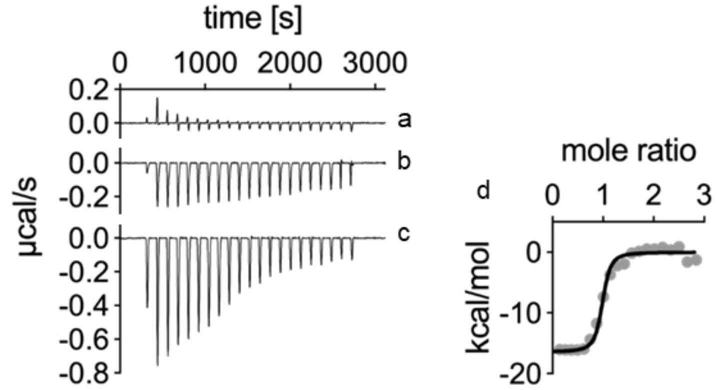

Figure S1. Isothermal titration calorimetry showing binding of **53** to the recombinant NR4A1 LBD. Representative buffer-protein (a), compound-buffer (b) and compound-protein (c) titrations are shown. (d) Fitting of the blank corrected heat of binding of **53** (K_d 0.10±0.01 µM).

Table S1. In vitro pharmacological profiles of selected optimized NR4A agonists from the literature.

structure<br>name/ID	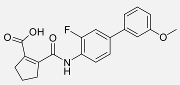<br>vidofludimus (**4**)	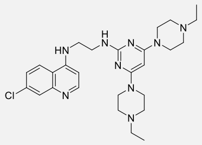<br>4A7C-301 (**5**)	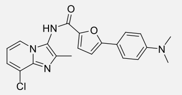<br>**6**	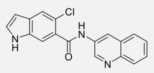<br>**7**	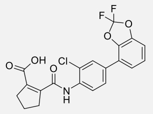<br>**53**
EC_{50}(NR4A1/2/3) [a]	3.1/0.4/2.9 µM	n.a/6.5/n.a µM	0.33/0.09/0.11 µM	0.04/0.06/0.07 µM	0.098/0.092/0.09 µM
K_d [a]	0.7 µM (NR4A2)	(0.1 µM) [b]	0.17 µM (NR4A2)	0.12 µM (NR4A2)	0.10 µM (NR4A1)
neg. ctrl compound available [c]	no	no	yes	yes	yes
fold NR4A1/2/3 activation [d] at the indicated conc.	2.4/2.8/2.2<br>(1 µM)	2.4/2.1/3.9<br>(20 µM)	1.3/2.1/1.6<br>(0.3 µM)	1.9/1.9/1.9<br>(1 µM)	5.0/4.2/4.1<br>(0.3 µM)
reference	[1]	[2]	[3]	[4]	(this study)

[a] From the original reference.
[b] IC_{50} for competition with labeled chloroquine for binding to NR4A2.
[c] According to the original reference.
[d] Data from our uniform Gal4-hybrid reporter gene assays (see also ref. [5]).

NMR spectra of **8-13** and **16-53**

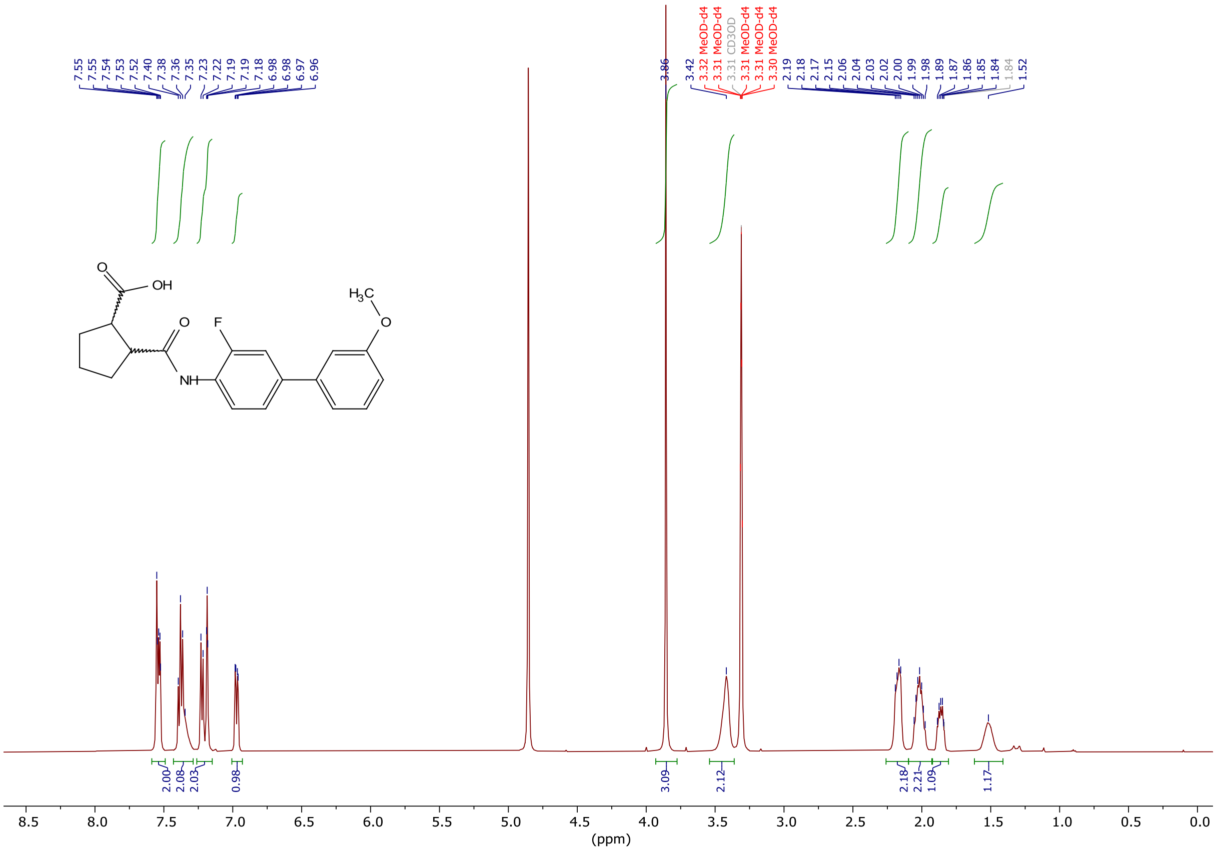

^{1}H NMR (500 MHz, MeOD-d_4) of **8**

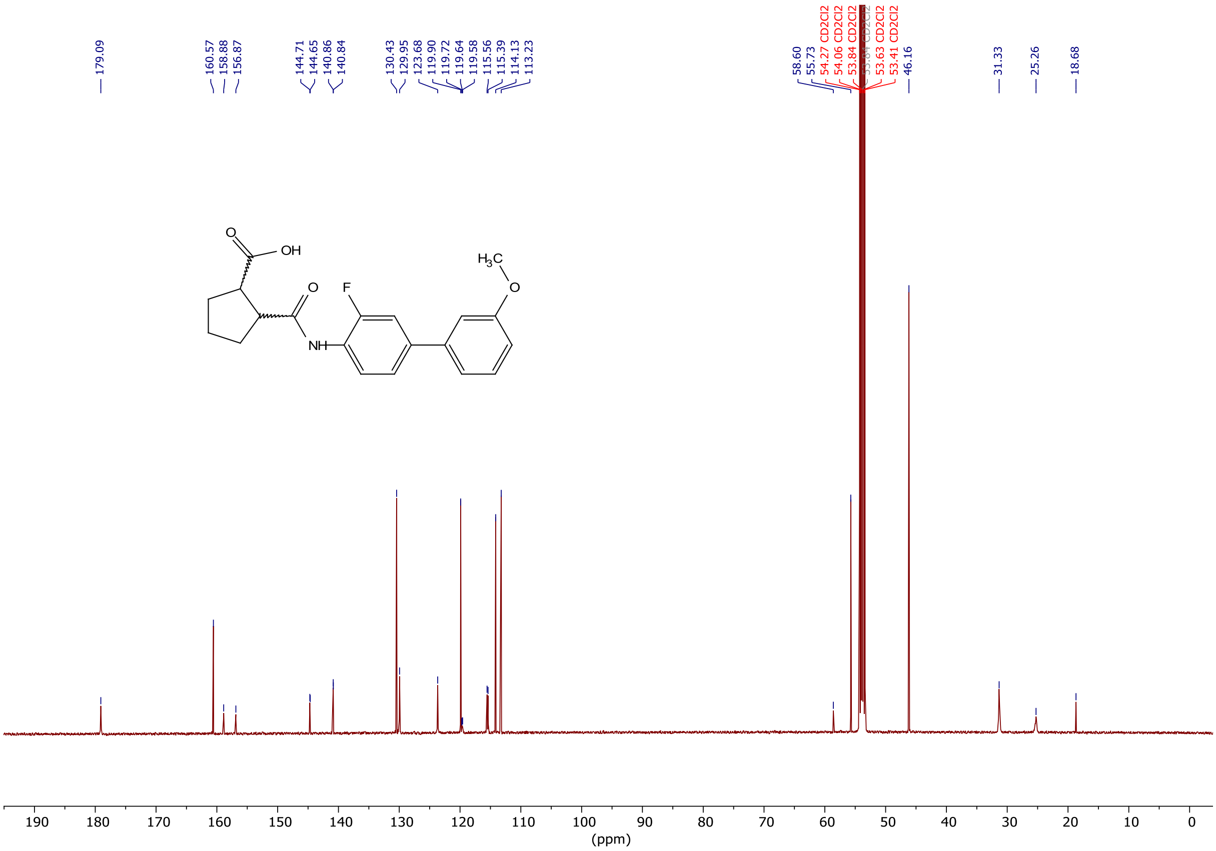

^{13}C NMR (126 MHz, CD_2Cl_2) of **8**

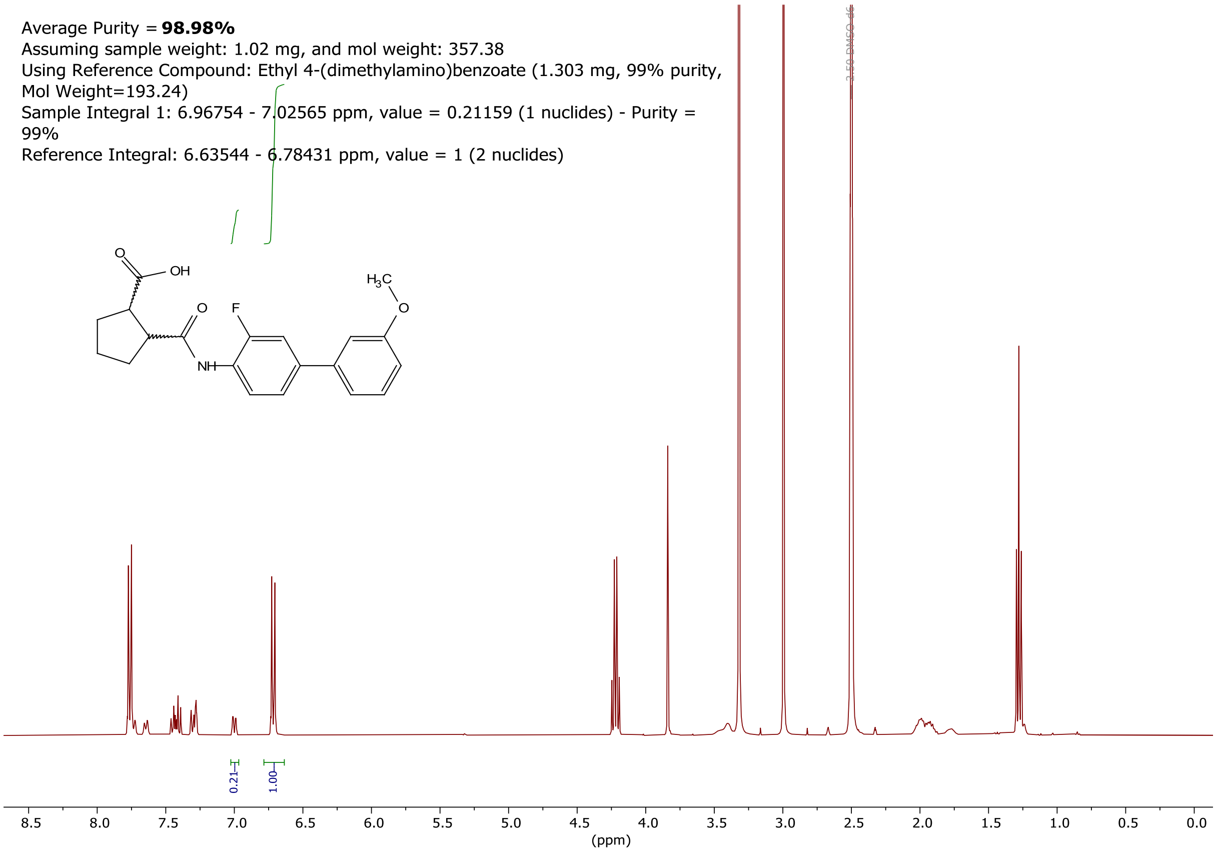

qH NMR (400 MHz, DMSO-d_6) of **8**

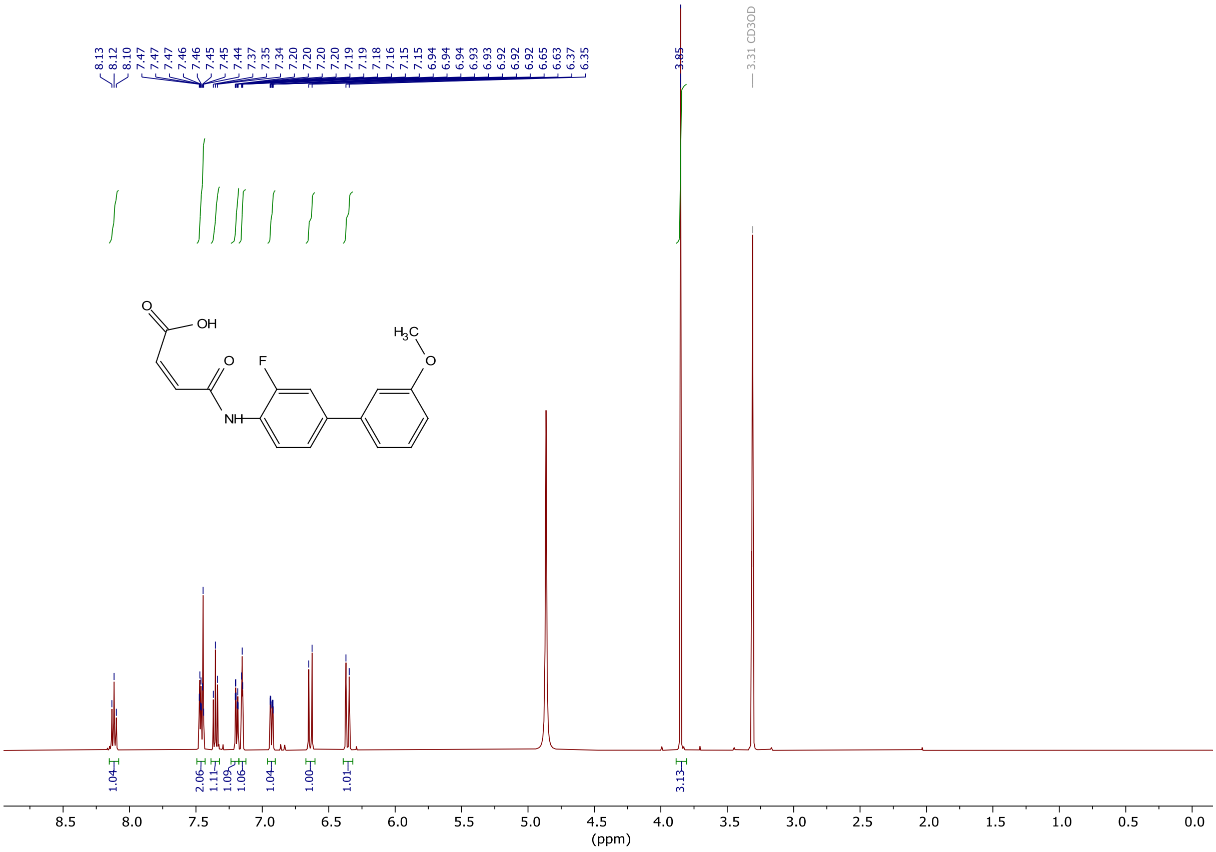

^{1}H NMR (500 MHz, MeOD-d_4) of **9**

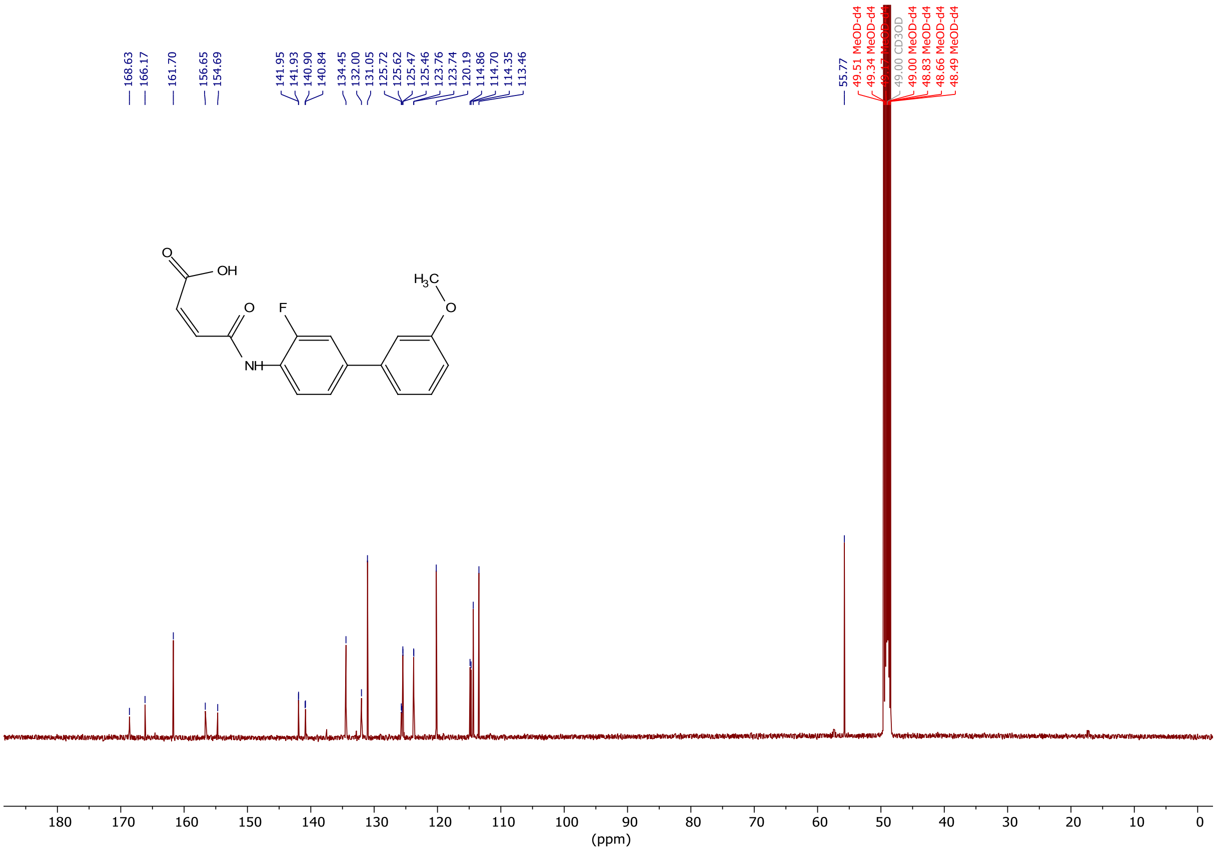

^{13}C NMR (126 MHz, MeOD-d_4) of **9**

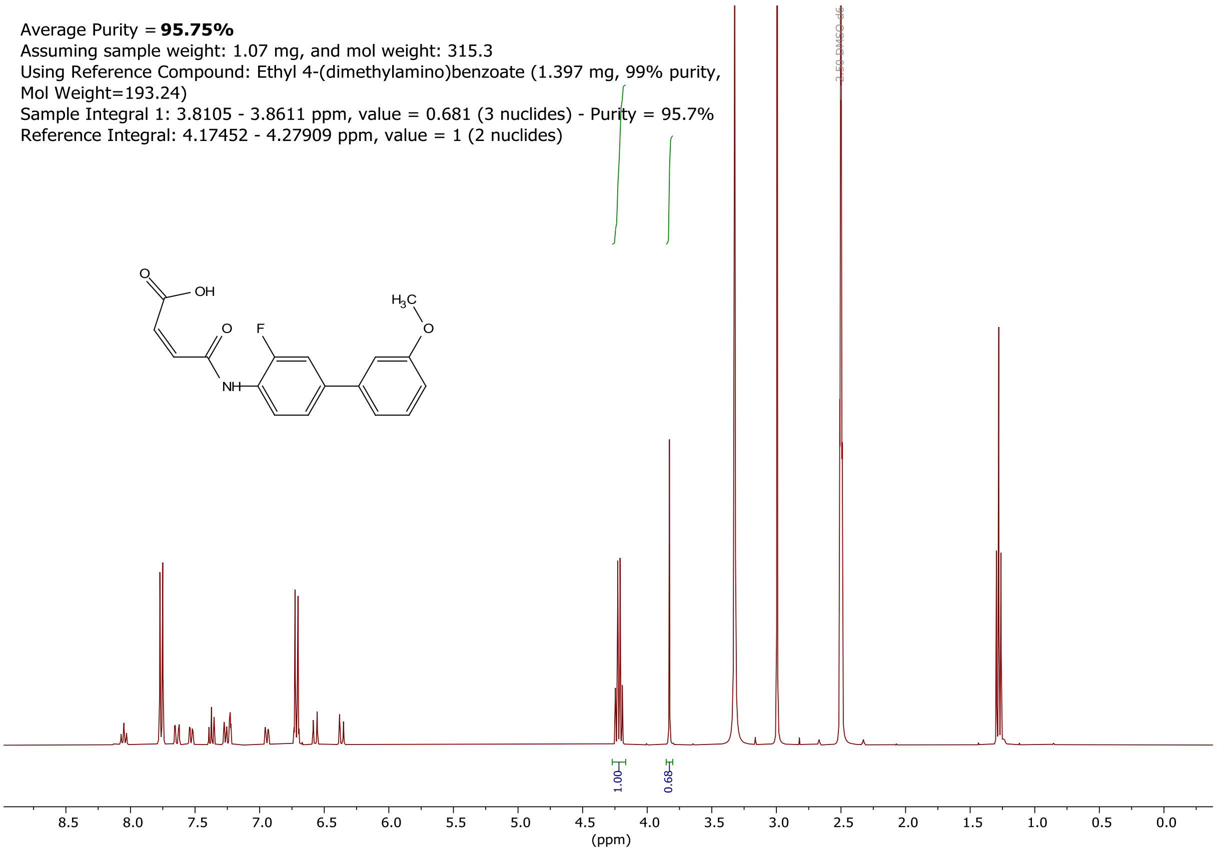

qH NMR (400 MHz, DMSO-d_6) of **9**

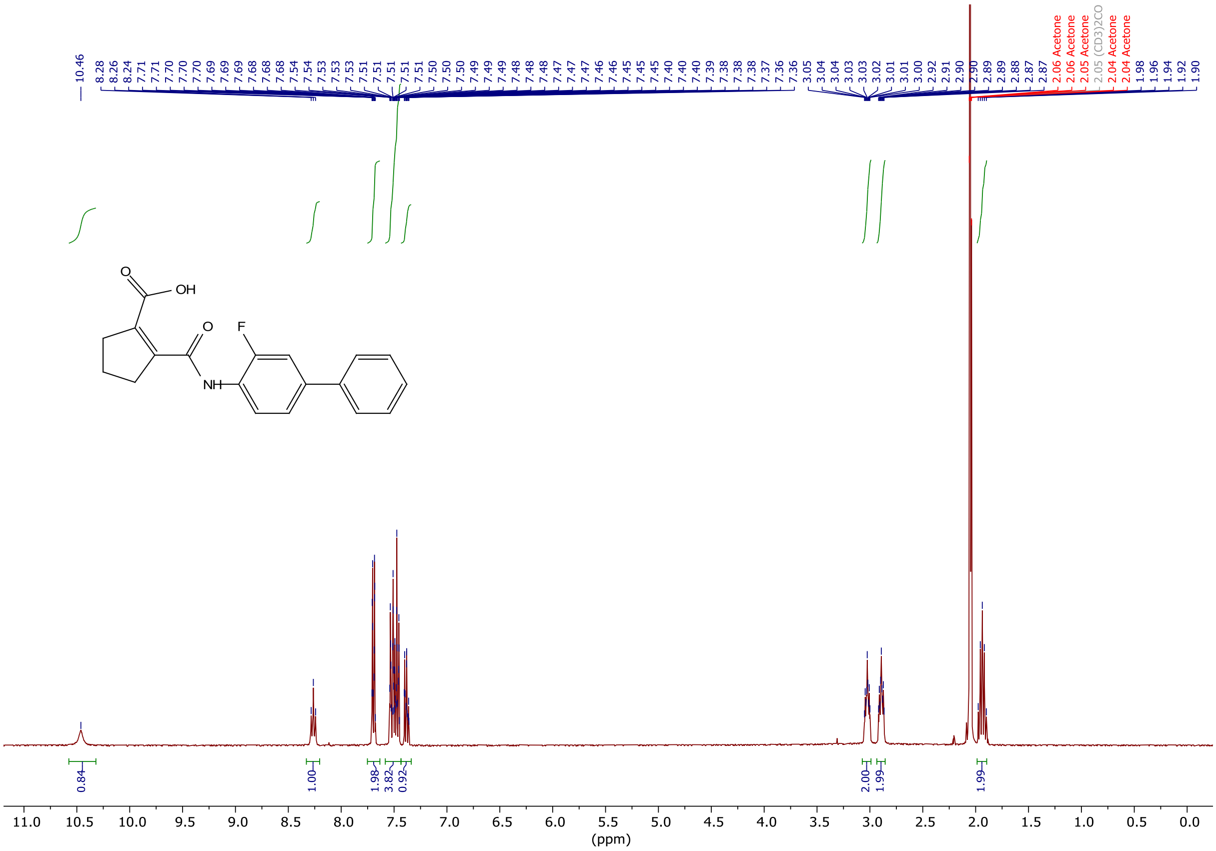

^{1}H NMR (400 MHz, acetone-d_6) of **10**

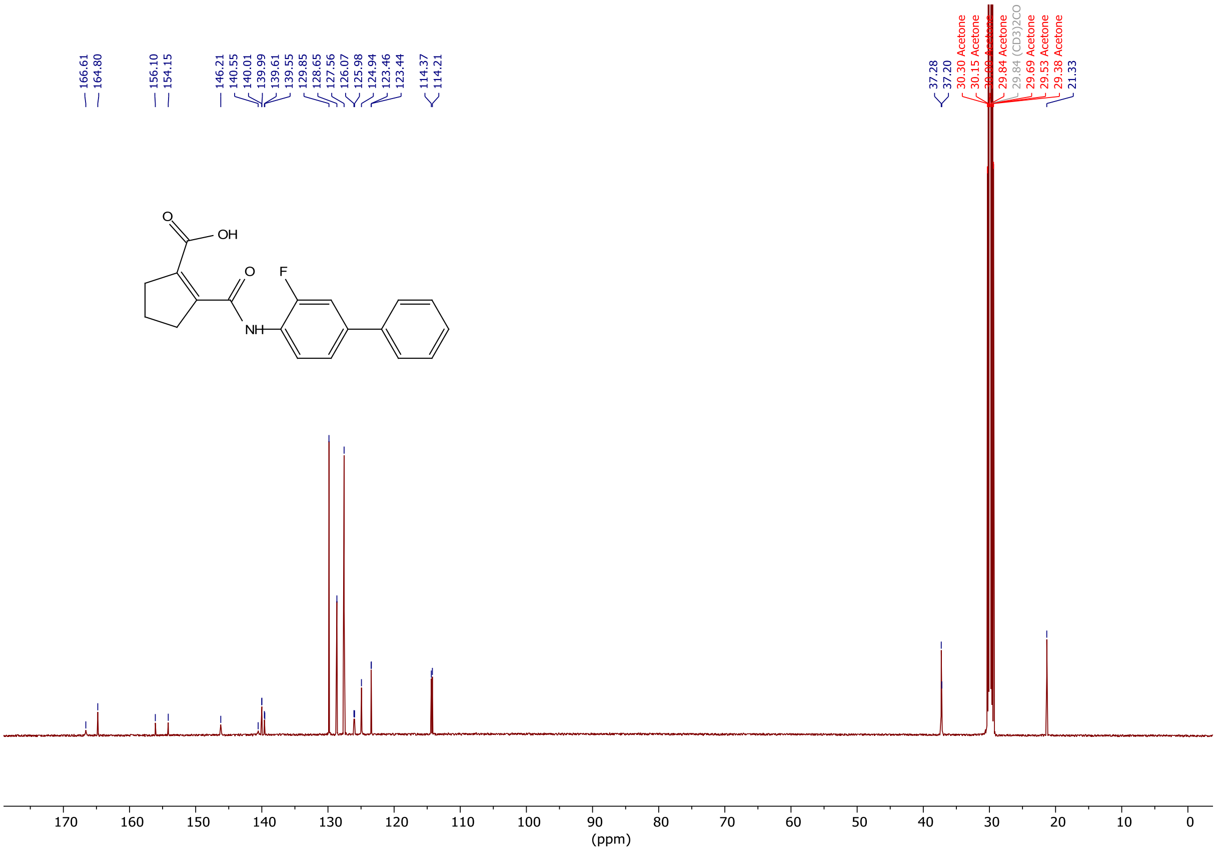

^{13}C NMR (126 MHz, acetone-d_6) of **10**

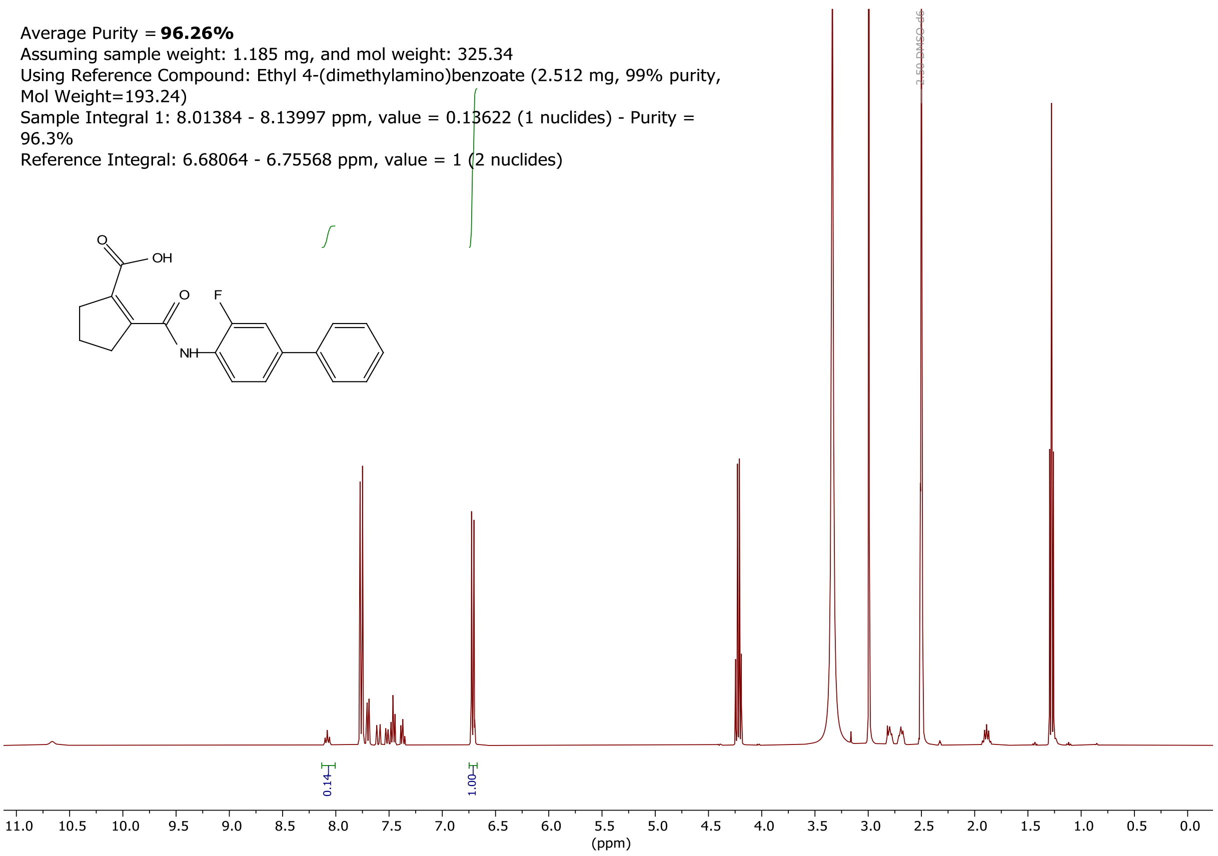

qH NMR (400 MHz, DMSO-d_6) of **10**

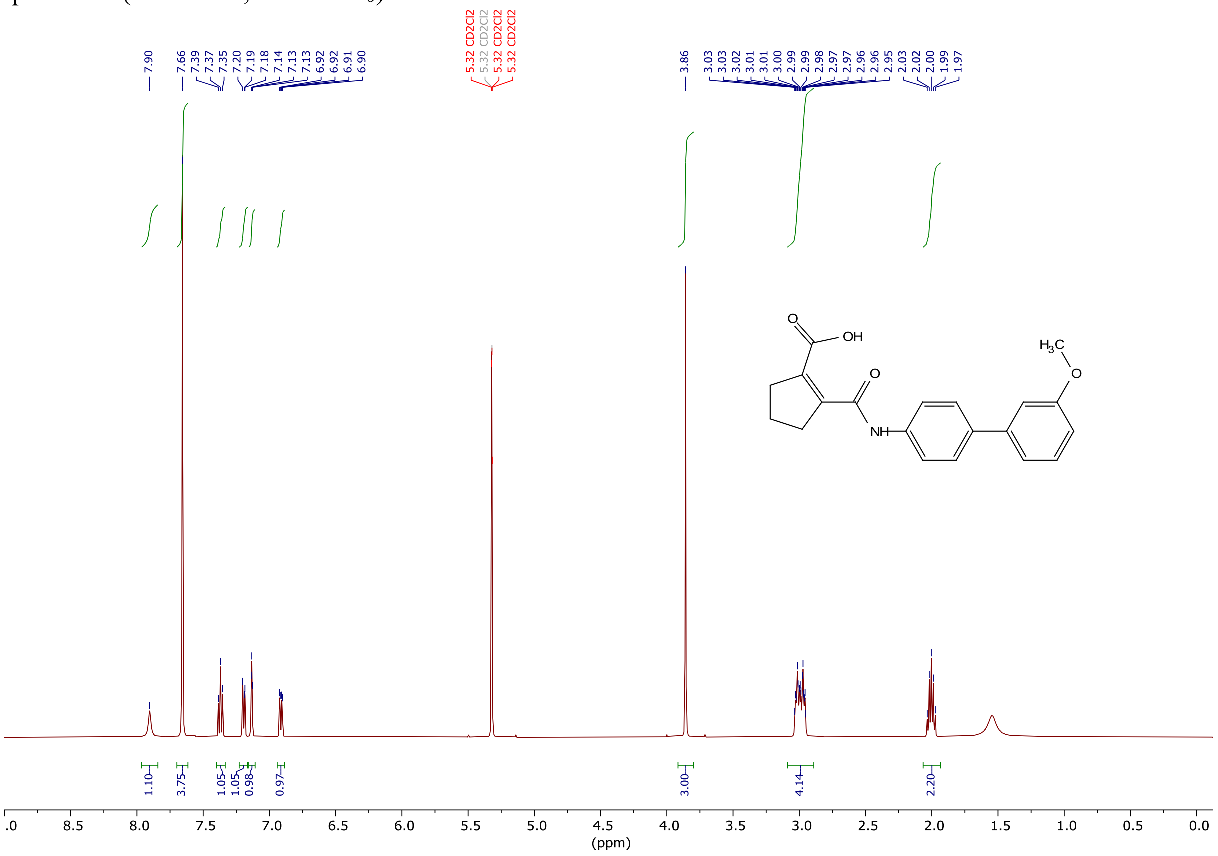

^{1}H NMR (500 MHz, CD_2Cl_2) of **11**

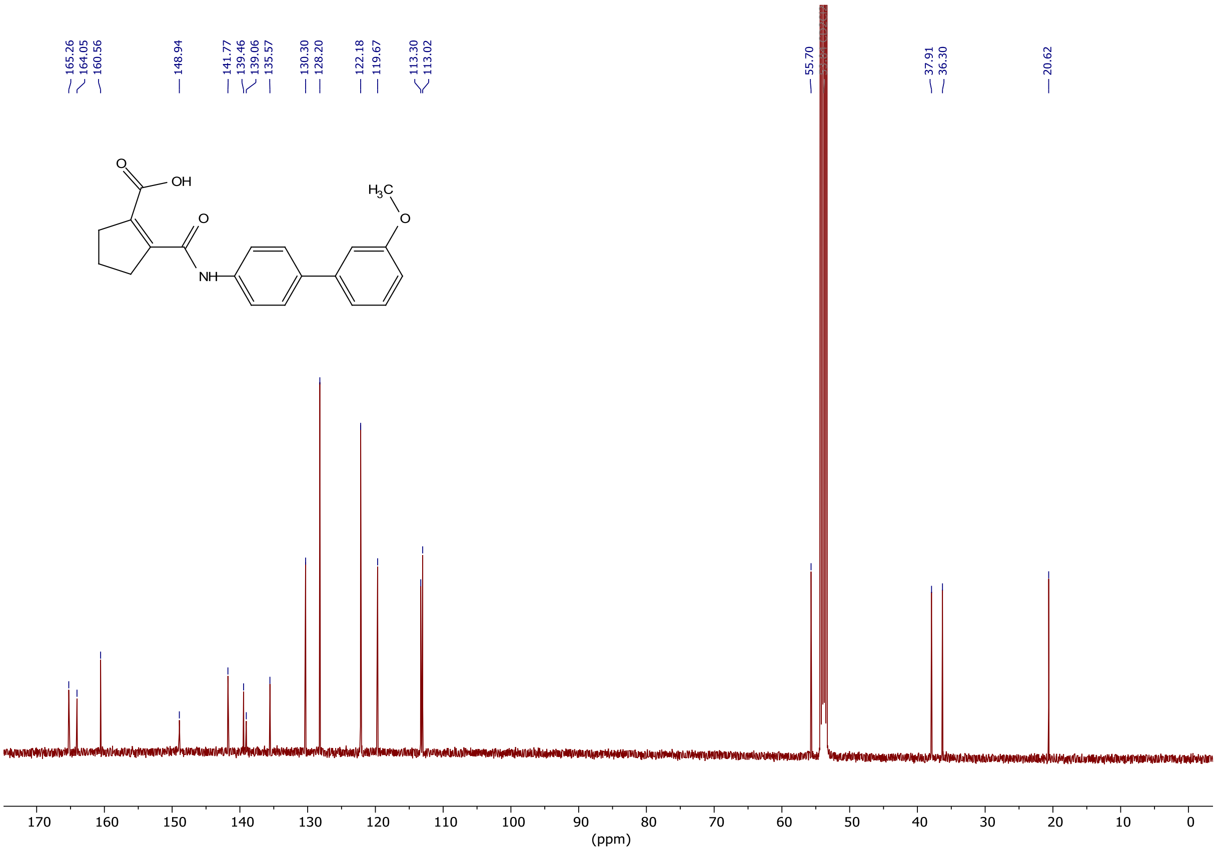

^{13}C NMR (126 MHz, CD_2Cl_2) of **11**

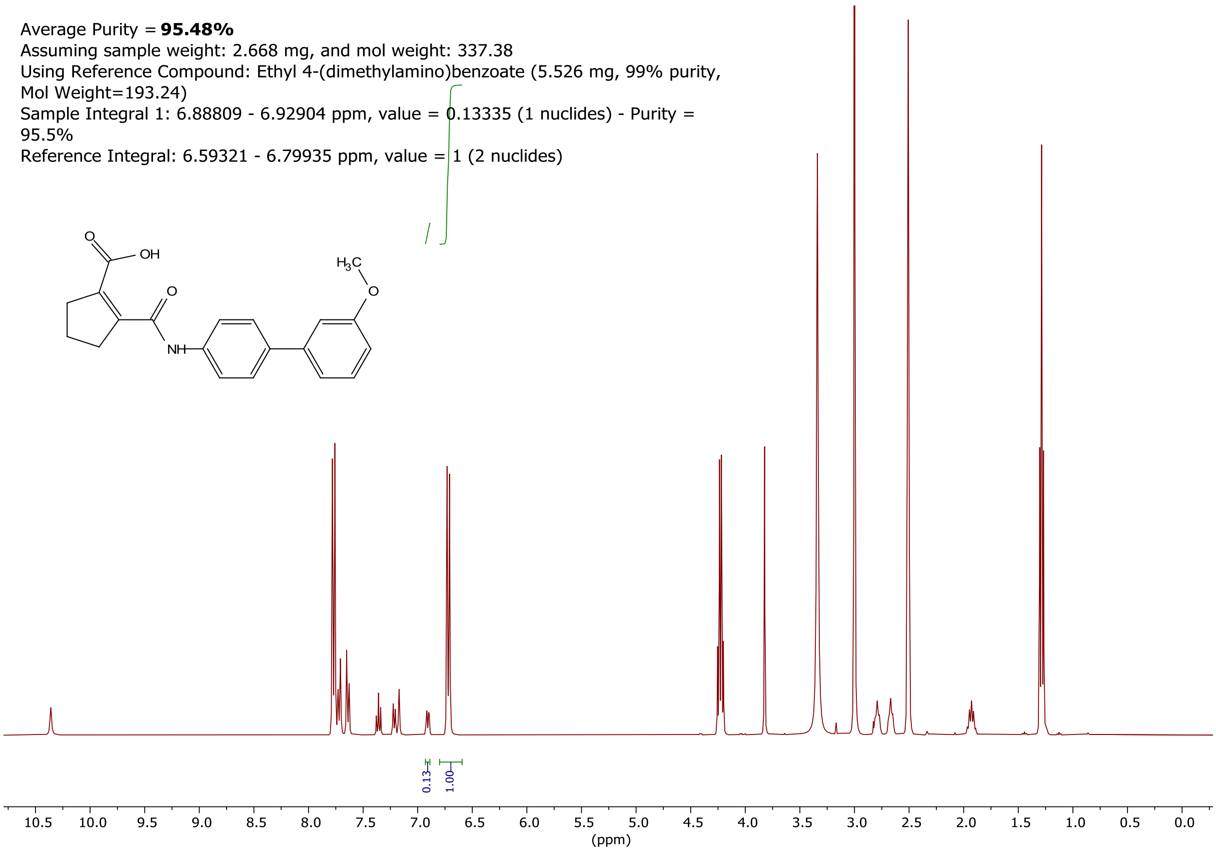

qH NMR (400 MHz, DMSO-d_6) of **11**

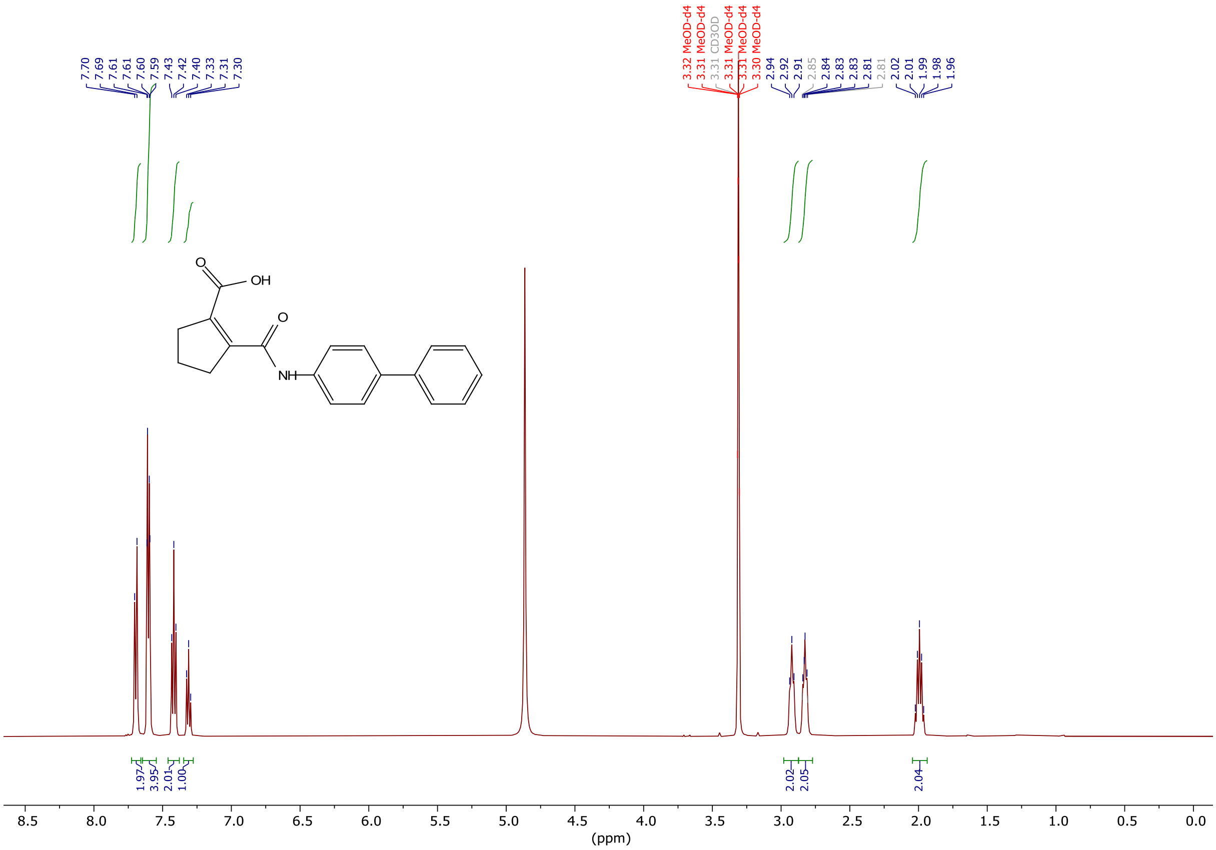

^{1}H NMR (500 MHz, MeOD-d_4) of **12**

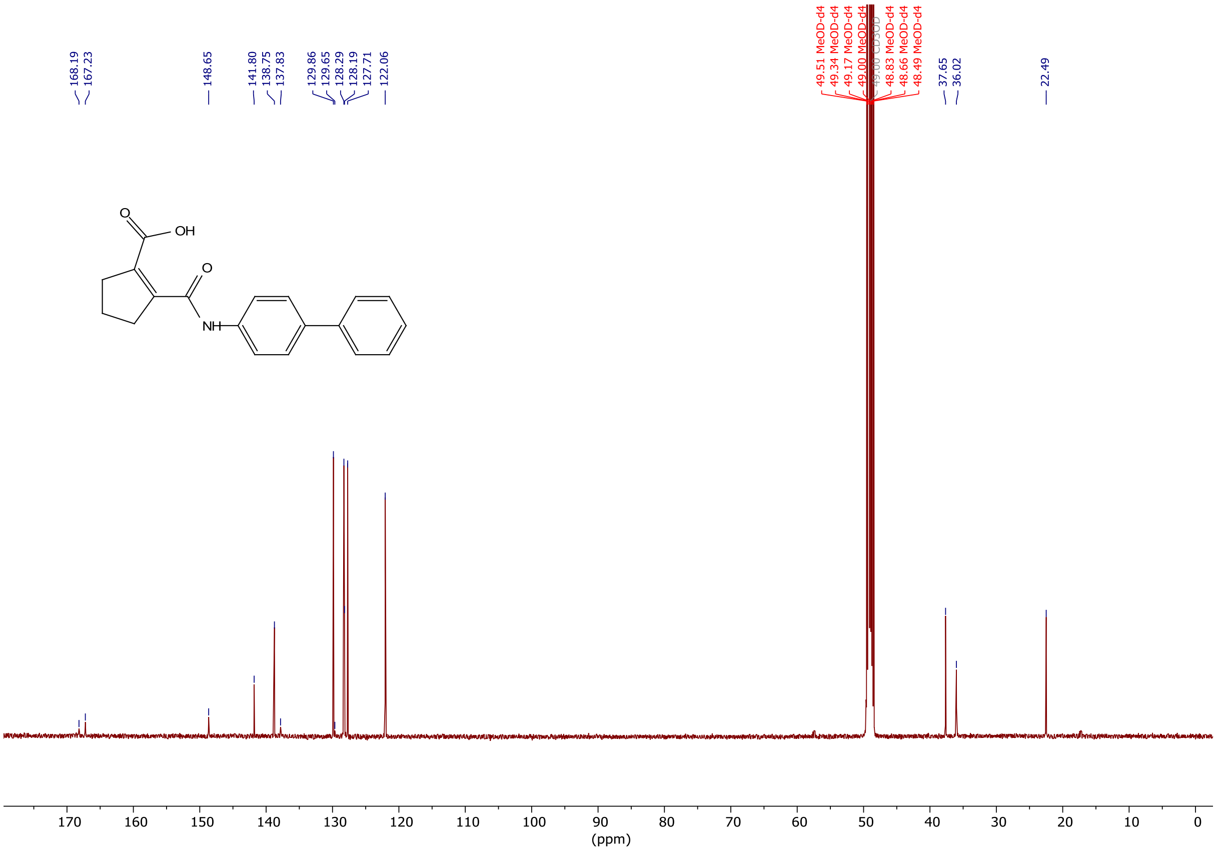

^{13}C NMR (126 MHz, MeOD-d_4) of **12**

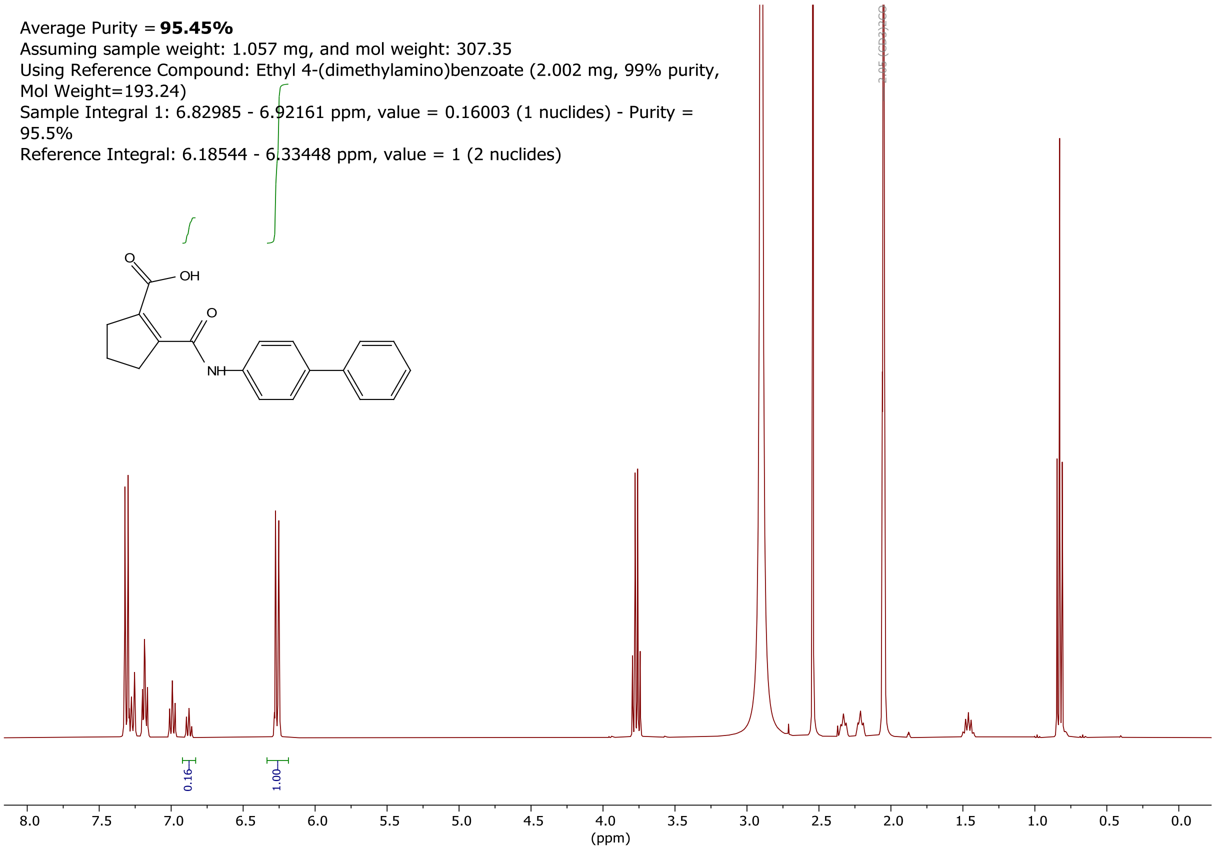

qH NMR (400 MHz, DMSO-d_6) of **12**

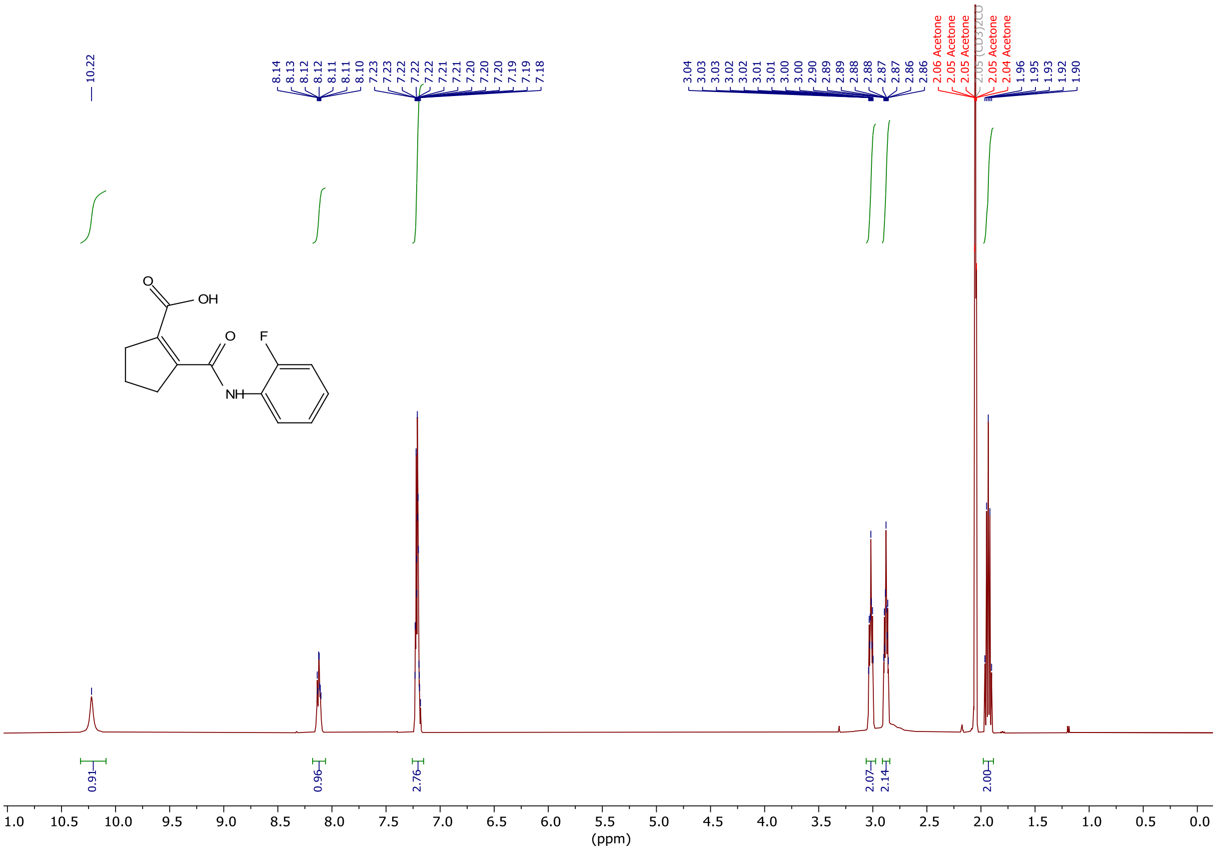

^{1}H NMR (500 MHz, acetone-d_6) of **13**

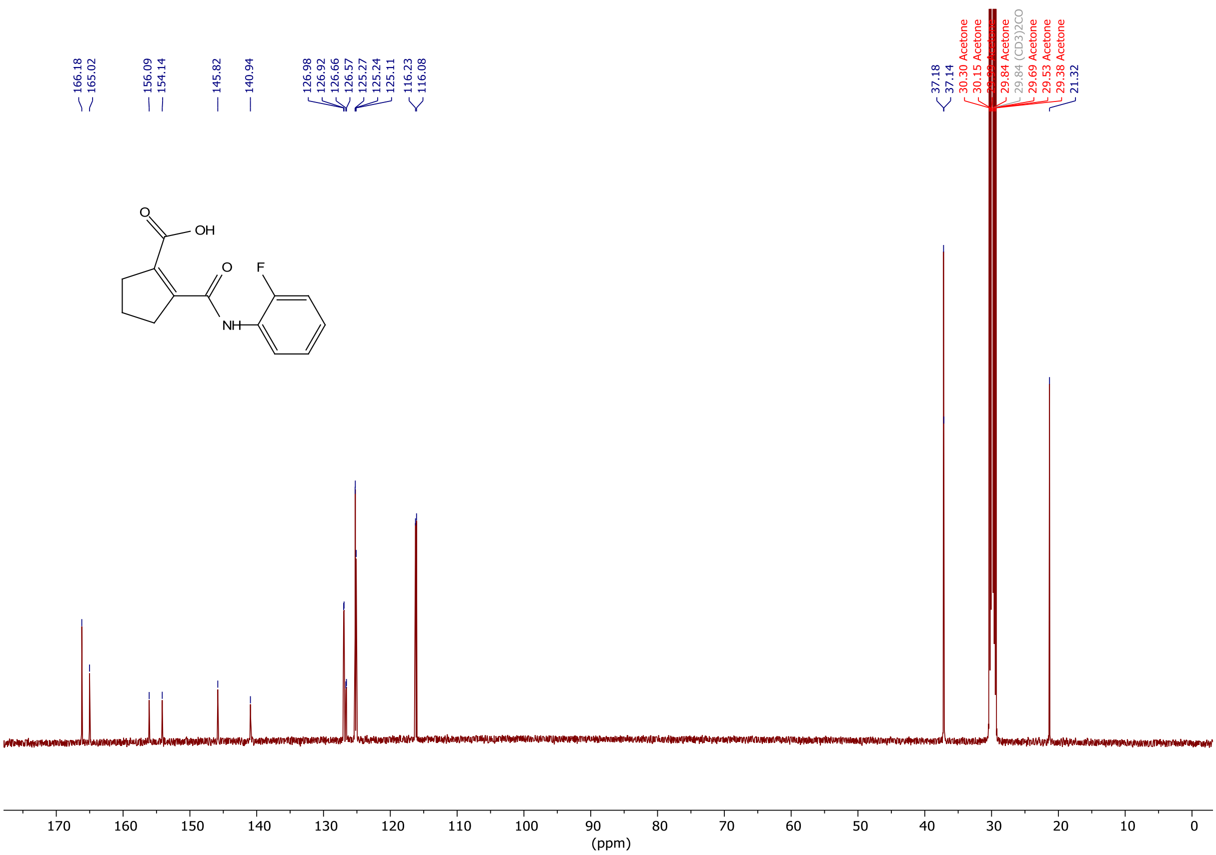

^{13}C NMR (126 MHz, acetone-d_6) of **13**

Average Purity = **96.85%**
Assuming sample weight: 1.018 mg, and mol weight: 249.24
Using Reference Compound: Ethyl 4-(dimethylamino)benzoate (2.049 mg, 99% purity, Mol Weight=193.24)
Sample Integral 1: 7.89156 - 8.00703 ppm, value = 0.99538 (1 nuclides) - Purity = 96.8%
Reference Integral: 7.70913 - 7.80191 ppm, value = 5.28307 (2 nuclides)

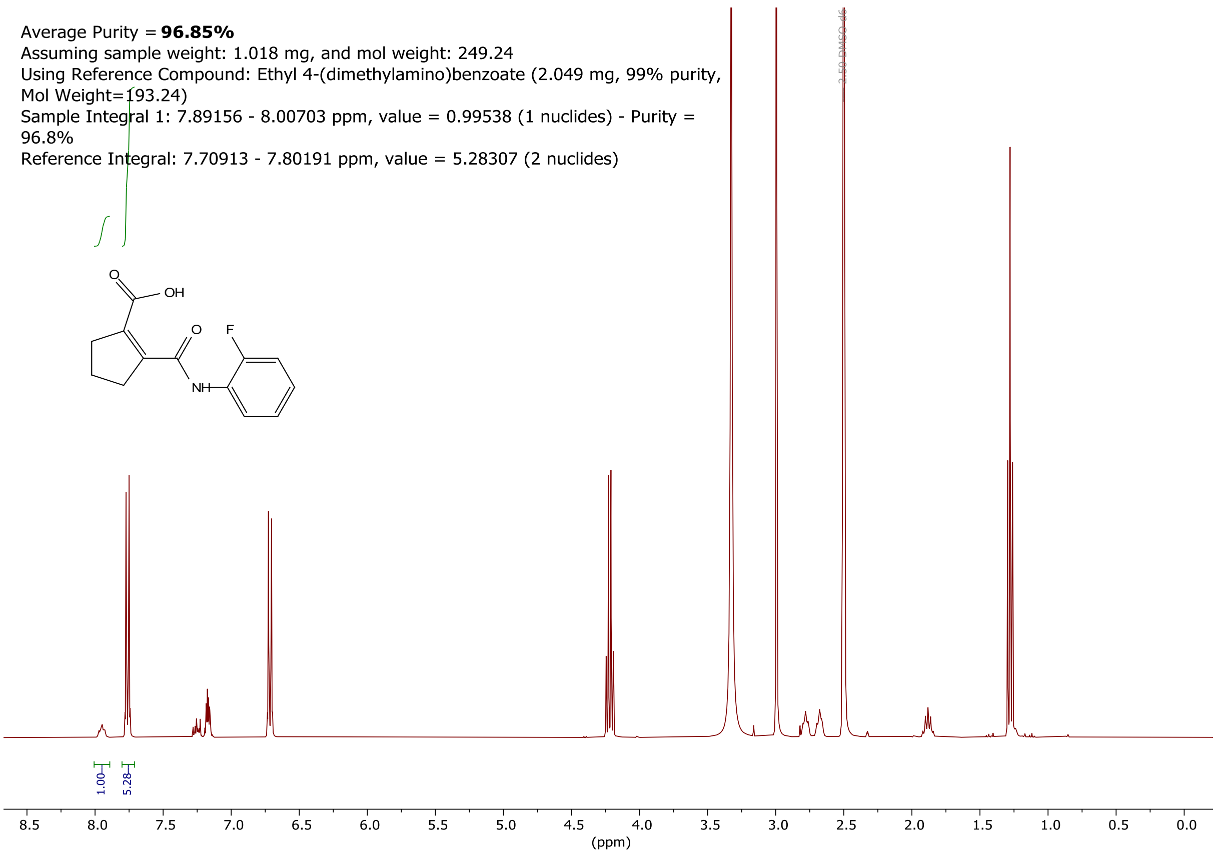

qH NMR (400 MHz, DMSO-d_6) of **13**

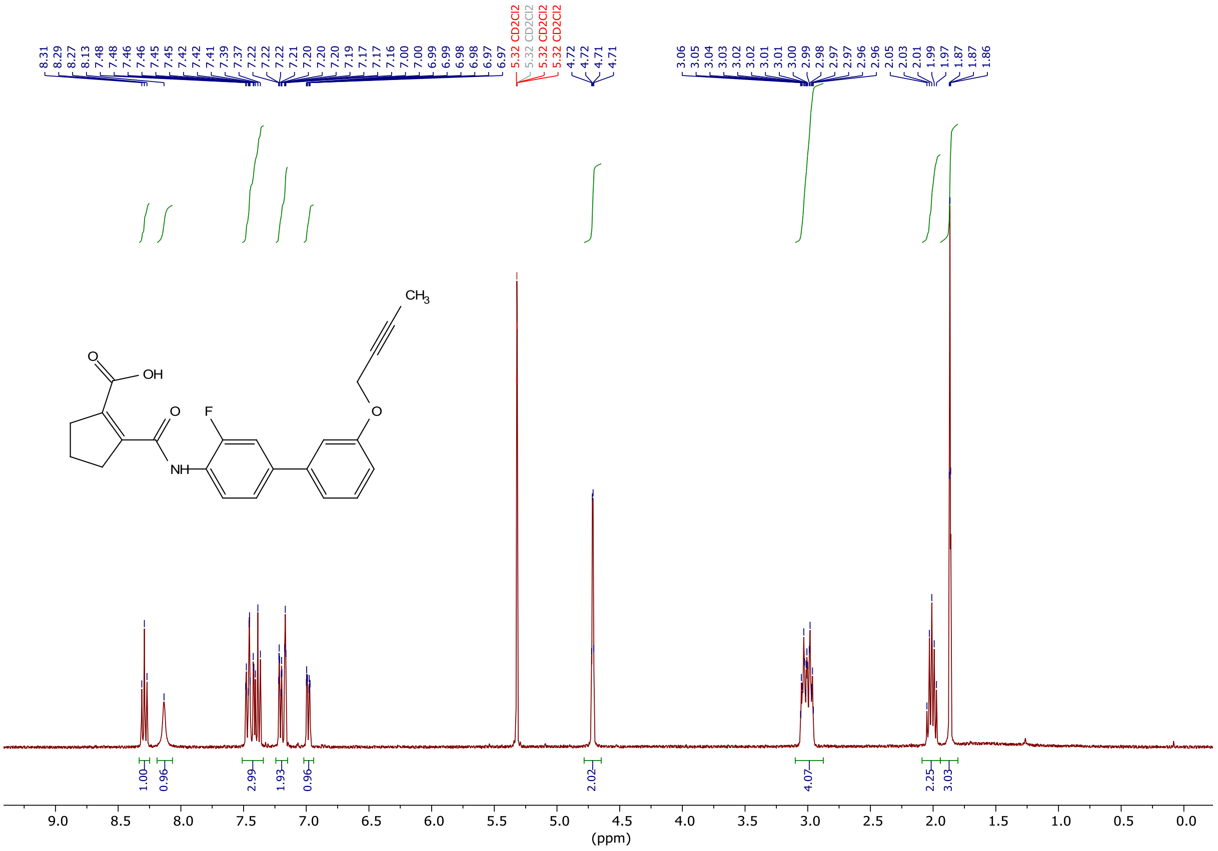

^{1}H NMR (400 MHz, CD_2Cl_2) of **16**

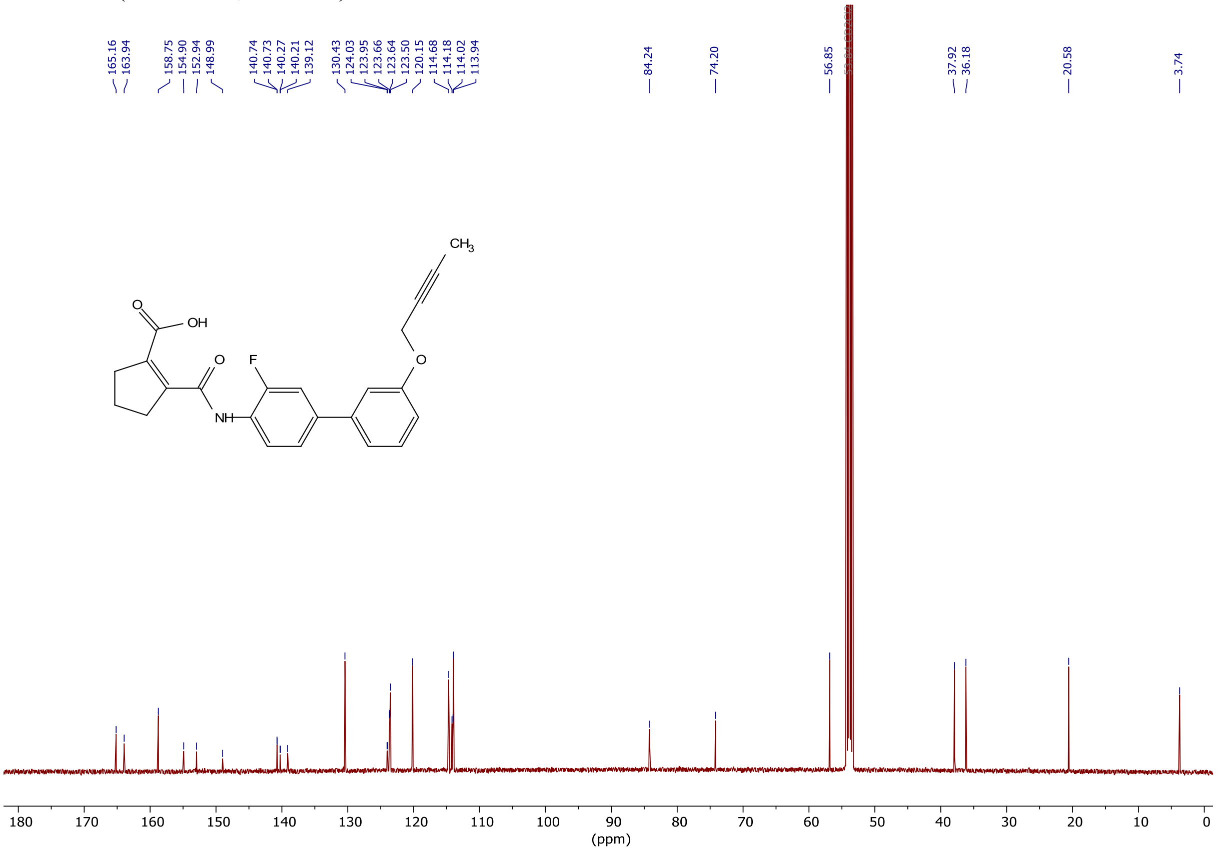

^{13}C NMR (126 MHz, CD_2Cl_2) of **16**

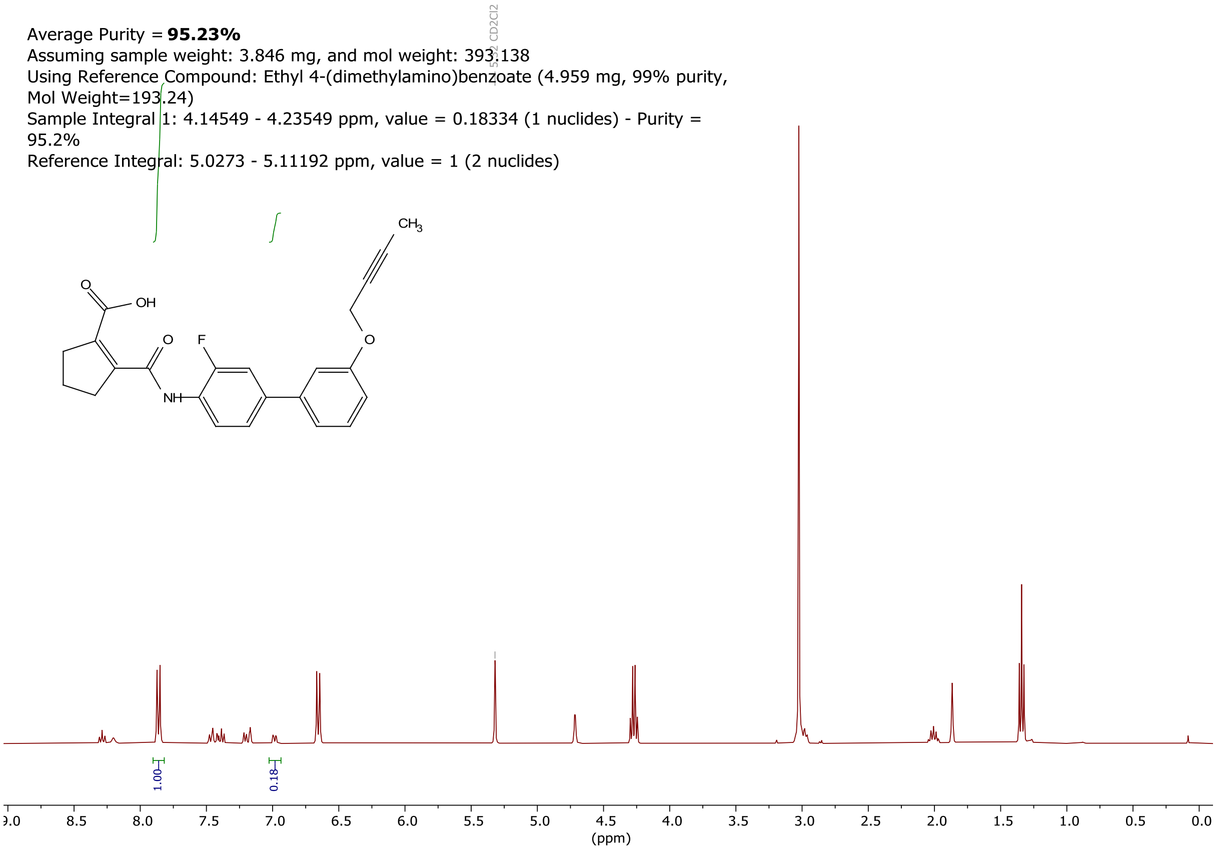

qH NMR (400 MHz, DMSO-d_6) of **16**

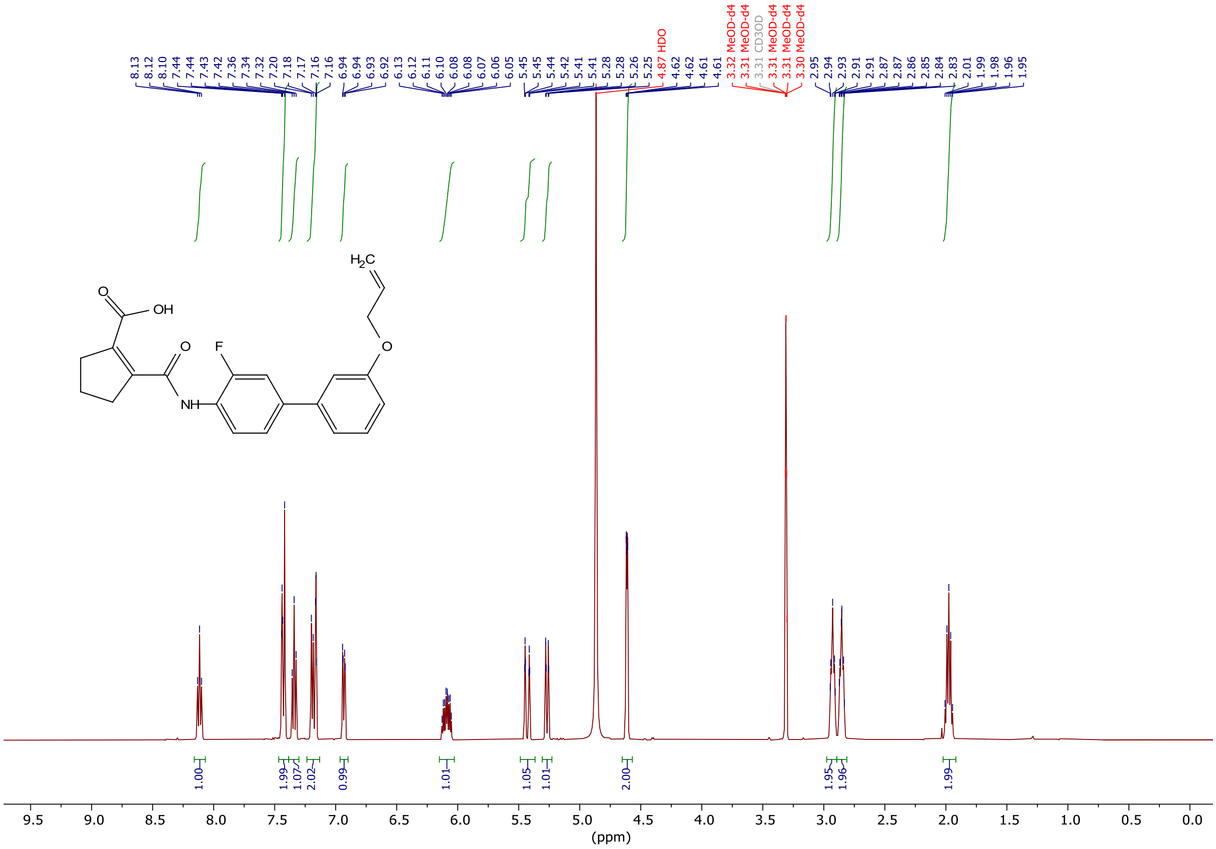

^{1}H NMR (500 MHz, MeOD-d_4) of **17**

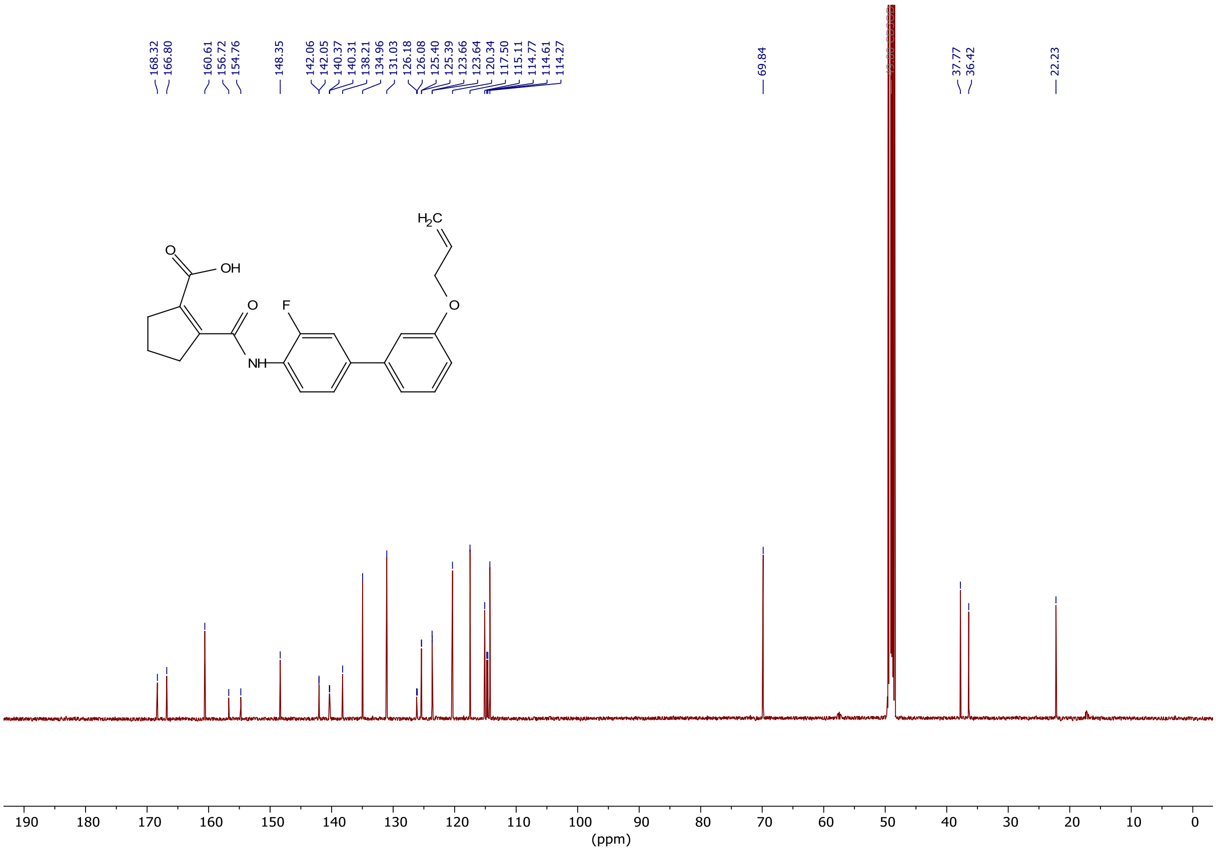

^{13}C NMR (126 MHz, acetone-d_6) of **17**

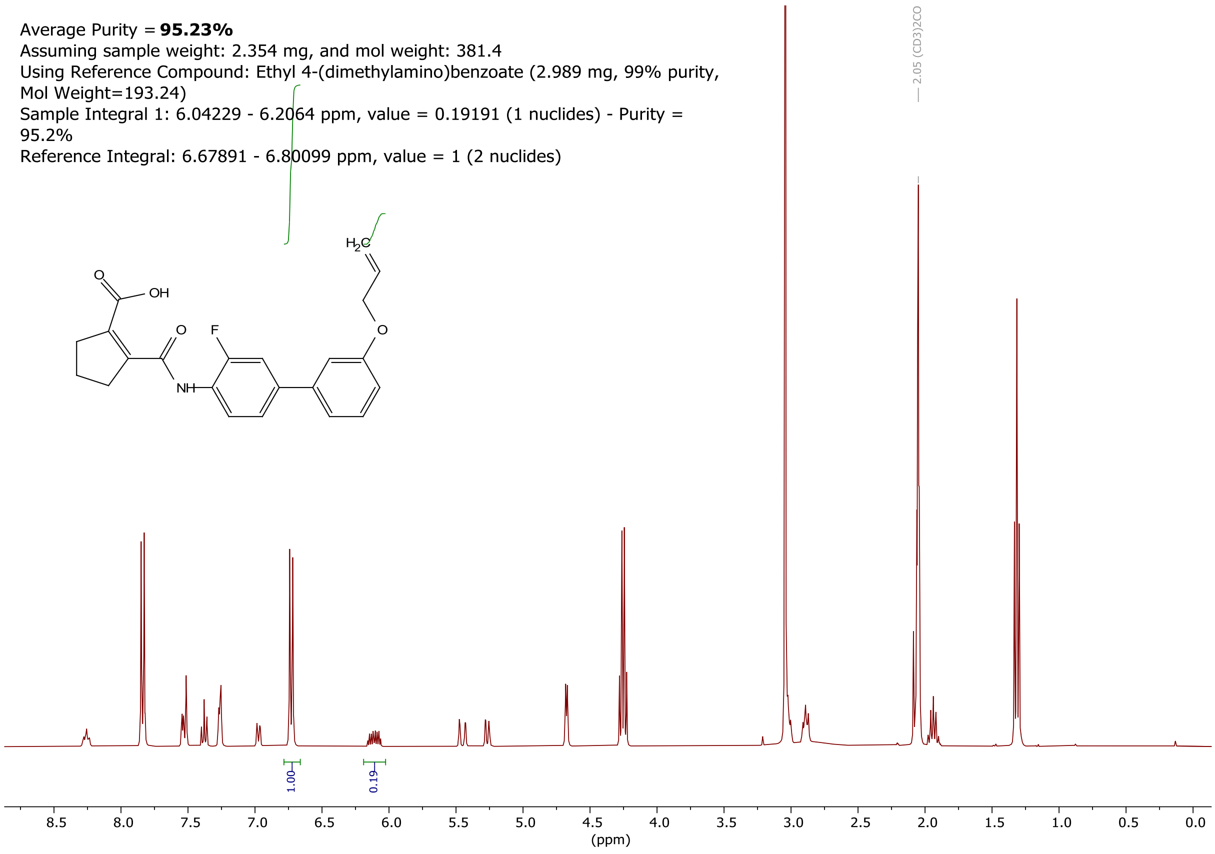

qH NMR (400 MHz, DMSO-d_6) of **17**

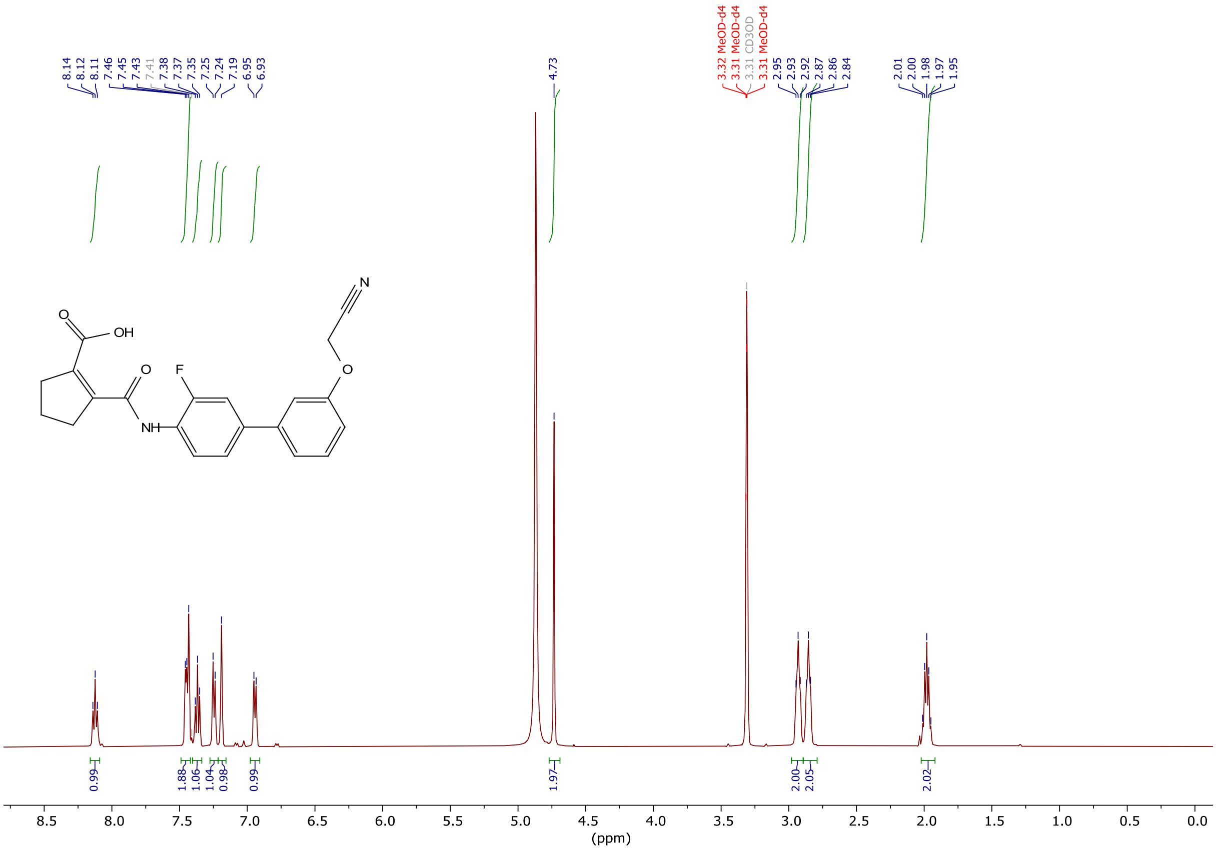

^{1}H NMR (500 MHz, MeOD-d_4) of **18**

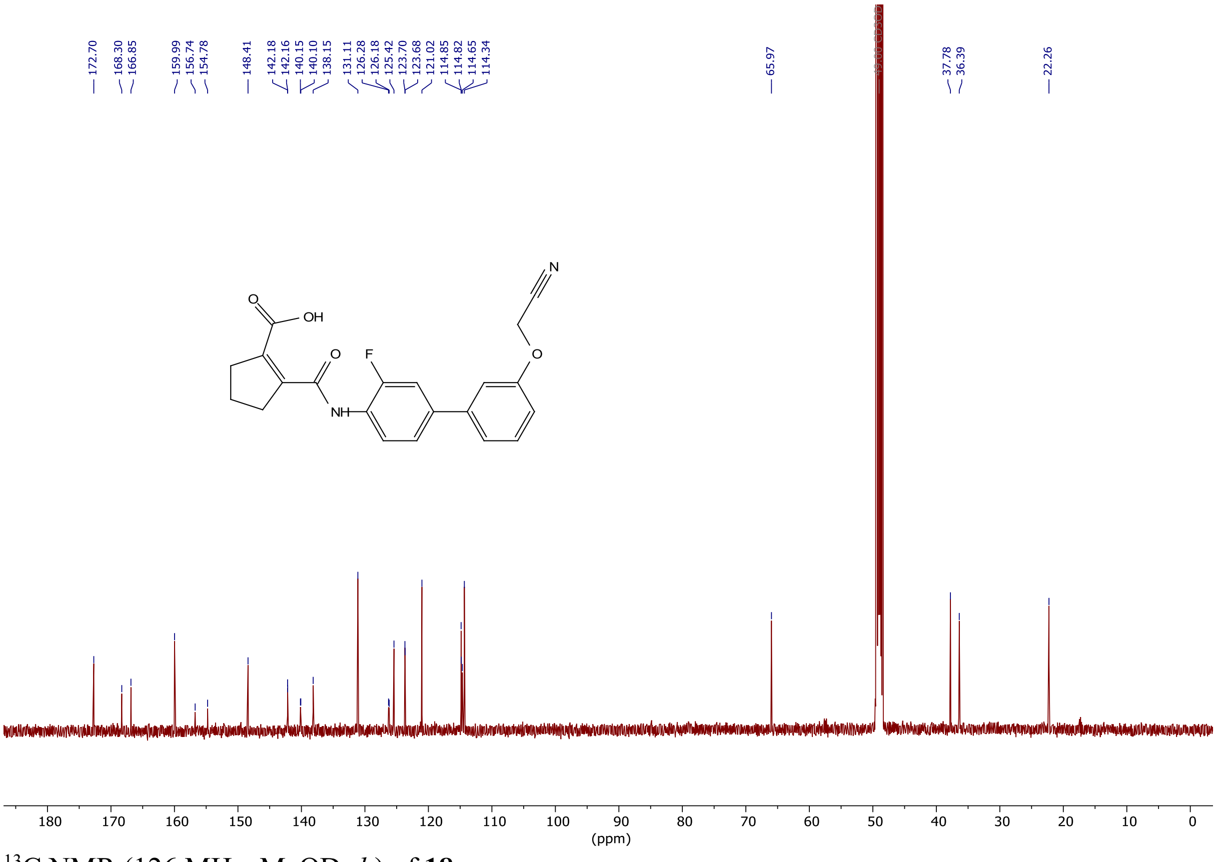

^{13}C NMR (126 MHz, MeOD-d_4) of **18**

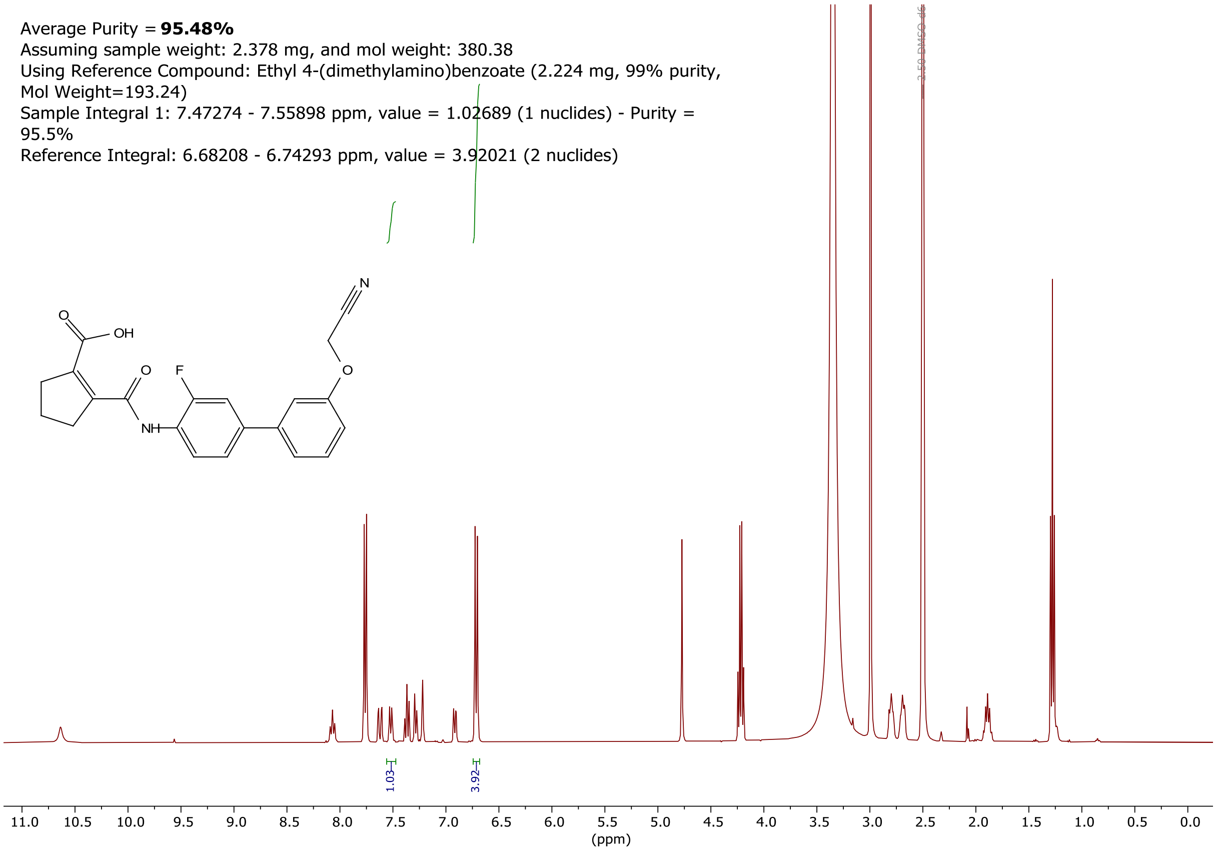

qH NMR (400 MHz, DMSO-d_6) of **18**

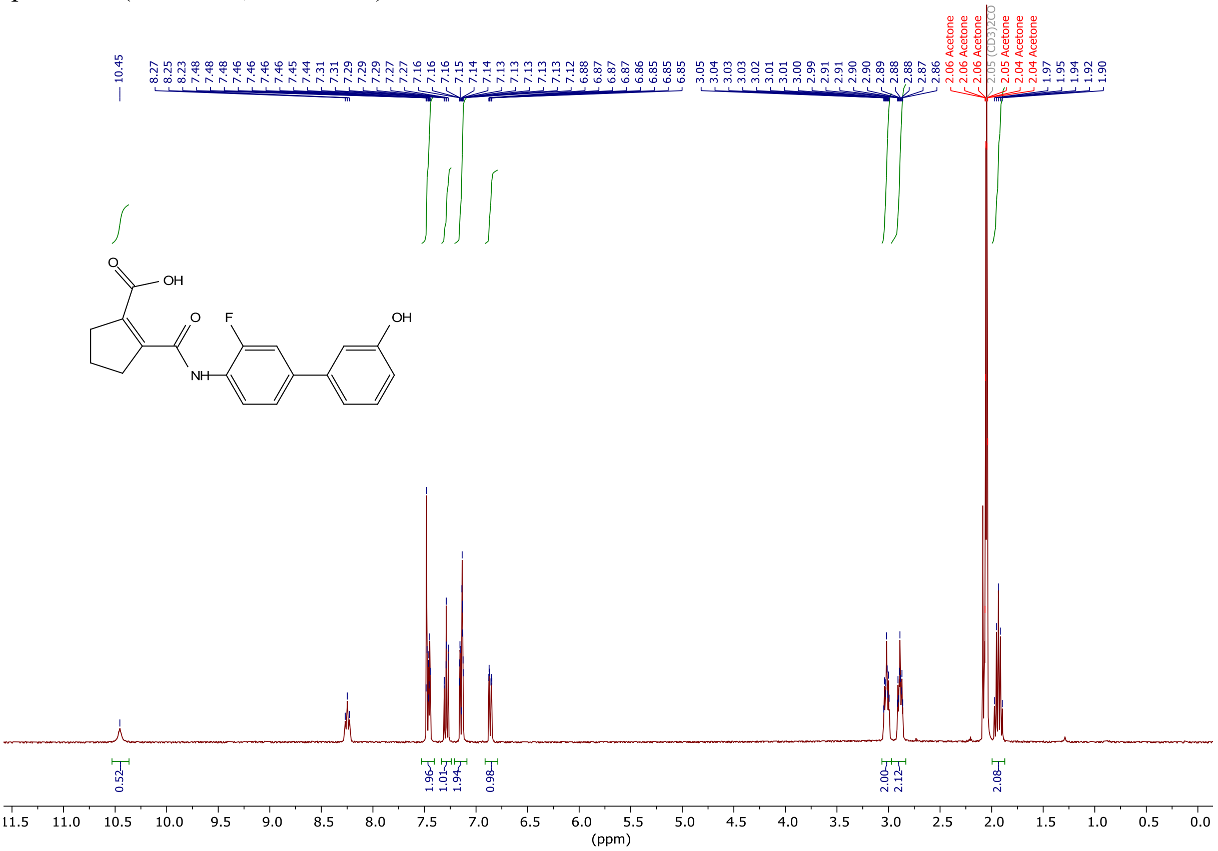

^{1}H NMR (400 MHz, acetone-d_6) of **19**

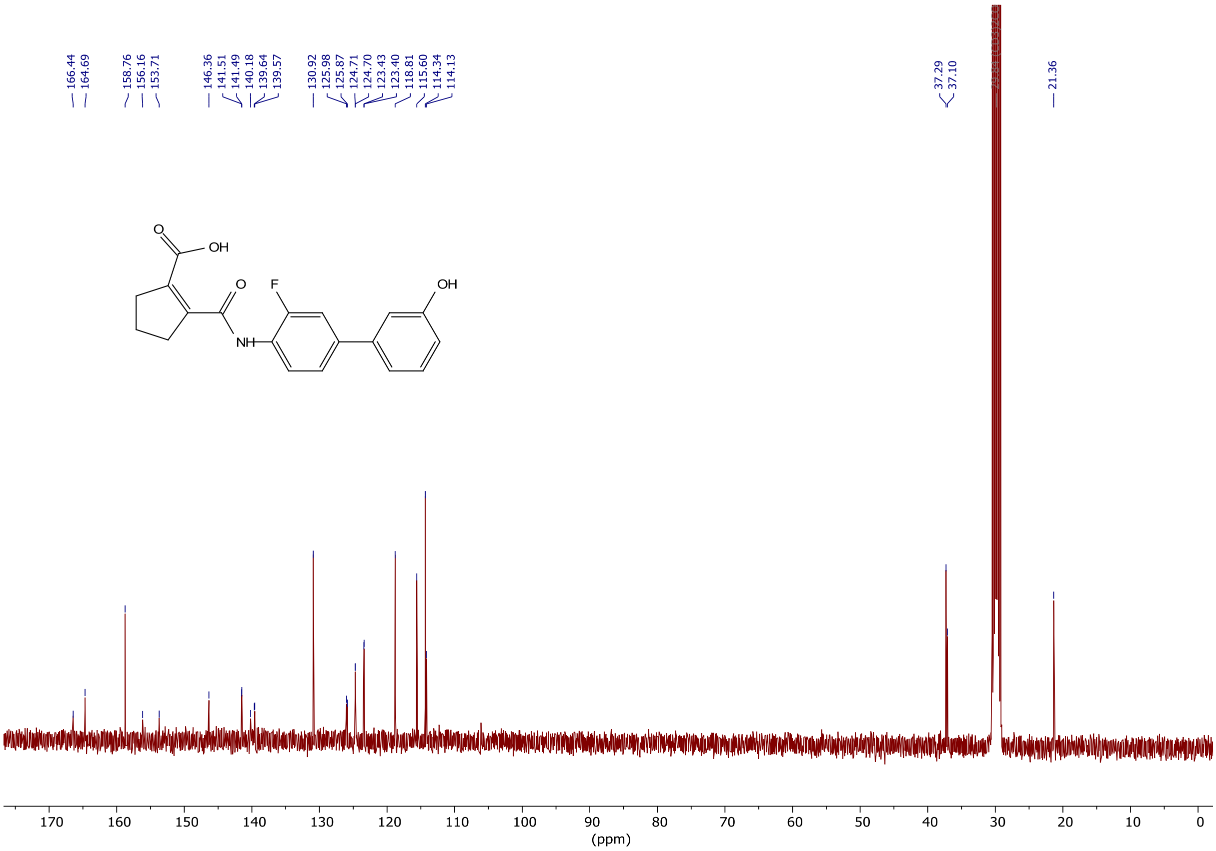

^{13}C NMR (101 MHz, acetone-d_6) of **19**

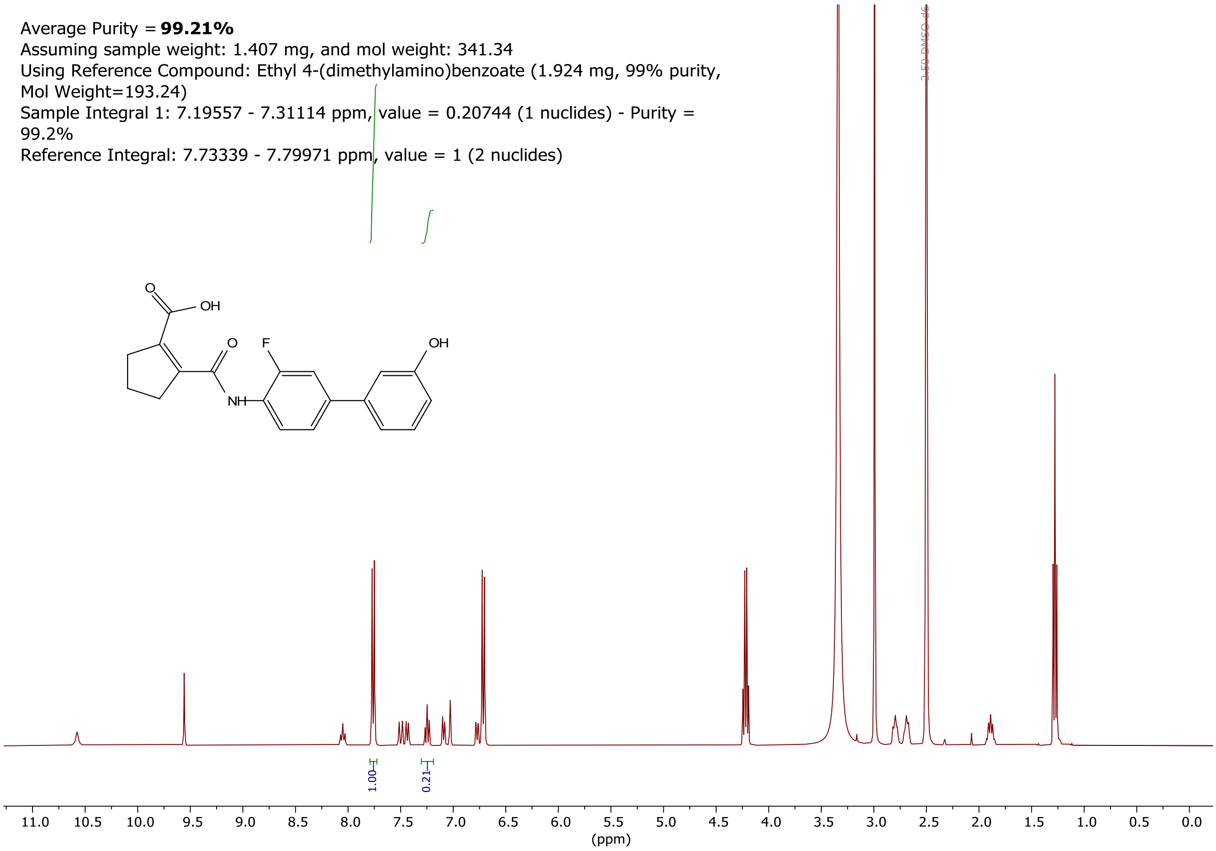

qH NMR (400 MHz, DMSO-d_6) of **19**

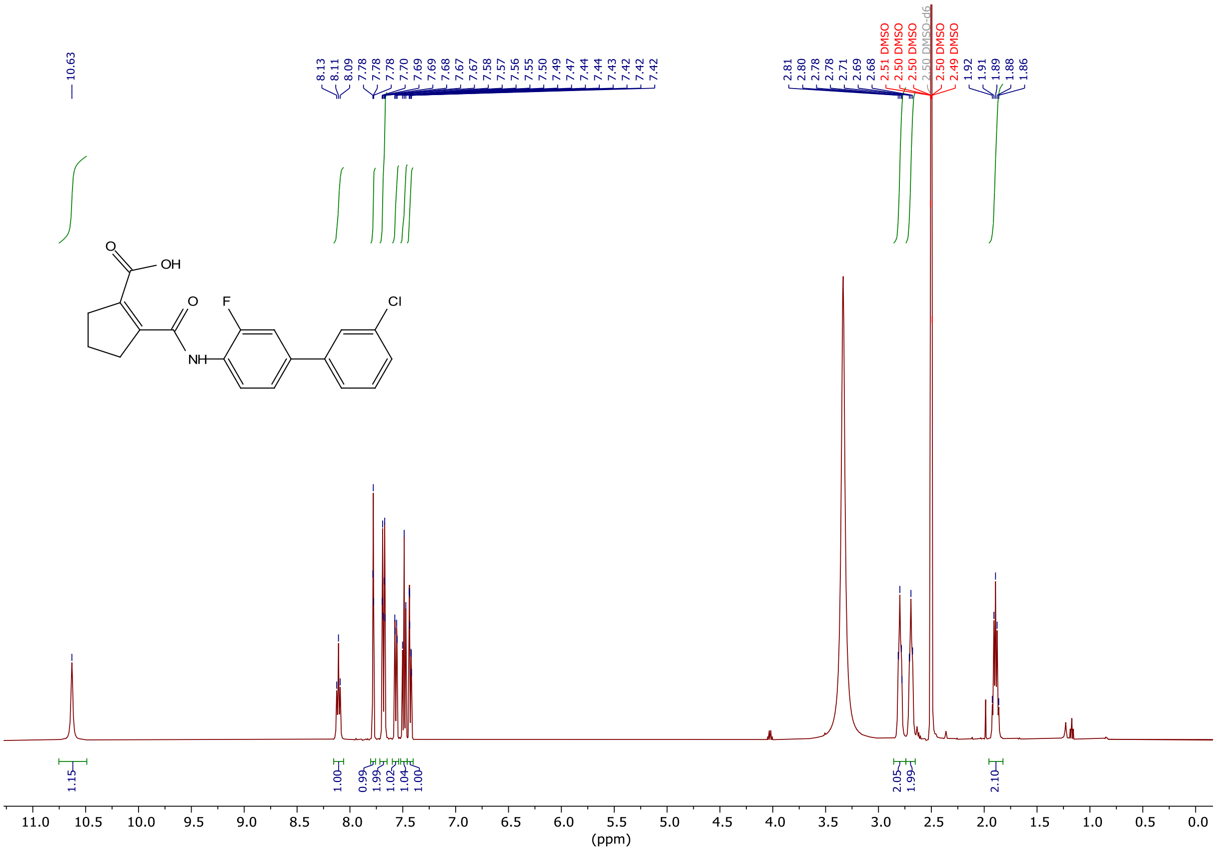

^{1}H NMR (500 MHz, DMSO-d_6) of **20**

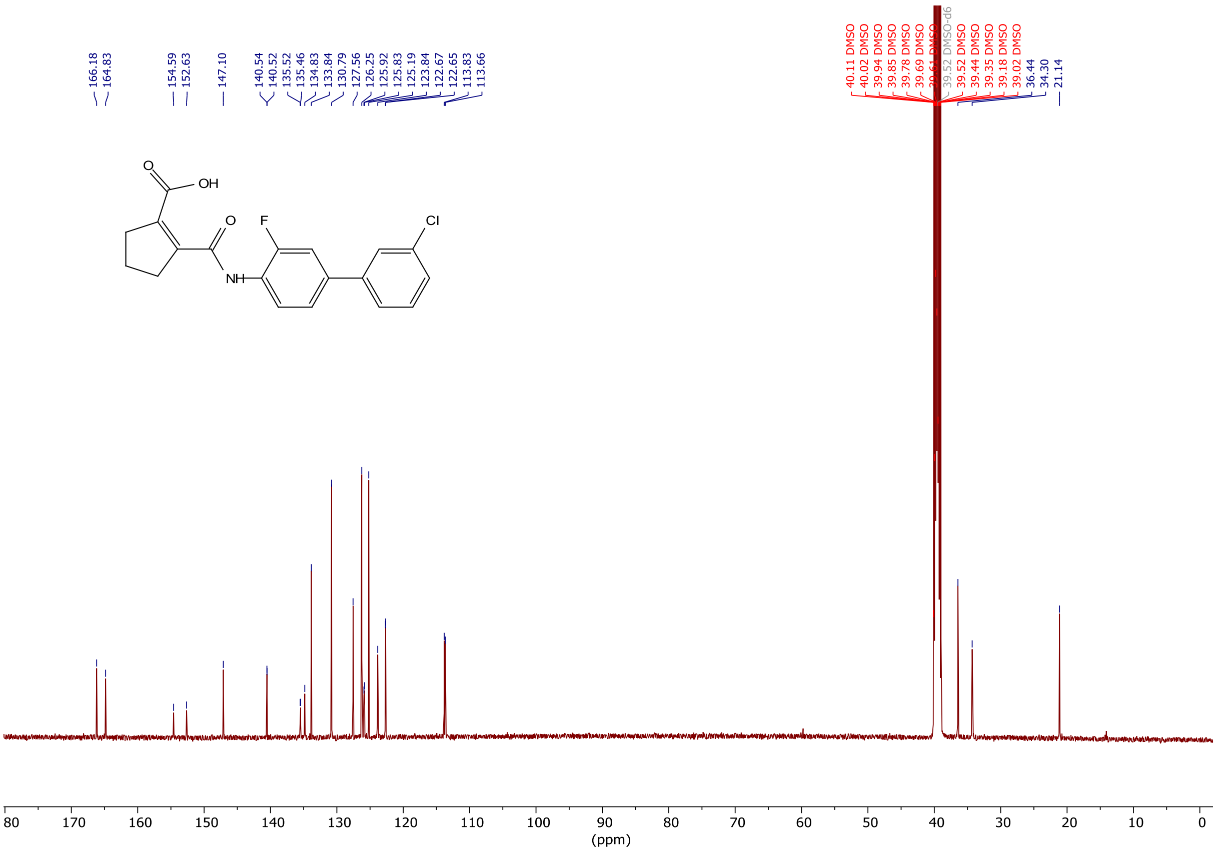

^{13}C NMR (126 MHz, DMSO-d_6) of **20**

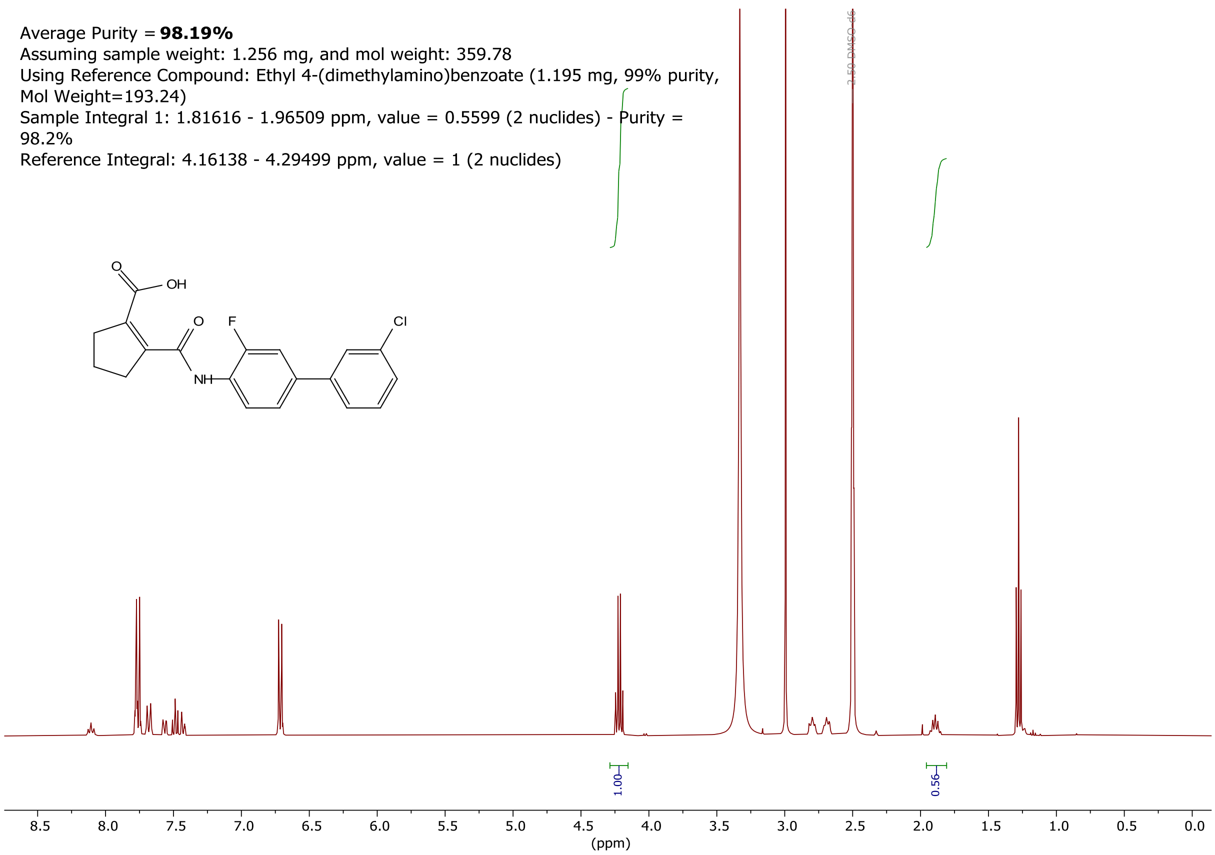

qH NMR (400 MHz, DMSO-d_6) of **20**

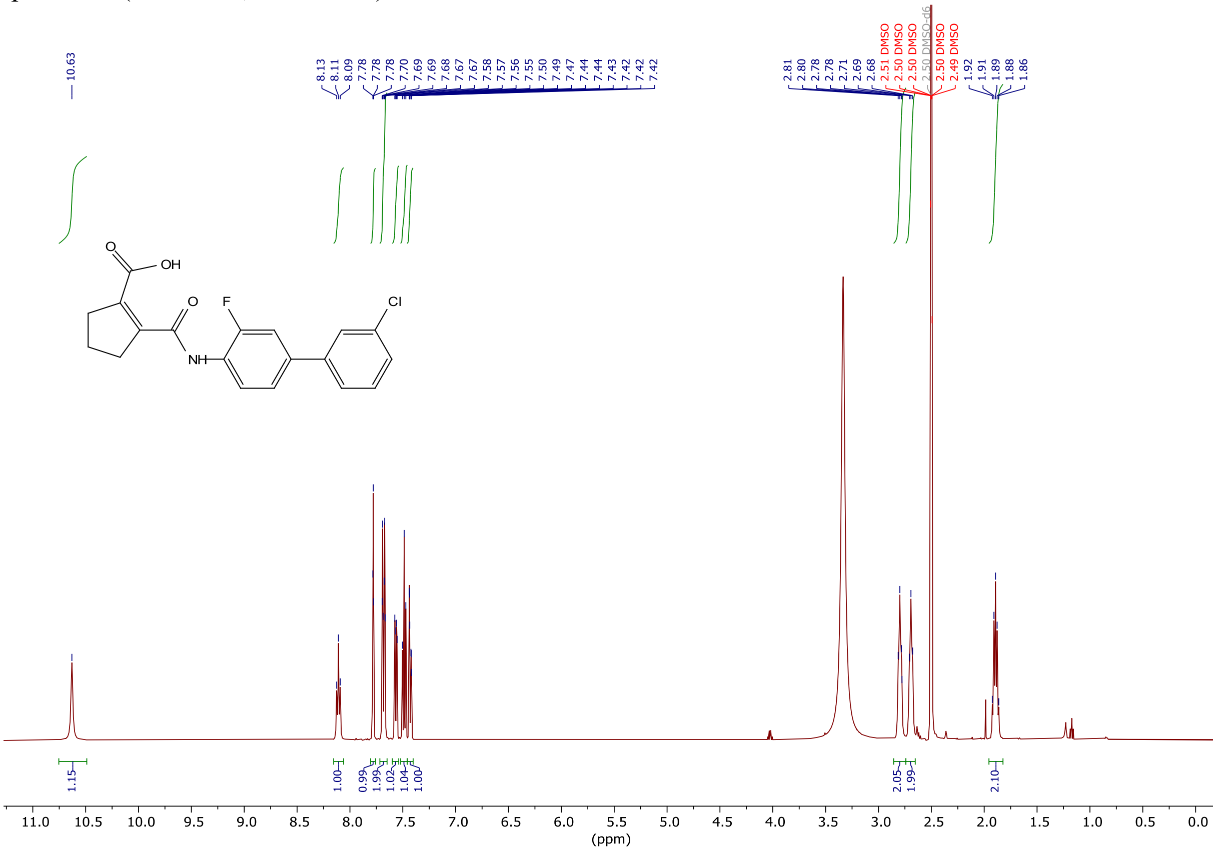

^{1}H NMR (500 MHz, DMSO-d_6) of **21**

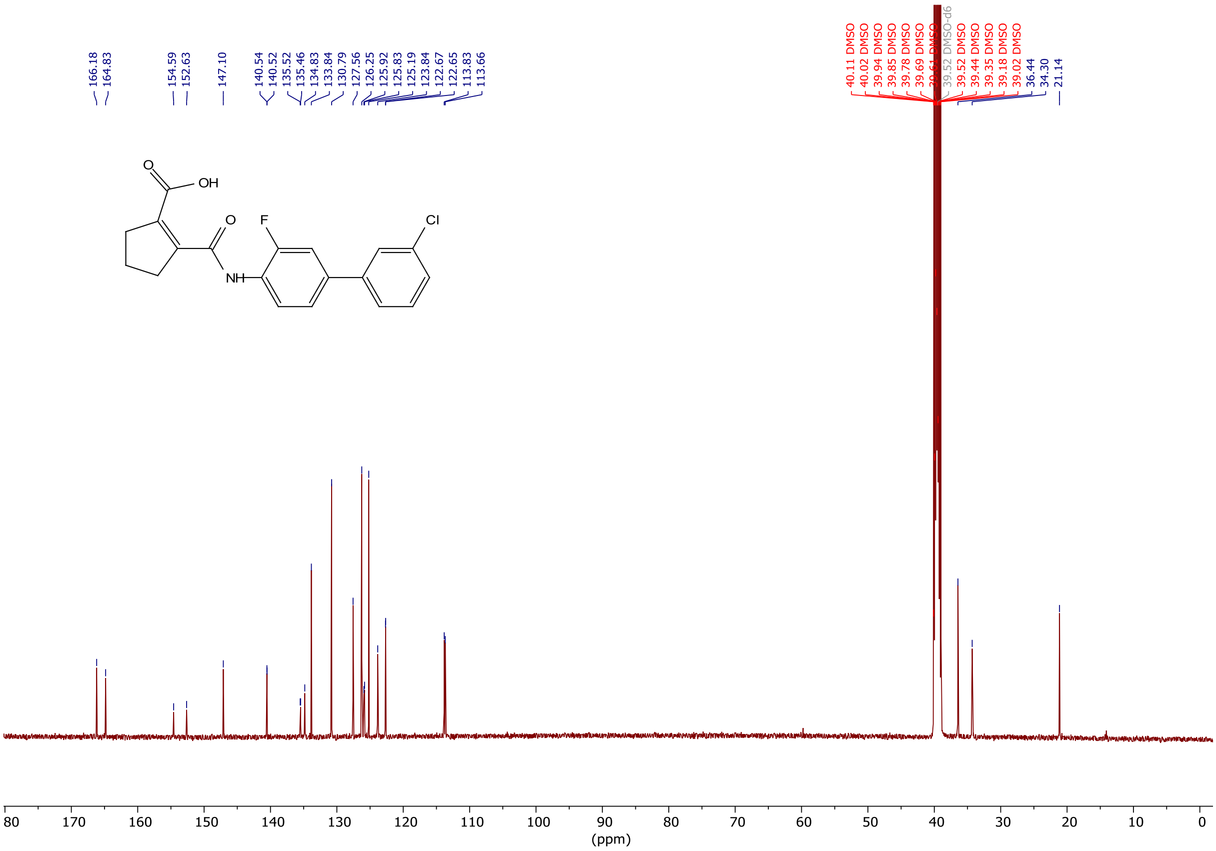

^{13}C NMR (126 MHz, DMSO-d_6) of **21**

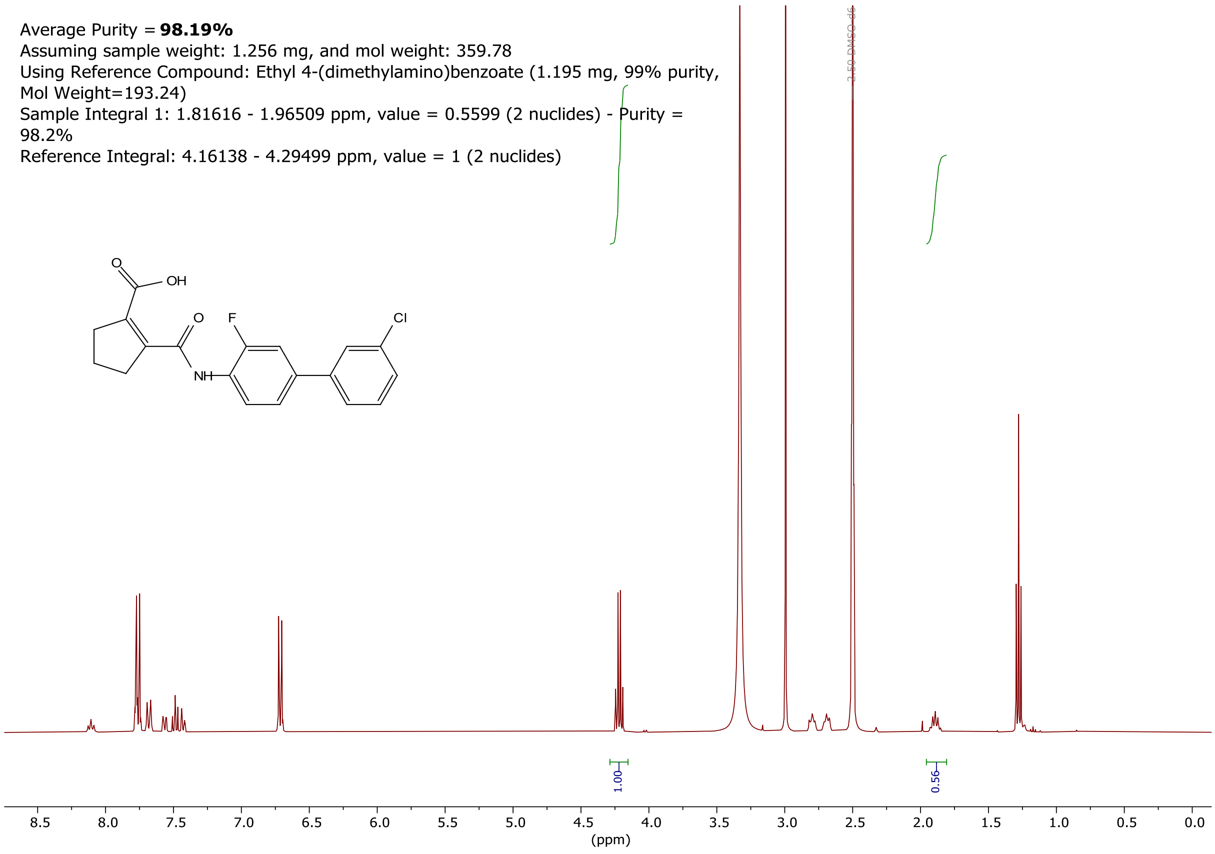

qH NMR (400 MHz, DMSO-d_6) of **21**

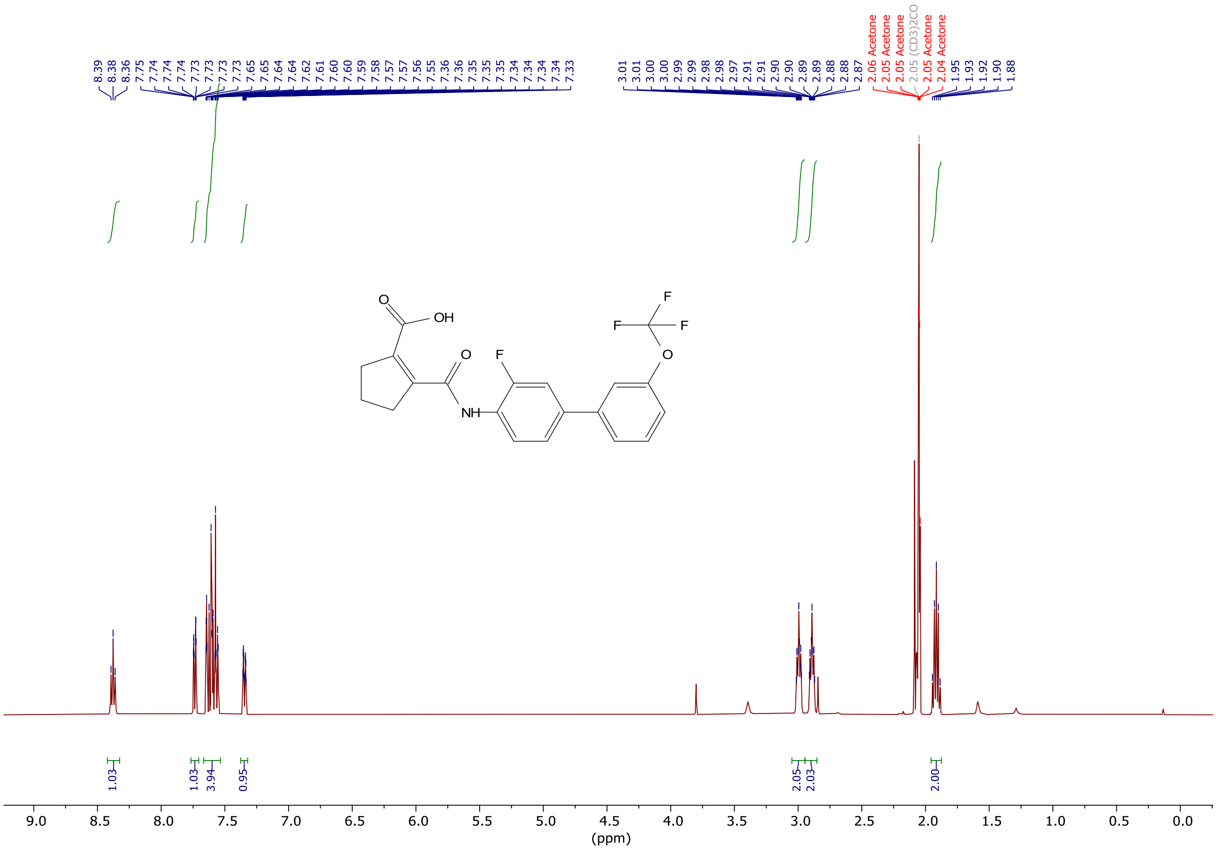

^{1}H NMR (500 MHz, acetone-d_6) of **22**

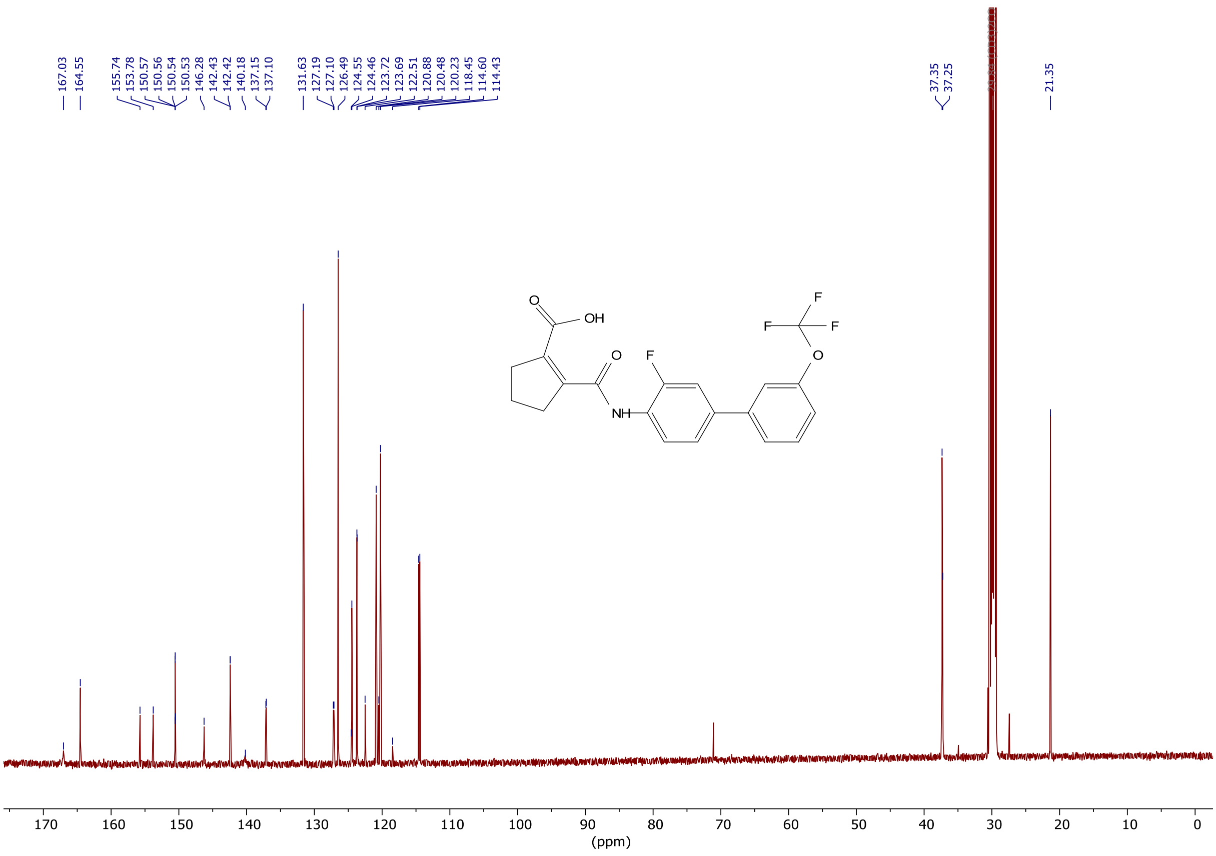

^{13}C NMR (126 MHz, acetone-d_6) of **22**

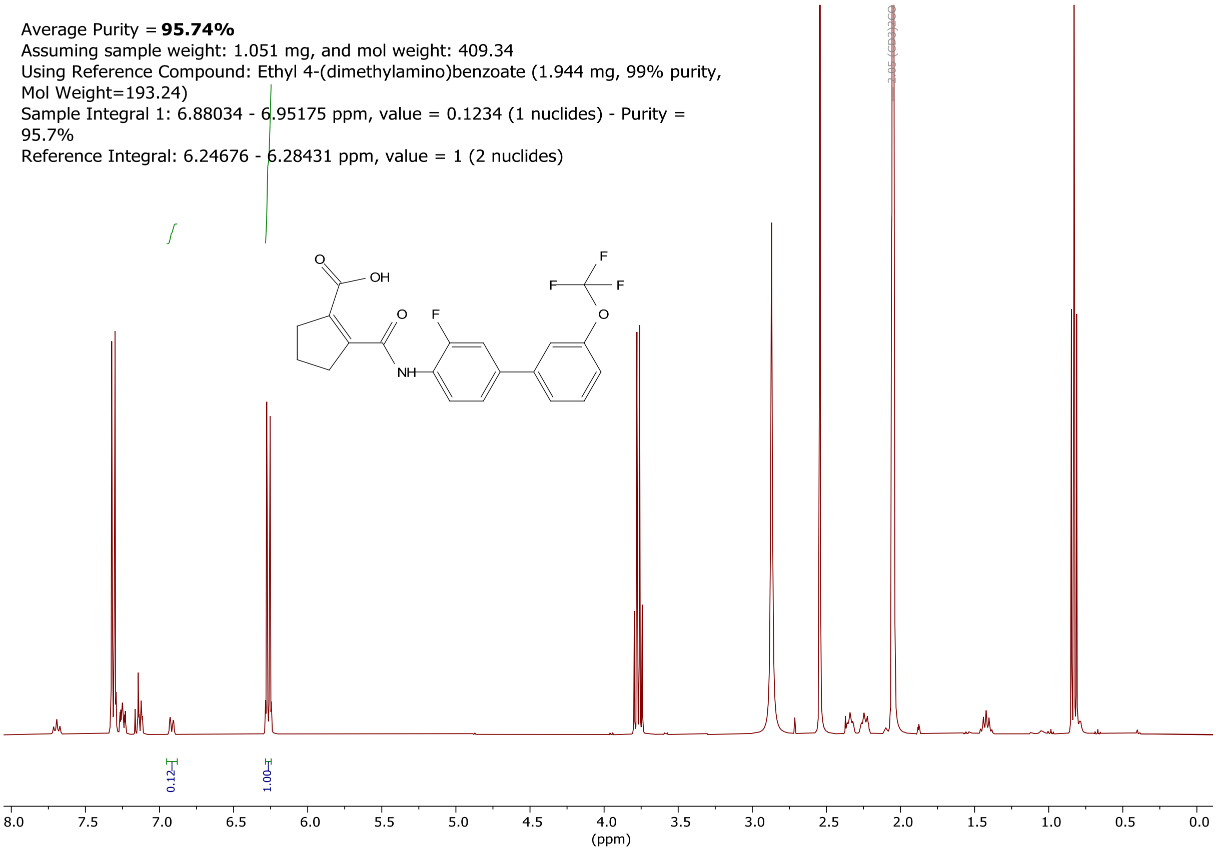

qH NMR (400 MHz, DMSO-d_6) of **22**

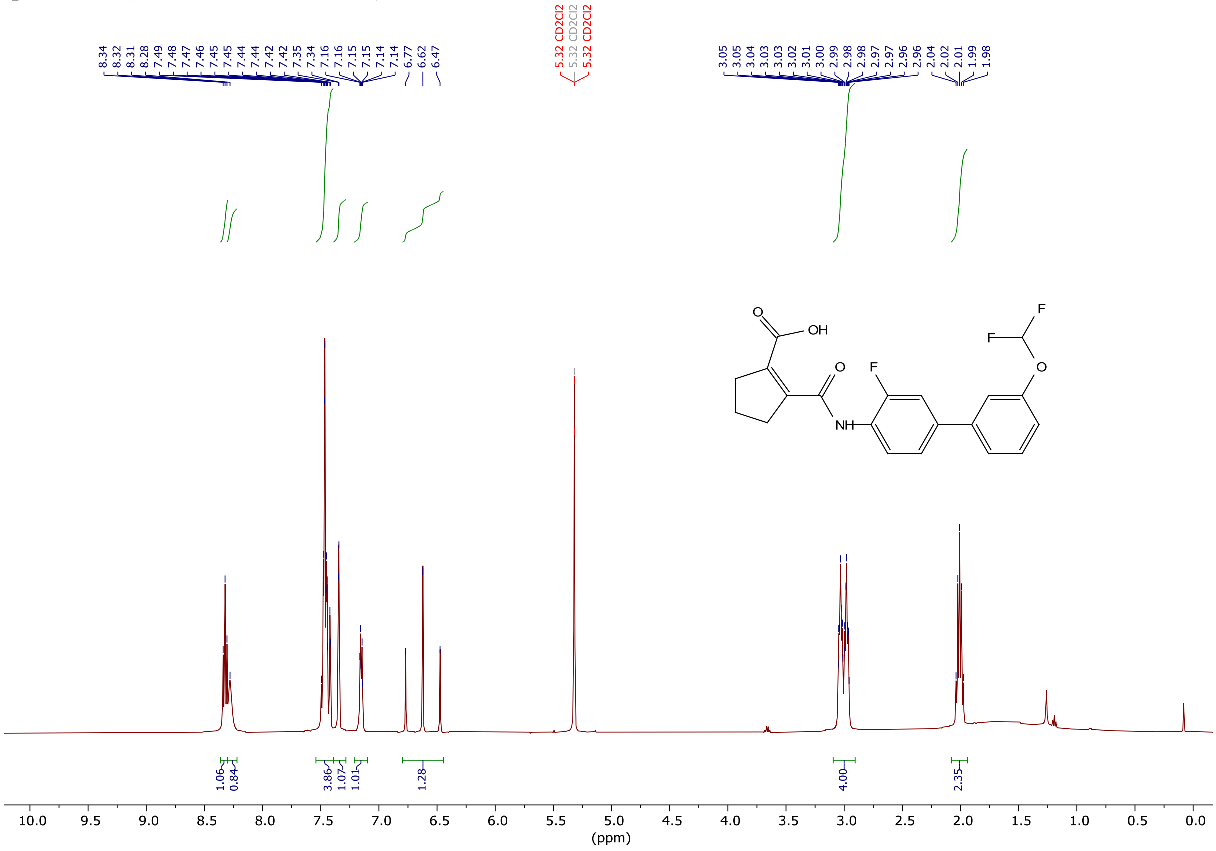

^{1}H NMR (500 MHz, CD_2Cl_2) of **23**

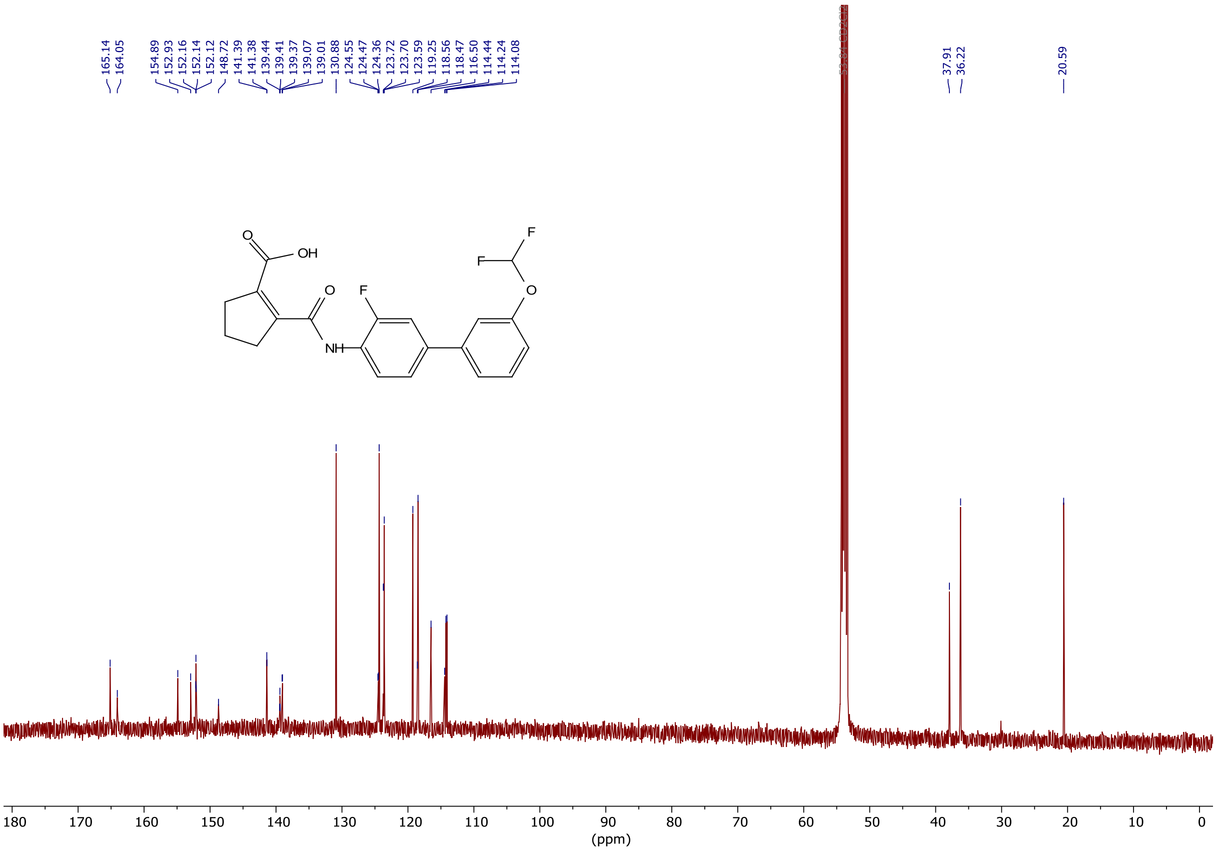

^{13}C NMR (126 MHz, CD_2Cl_2) of **23**

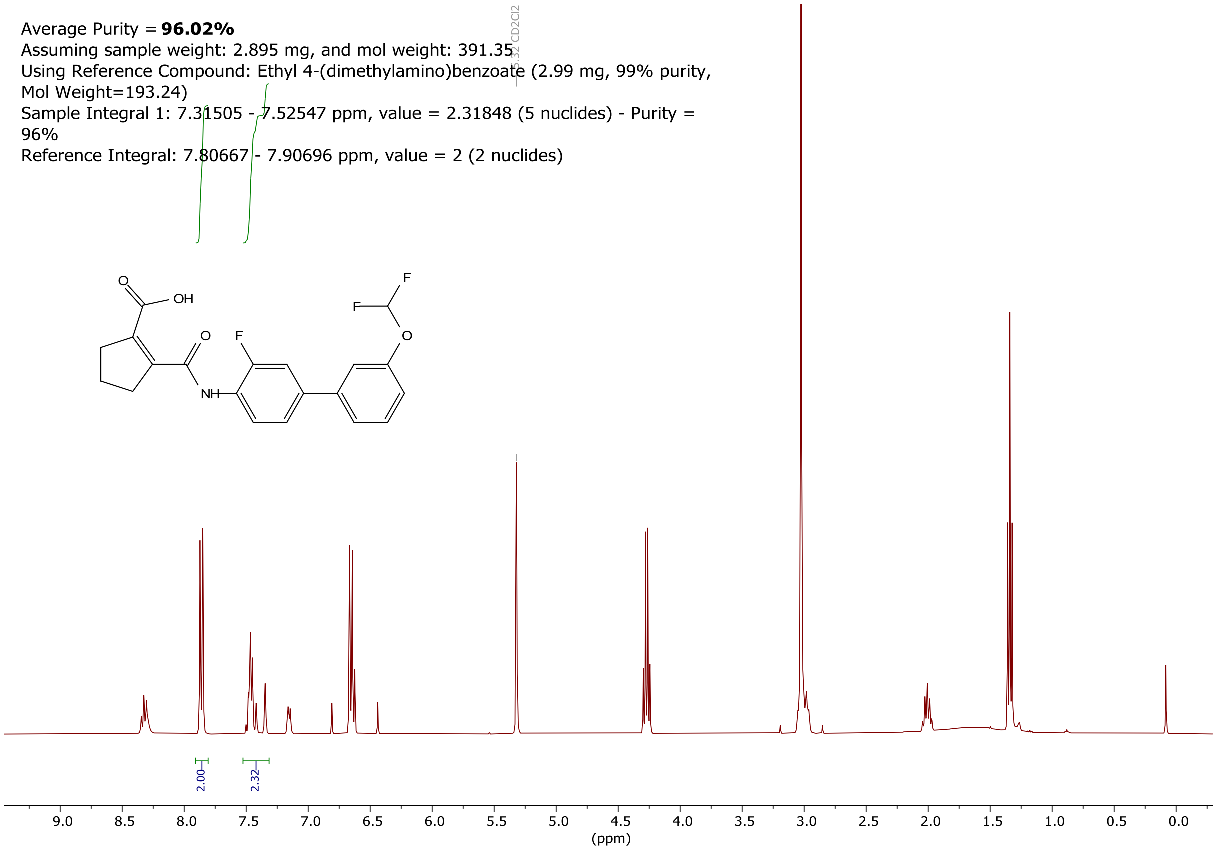

qH NMR (400 MHz, CD_2Cl_2) of **23**

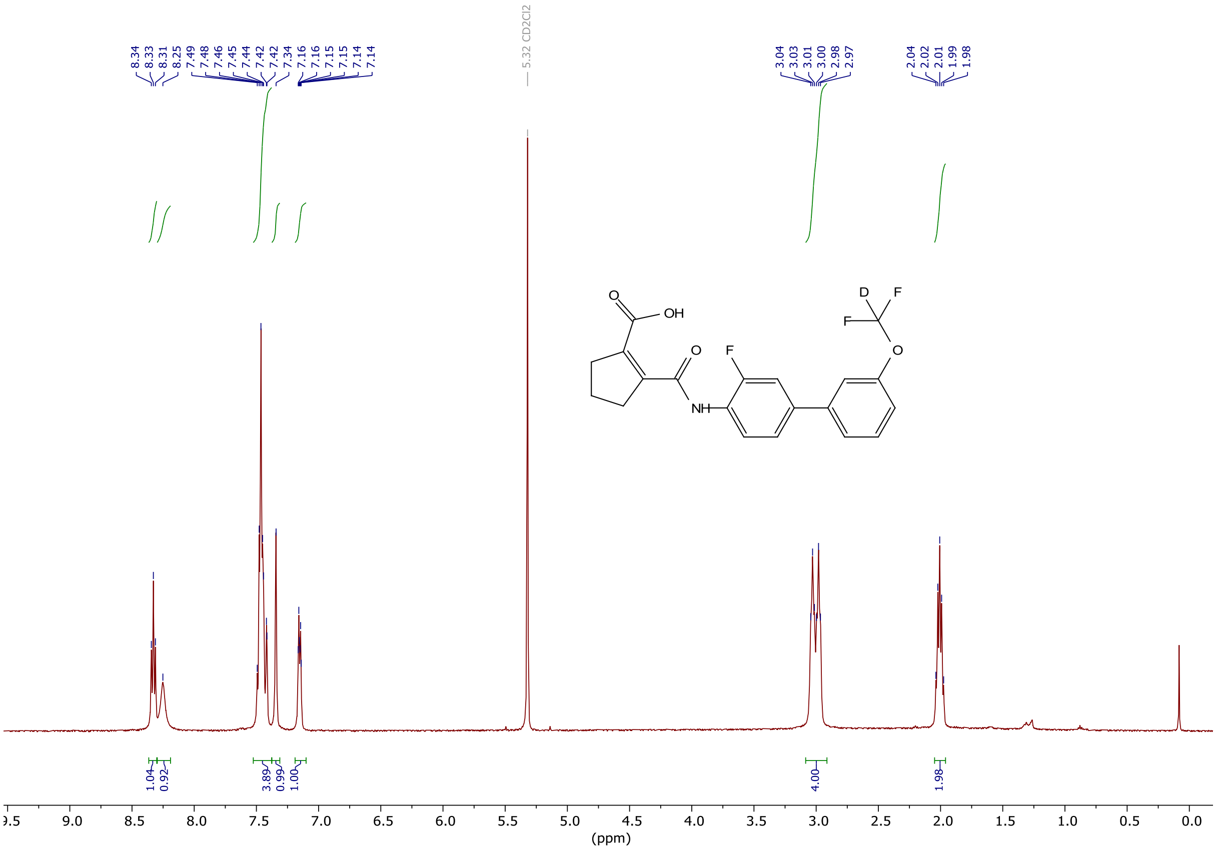

^{1}H NMR (500 MHz, CD_2Cl_2) of **24**

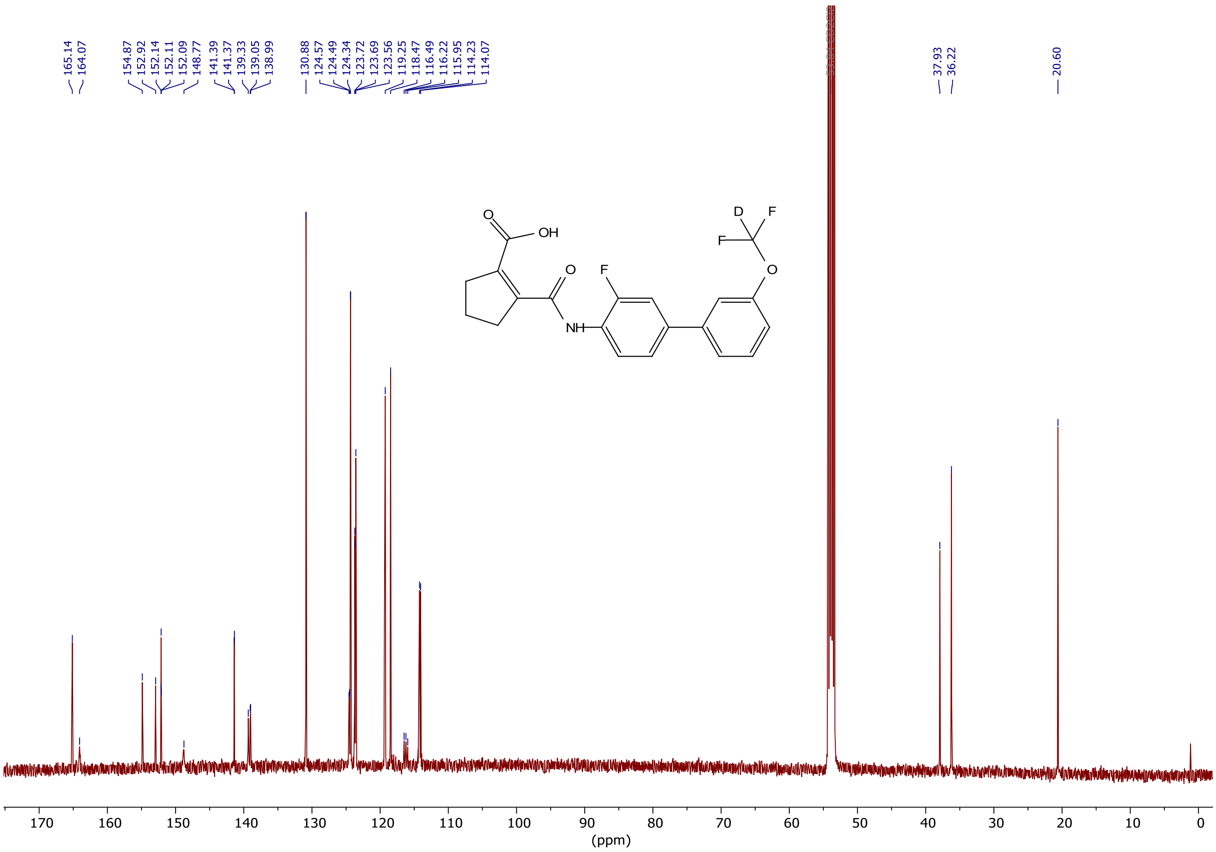

^{13}C NMR (126 MHz, CD_2Cl_2) of **24**

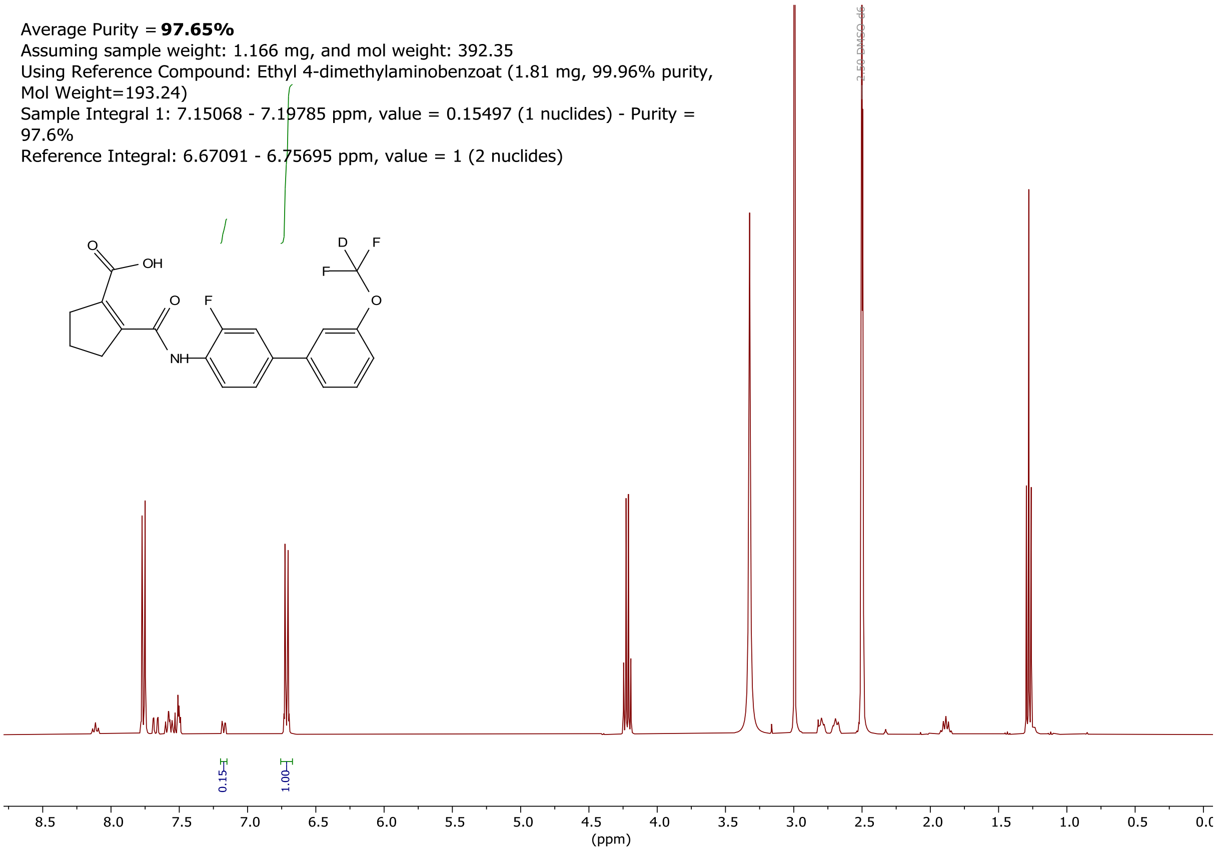

qH NMR (400 MHz, DMSO-d_6) of **24**

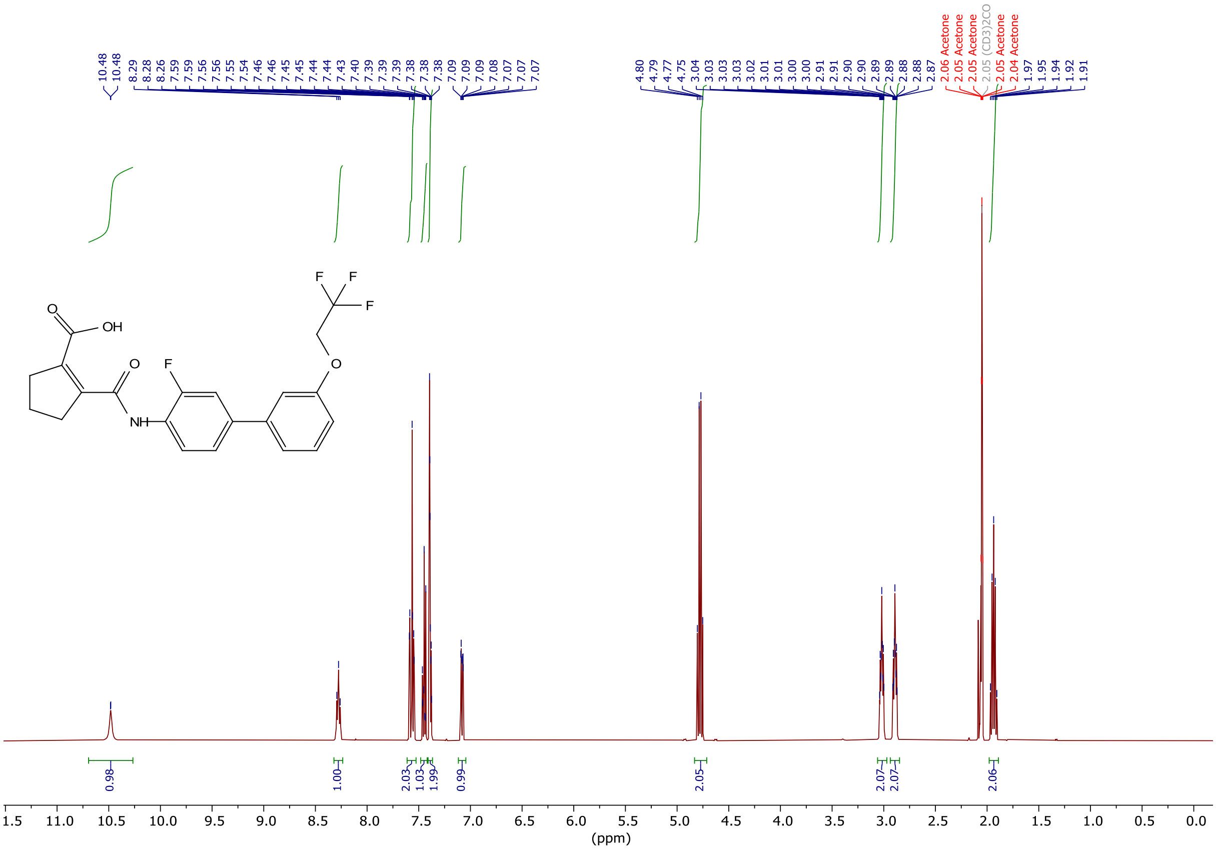

^{1}H NMR (500 MHz, acetone-d_6) of **25**

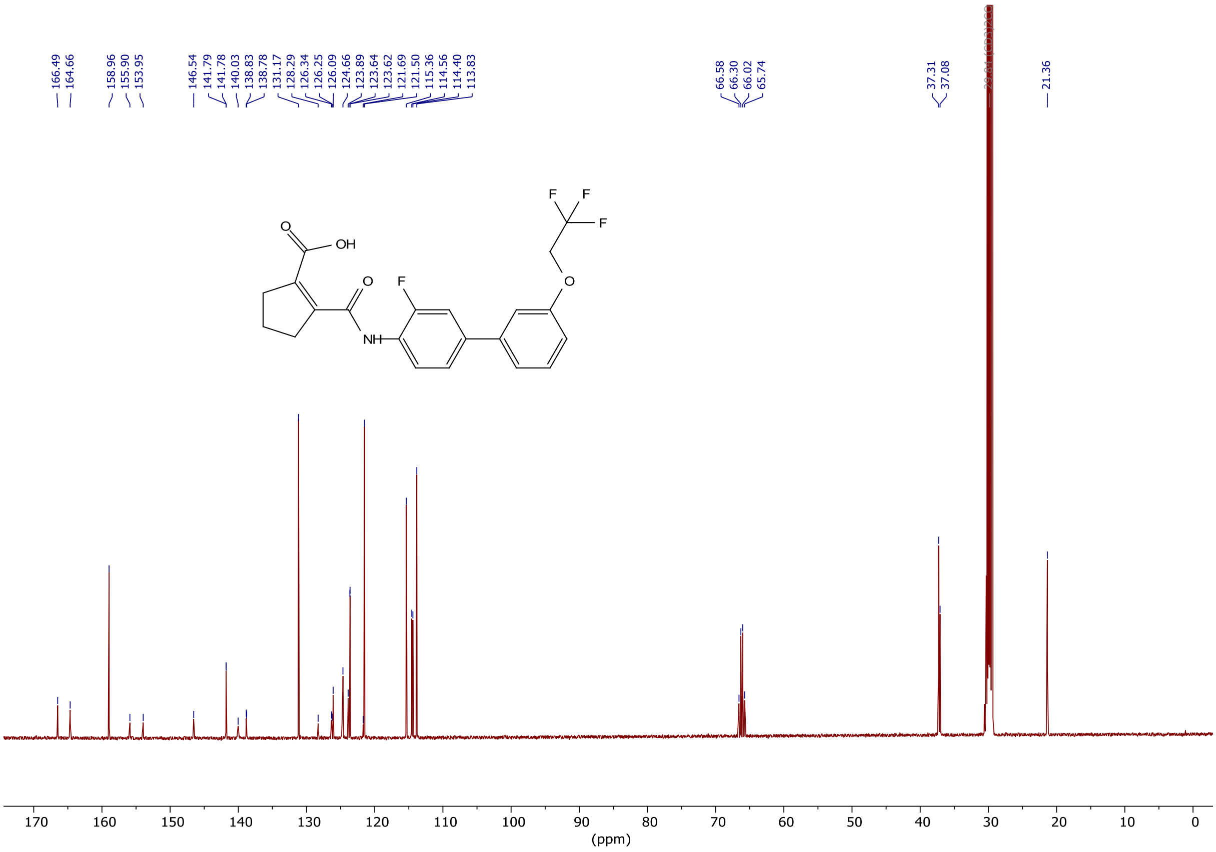

^{13}C NMR (126 MHz, acetone-d_6) of **25**

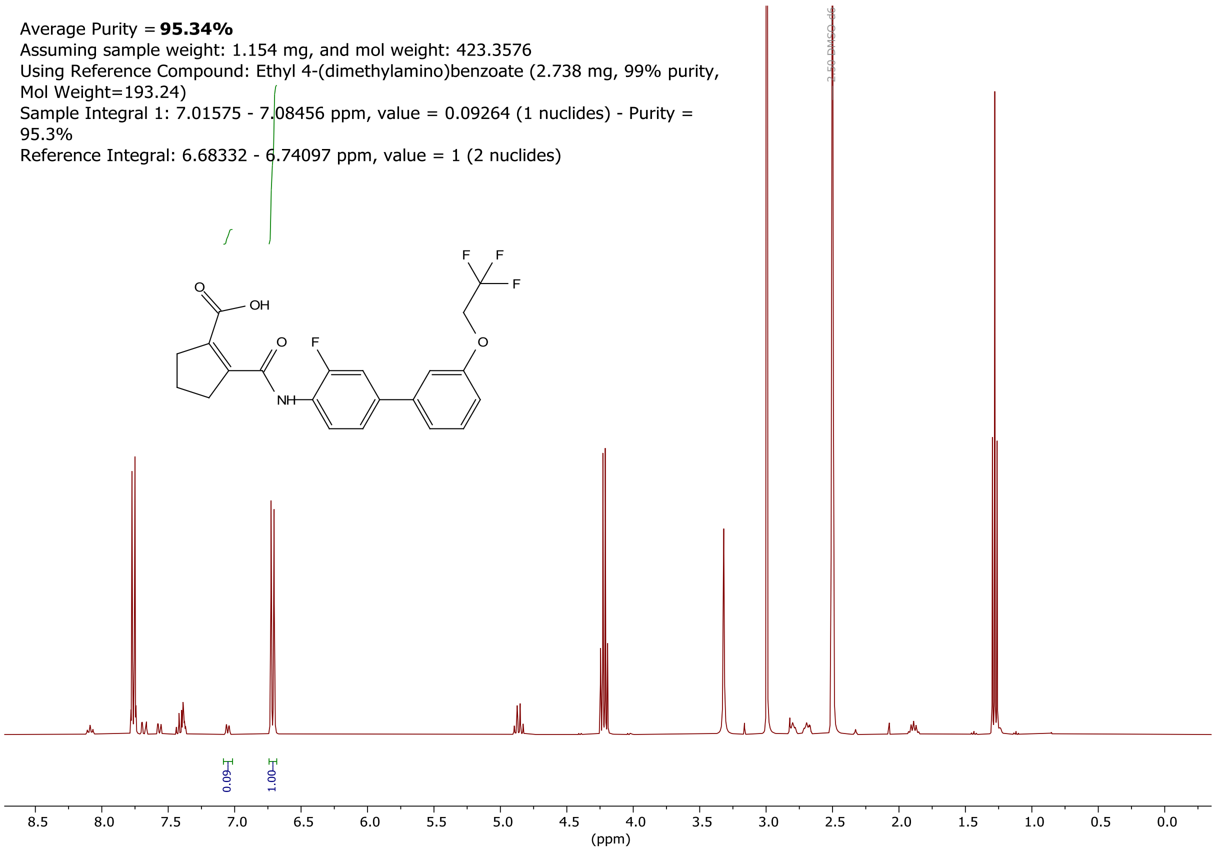

qH NMR (400 MHz, DMSO-d_6) of **25**

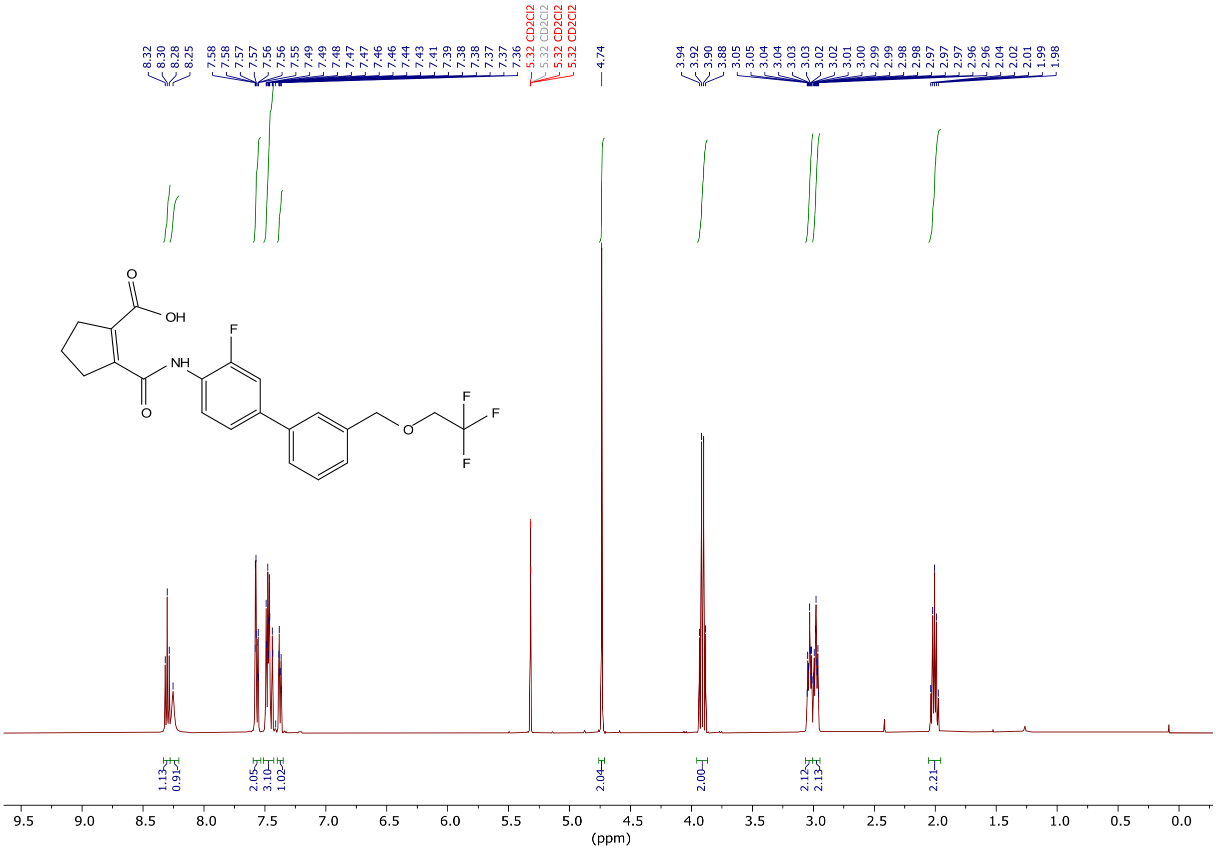

^{1}H NMR (500 MHz, CD_2Cl_2) of **26**

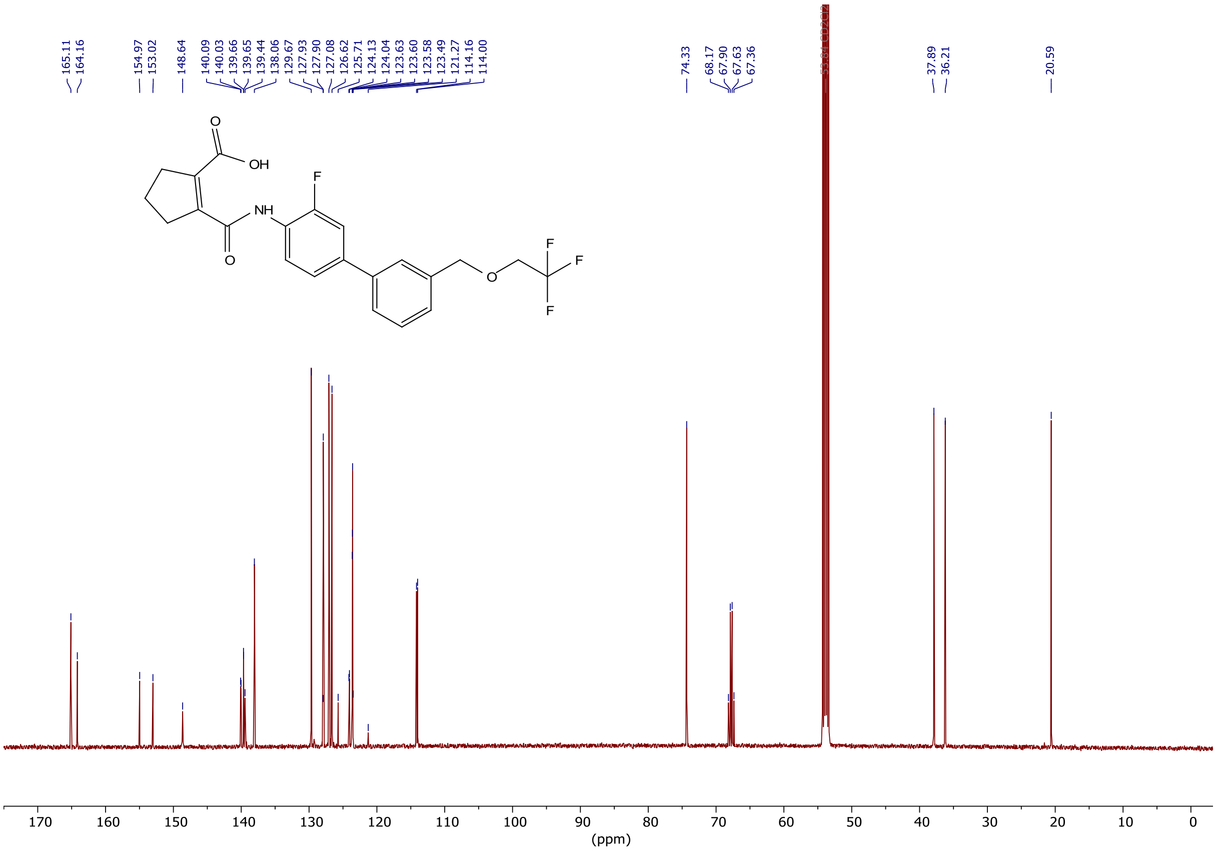

^{13}C NMR (126 MHz, CD_2Cl_2) of **26**

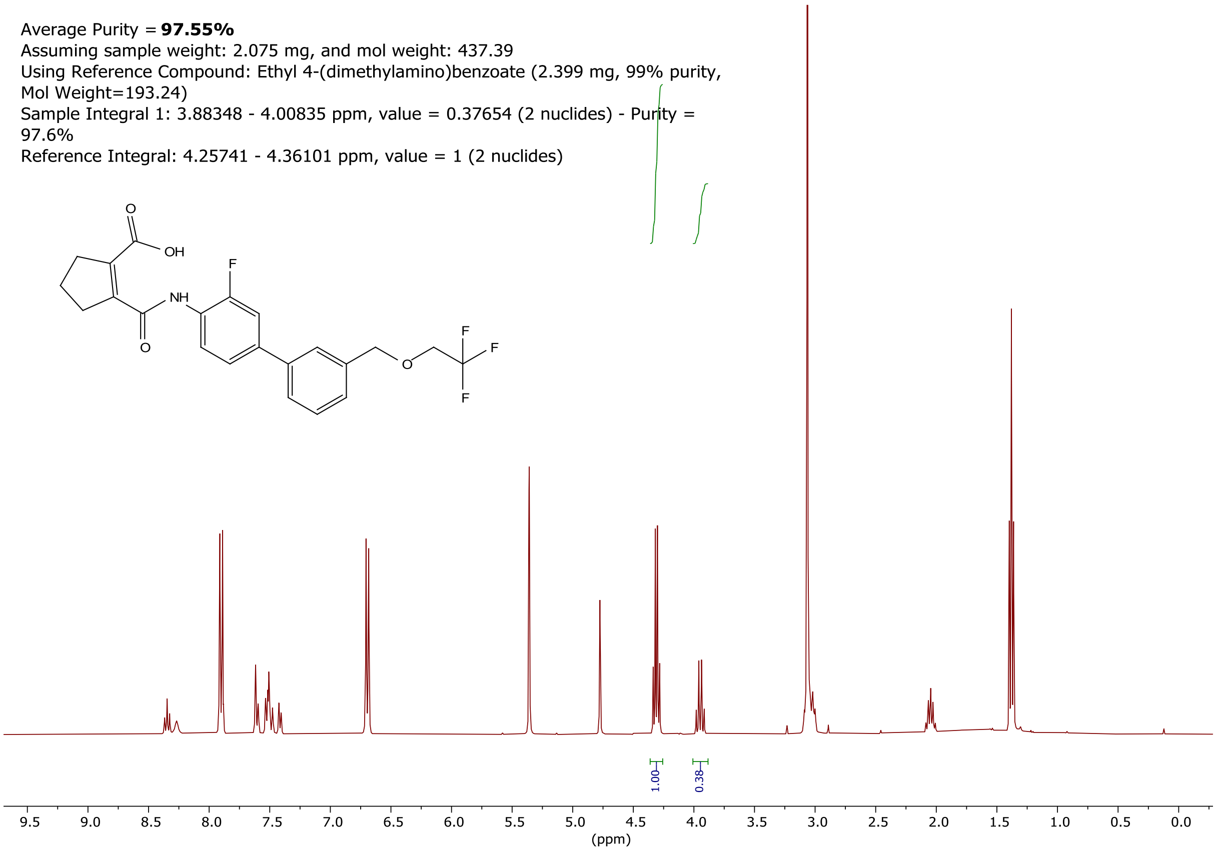

qH NMR (400 MHz, DMSO-d_6) of **26**

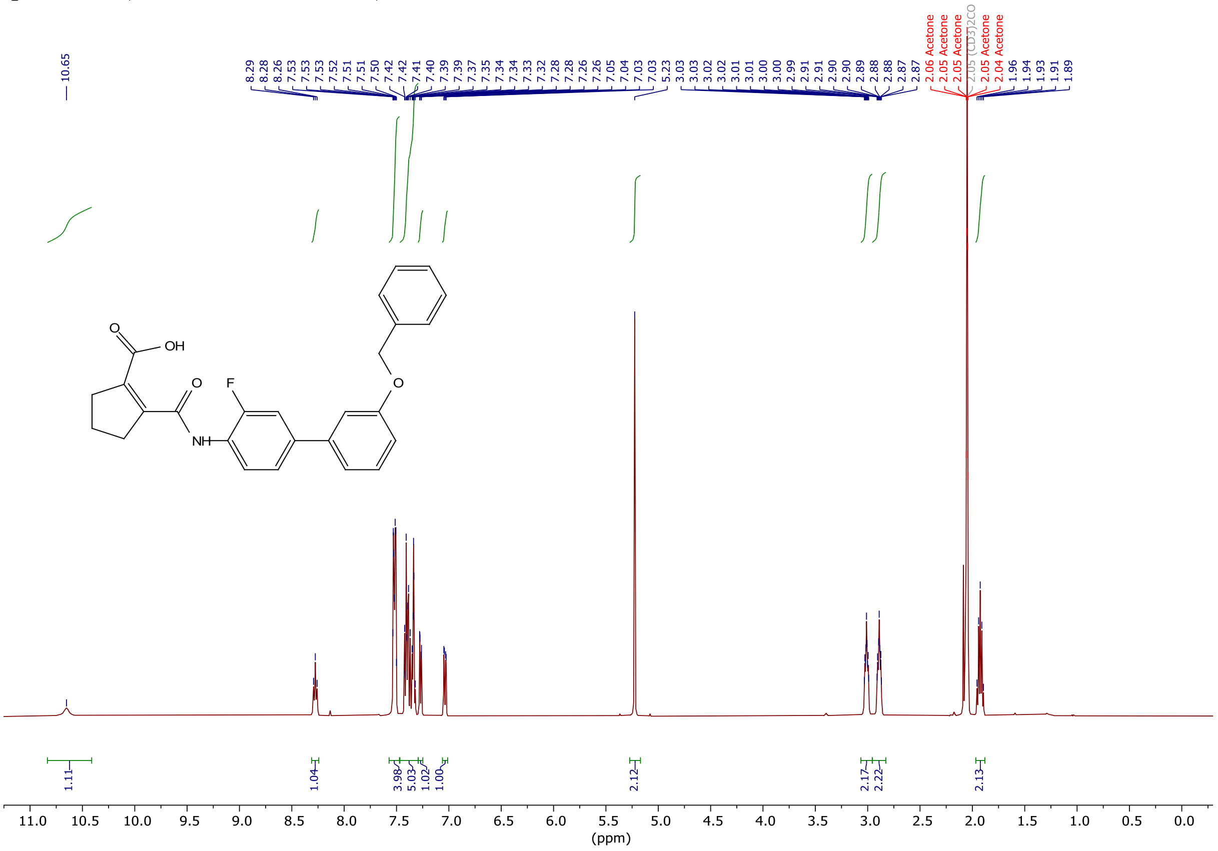

^{1}H NMR (500 MHz, acetone-d_6) of **27**

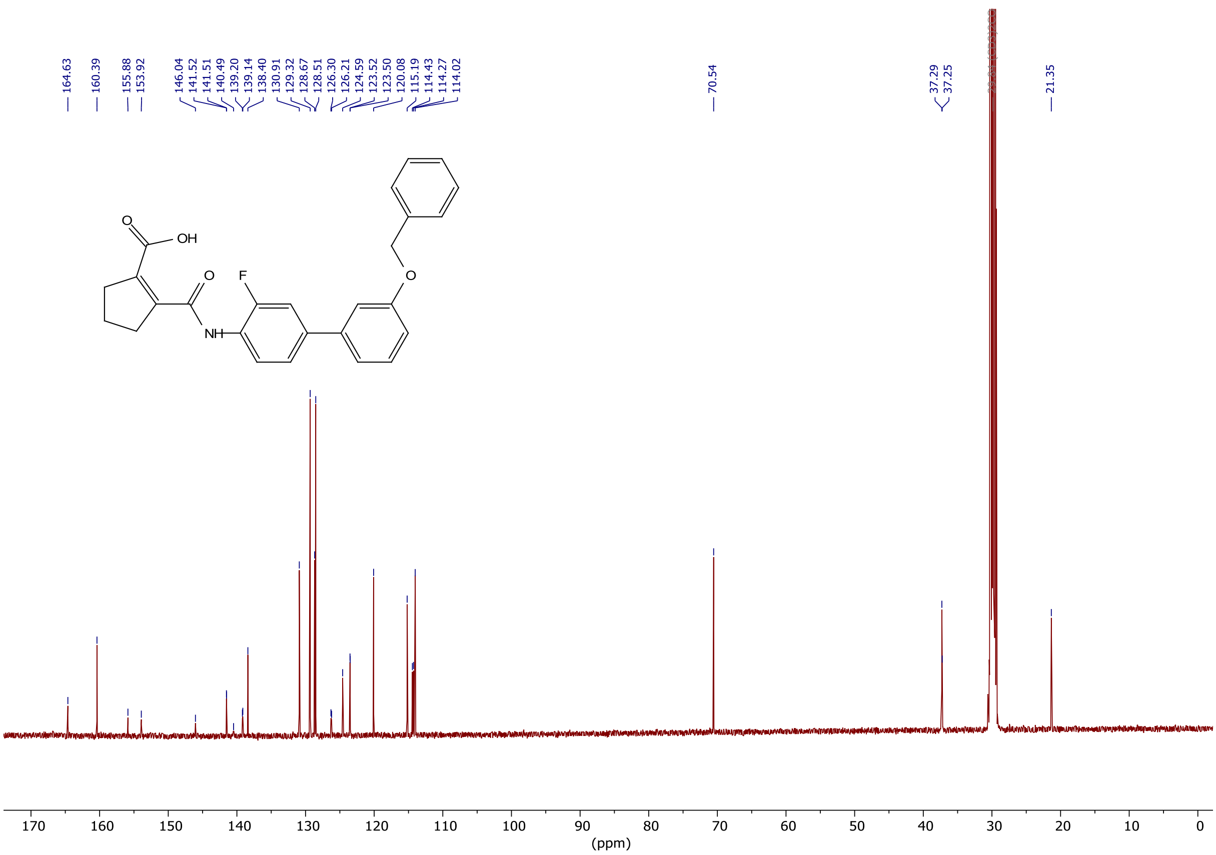

^{13}C NMR (126 MHz, acetone-d_6) of **27**

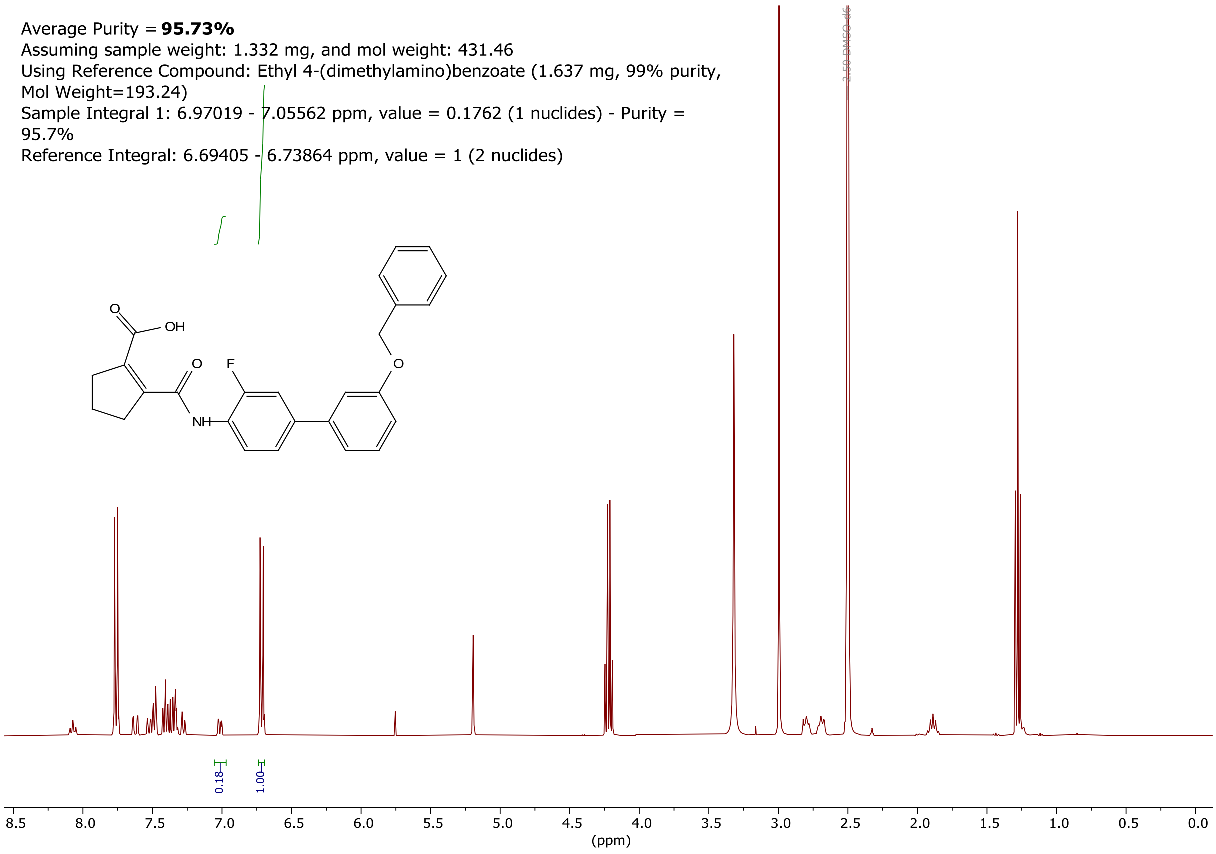

qH NMR (400 MHz, DMSO-d_6) of **27**

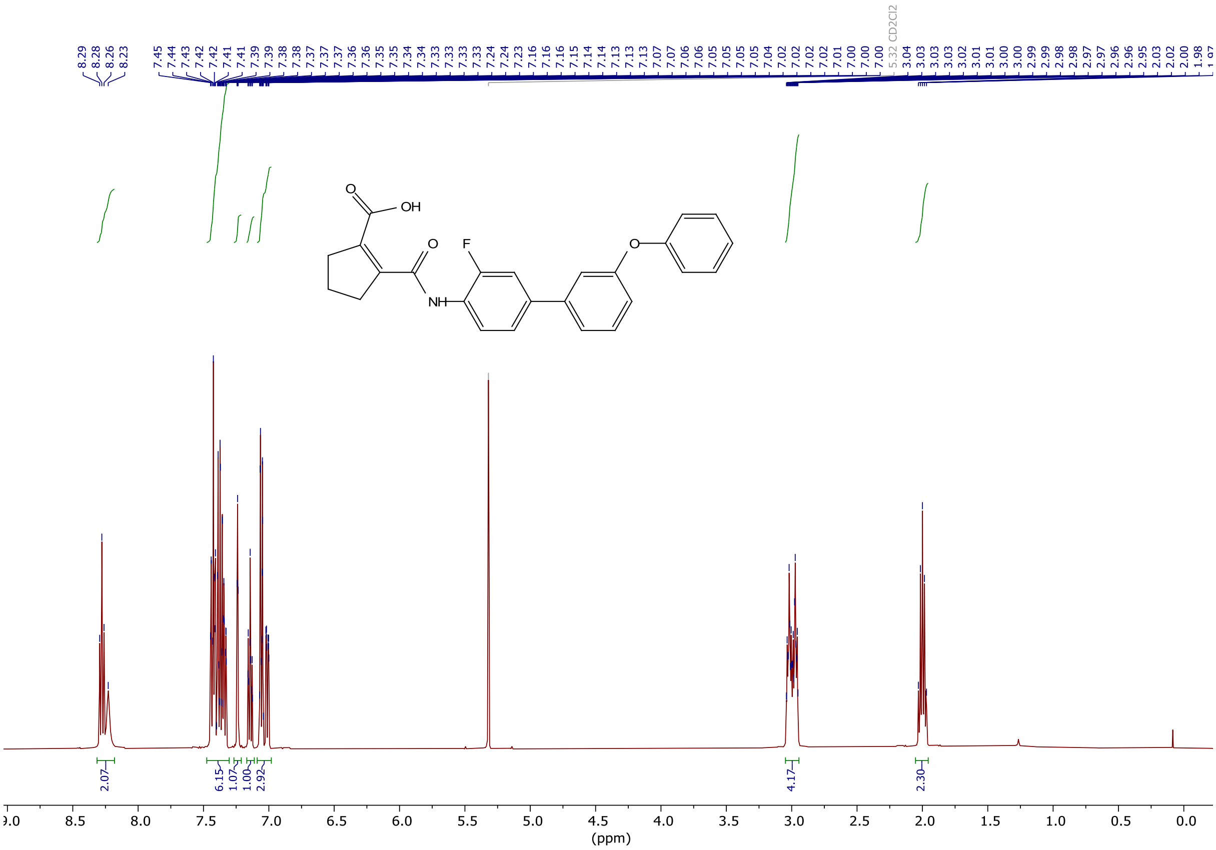

^{1}H NMR (500 MHz, CD_2Cl_2) of **28**

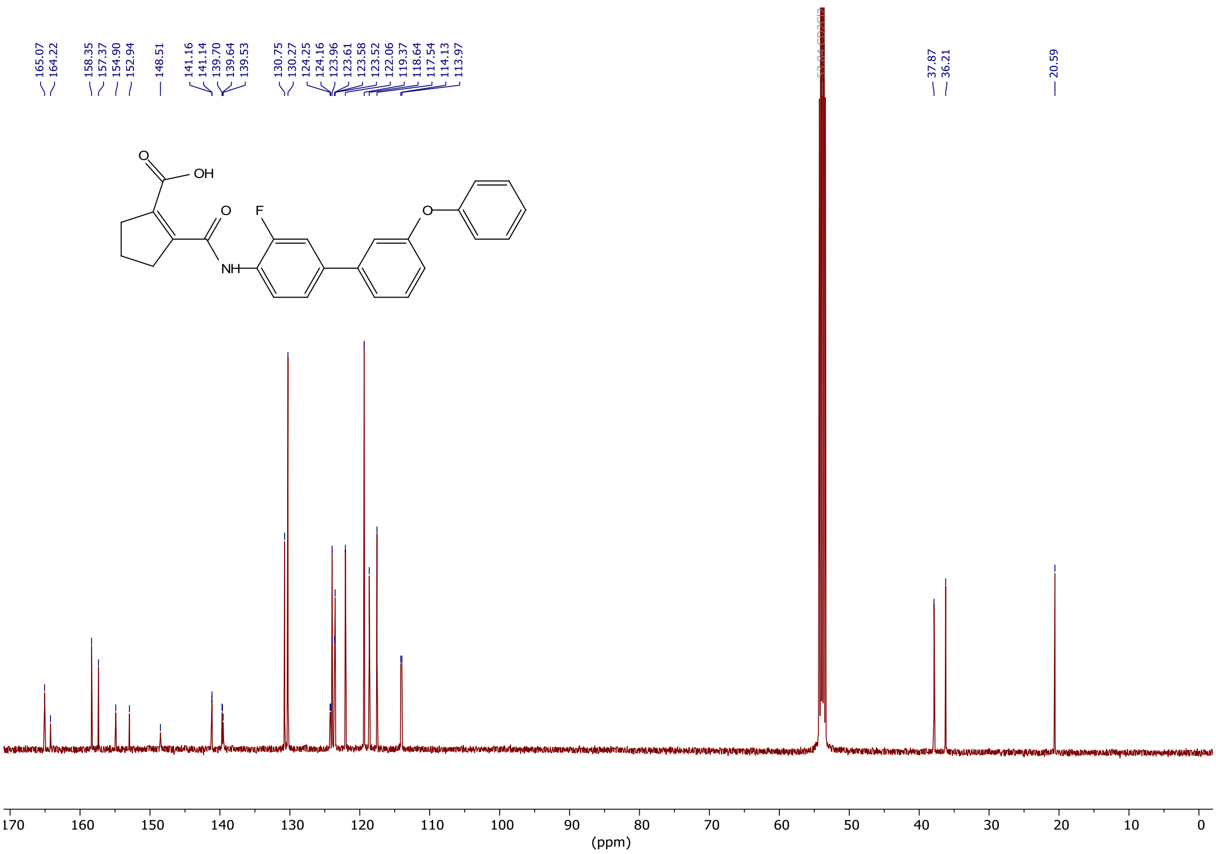

^{13}C NMR (126 MHz, CD_2Cl_2) of **28**

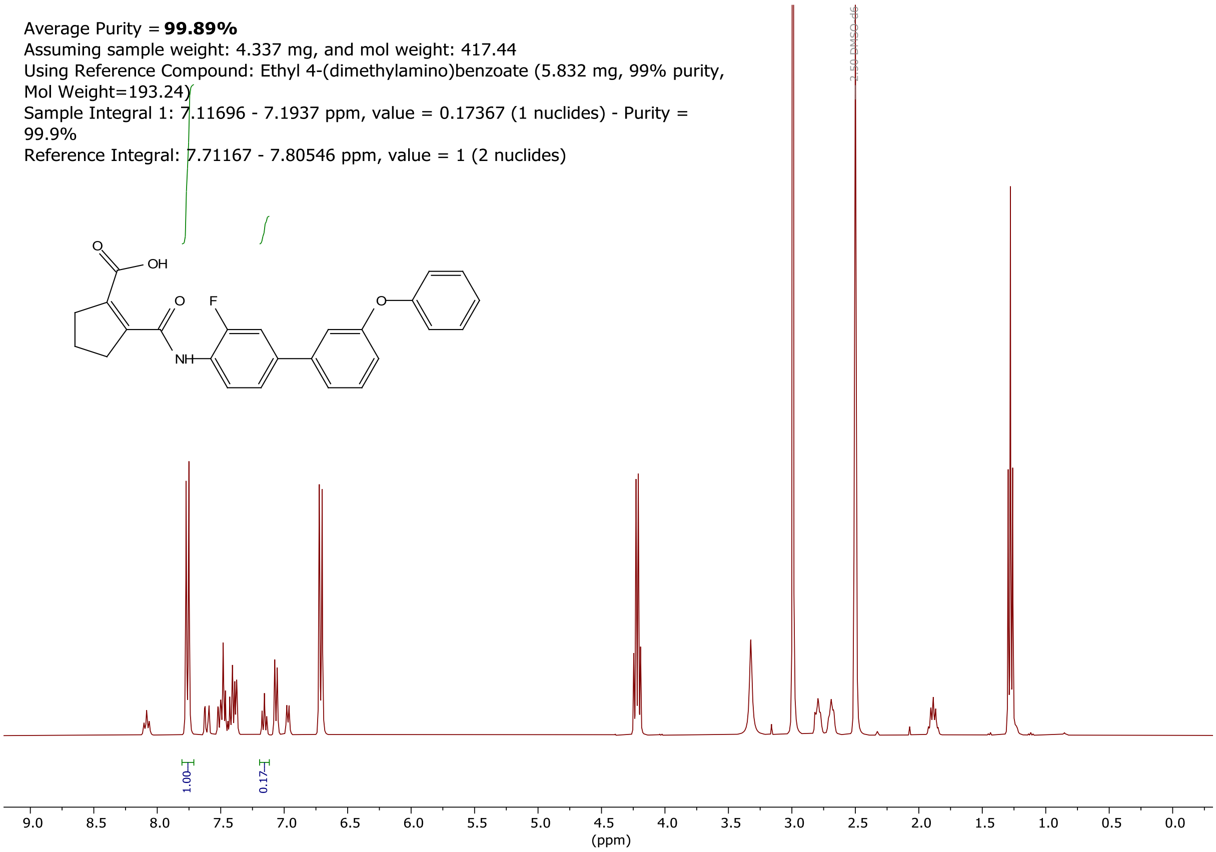

qH NMR (400 MHz, DMSO-d_6) of **28**

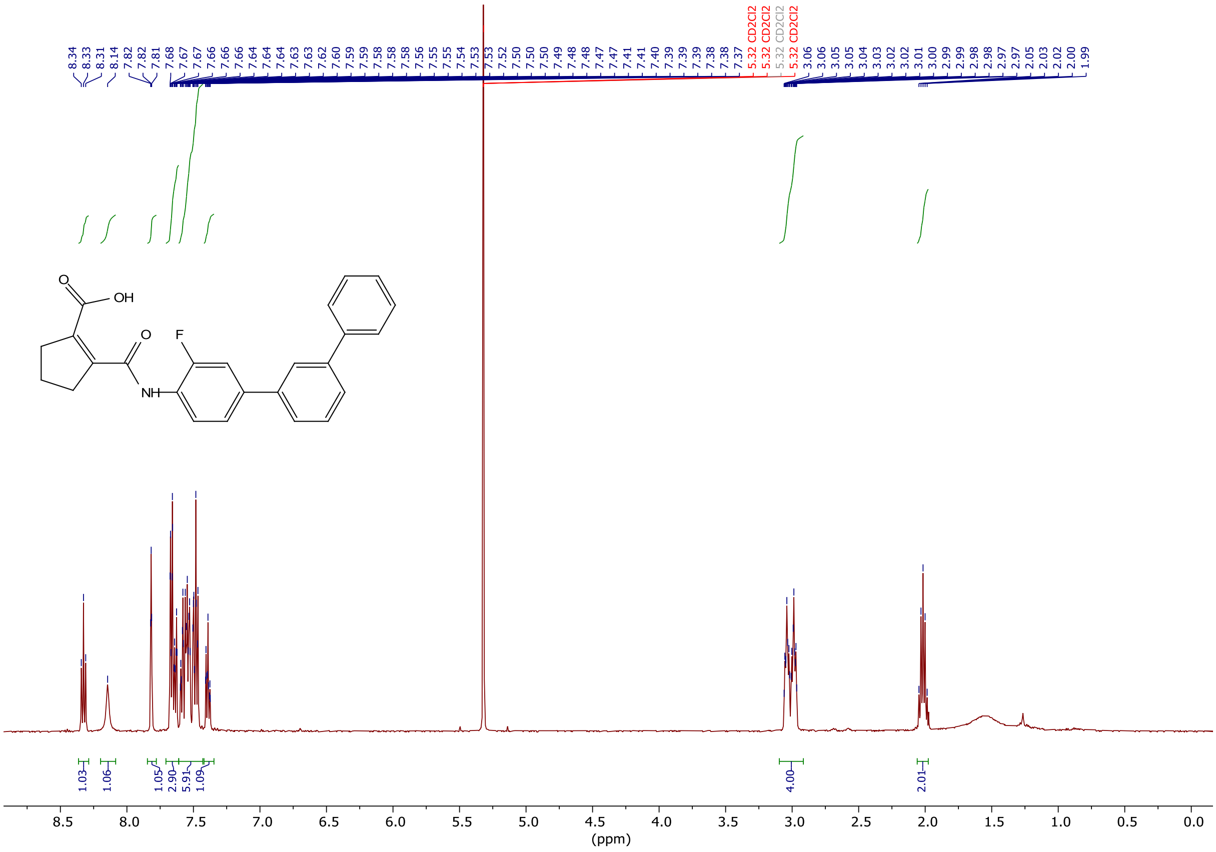

^{1}H NMR (500 MHz, CD_2Cl_2) of **29**

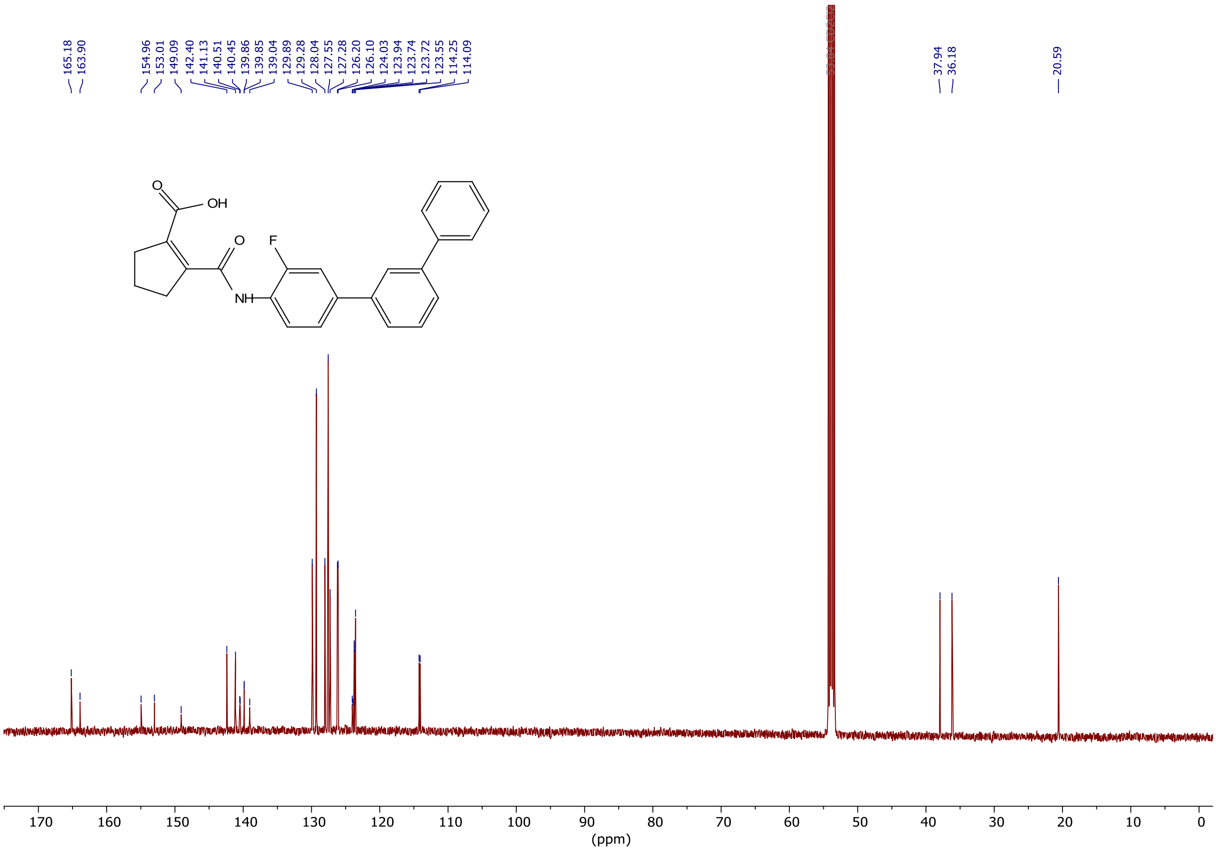

^{13}C NMR (126 MHz, CD_2Cl_2) of **29**

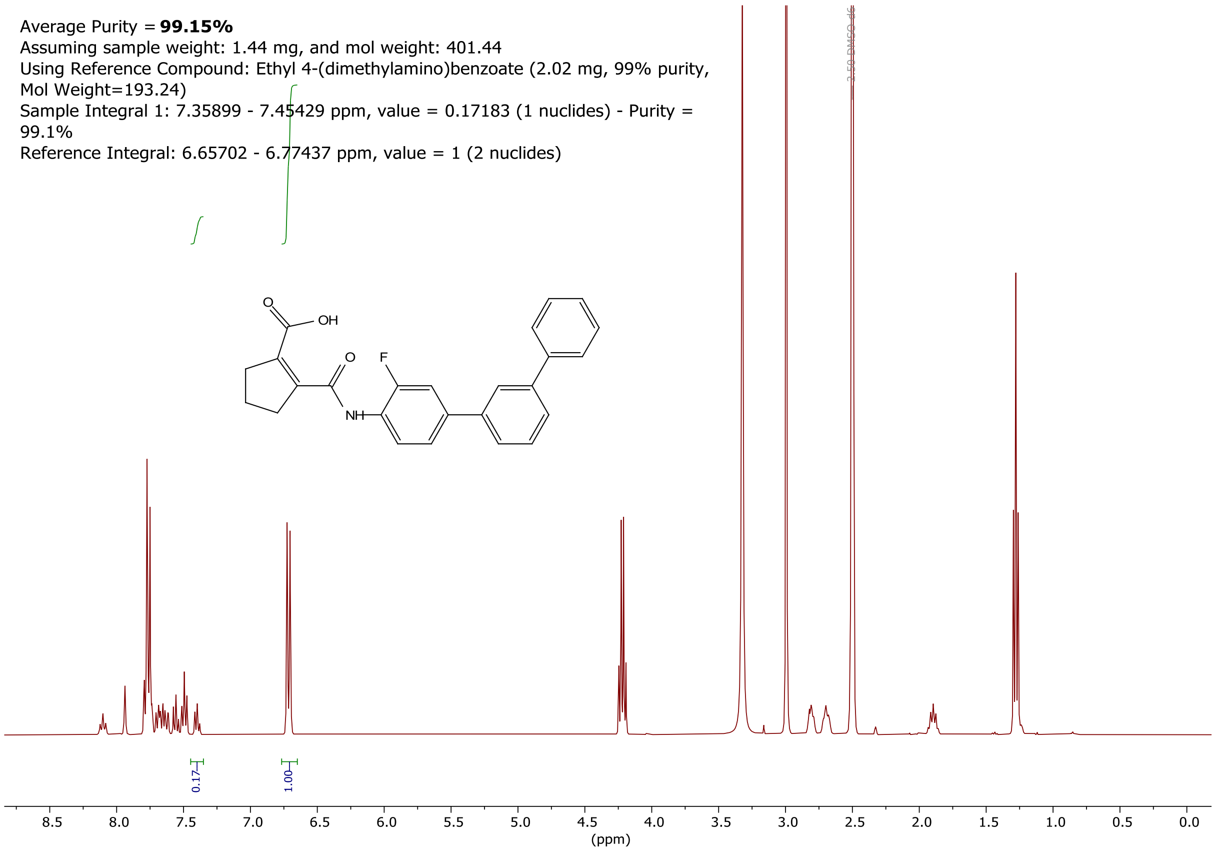

qH NMR (400 MHz, DMSO-d_6) of **29**

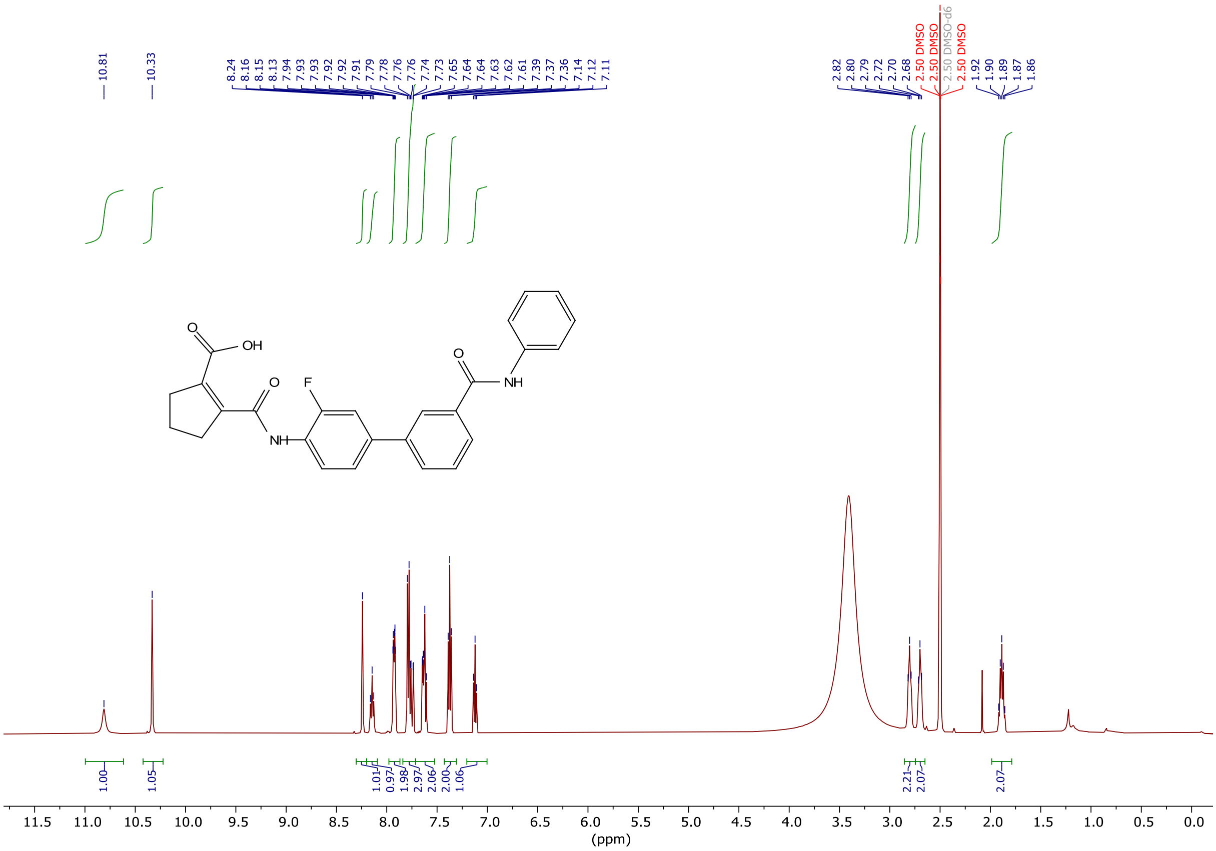

^{1}H NMR (500 MHz, DMSO-d_6) of **30**

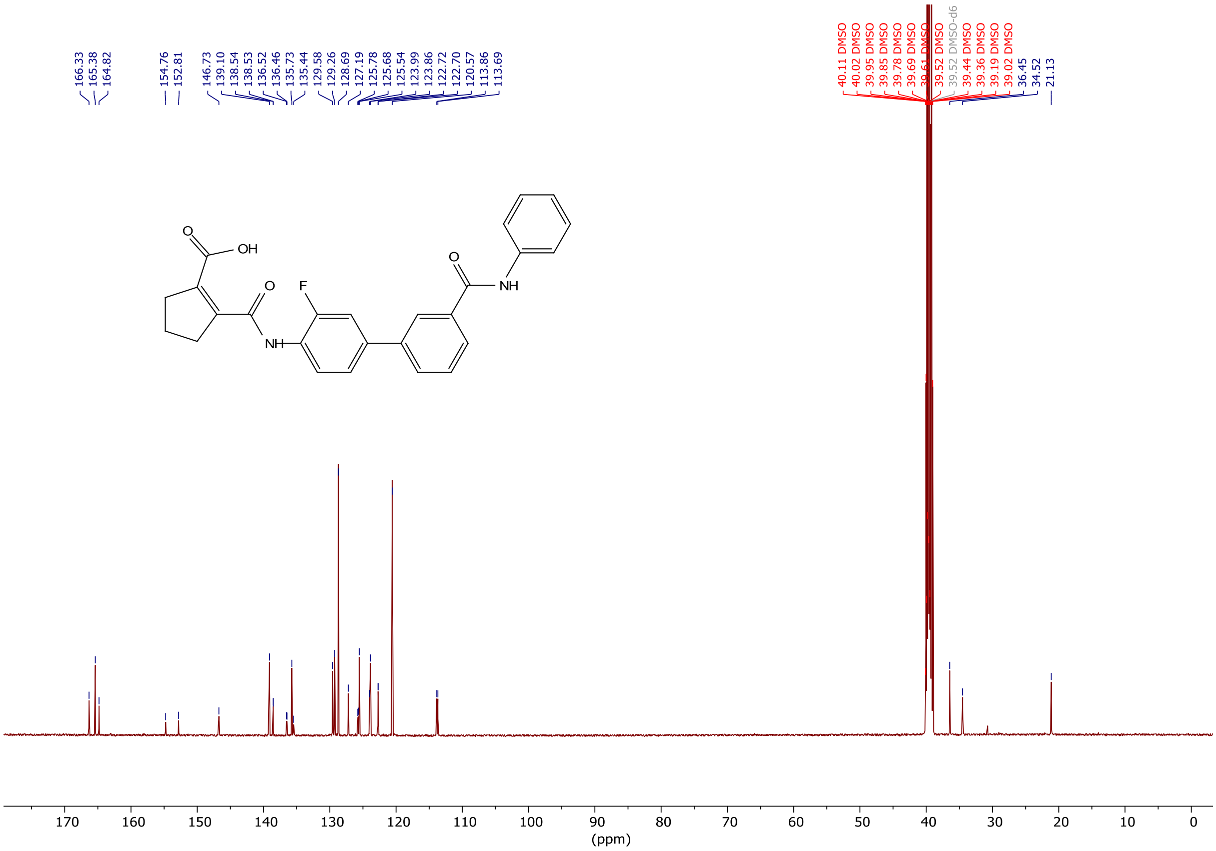

^{13}C NMR (126 MHz, DMSO-d_6) of **30**

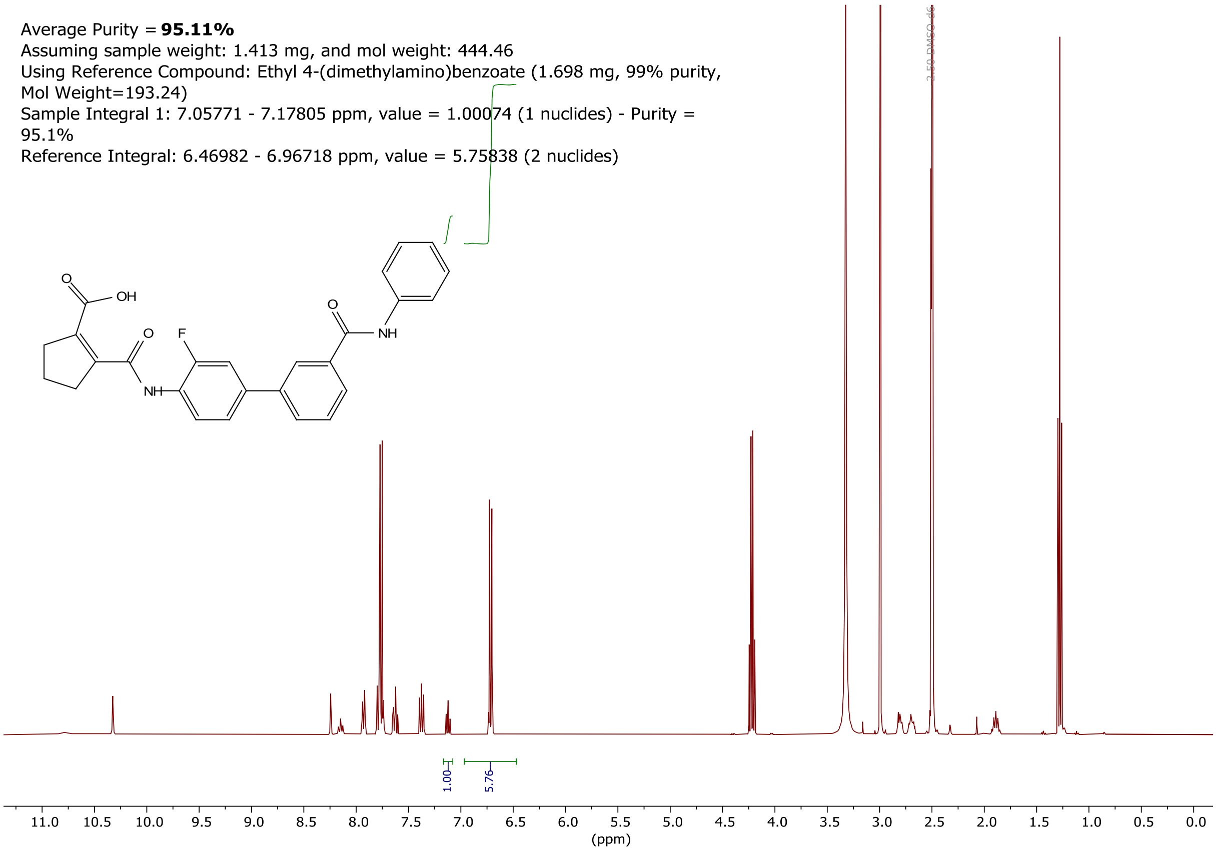

qH NMR (400 MHz, DMSO-d_6) of **30**

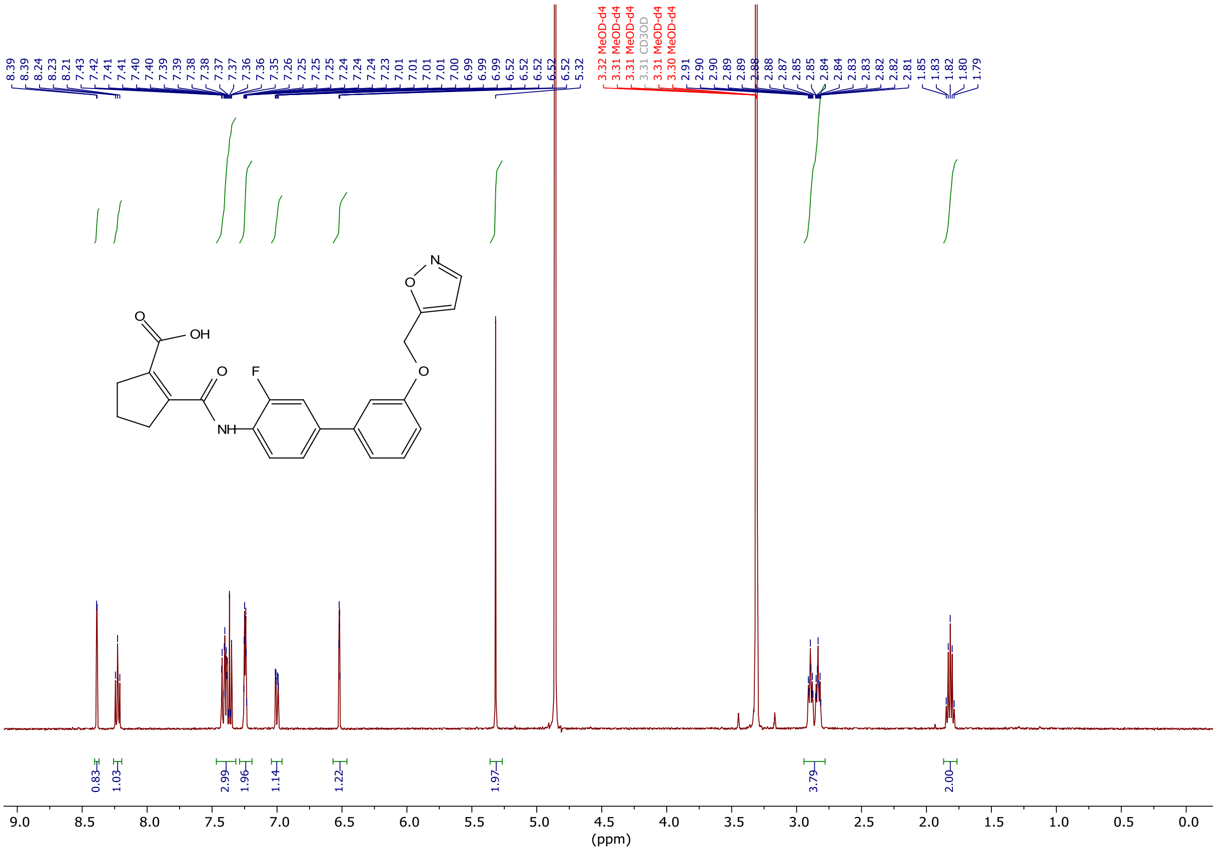

^{1}H NMR (500 MHz, MeOD-d_4) of **31**

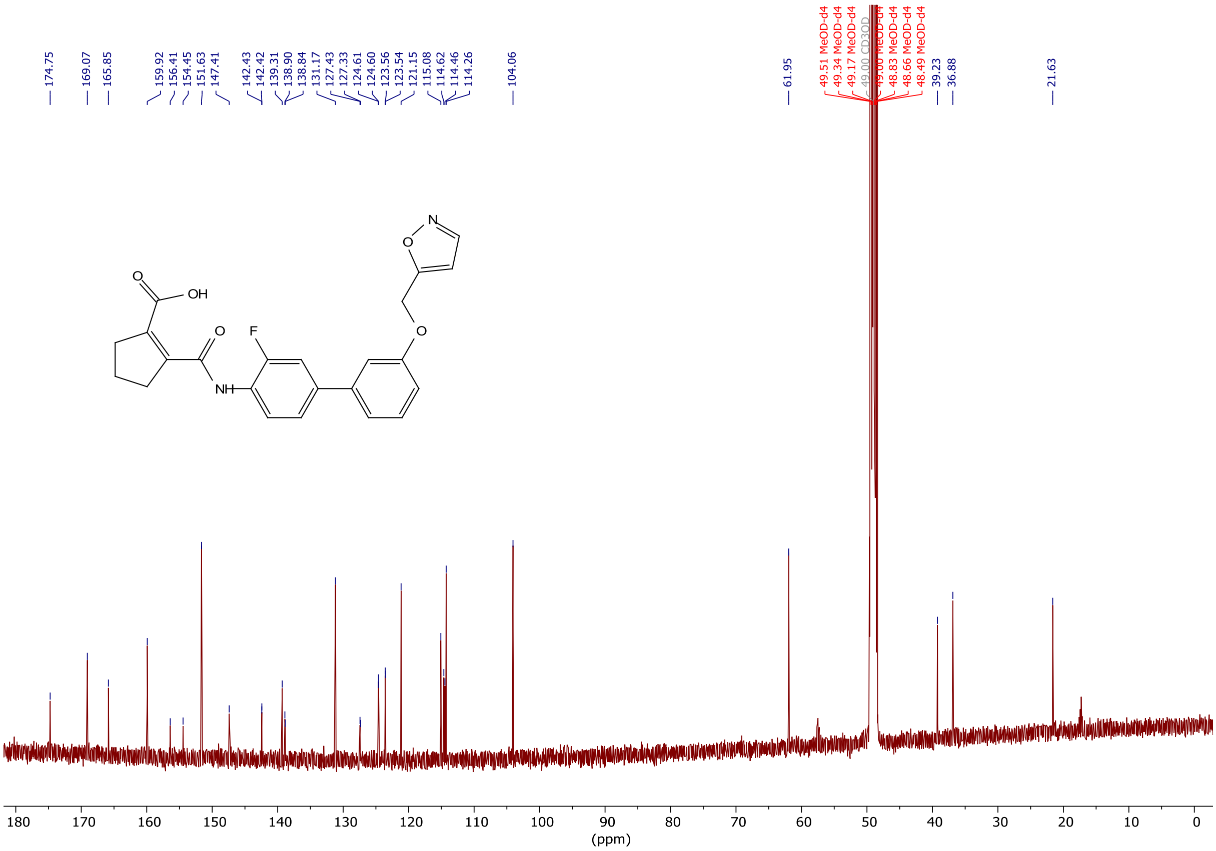

^{13}C NMR (126 MHz, MeOD-d_4) of **31**

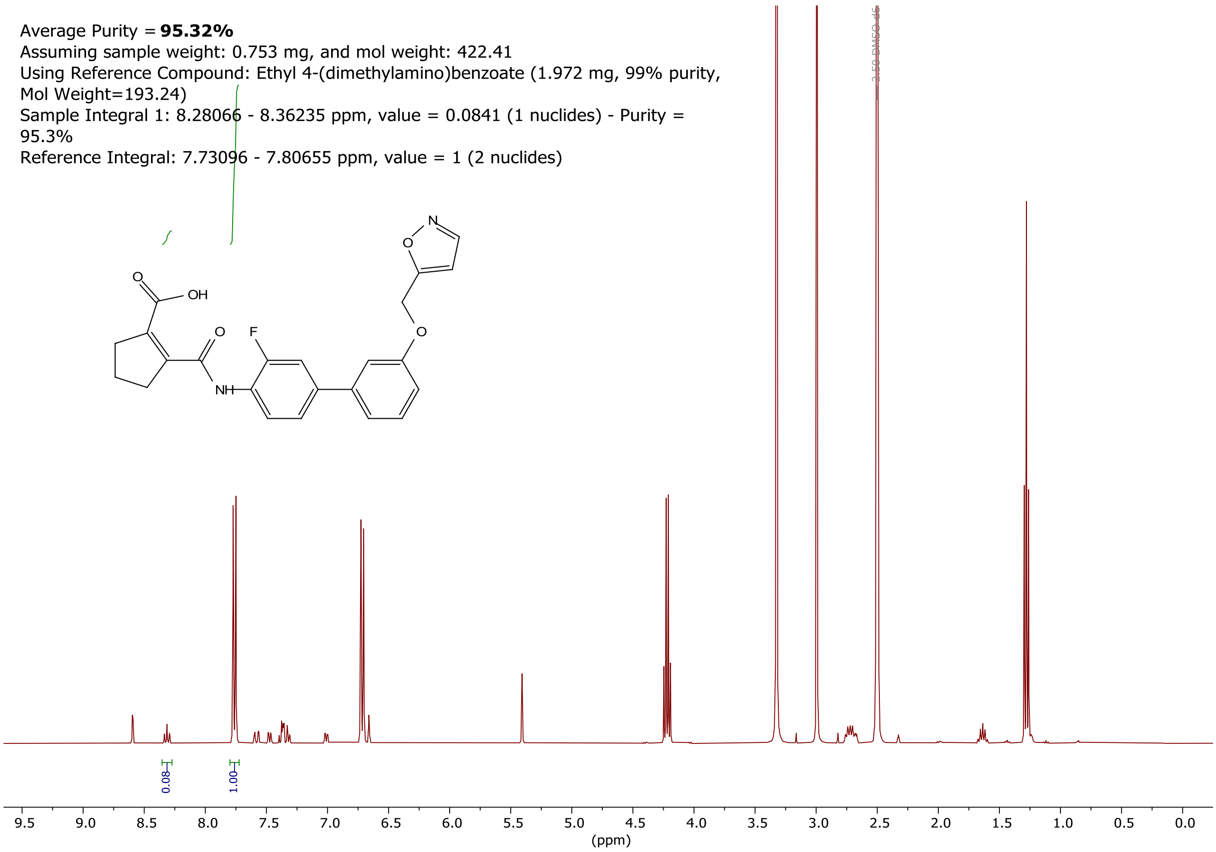

qH NMR (400 MHz, DMSO-d_6) of **31**

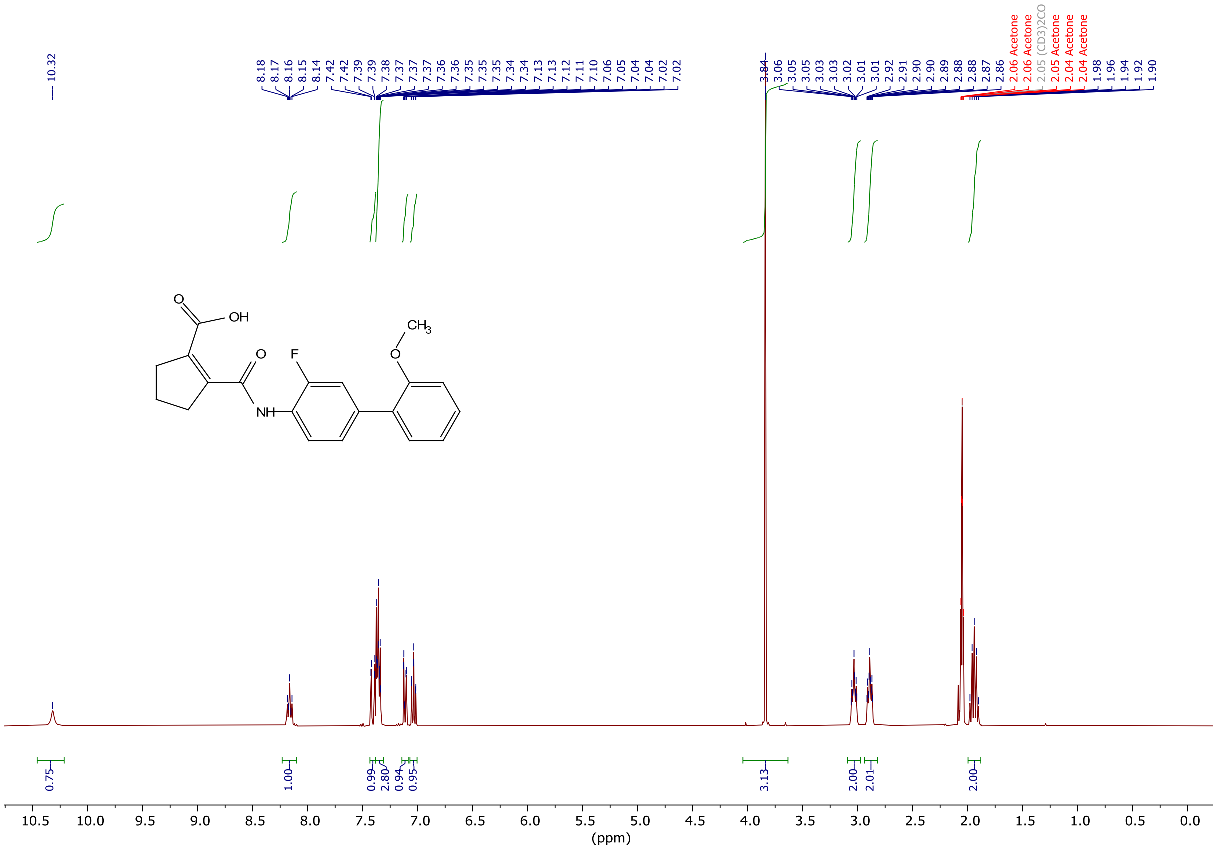

^{1}H NMR (400 MHz, acetone-d_6) of **32**

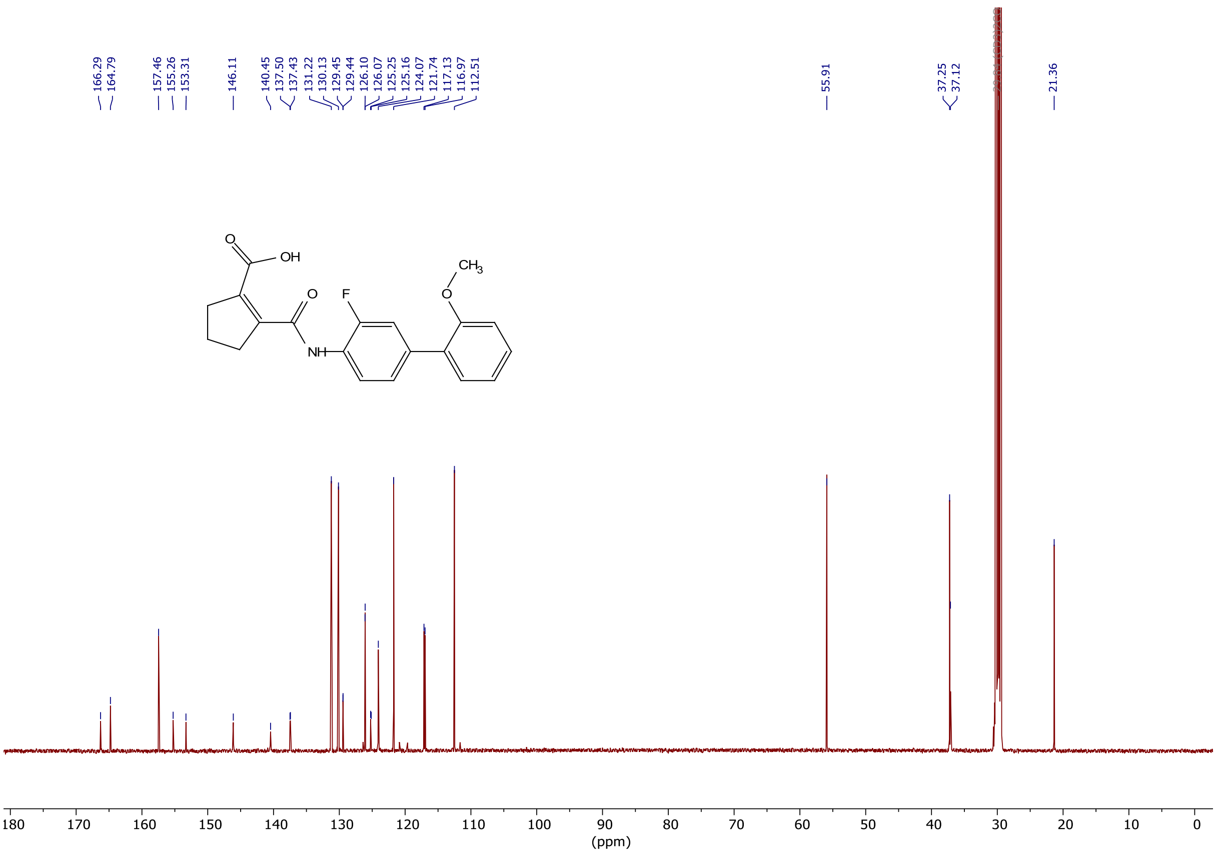

^{13}C NMR (126 MHz, acetone-d_6) of **32**

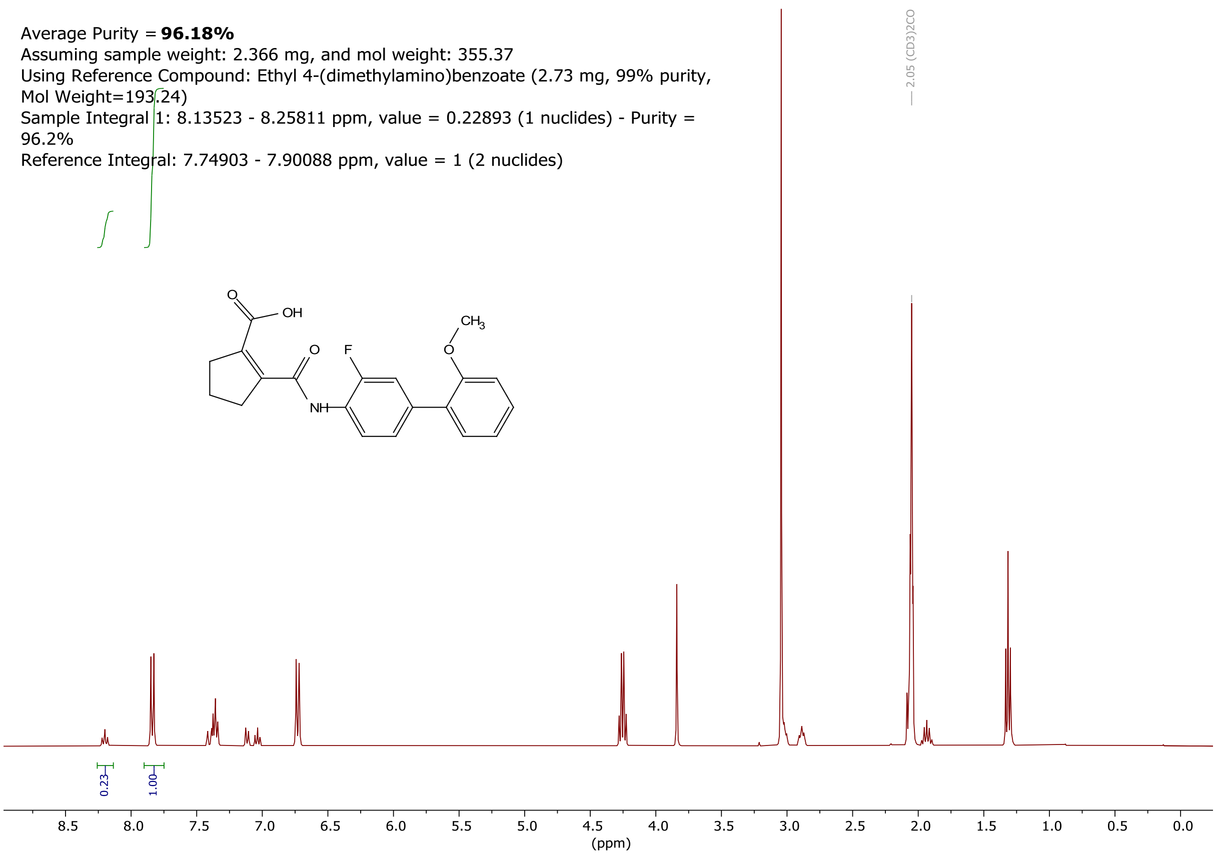

qH NMR (400 MHz, acetone-d_6) of **32**

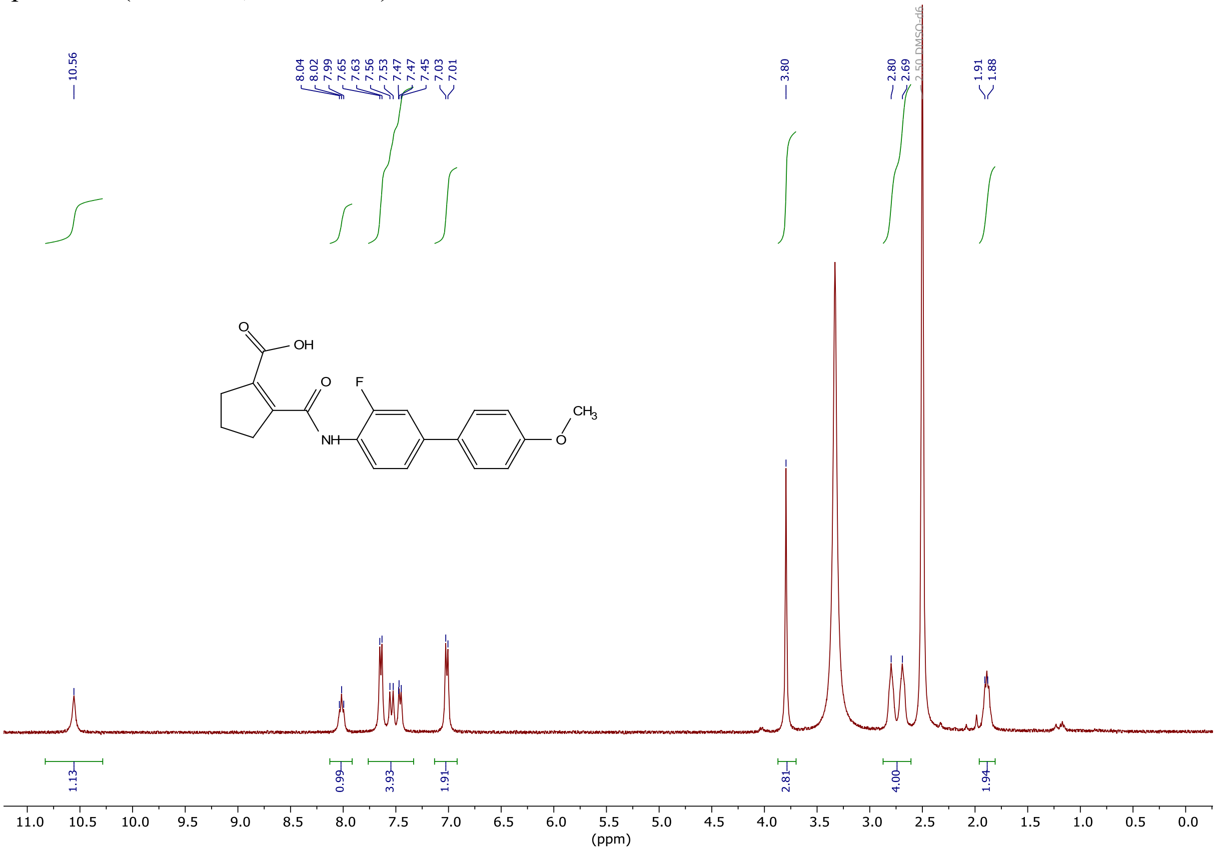

^{1}H NMR (400 MHz, DMSO-d_6) of **33**

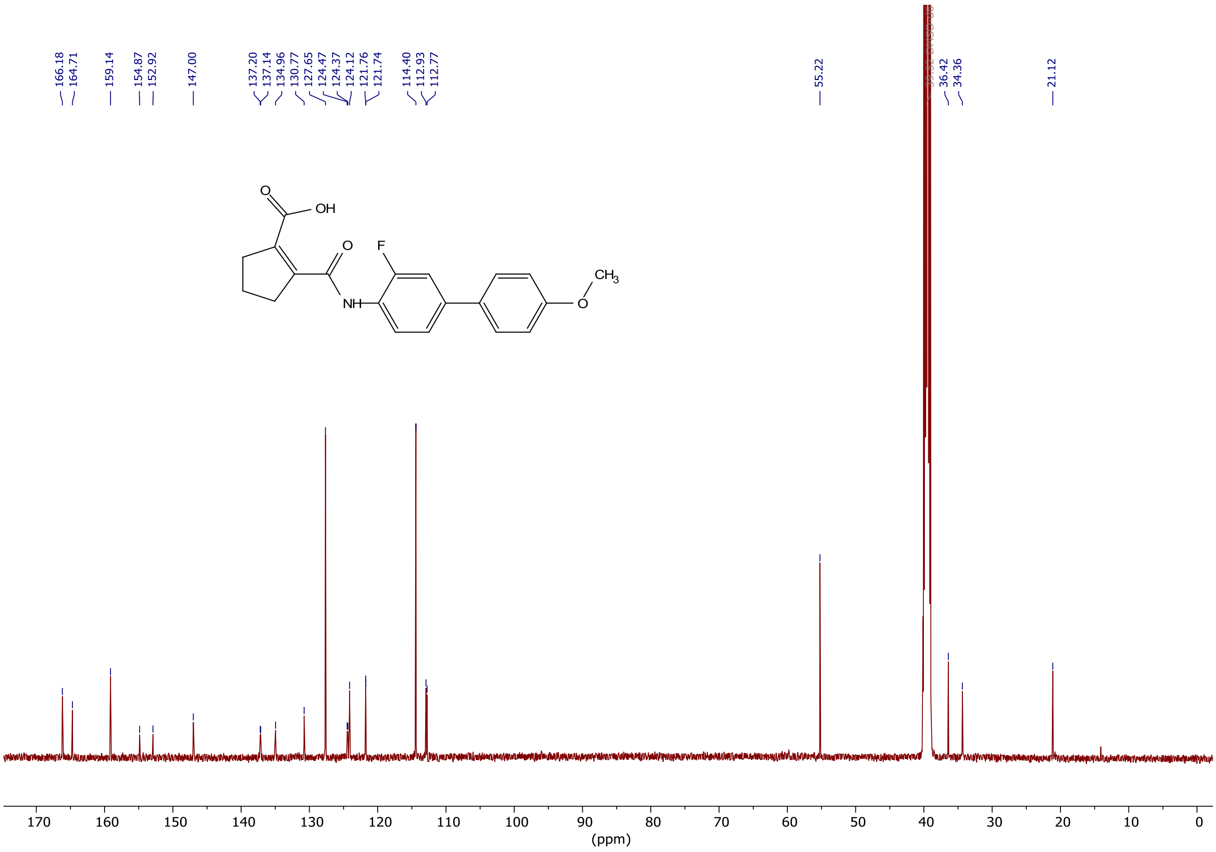

^{13}C NMR (126 MHz, DMSO-d_6) of **33**

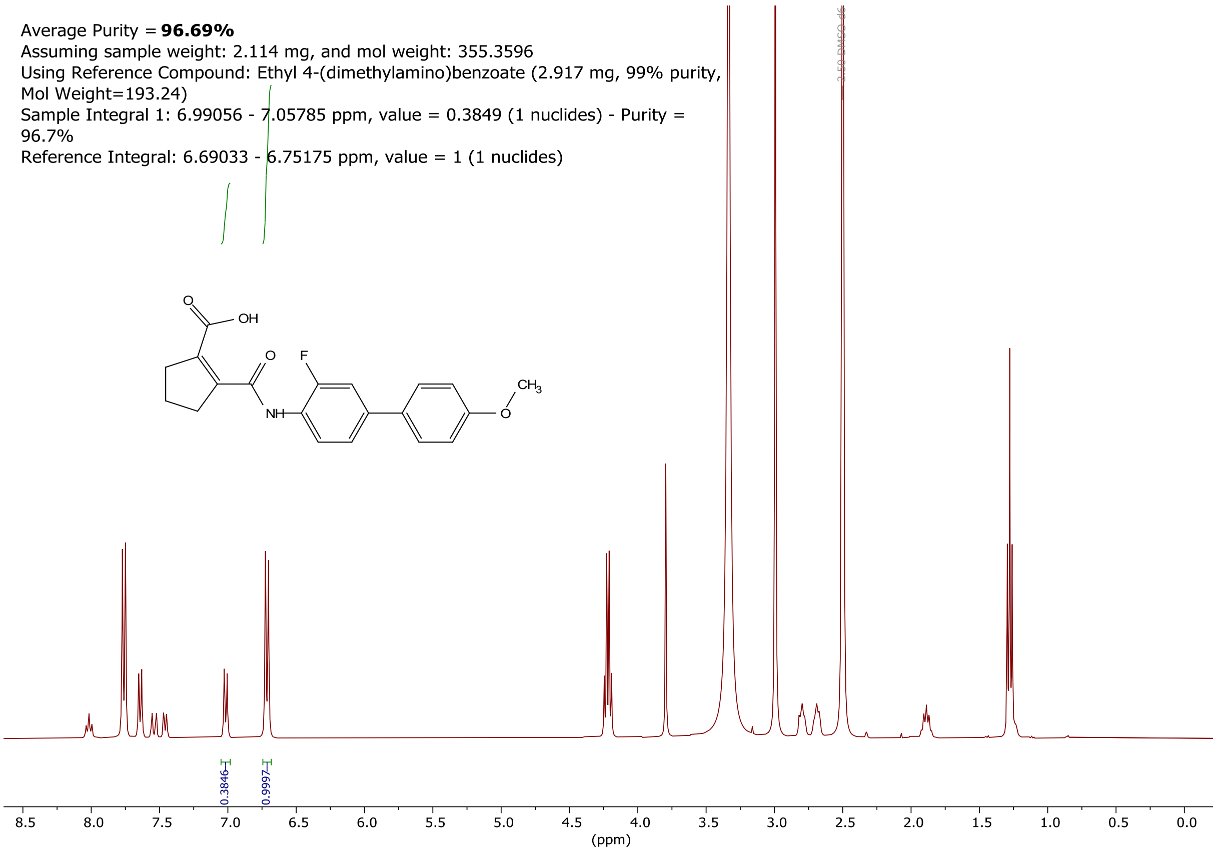

qH NMR (400 MHz, DMSO-d_6) of **33**

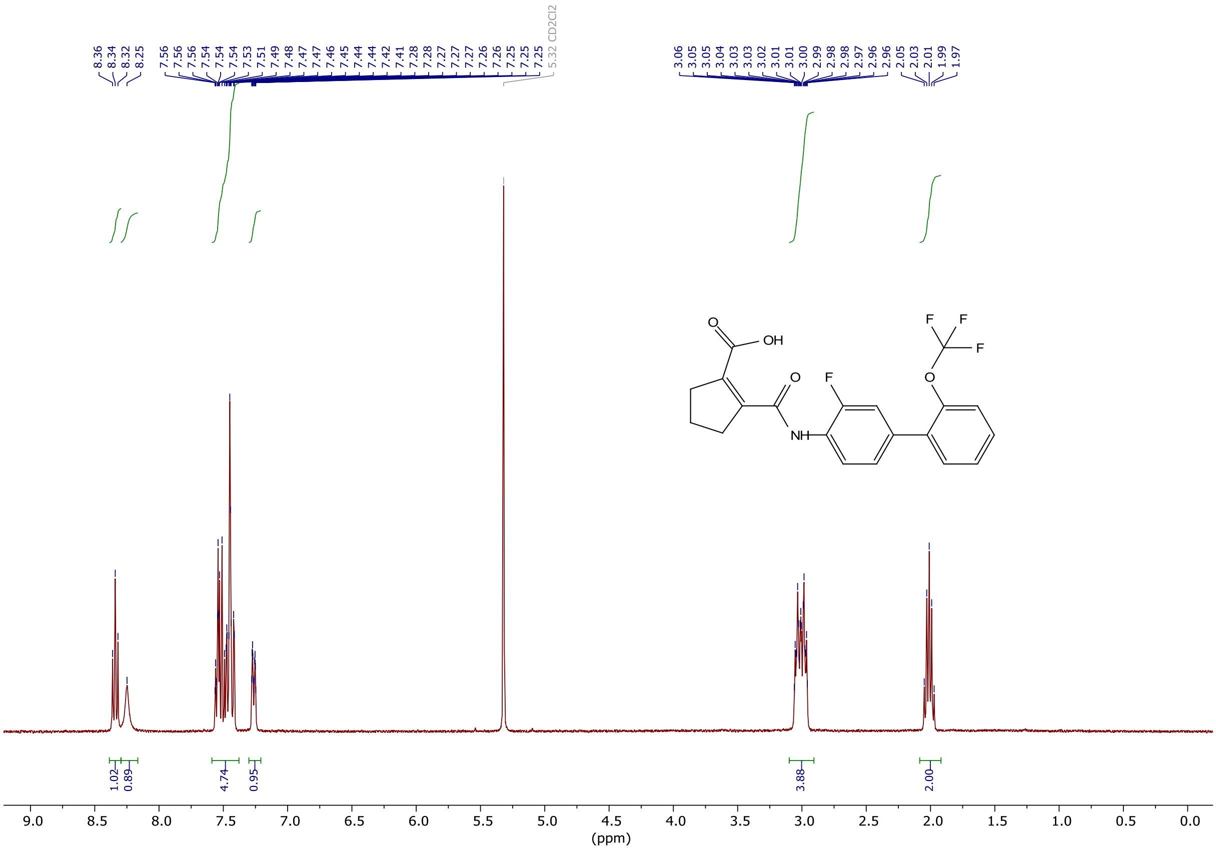

^{1}H NMR (400 MHz, CD_2Cl_2) of **34**

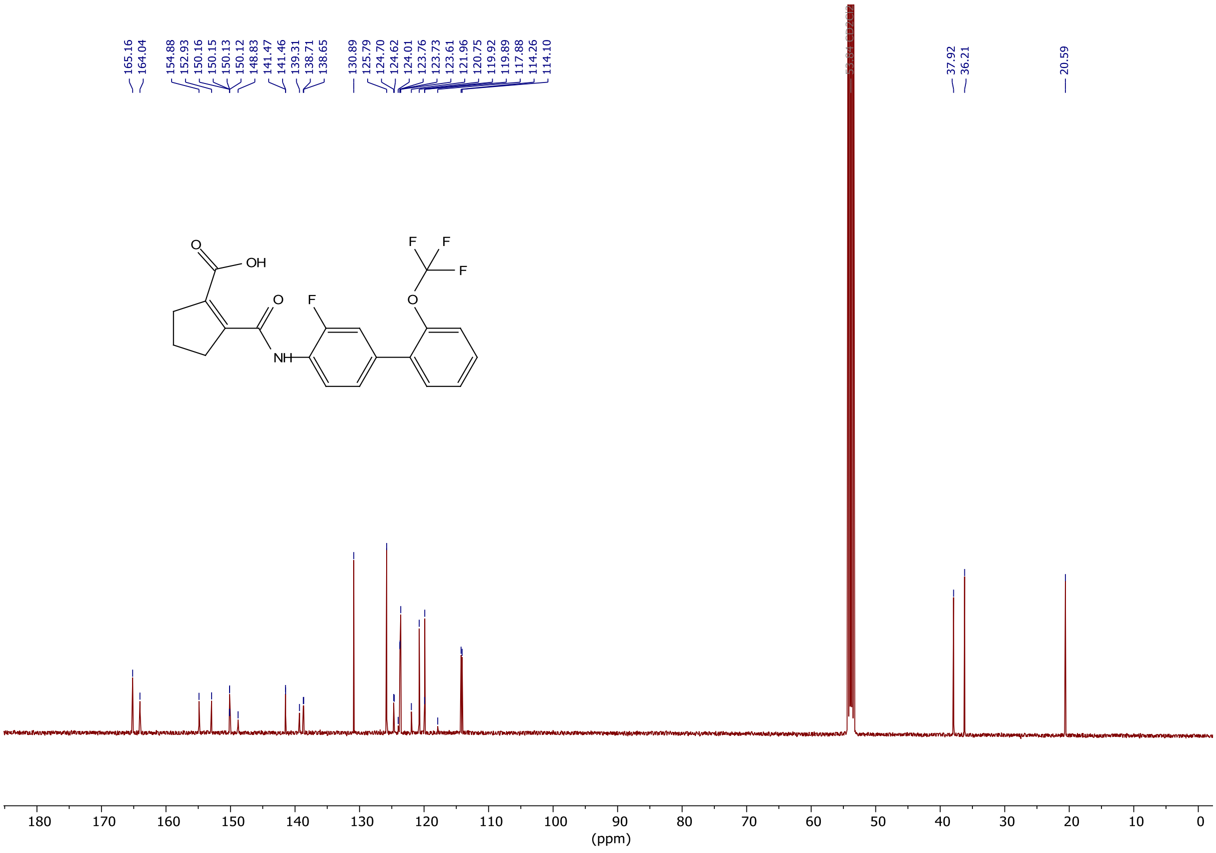

^{13}C NMR (126 MHz, CD_2Cl_2) of **34**

Average Purity = **96.11%**
Assuming sample weight: 1.29 mg, and mol weight: 409.34
Using Reference Compound: Ethyl 4-(dimethylamino)benzoate (1.526 mg, 99% purity, Mol Weight=193.24)
Sample Integral 1: 7.32199 - 7.41627 ppm, value = 0.1937 (1 nuclides) - Purity = 96.1%
Reference Integral: 6.63891 - 6.77861 ppm, value = 1 (2 nuclides)

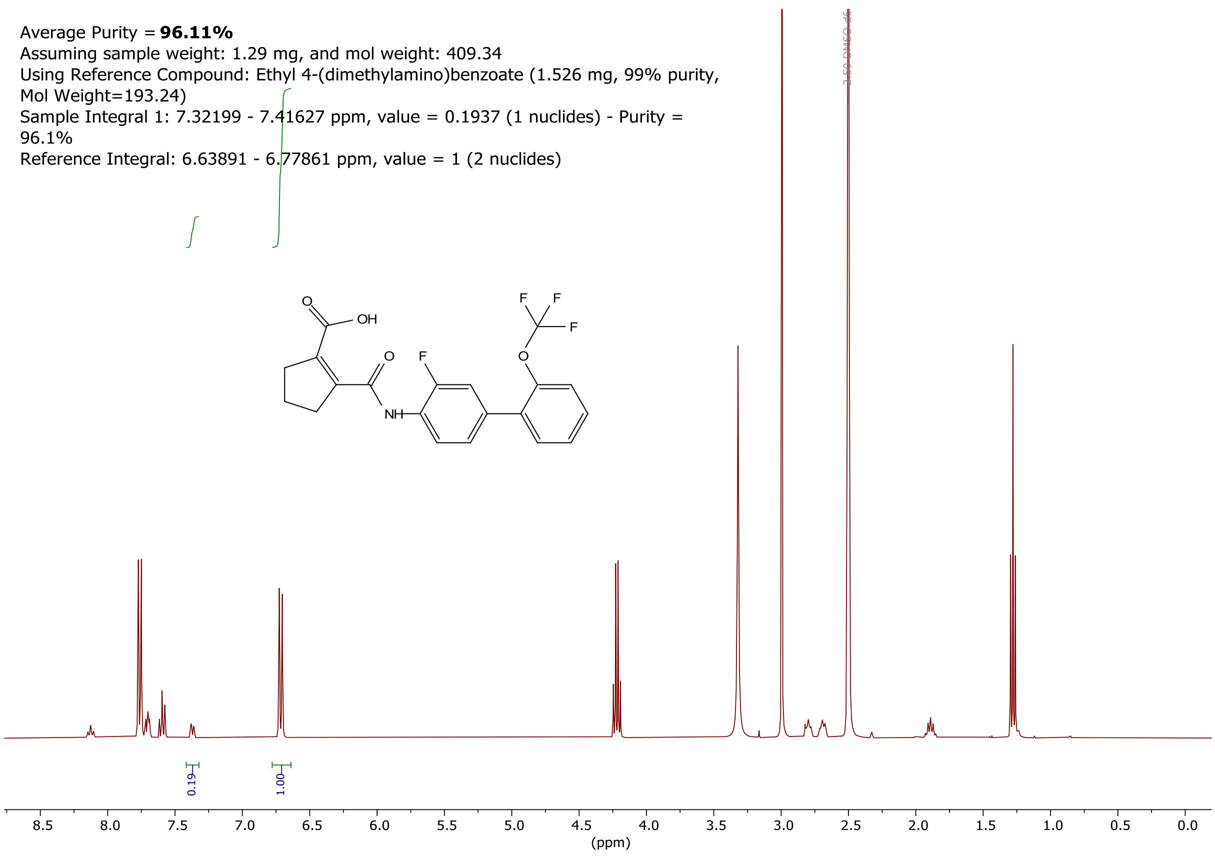

qH NMR (400 MHz, DMSO-d_6) of **34**

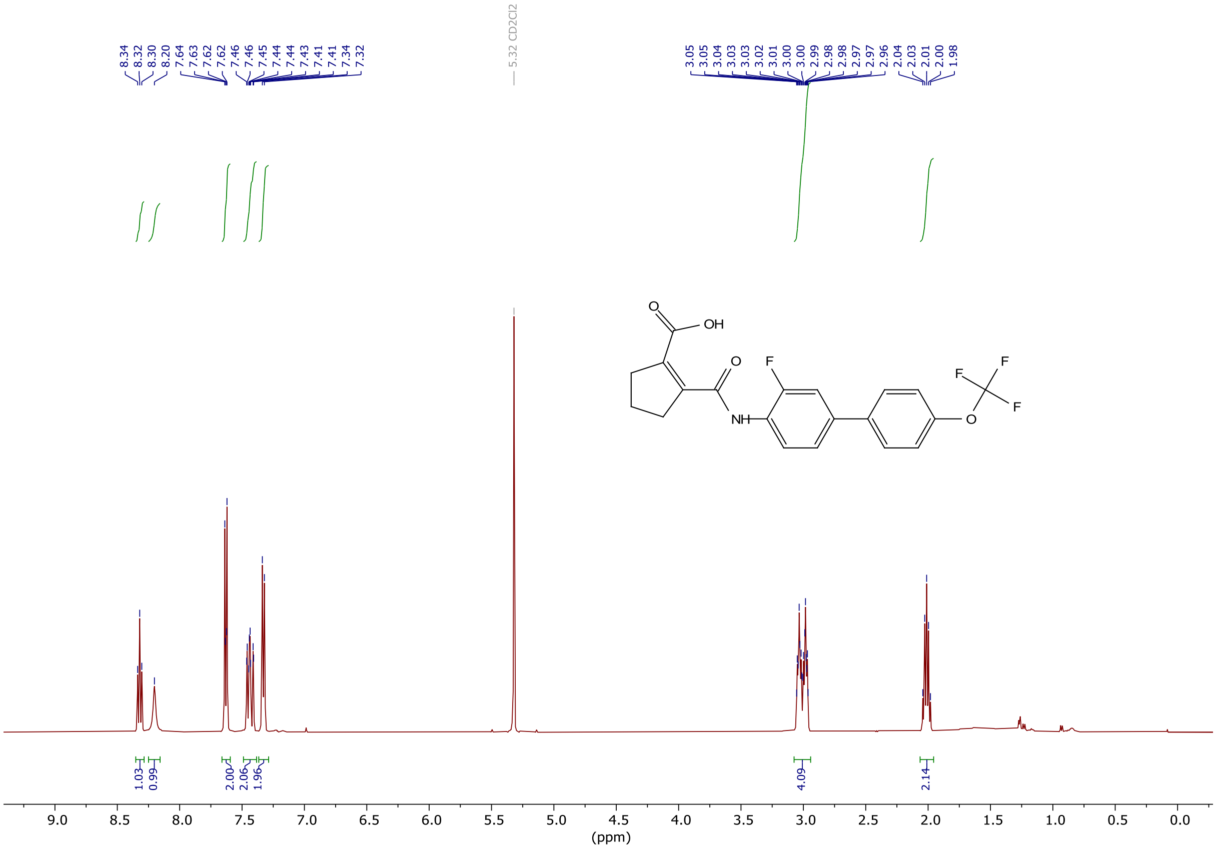

^{1}H NMR (500 MHz, CD_2Cl_2) of **35**

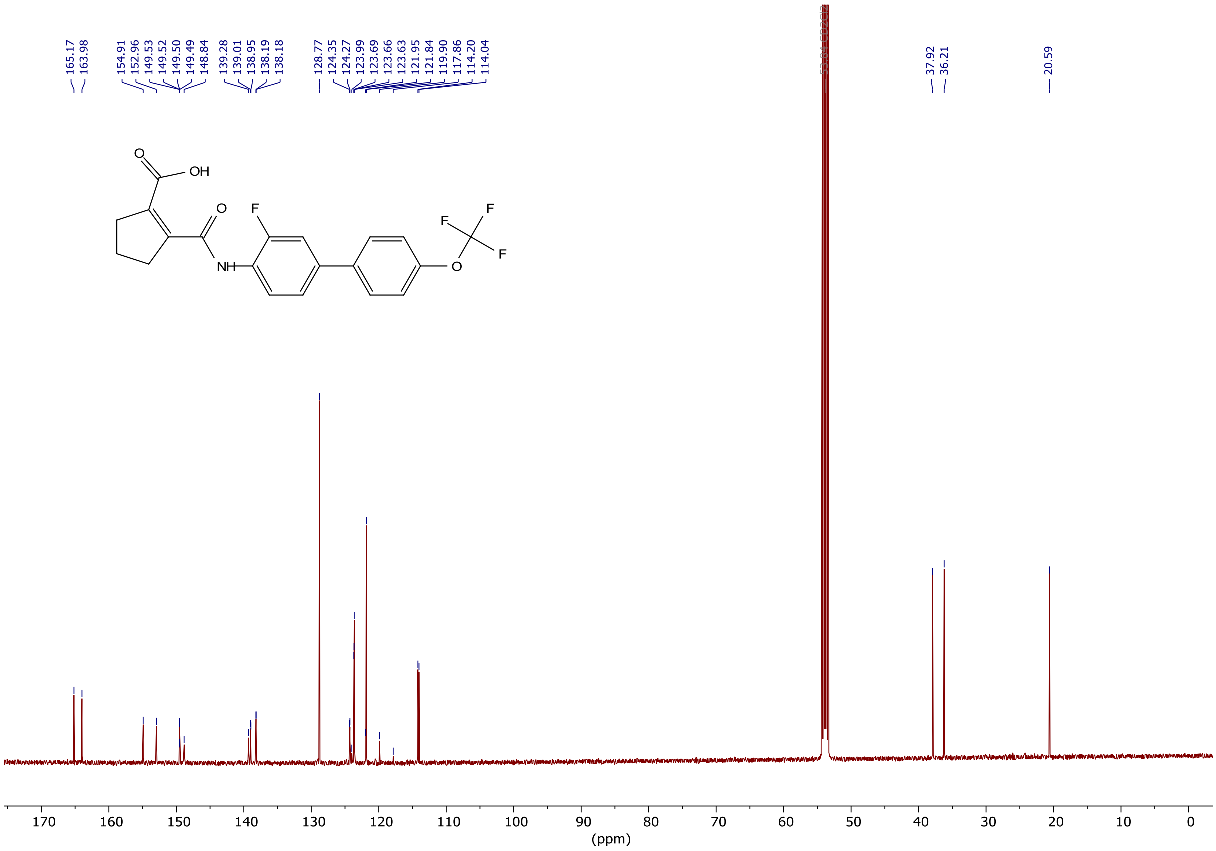

^{13}C NMR (126 MHz, CD_2Cl_2) of **35**

Average Purity = **95.19%**
Assuming sample weight: 1.108 mg, and mol weight: 409.34
Using Reference Compound: Ethyl 4-(dimethylamino)benzoate (1.022 mg, 99% purity, Mol Weight=193.24)
Sample Integral 1: 7.79339 - 7.86772 ppm, value = 0.4921 (2 nuclides) - Purity = 95.2%
Reference Integral: 7.72462 - 7.7914 ppm, value = 1 (2 nuclides)

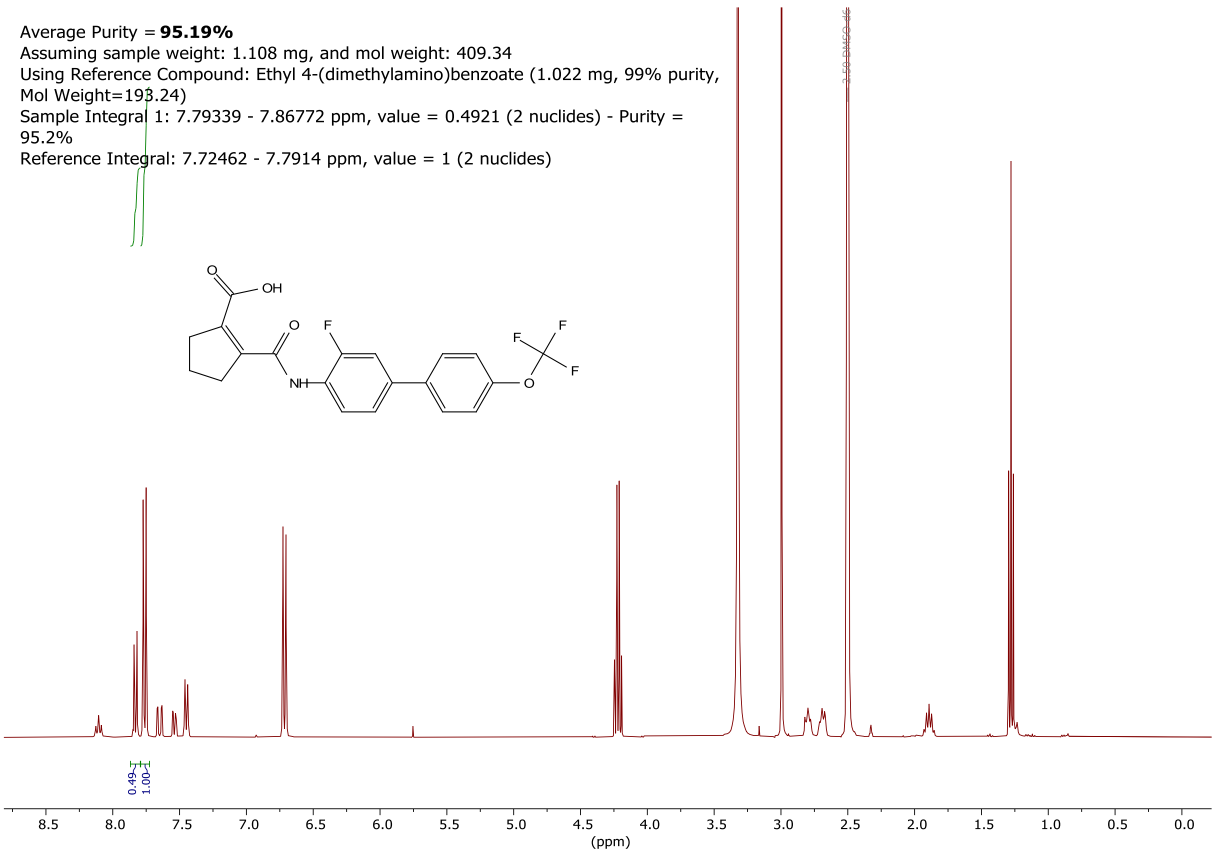

qH NMR (400 MHz, DMSO-d_6) of **35**

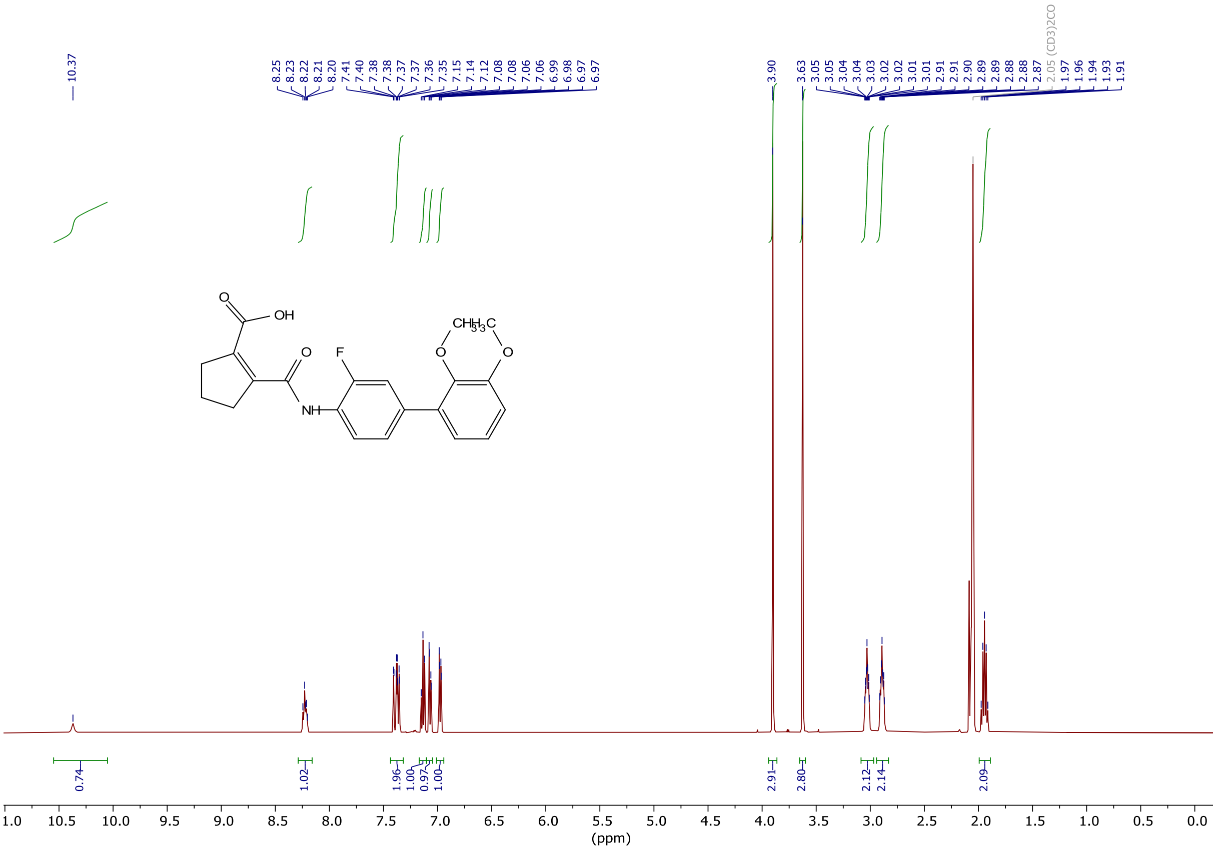

^{1}H NMR (500 MHz, acetone-d_6) of **36**

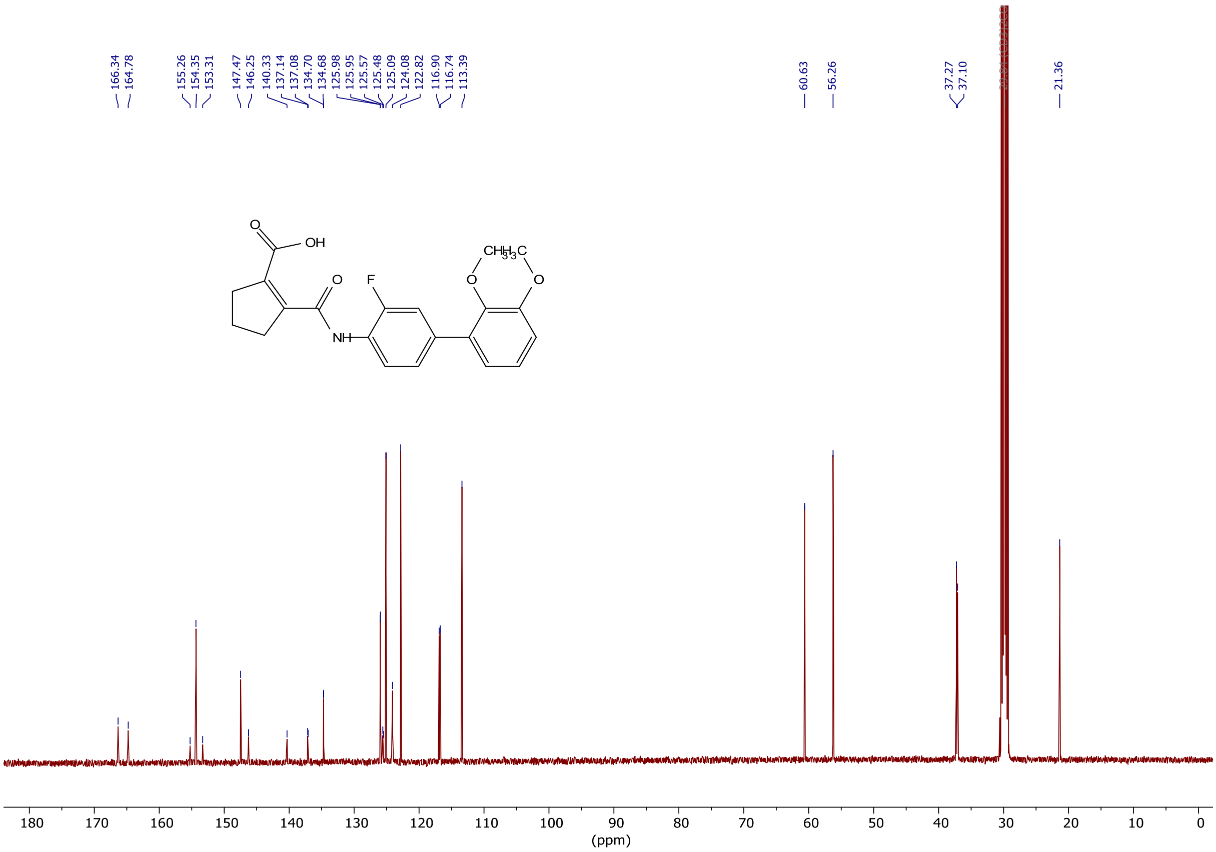

^{13}C NMR (126 MHz, acetone-d_6) of **36**

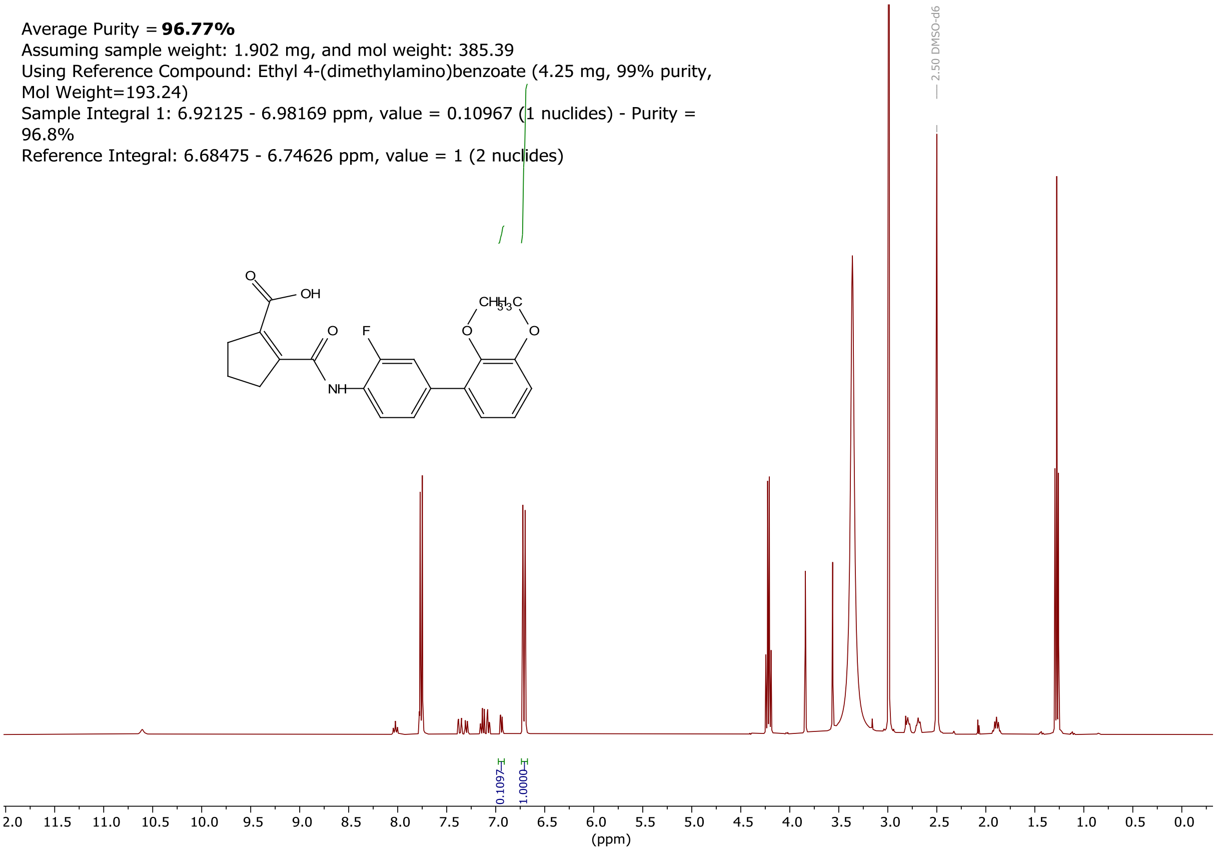

qH NMR (400 MHz, DMSO-d_6) of **36**

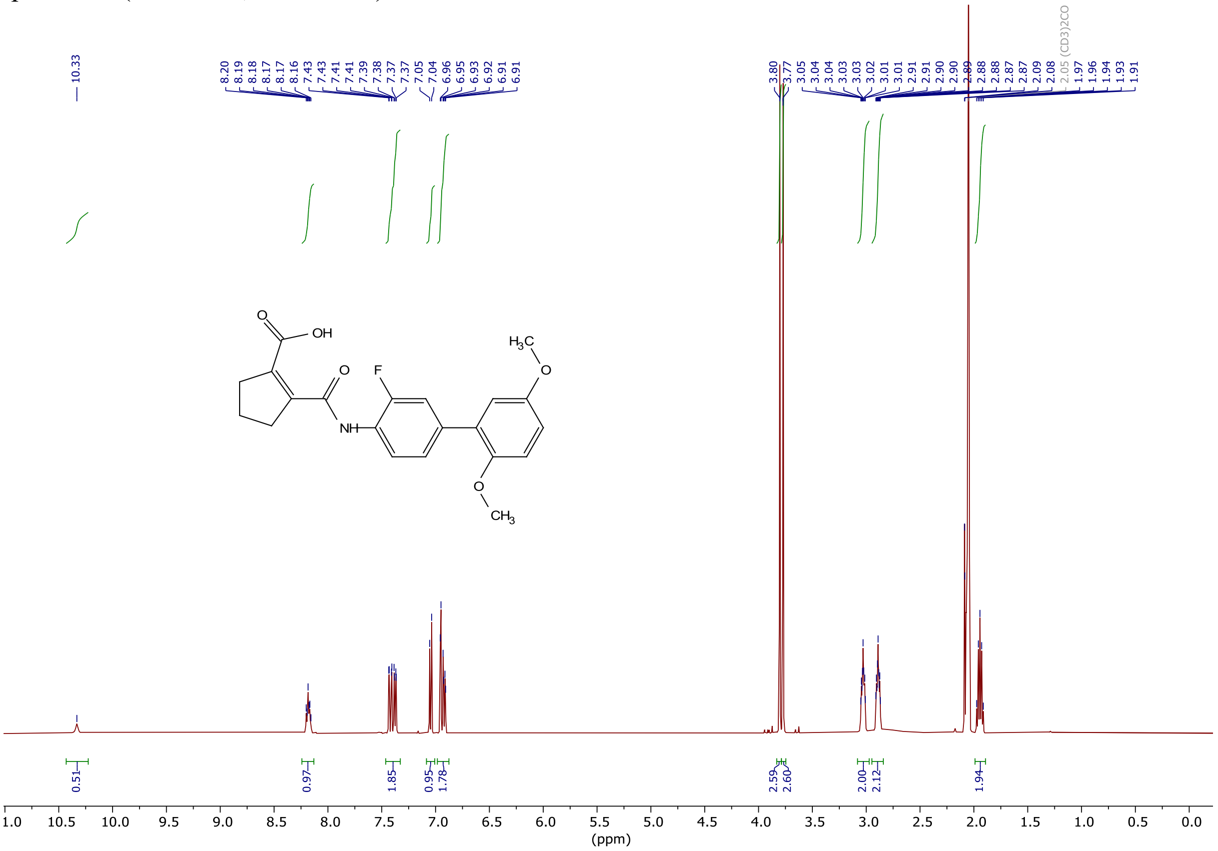

^{1}H NMR (500 MHz, acetone-d_6) of **37**

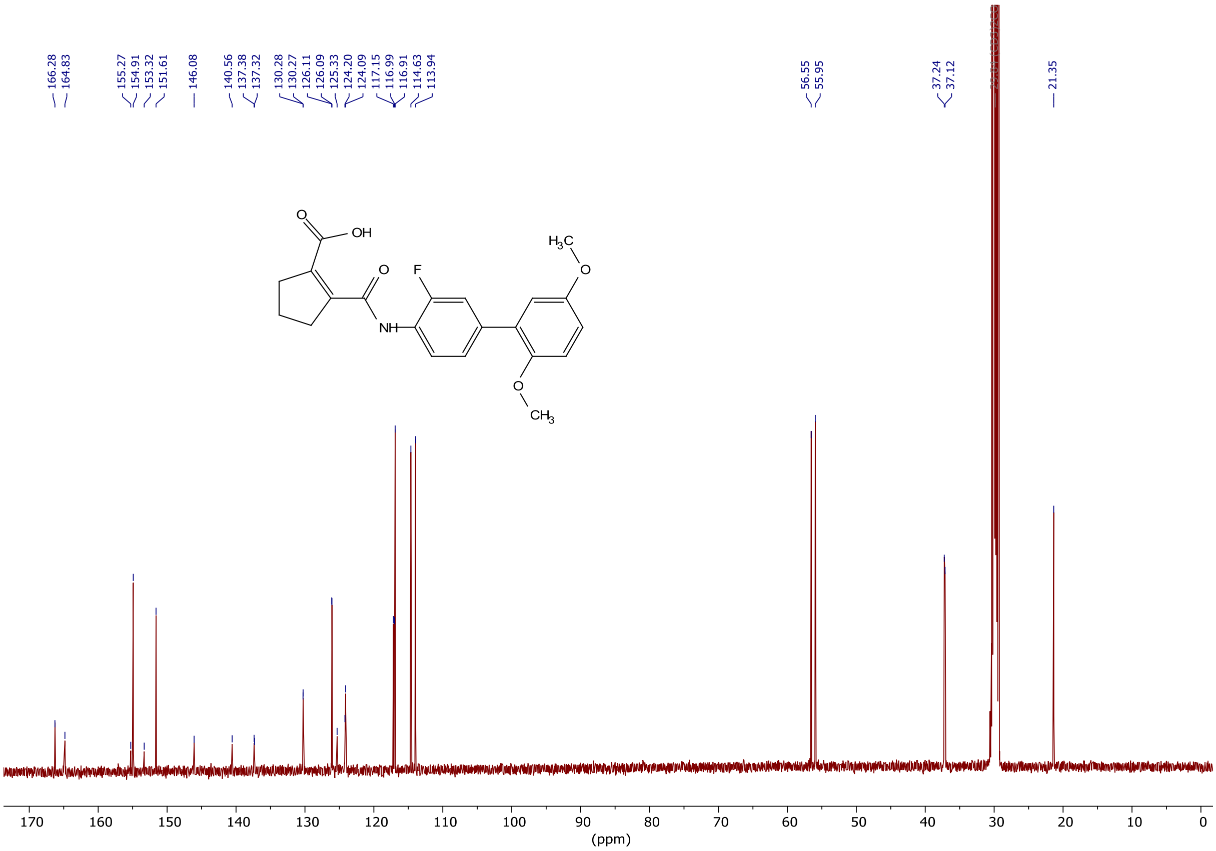

^{13}C NMR (126 MHz, acetone-d_6) of **37**

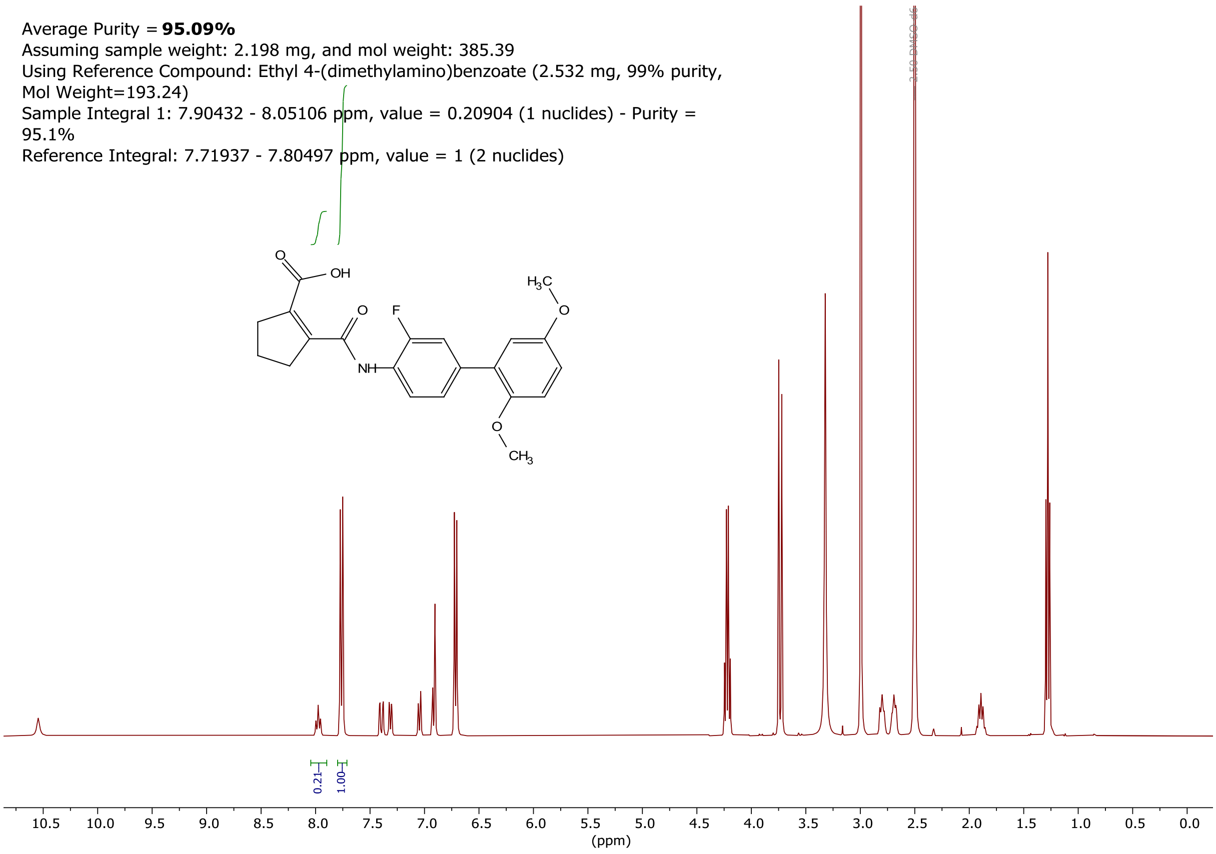

qH NMR (400 MHz, DMSO-d_6) of **37**

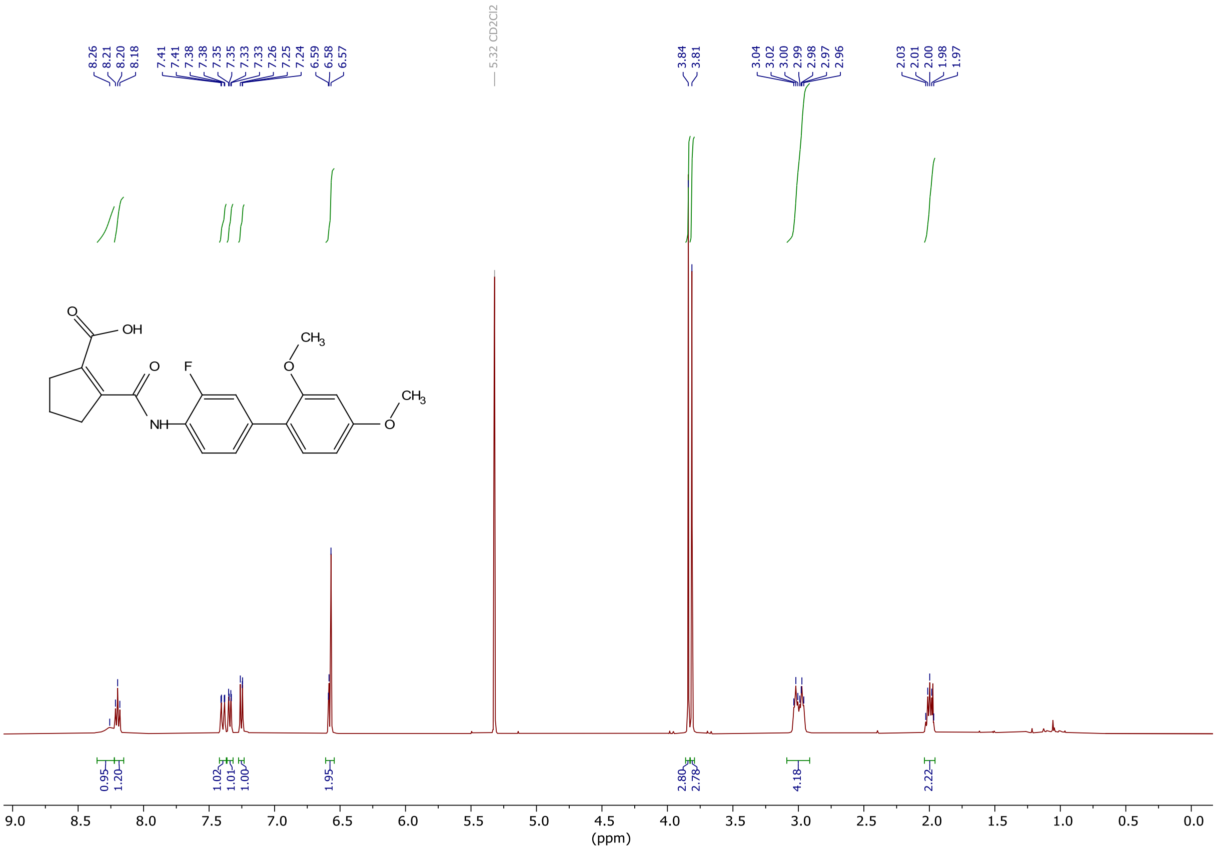

^{1}H NMR (500 MHz, CD_2Cl_2) of **38**

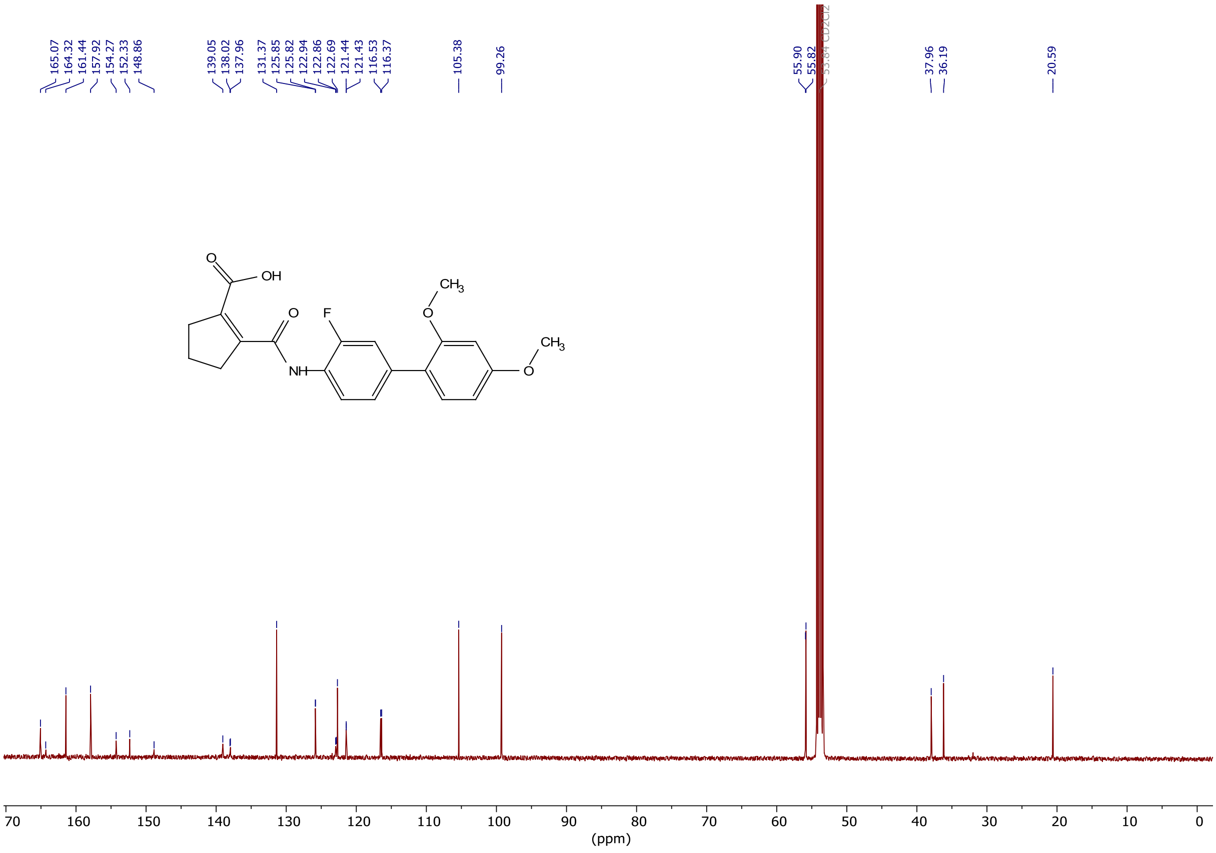

^{13}C NMR (126 MHz, CD_2Cl_2) of **38**

Average Purity = **95.29%**
Assuming sample weight: 1.409 mg, and mol weight: 385.39
Using Reference Compound: Ethyl 4-(dimethylamino)benzoate (1.882 mg, 99% purity, Mol Weight=193.24)
Sample Integral 1: 7.89746 - 7.98056 ppm, value = 0.18067 (1 nuclides) - Purity = 95.3%
Reference Integral: 7.74279 - 7.78005 ppm, value = 1 (2 nuclides)

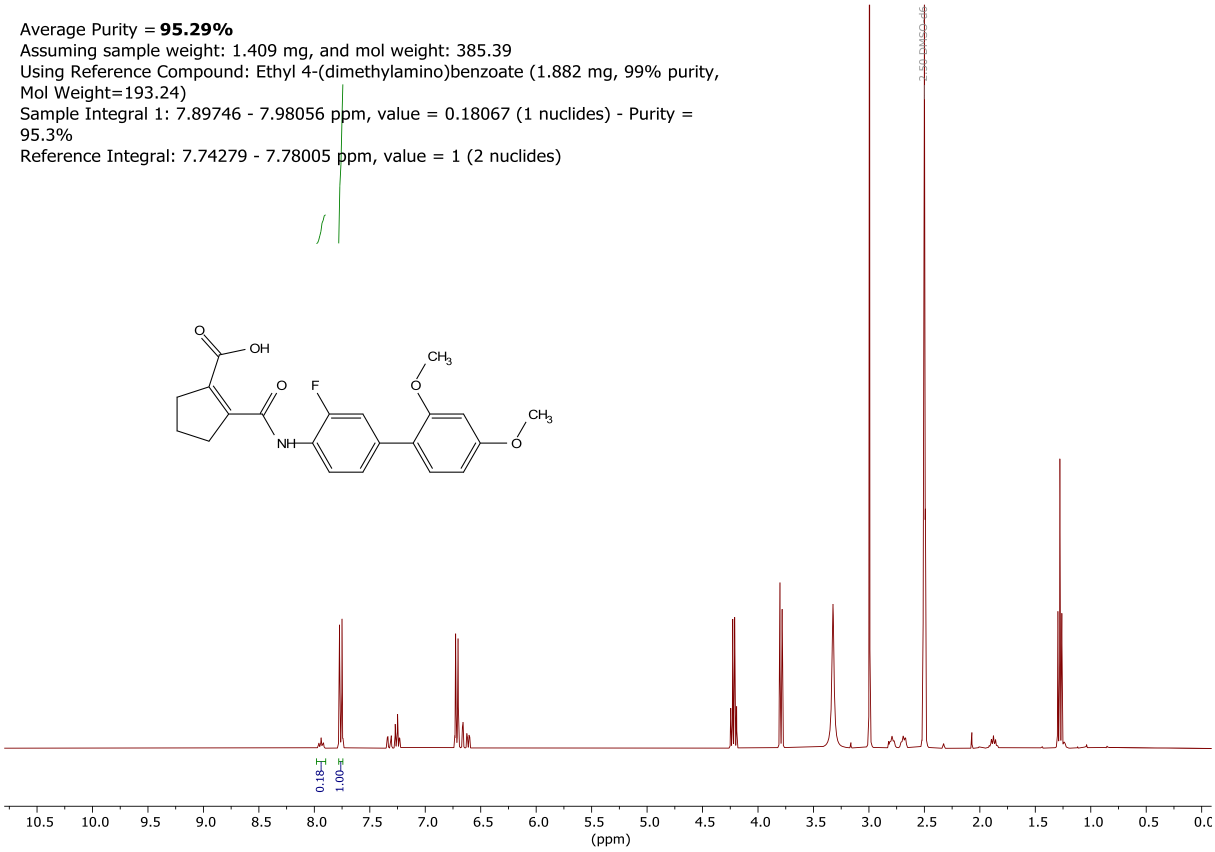

qH NMR (400 MHz, DMSO-d_6) of **38**

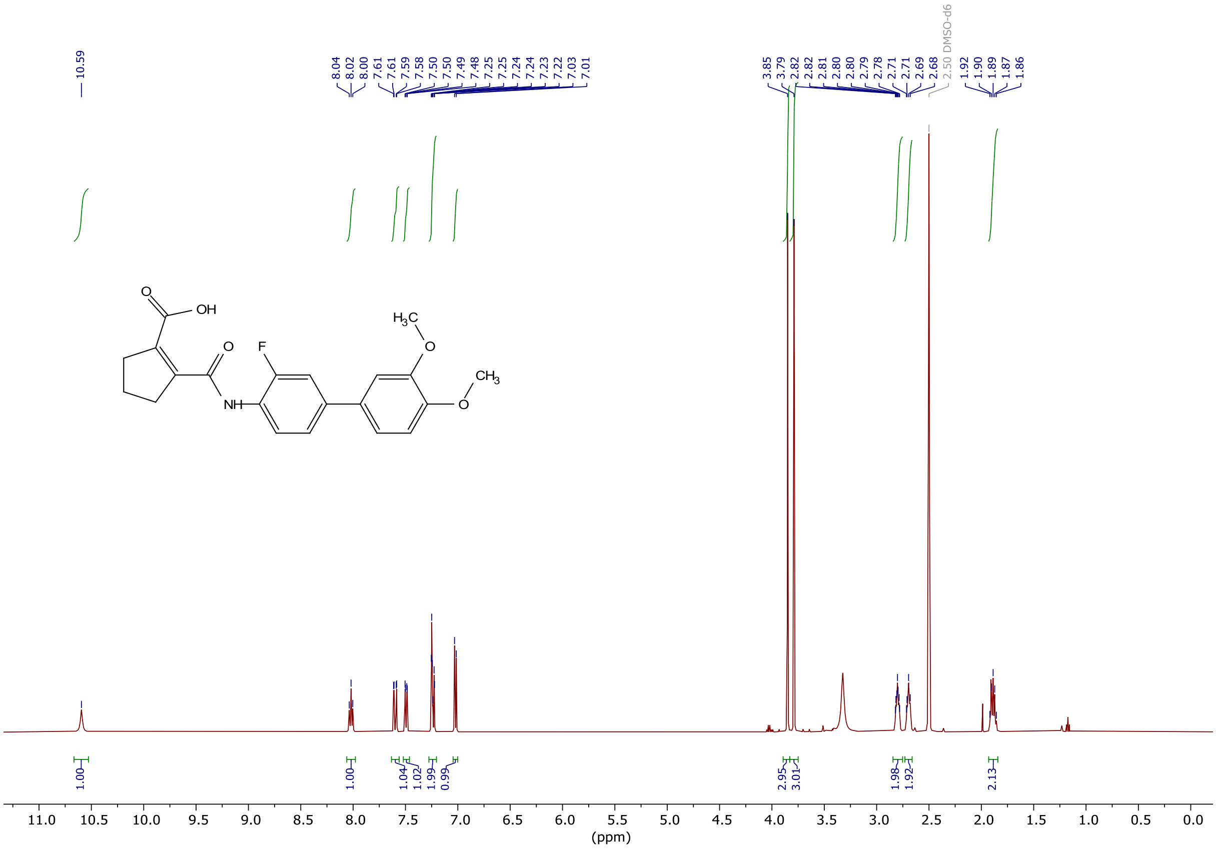

^{1}H NMR (500 MHz, DMSO-d_6) of **39**

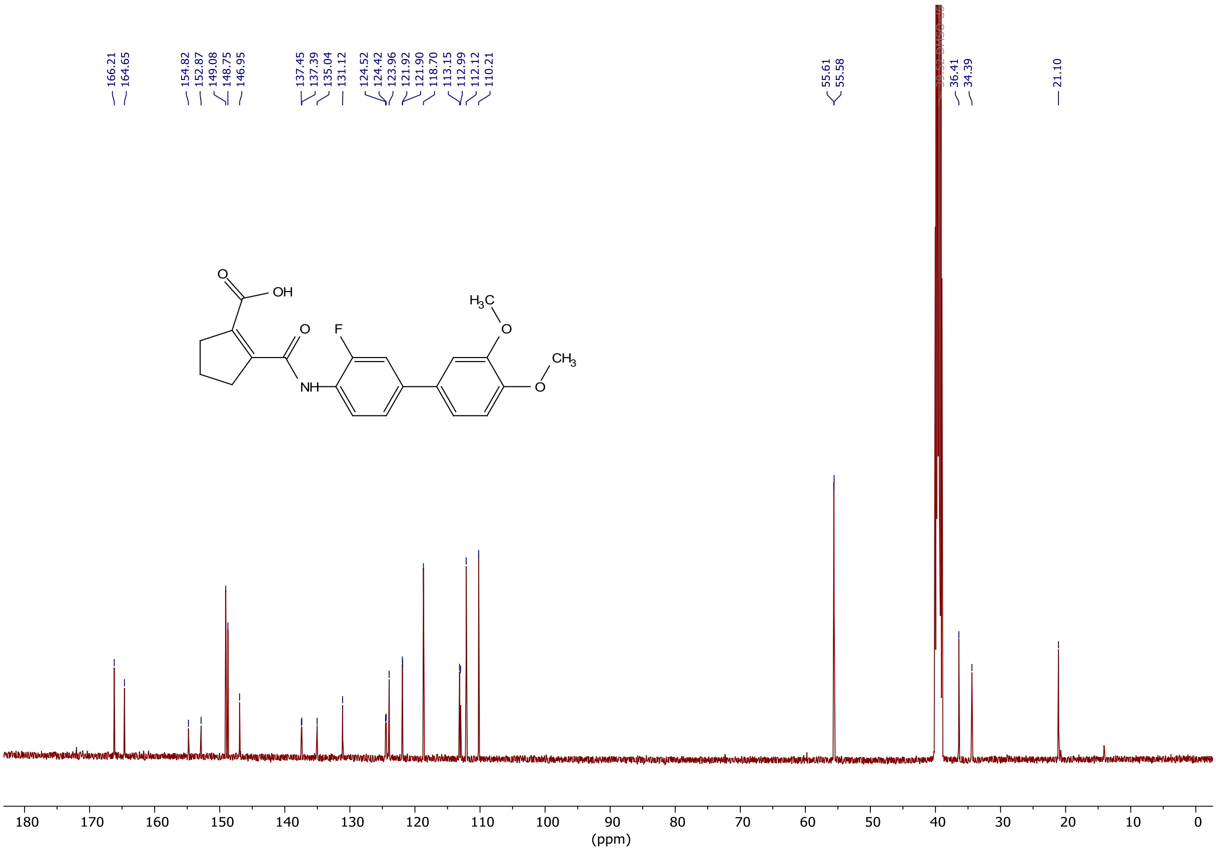

^{13}C NMR (126 MHz, DMSO-d_6) of **39**

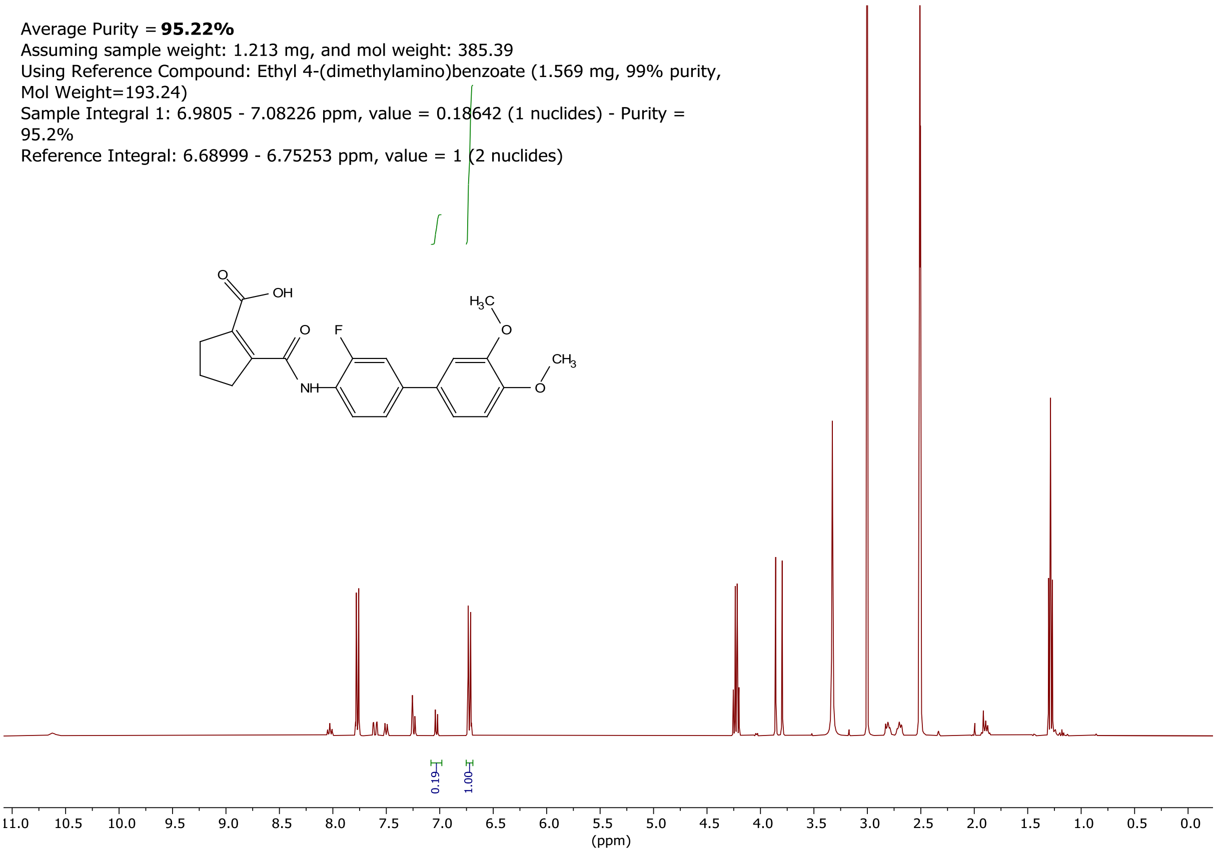

qH NMR (400 MHz, DMSO-d_6) of **39**

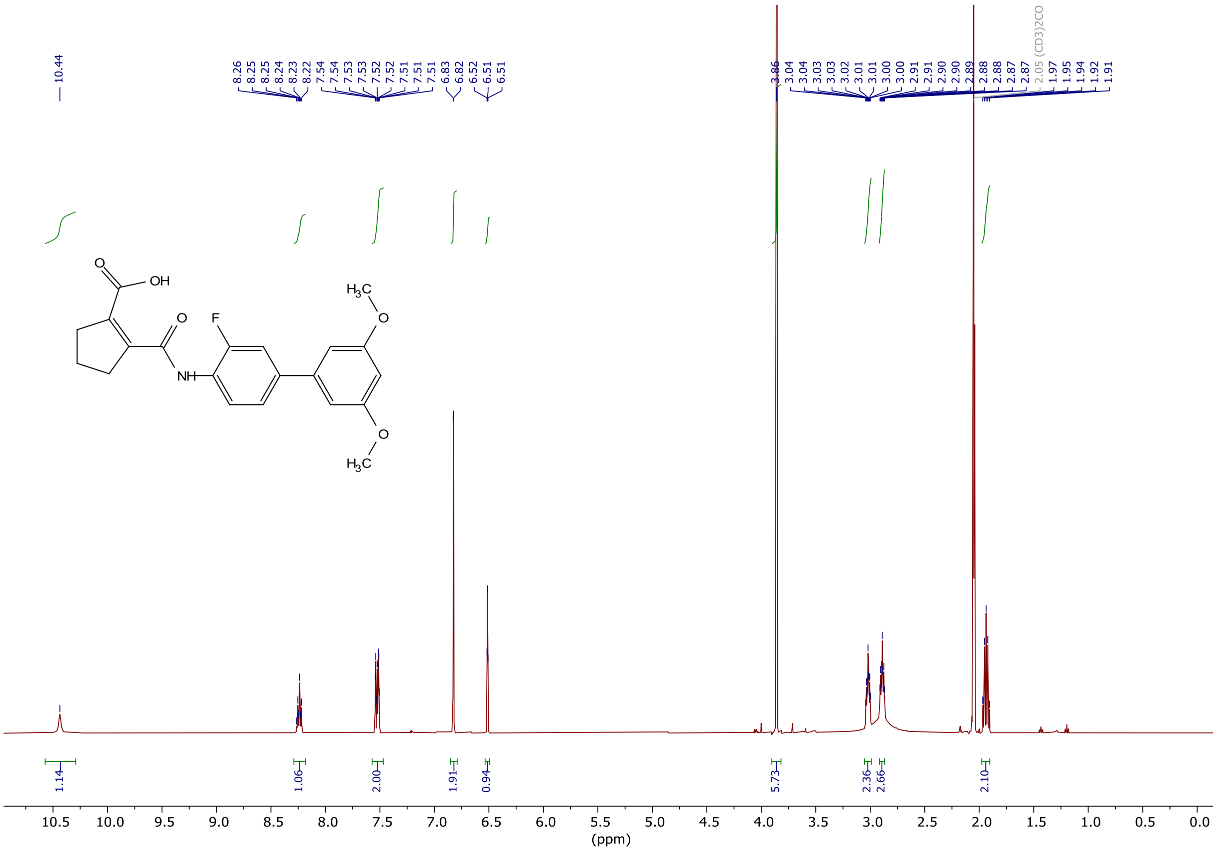

^{1}H NMR (500 MHz, acetone-d_6) of **40**

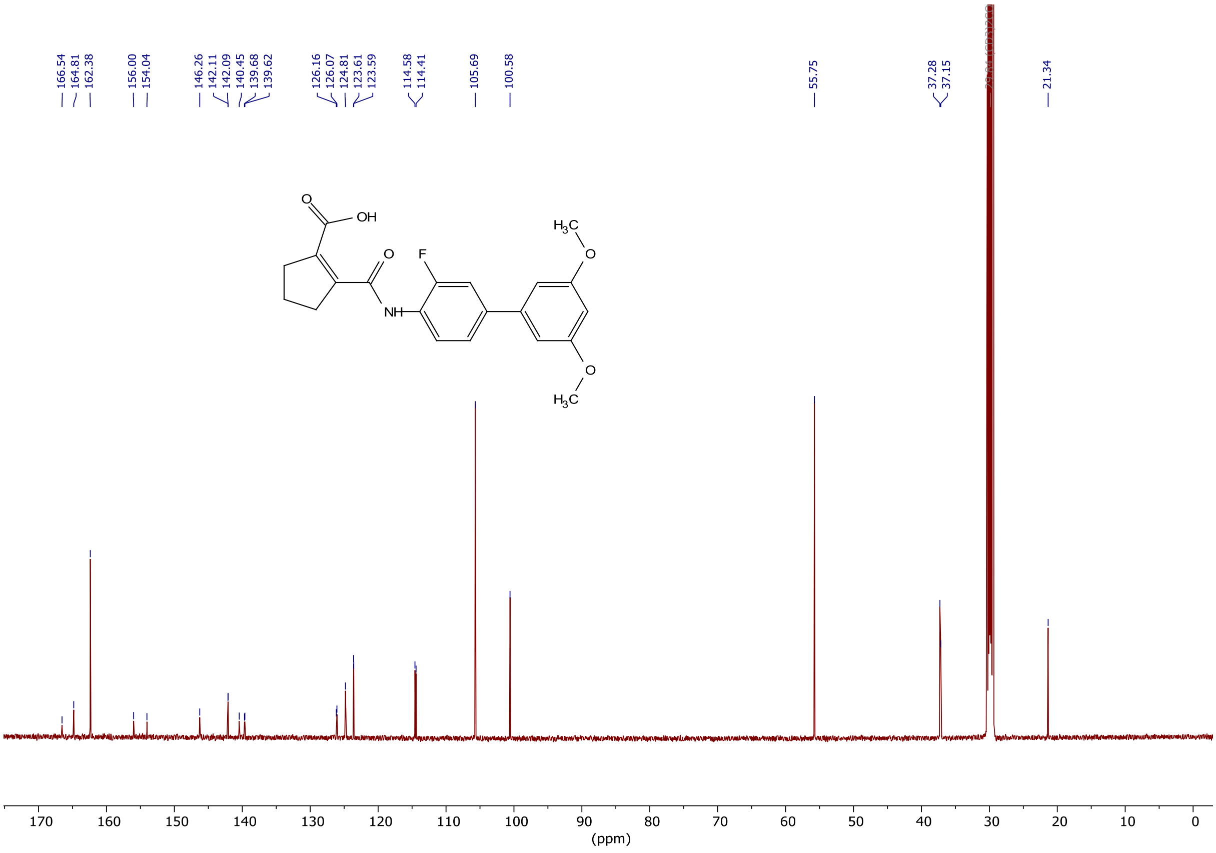

^{13}C NMR (126 MHz, acetone-d_6) of **40**

Average Purity = **96.65%**
Assuming sample weight: 1.213 mg, and mol weight: 385.39
Using Reference Compound: Ethyl 4-(dimethylamino)benzoate (2.334 mg, 99% purity, Mol Weight=193.24)
Sample Integral 1: 6.777 - 6.87928 ppm, value = 0.25441 (1 nuclides) - Purity = 96.7%
Reference Integral: 6.69707 - 6.74381 ppm, value = 1 (1 nuclides)

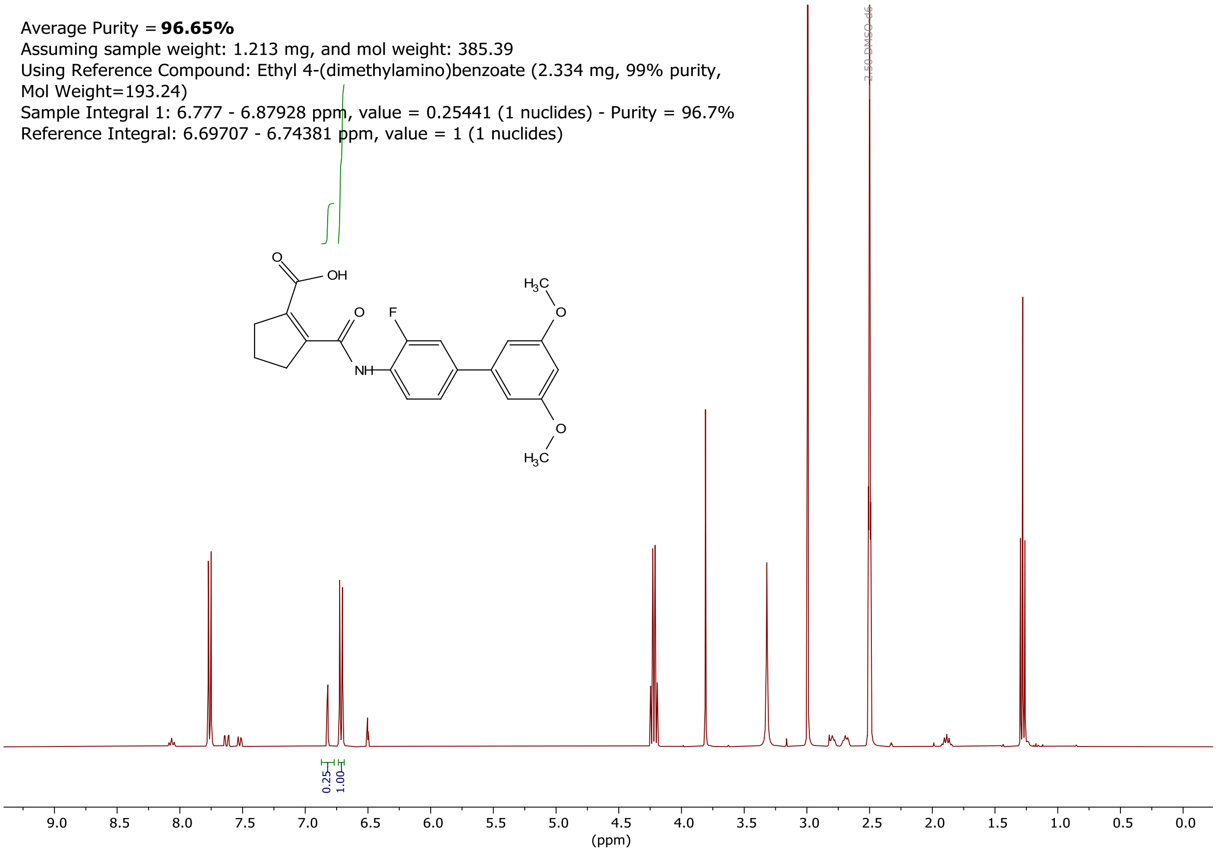

qH NMR (400 MHz, DMSO-d_6) of **40**

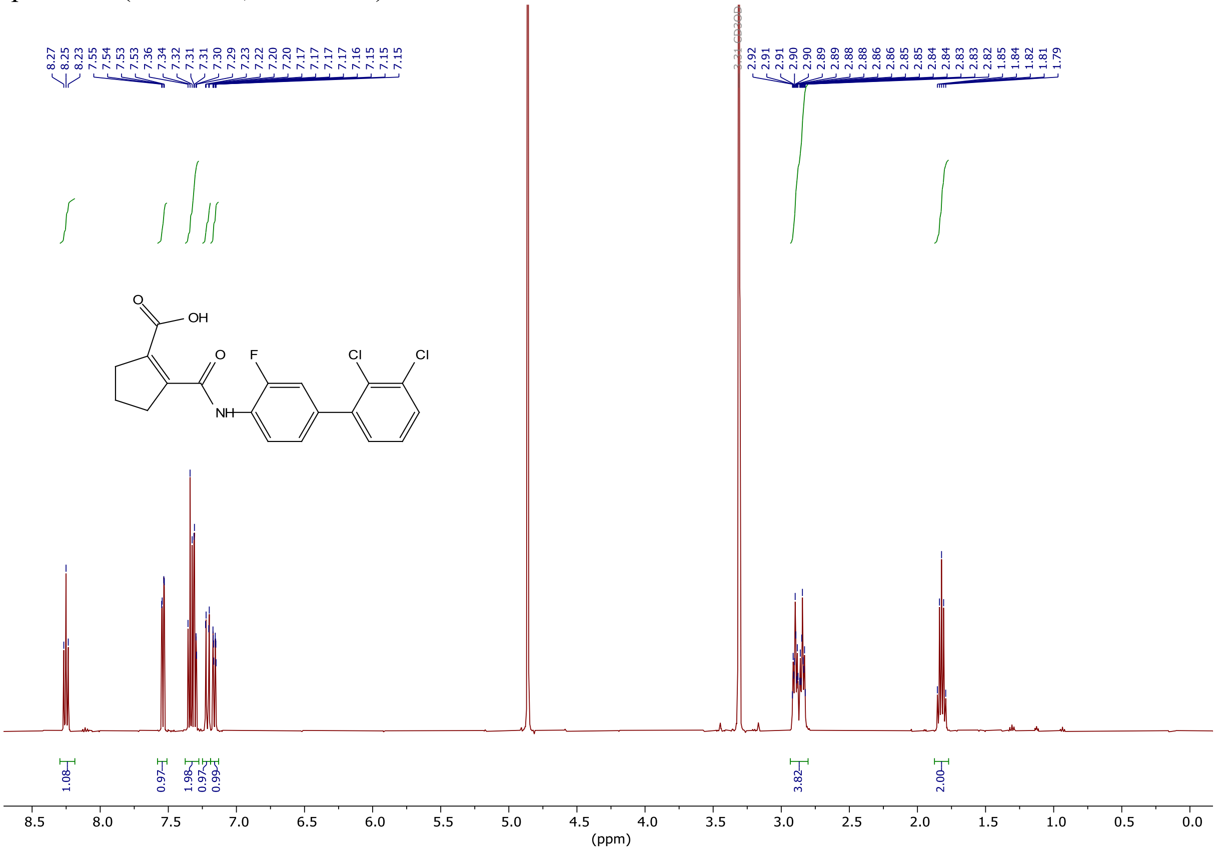

^{1}H NMR (500 MHz, MeOD-d_4) of **41**

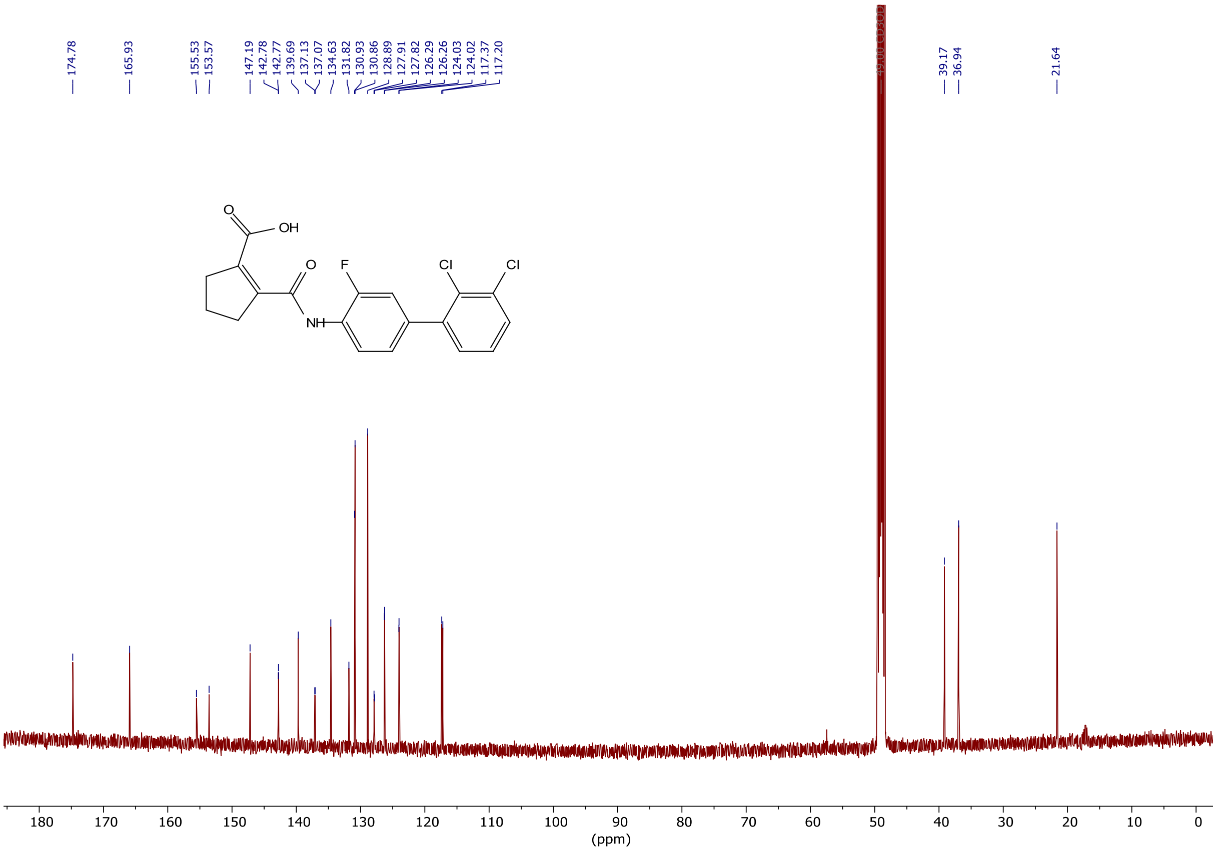

^{13}C NMR (126 MHz, MeOD-d_4) of **41**

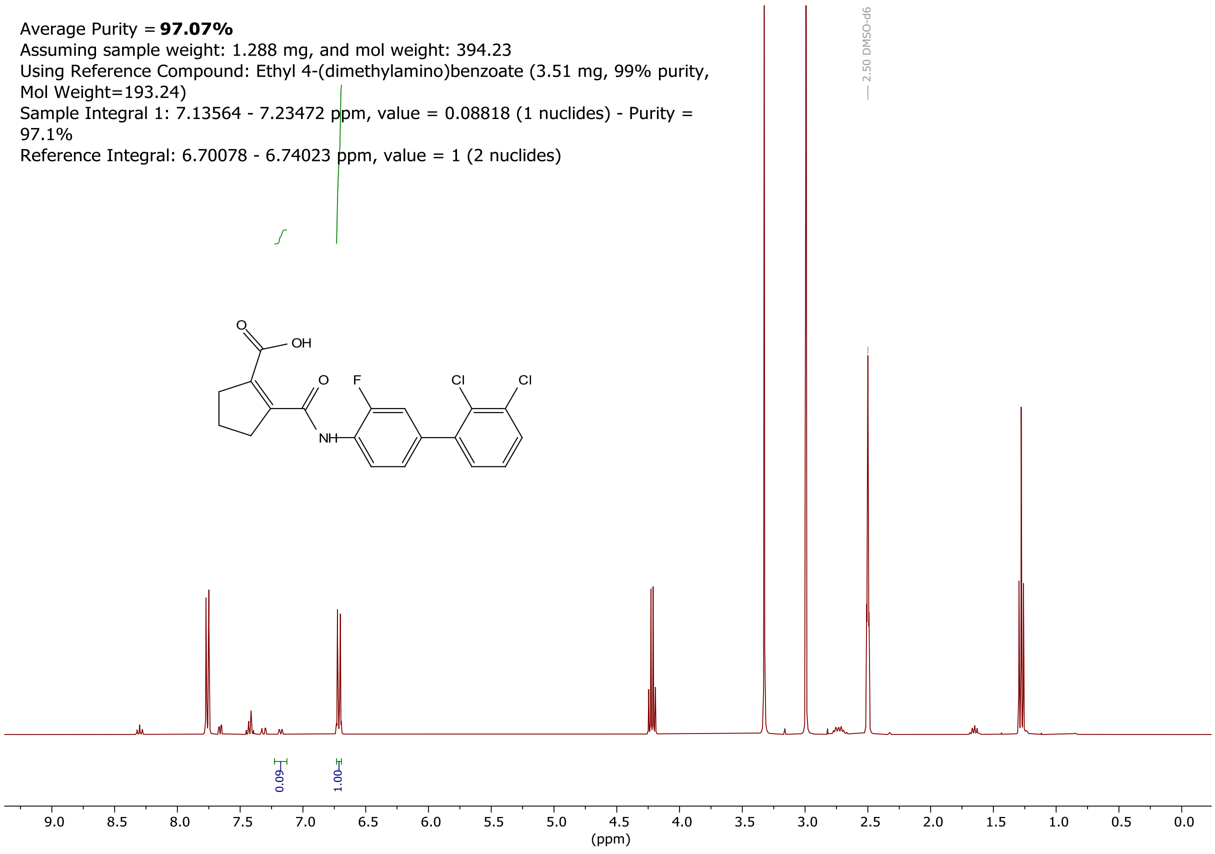

qH NMR (400 MHz, DMSO-d_6) of **41**

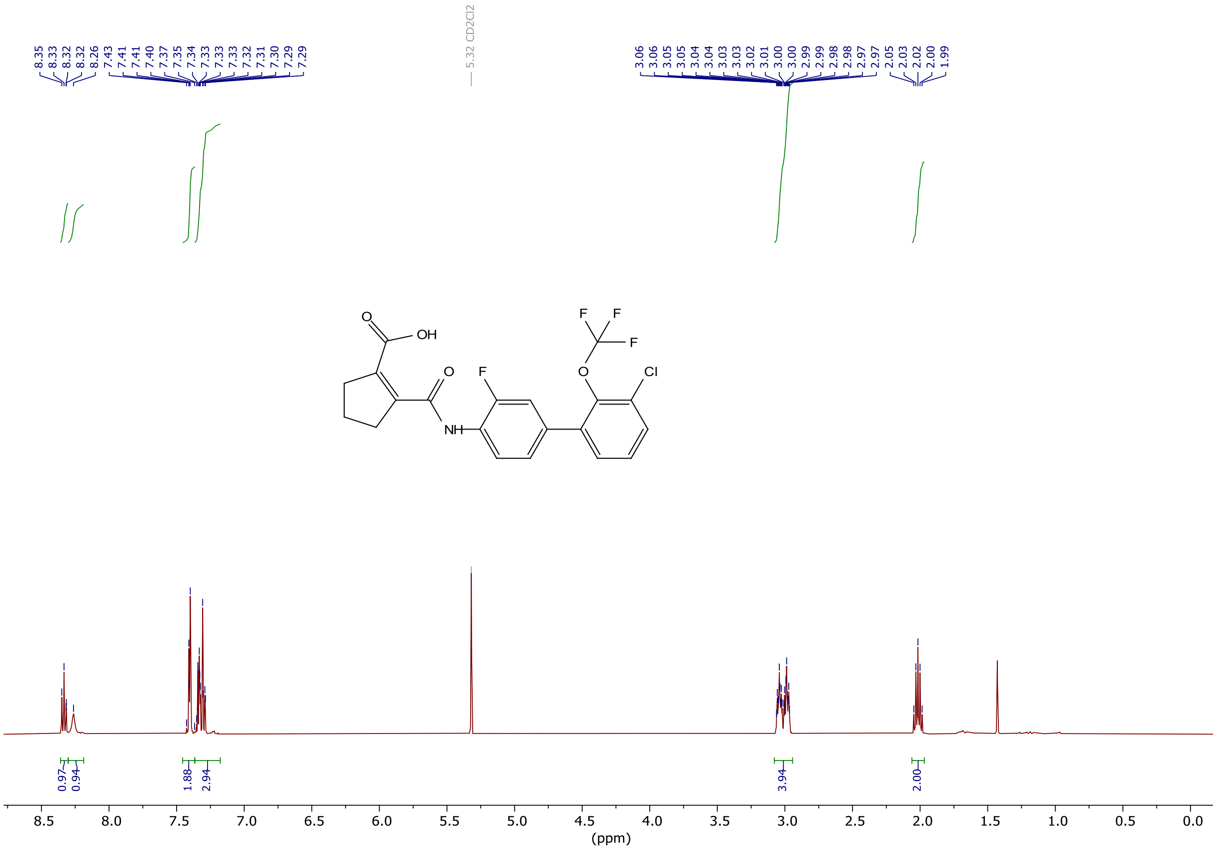
^{1}H NMR (500 MHz, CD_2Cl_2) of **42**

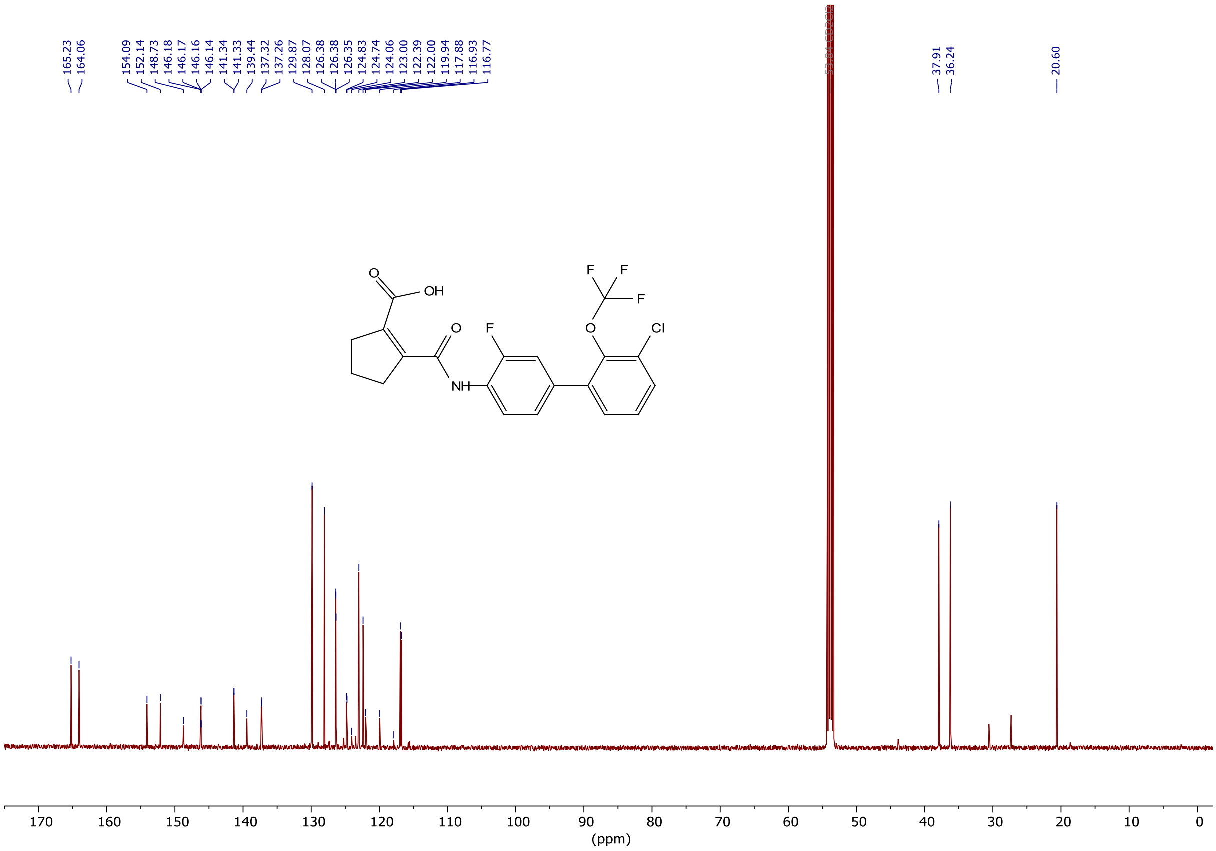
^{13}C NMR (126 MHz, CD_2Cl_2) of **42**

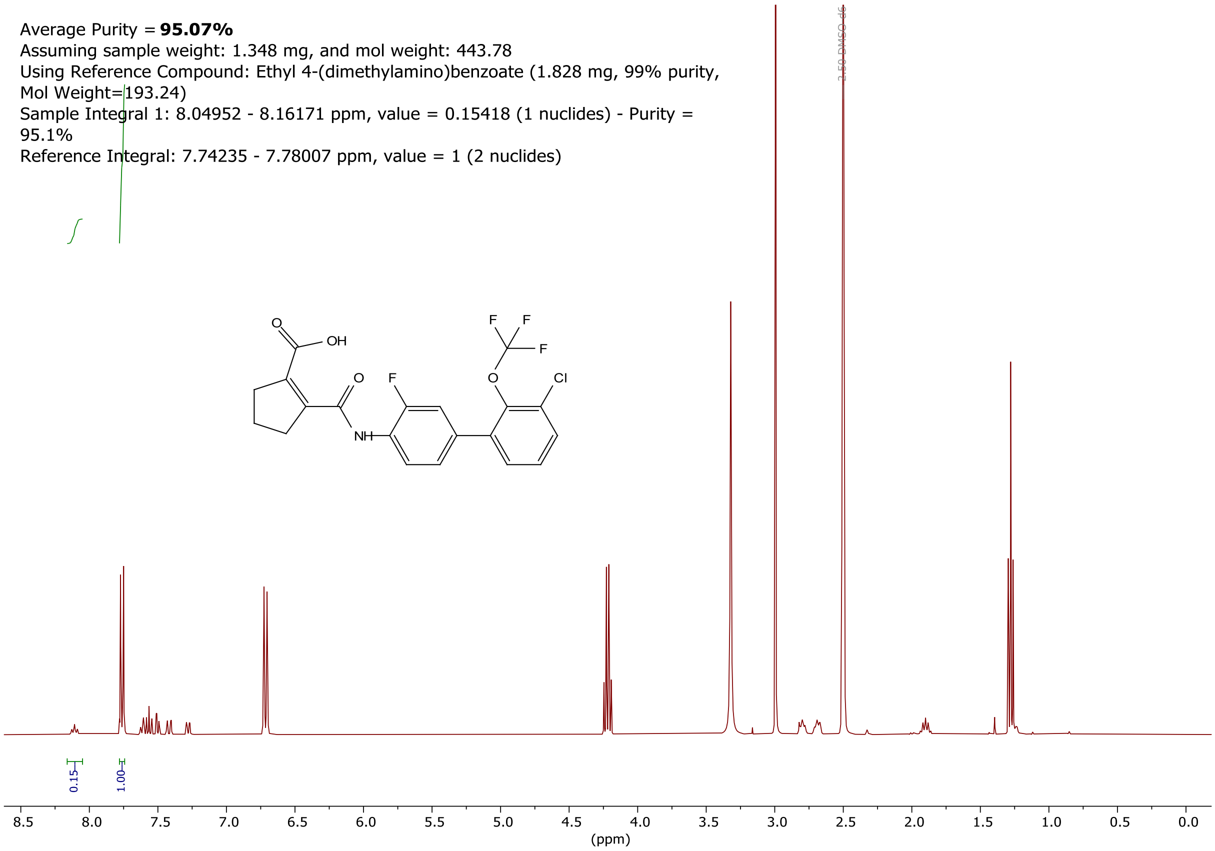

qH NMR (400 MHz, DMSO-d_6) of **42**

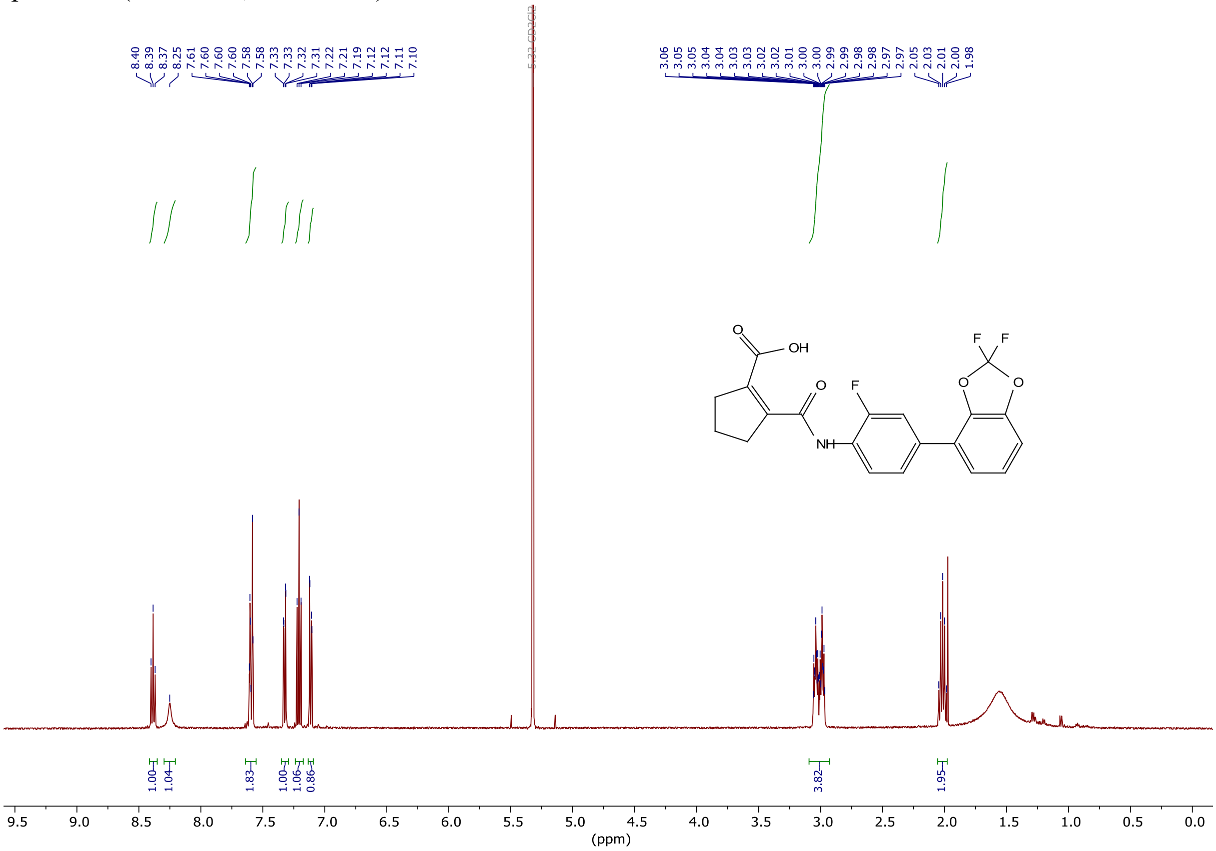

^{1}H NMR (500 MHz, CD_2Cl_2) of **43**

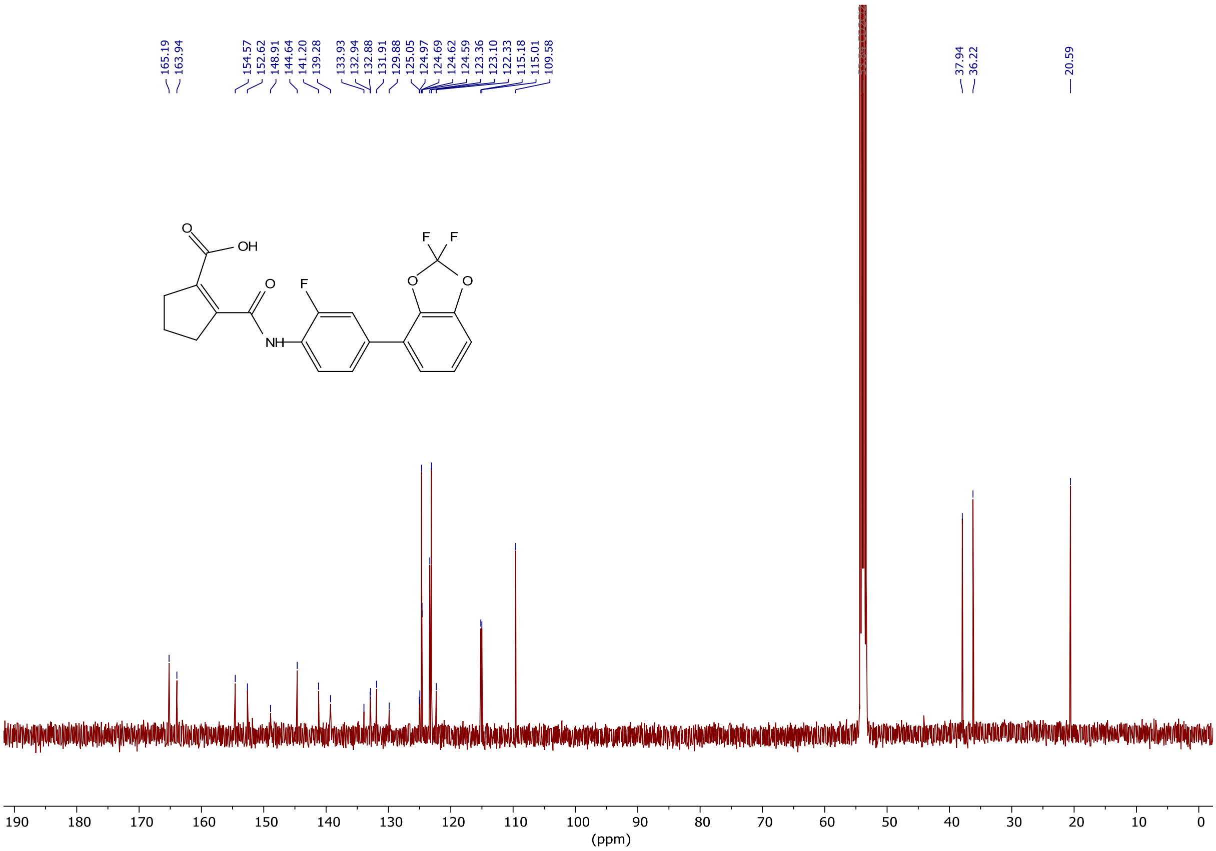

^{13}C NMR (126 MHz, CD_2Cl_2) of **43**

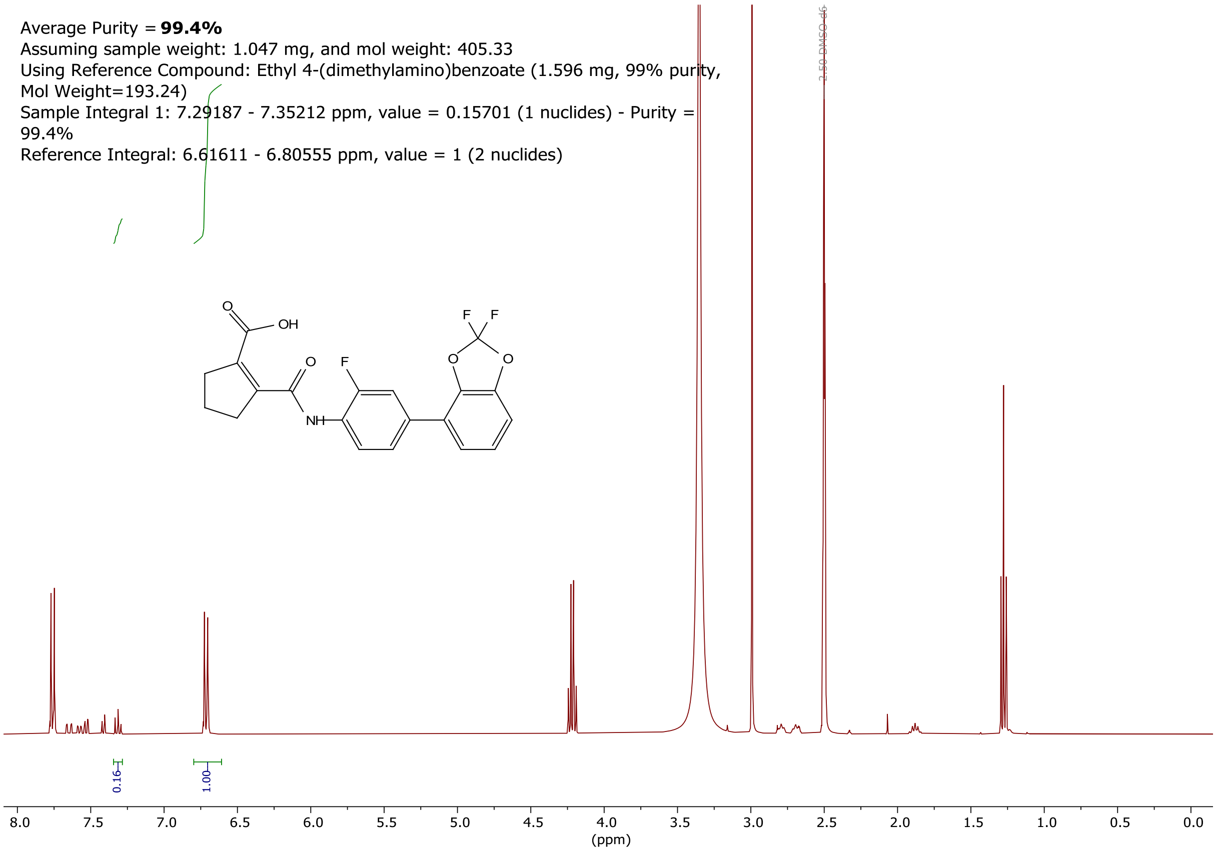

qH NMR (400 MHz, DMSO-d_6) of **43**

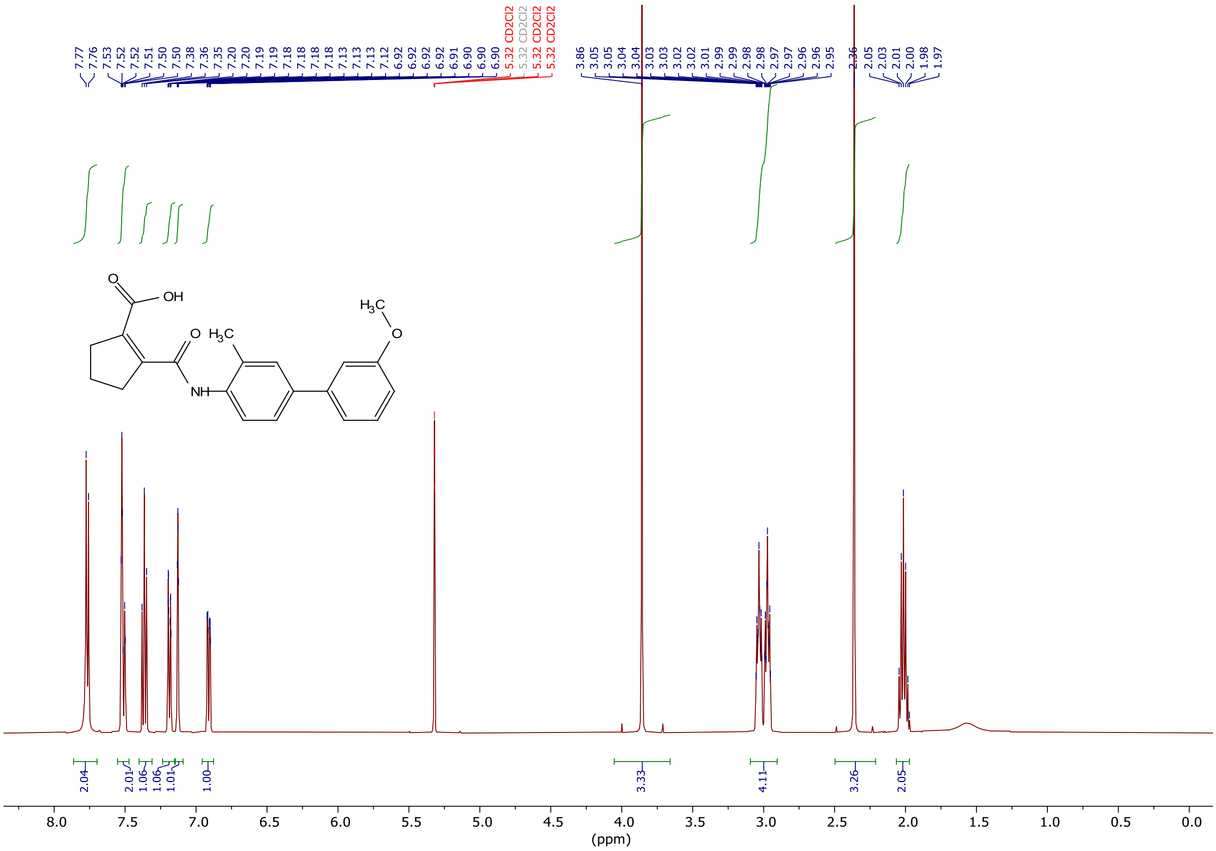

^{1}H NMR (500 MHz, CD_2Cl_2) of **44**

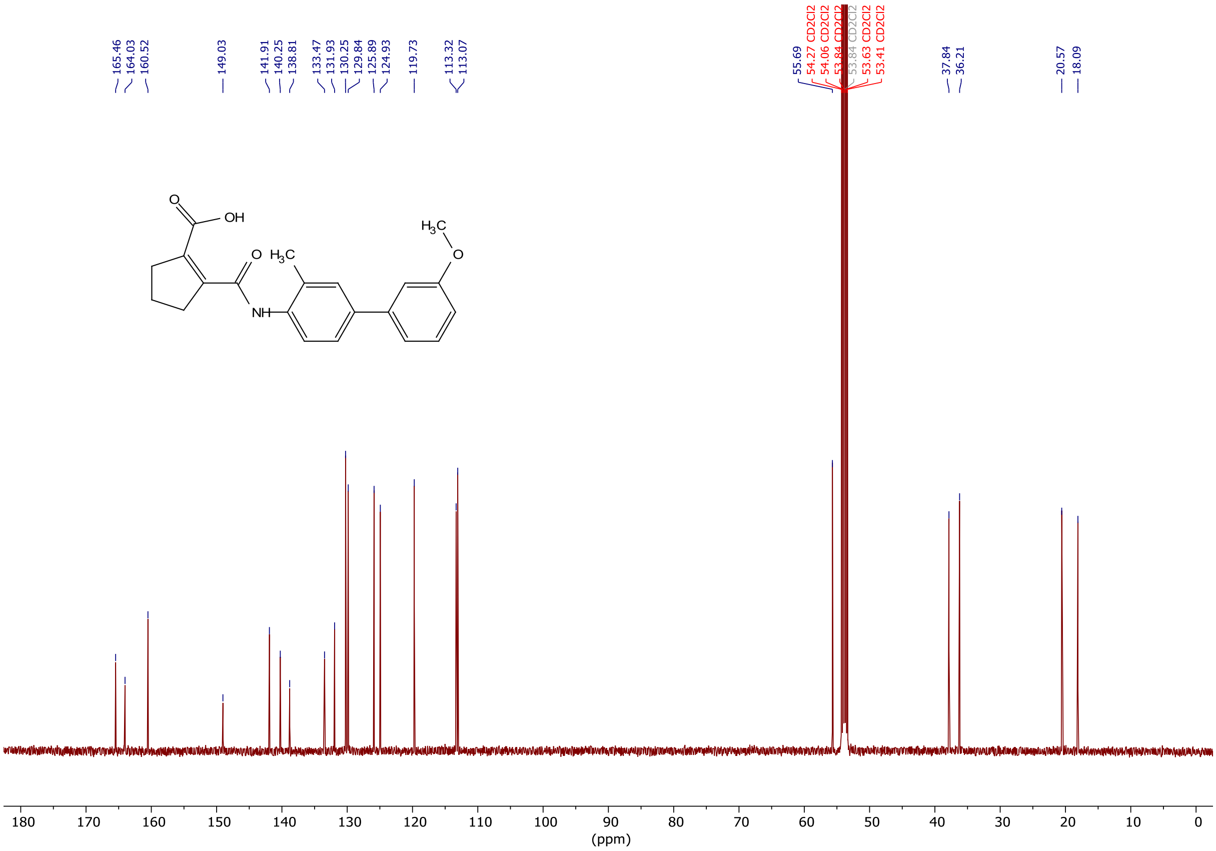

^{13}C NMR (126 MHz, CD_2Cl_2) of **44**

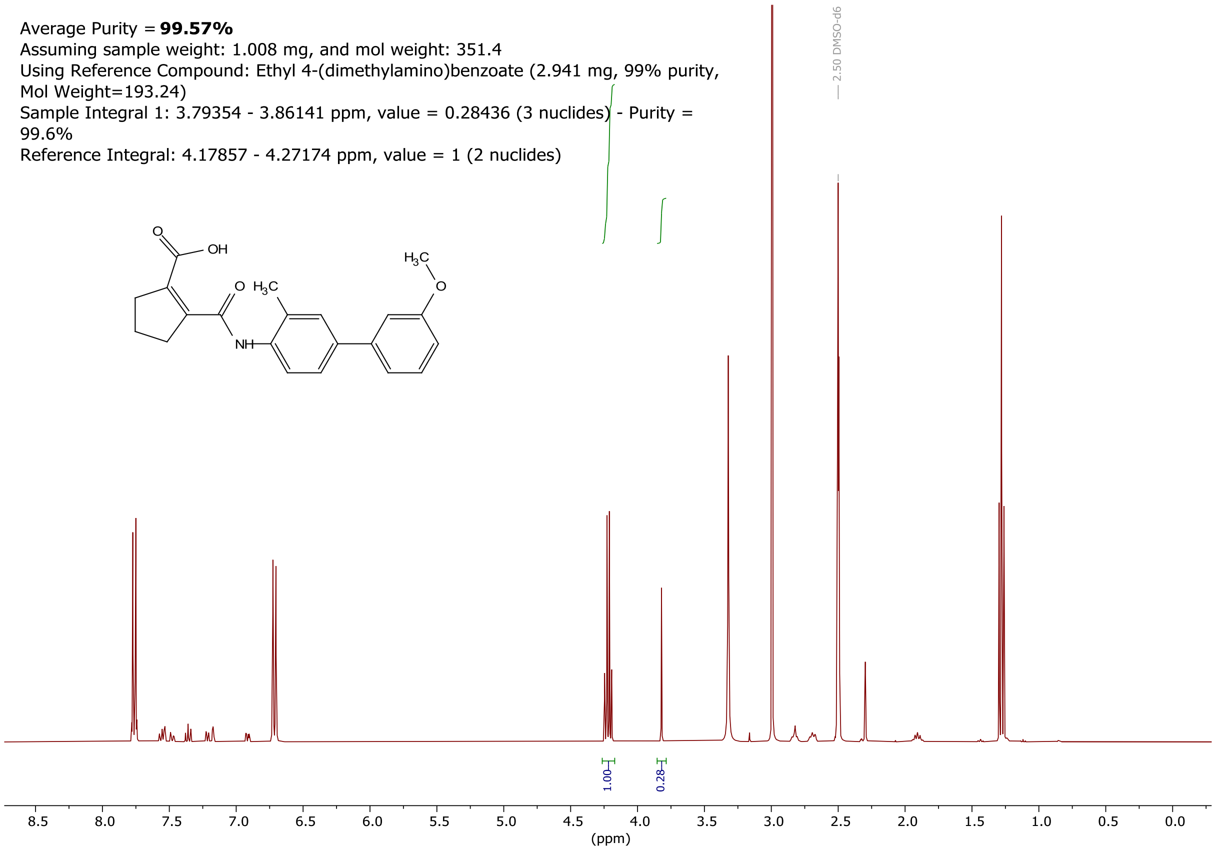

qH NMR (400 MHz, DMSO-d_6) of **44**

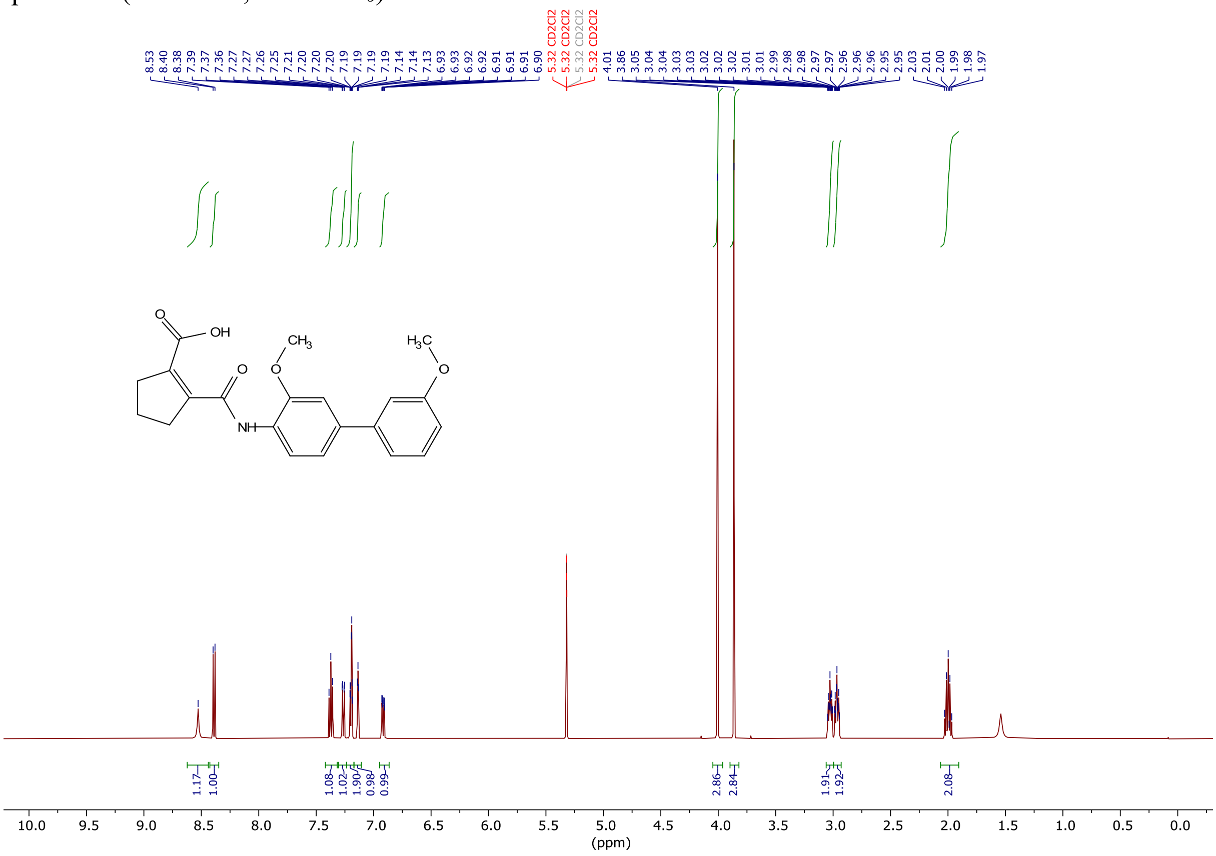

^{1}H NMR (500 MHz, CD_2Cl_2) of **45**

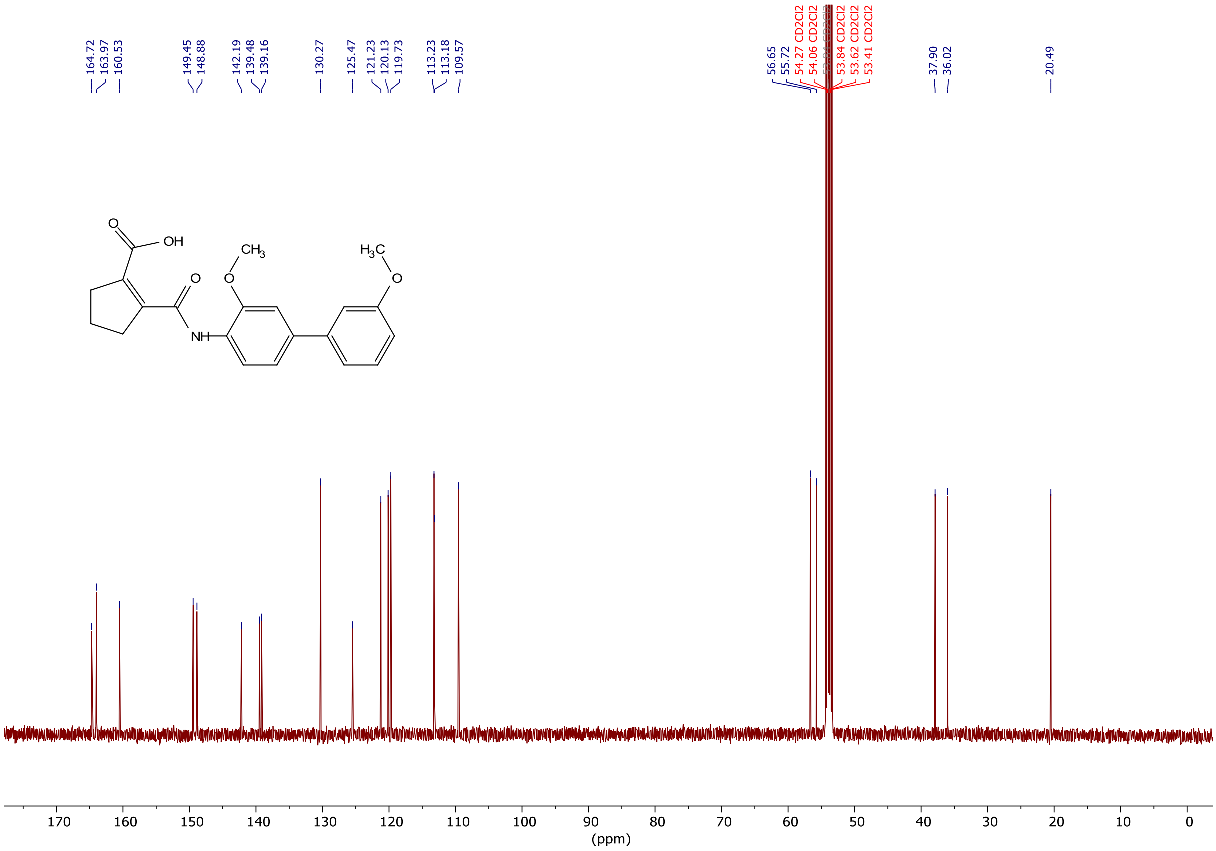

^{13}C NMR (126 MHz, CD_2Cl_2) of **45**

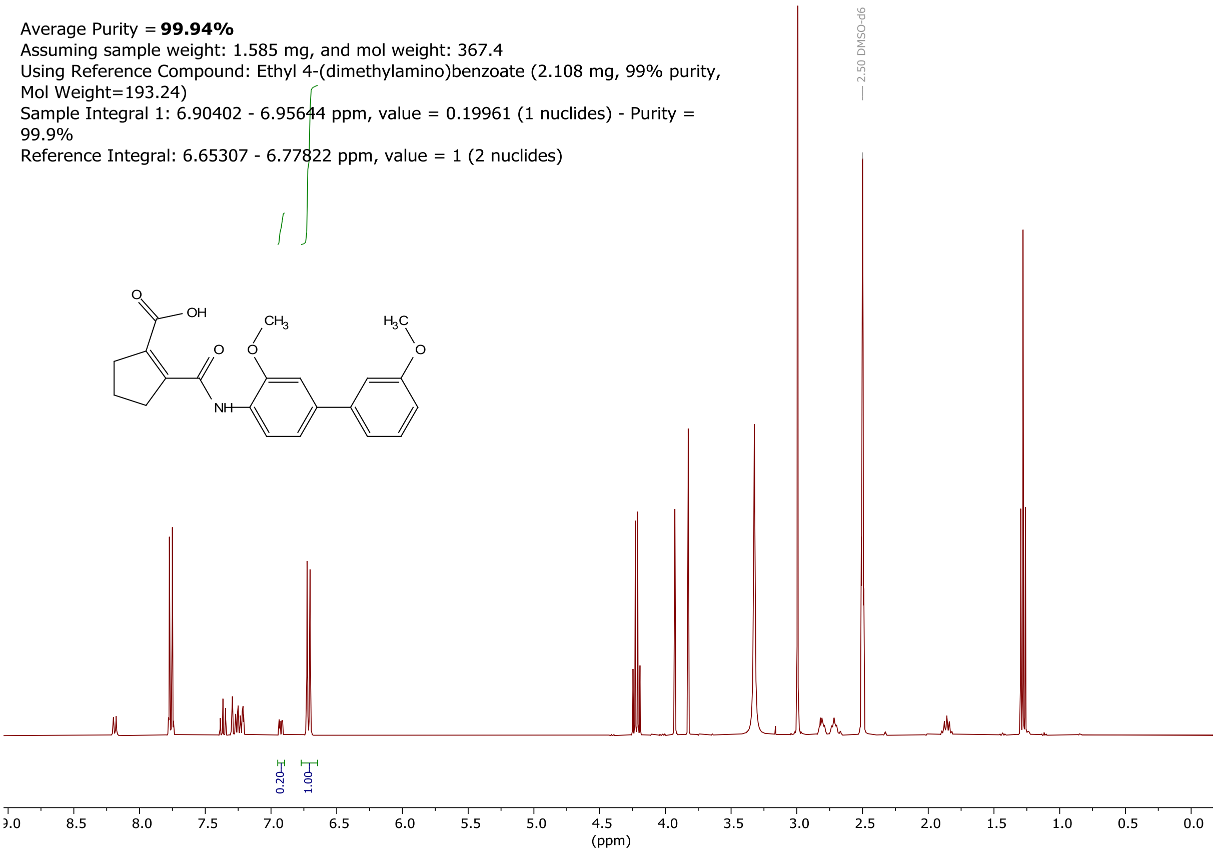

qH NMR (400 MHz, DMSO-d_6) of **45**

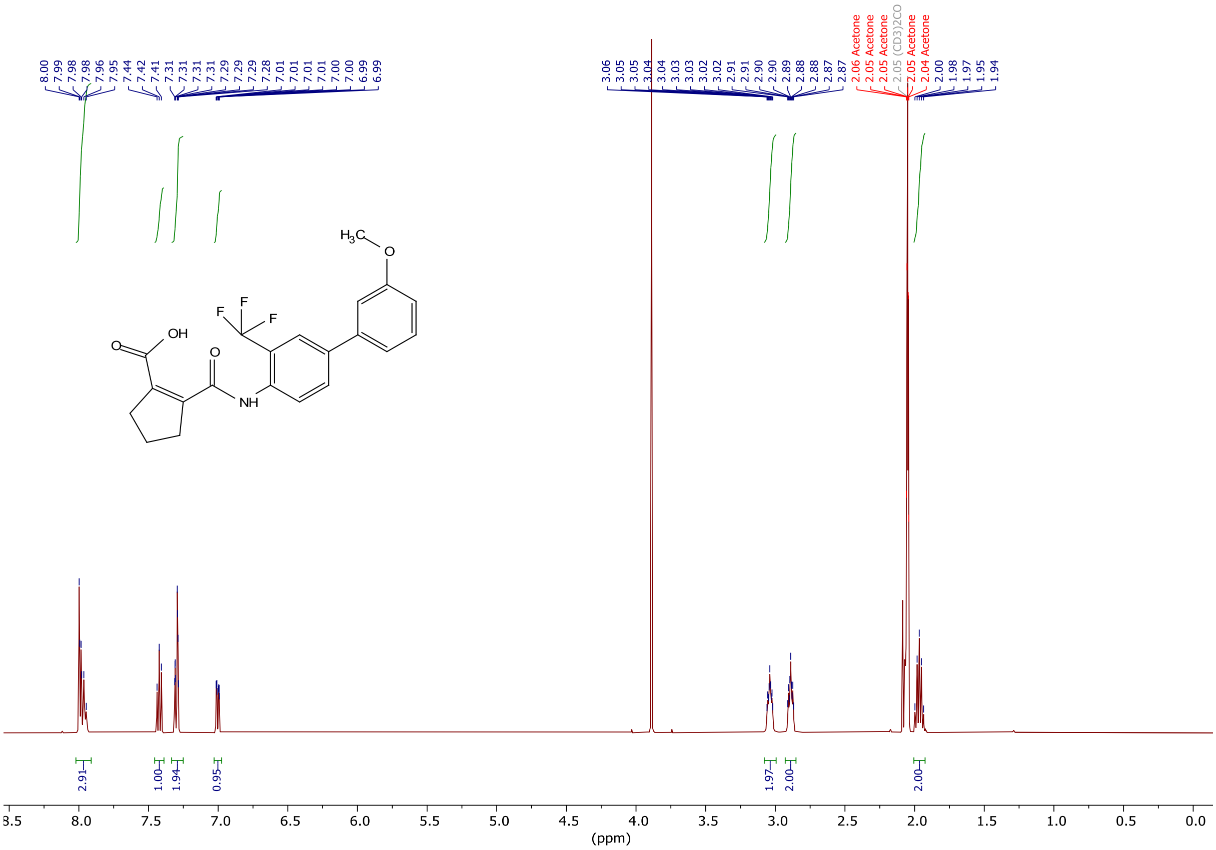

^{1}H NMR (500 MHz, acetone-d_6) of **46**

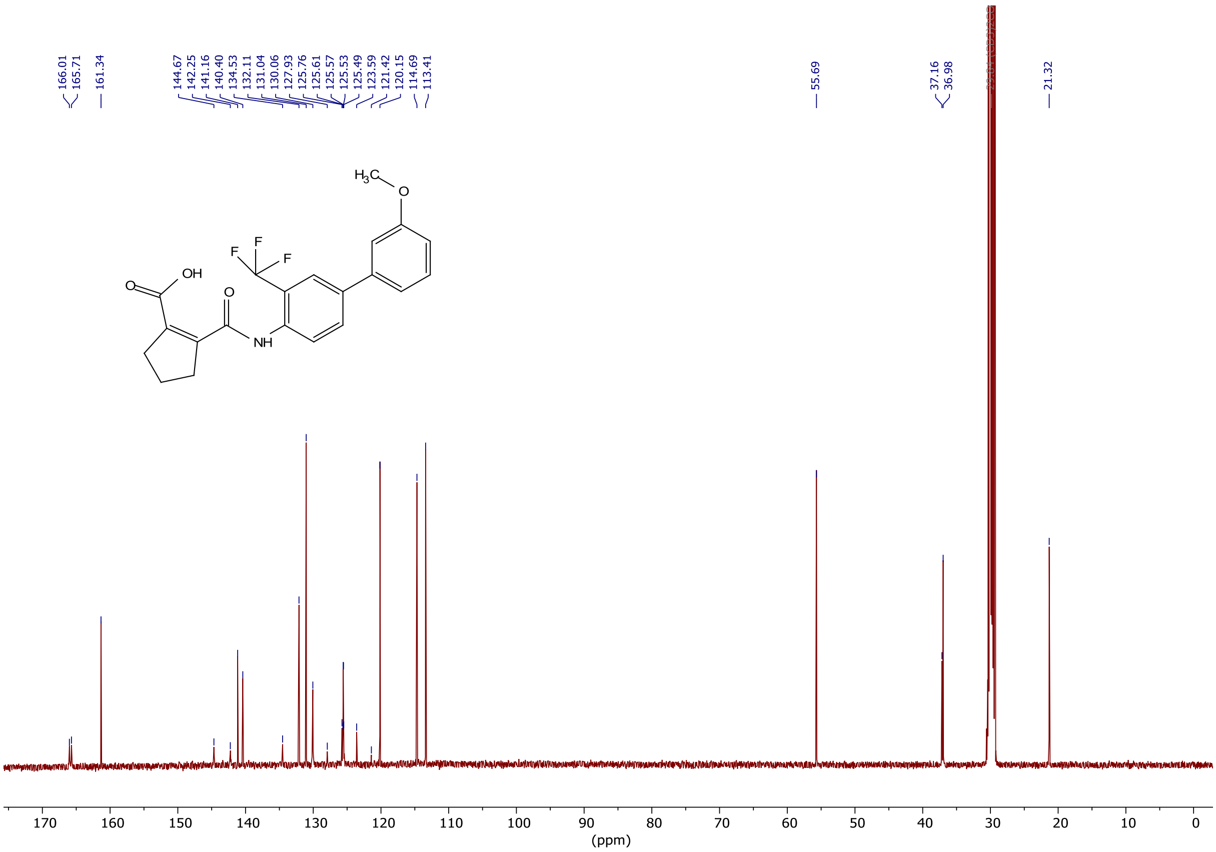

^{13}C NMR (126 MHz, acetone-d_6) of **46**

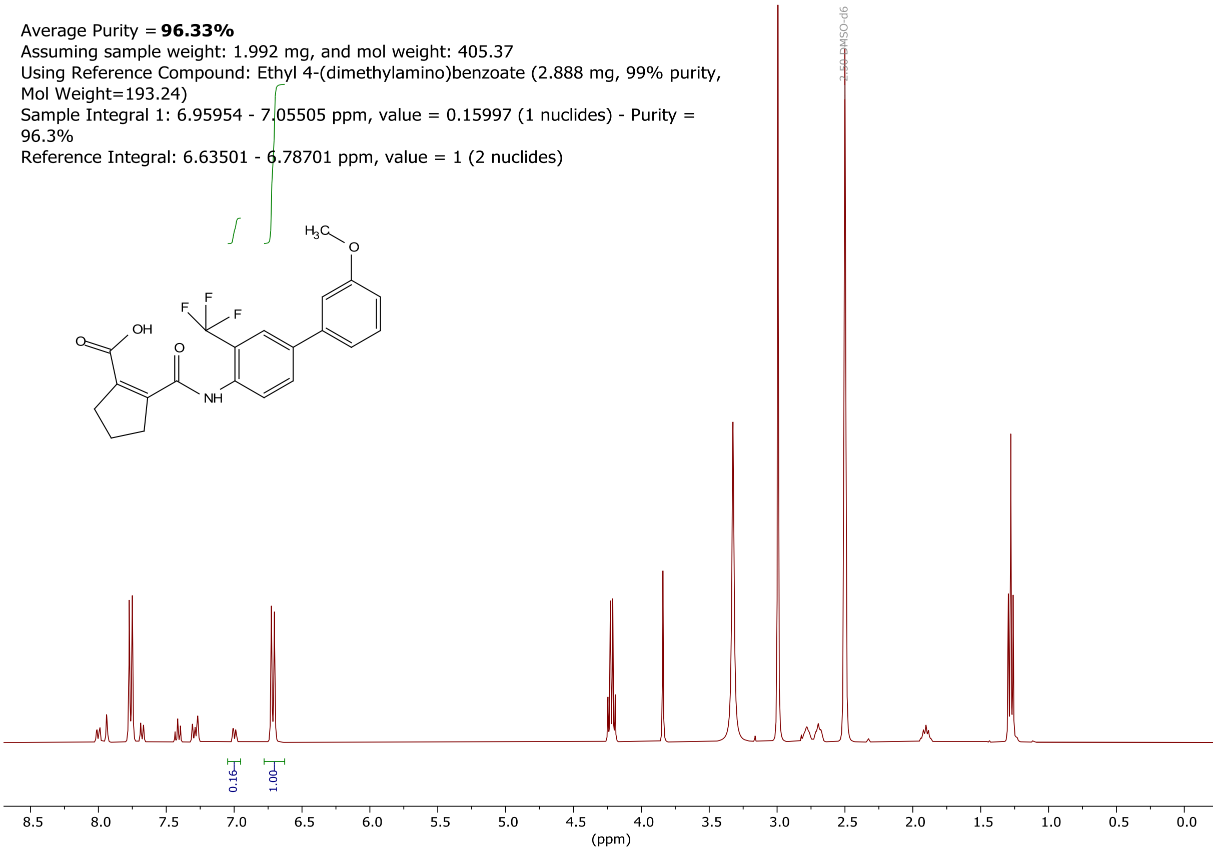

qH NMR (400 MHz, DMSO-d_6) of **46**

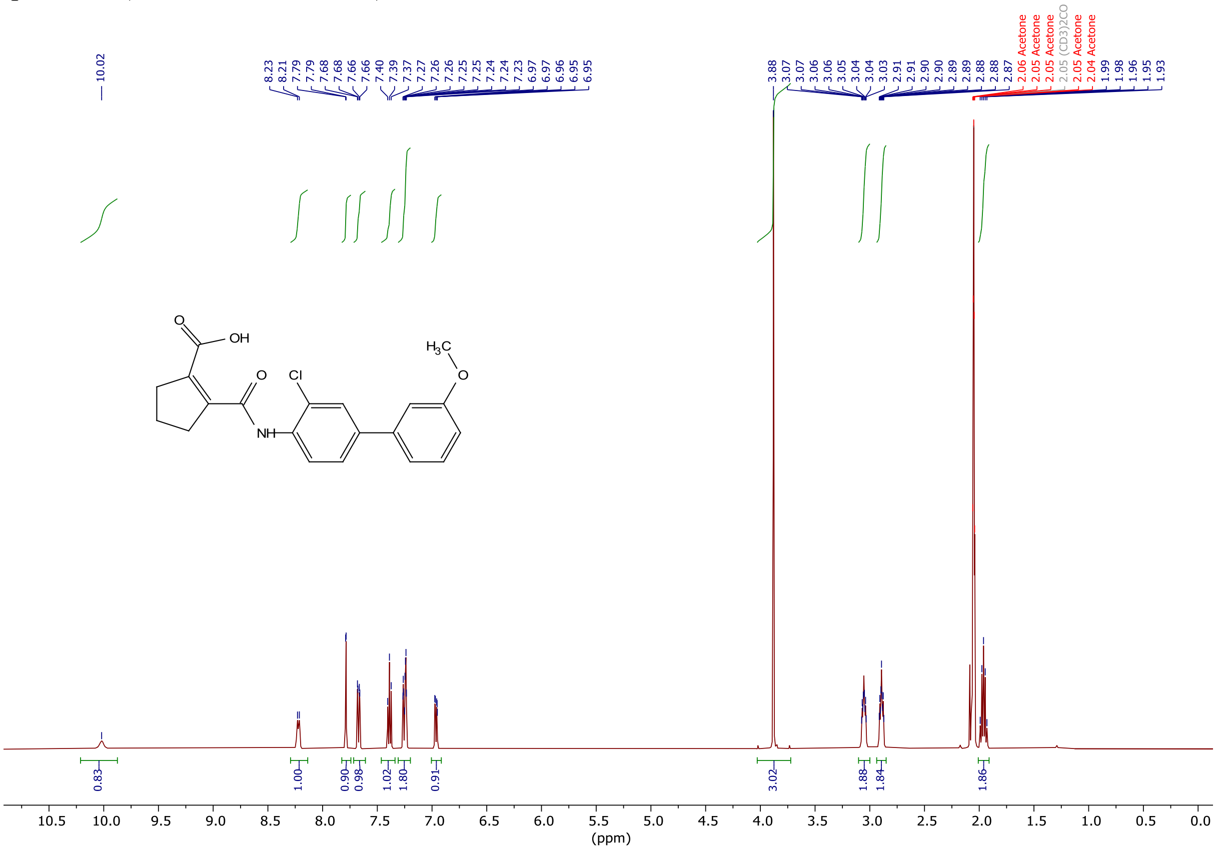

^{1}H NMR (500 MHz, acetone-d_6) of **47**

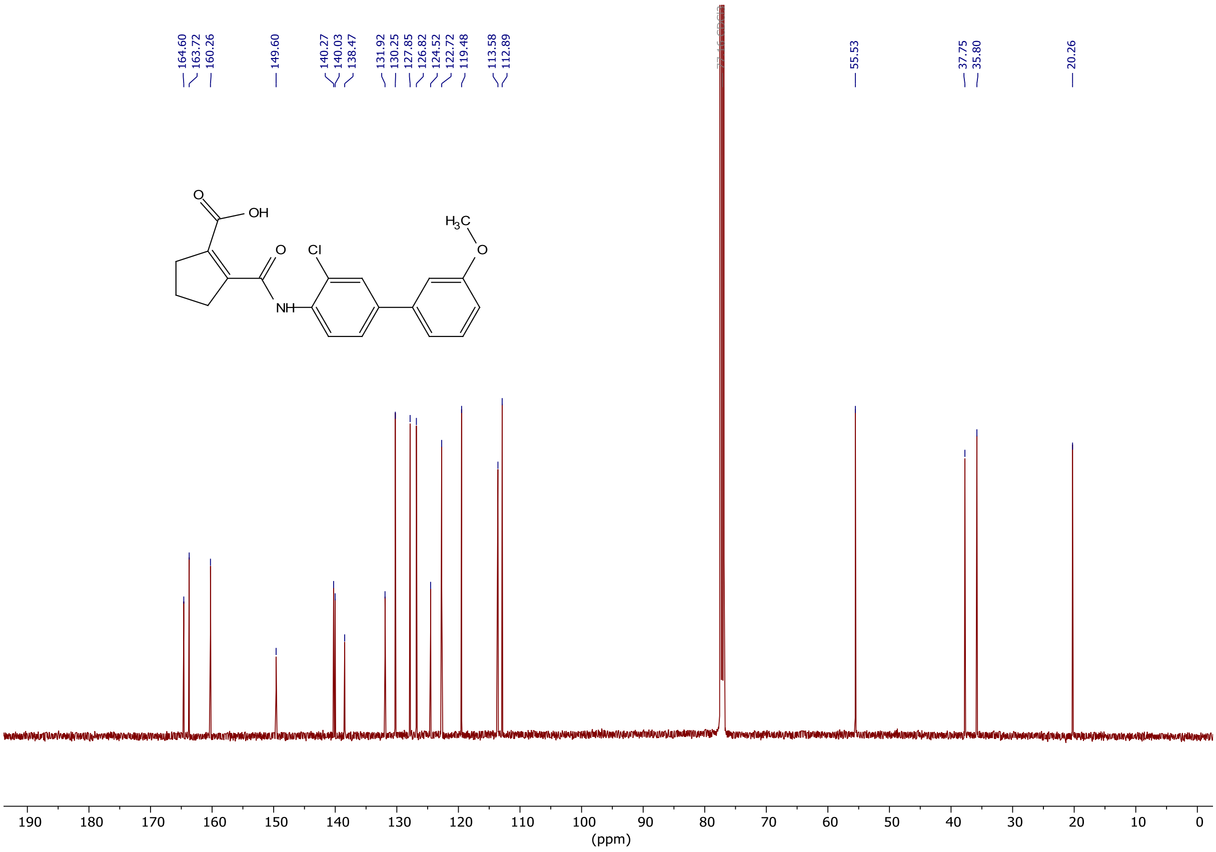

^{13}C NMR (126 MHz, $CDCl_3$) of **47**

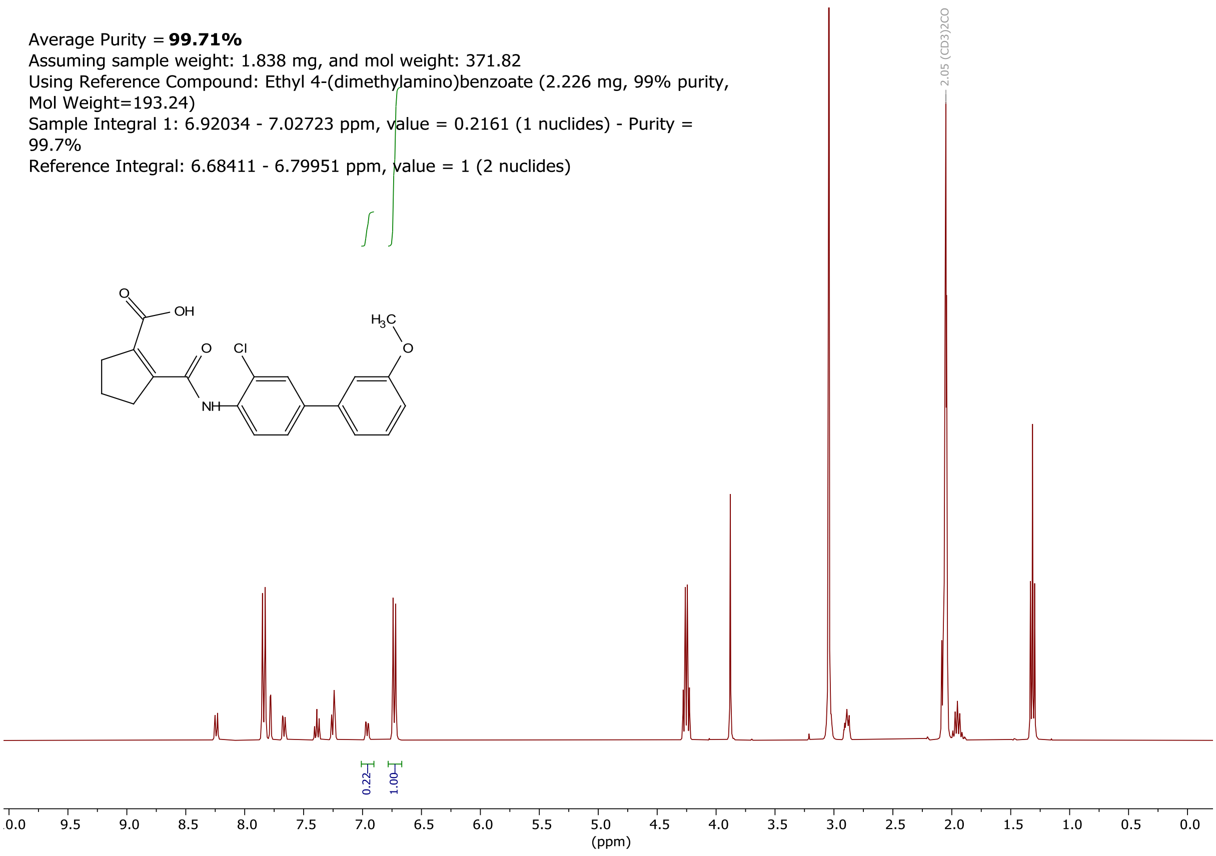

qH NMR (400 MHz, DMSO-d_6) of **47**

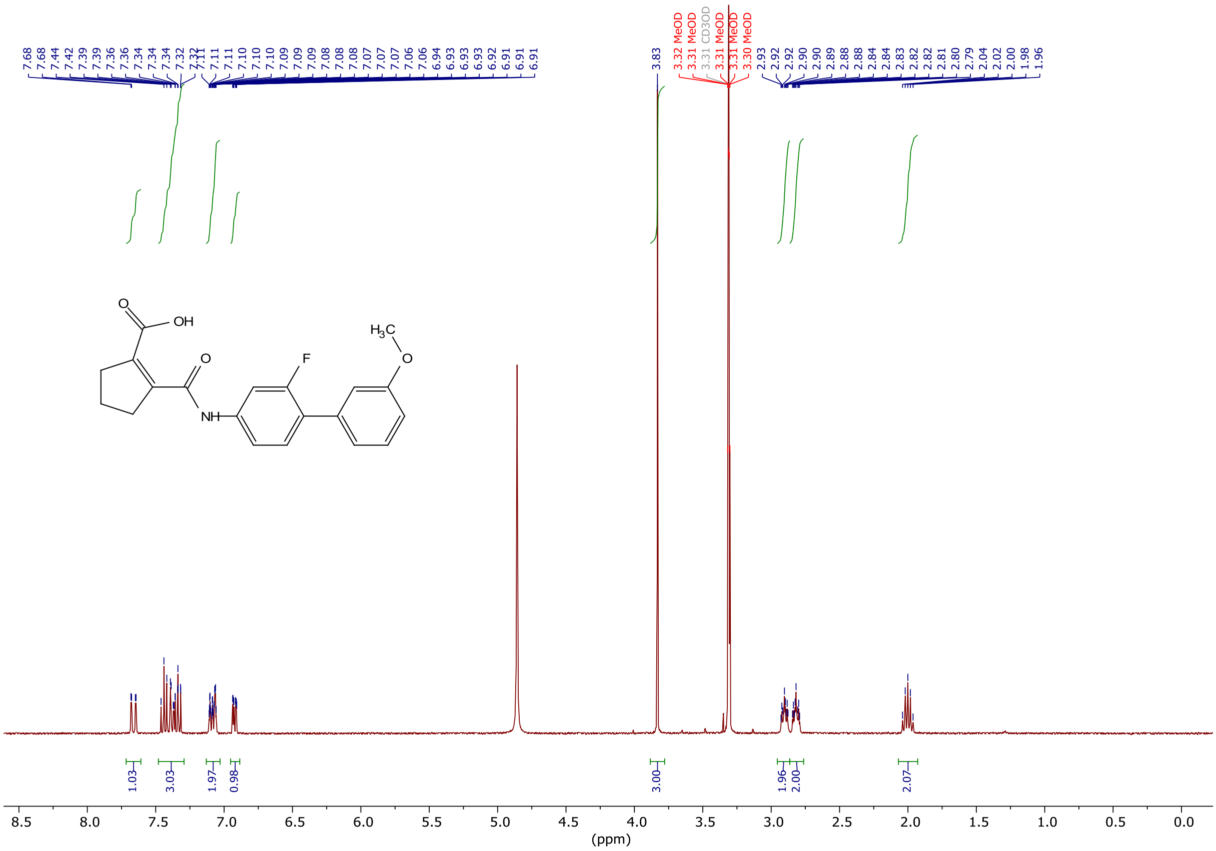

^{1}H NMR (500 MHz, MeOD-d_4) of **48**

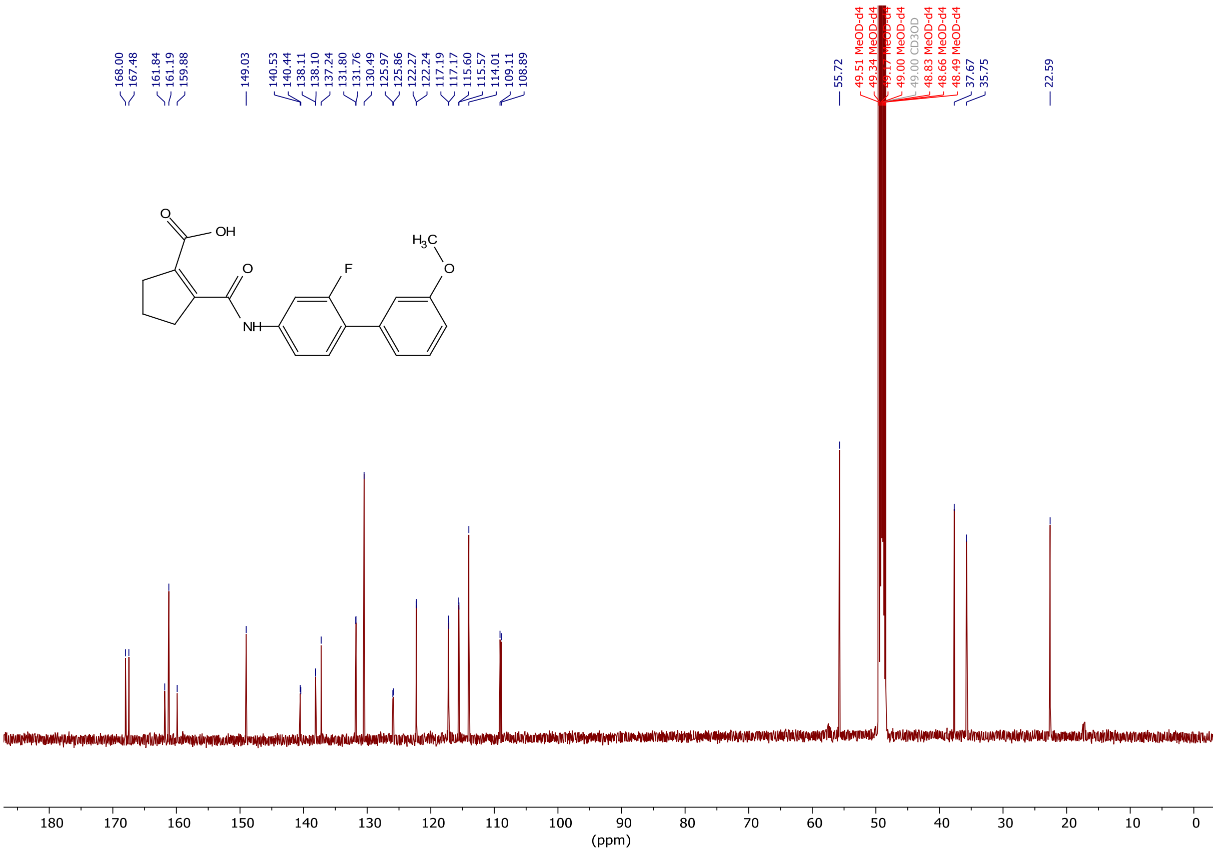

^{13}C NMR (126 MHz, MeOD-d_4) of **48**

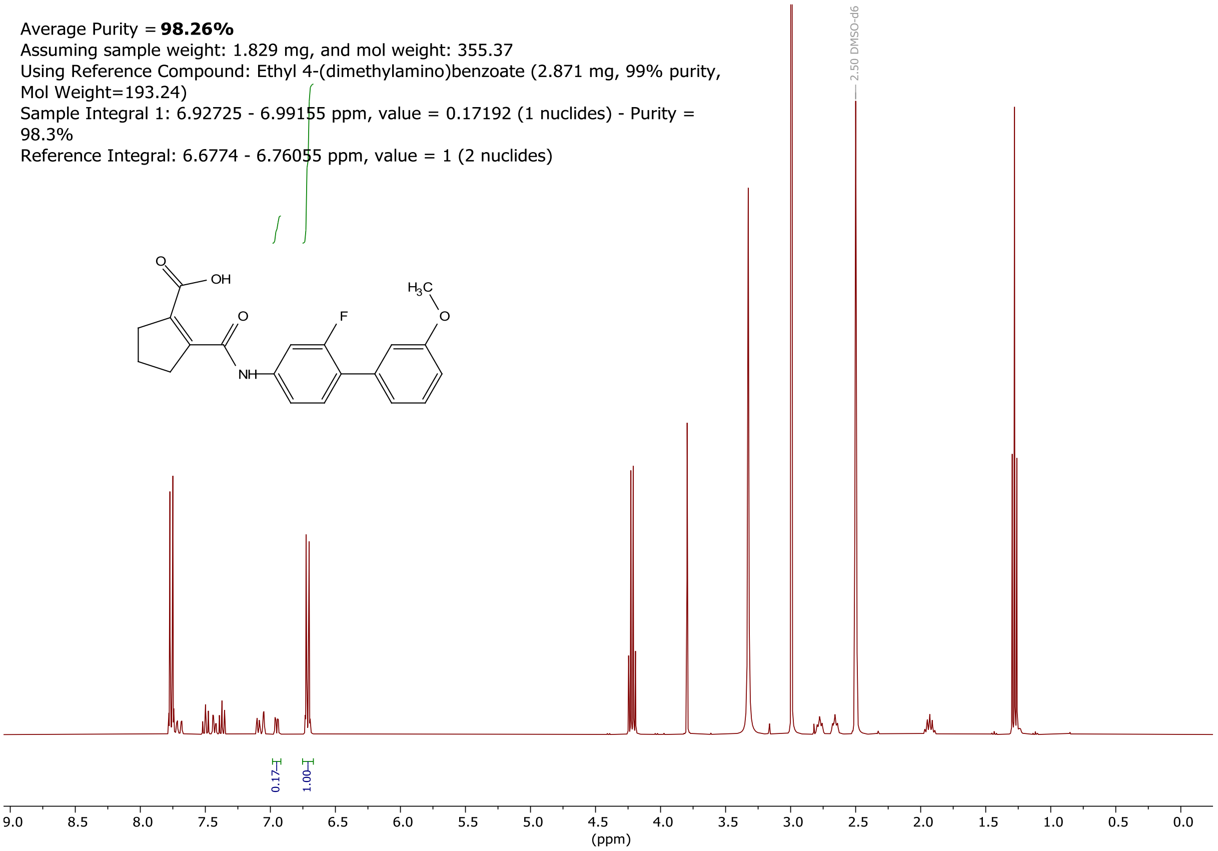

qH NMR (400 MHz, DMSO-d_6) of **48**

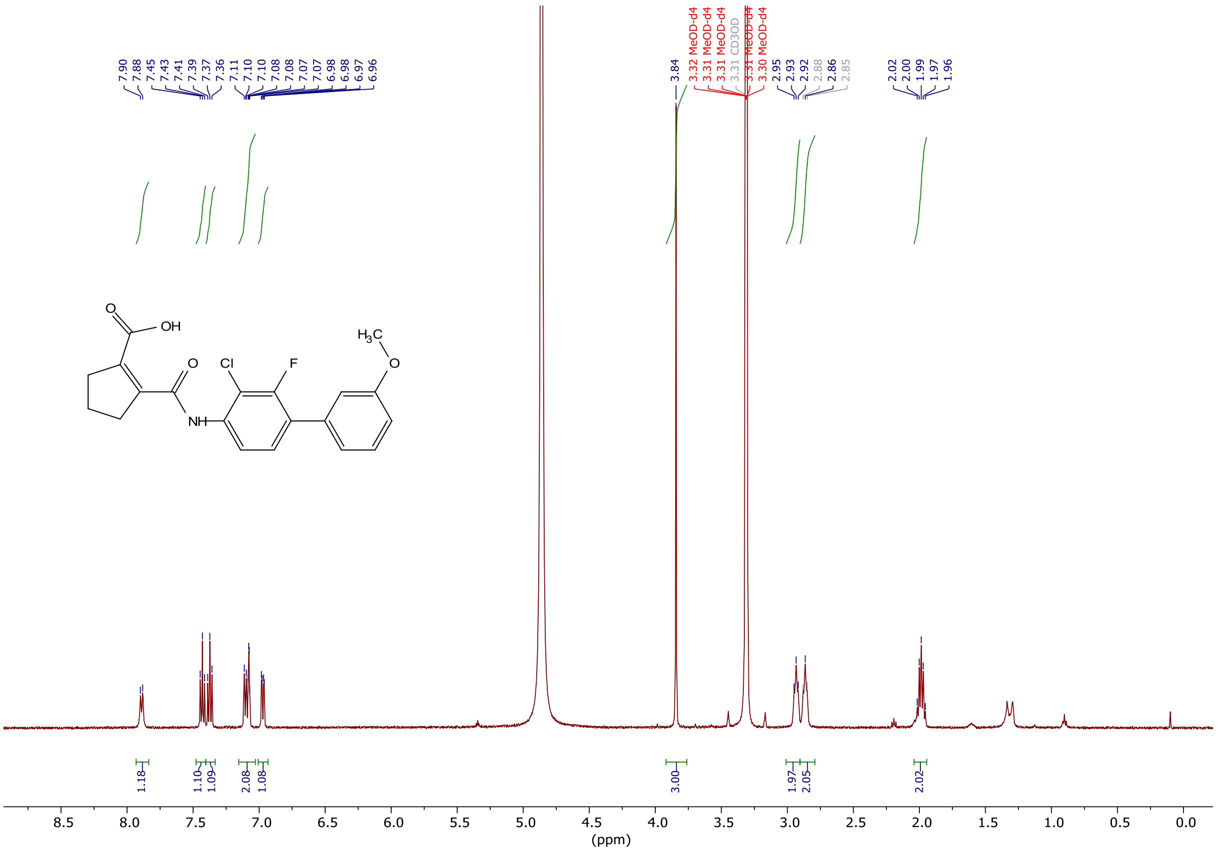

^{1}H NMR (500 MHz, MeOD-d_4) of **49**

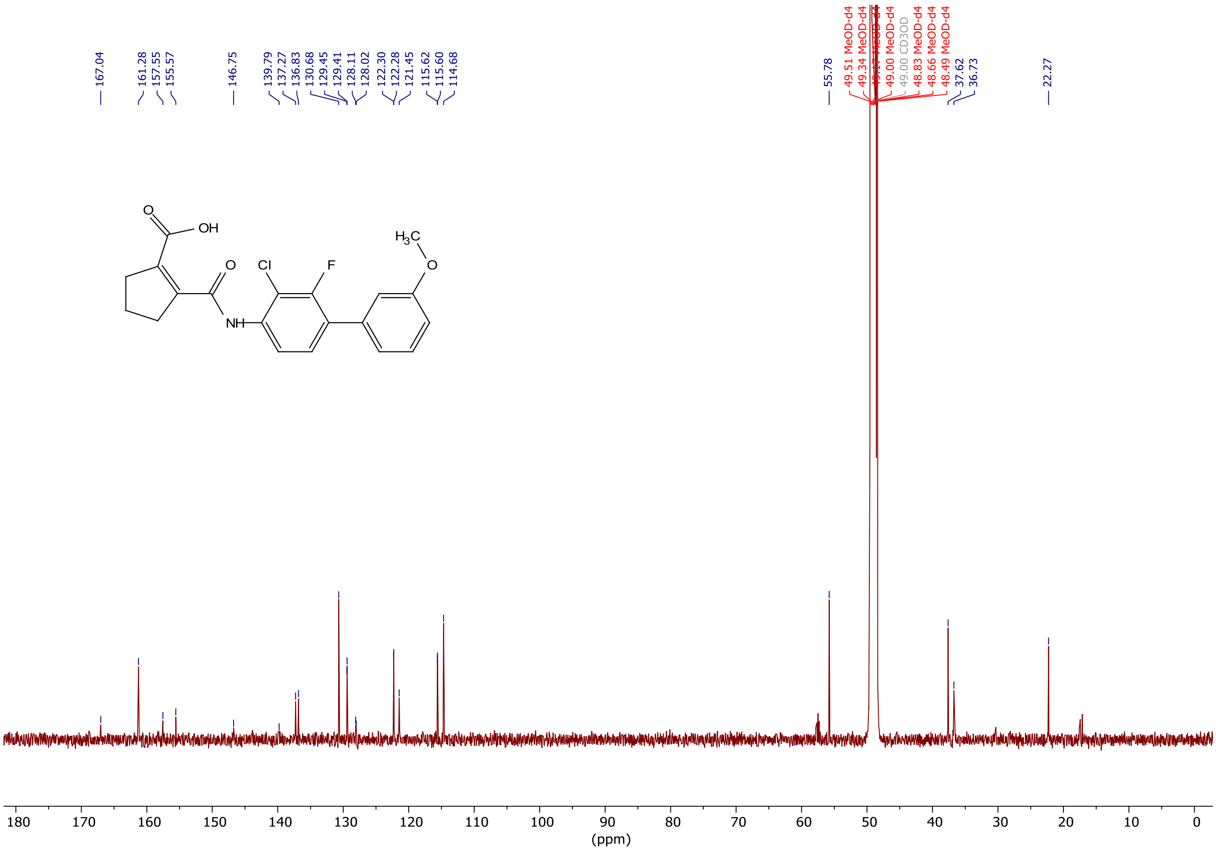

^{13}C NMR (126 MHz, MeOD-d_4) of **49**

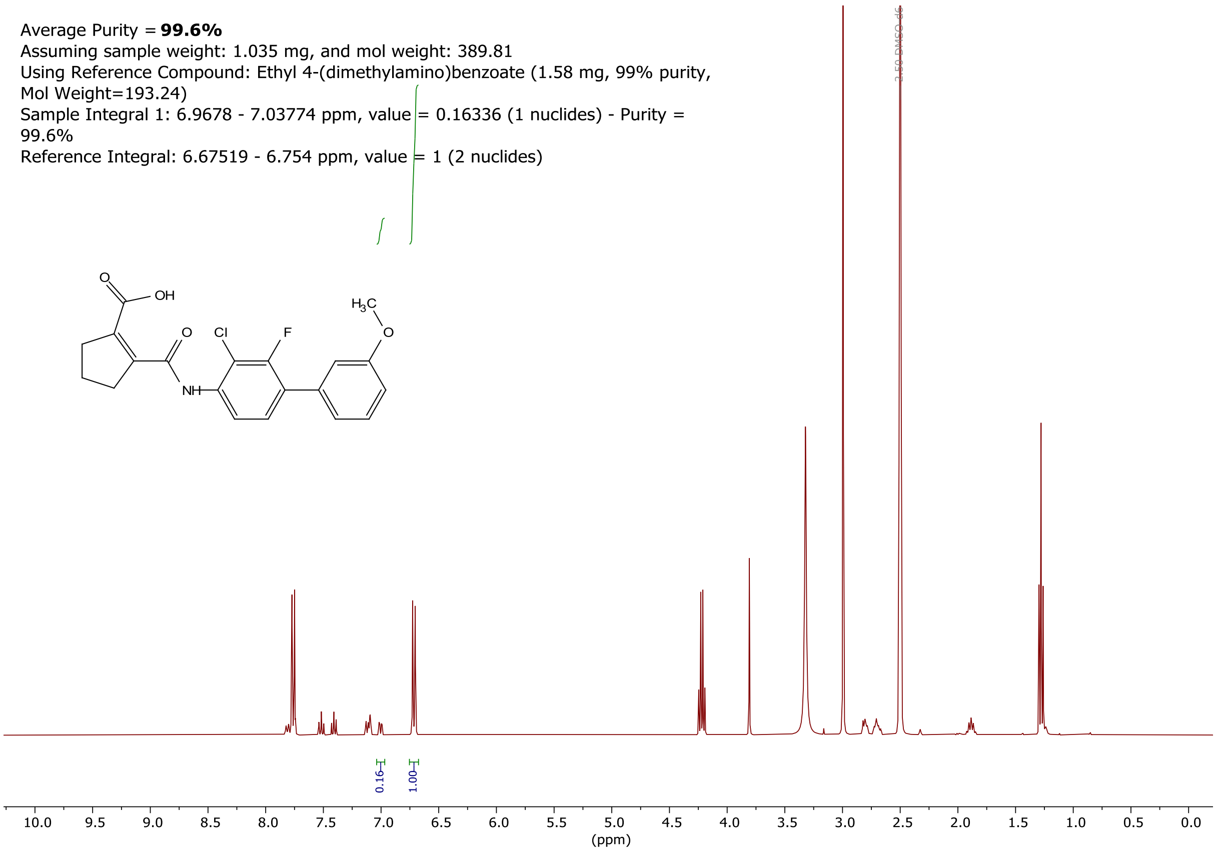

qH NMR (400 MHz, DMSO-d_6) of **49**

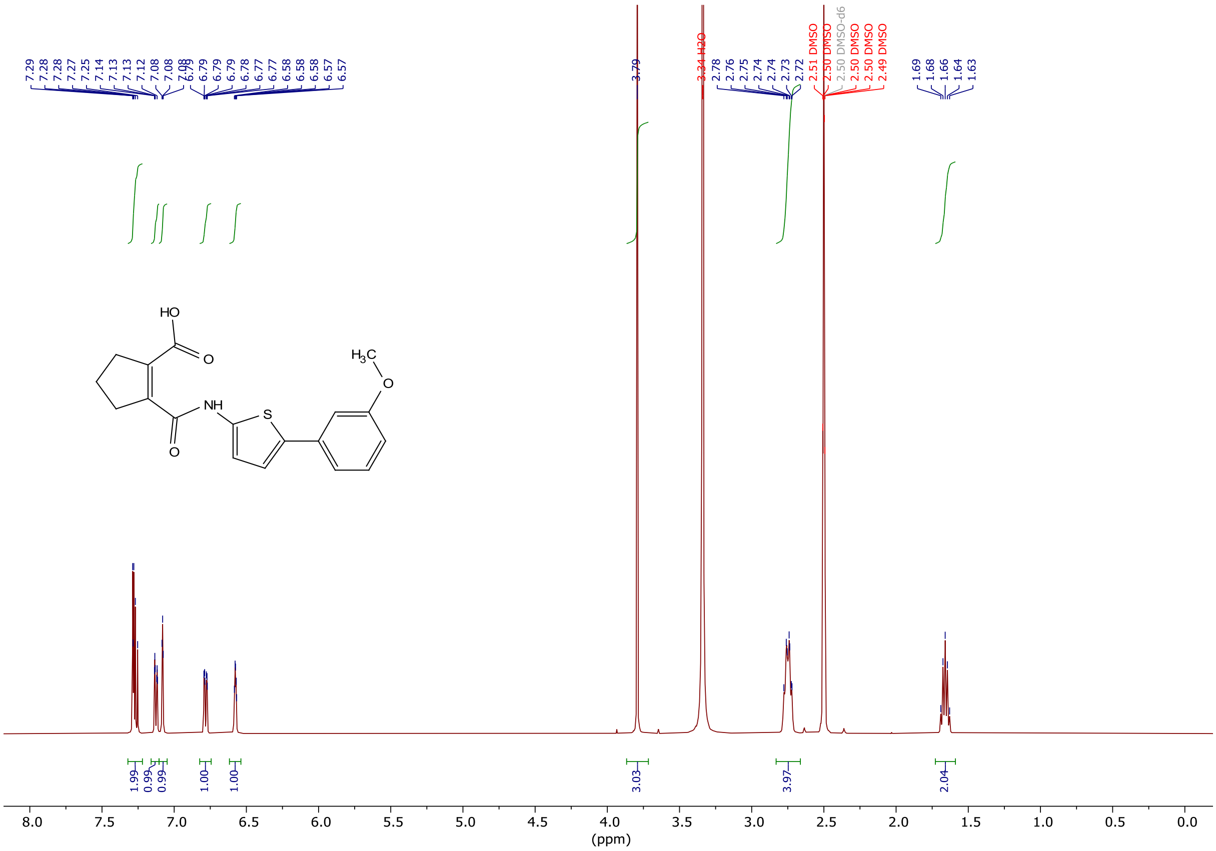

^{1}H NMR (500 MHz, DMSO-d_6) of **50**

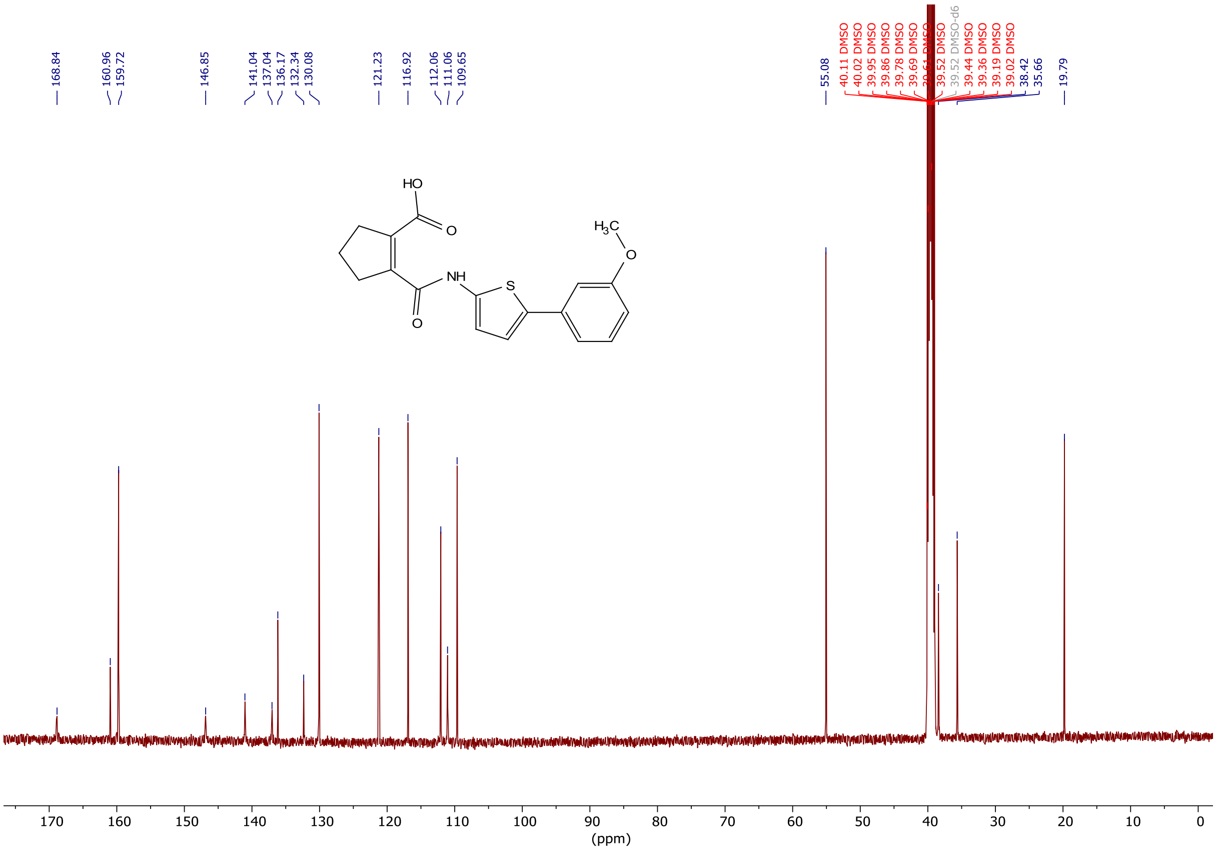

^{13}C NMR (126 MHz, DMSO-d_6) of **50**

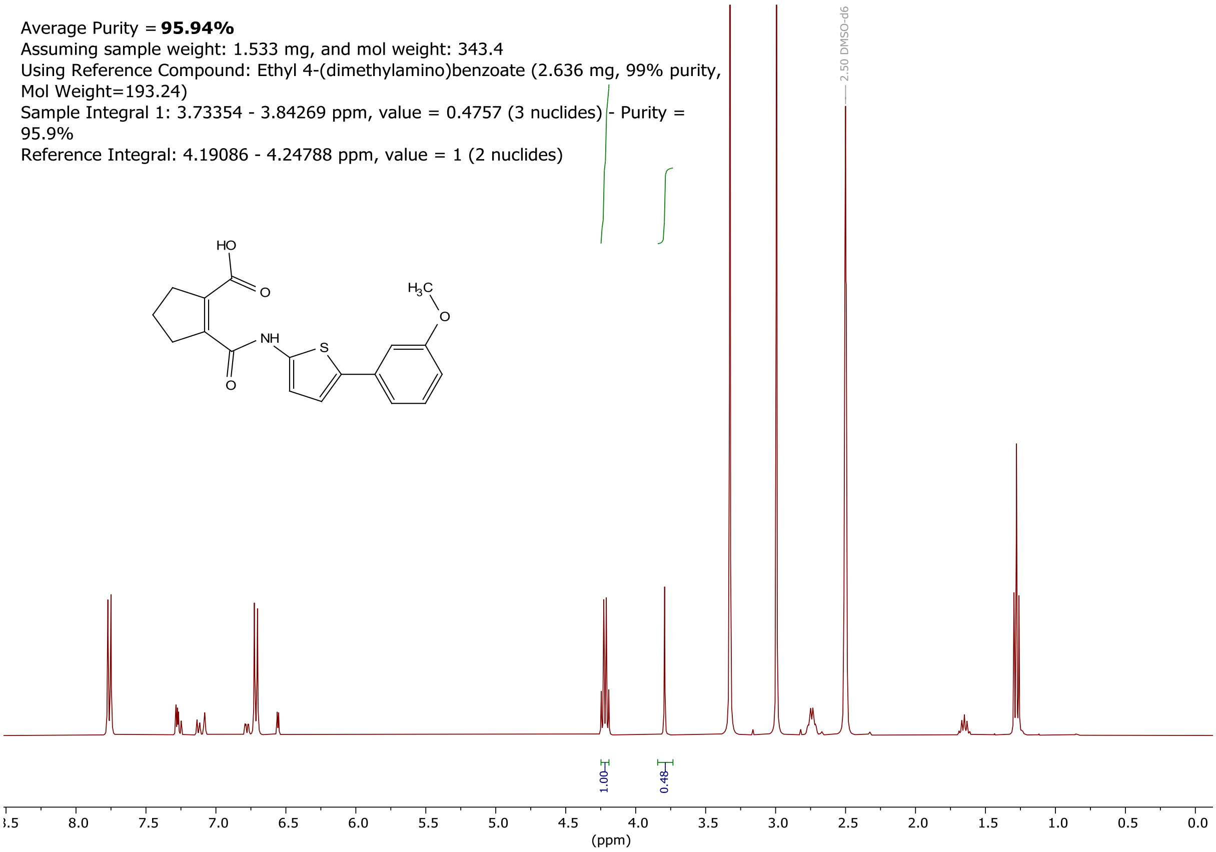

qH NMR (400 MHz, DMSO-d_6) of **50**

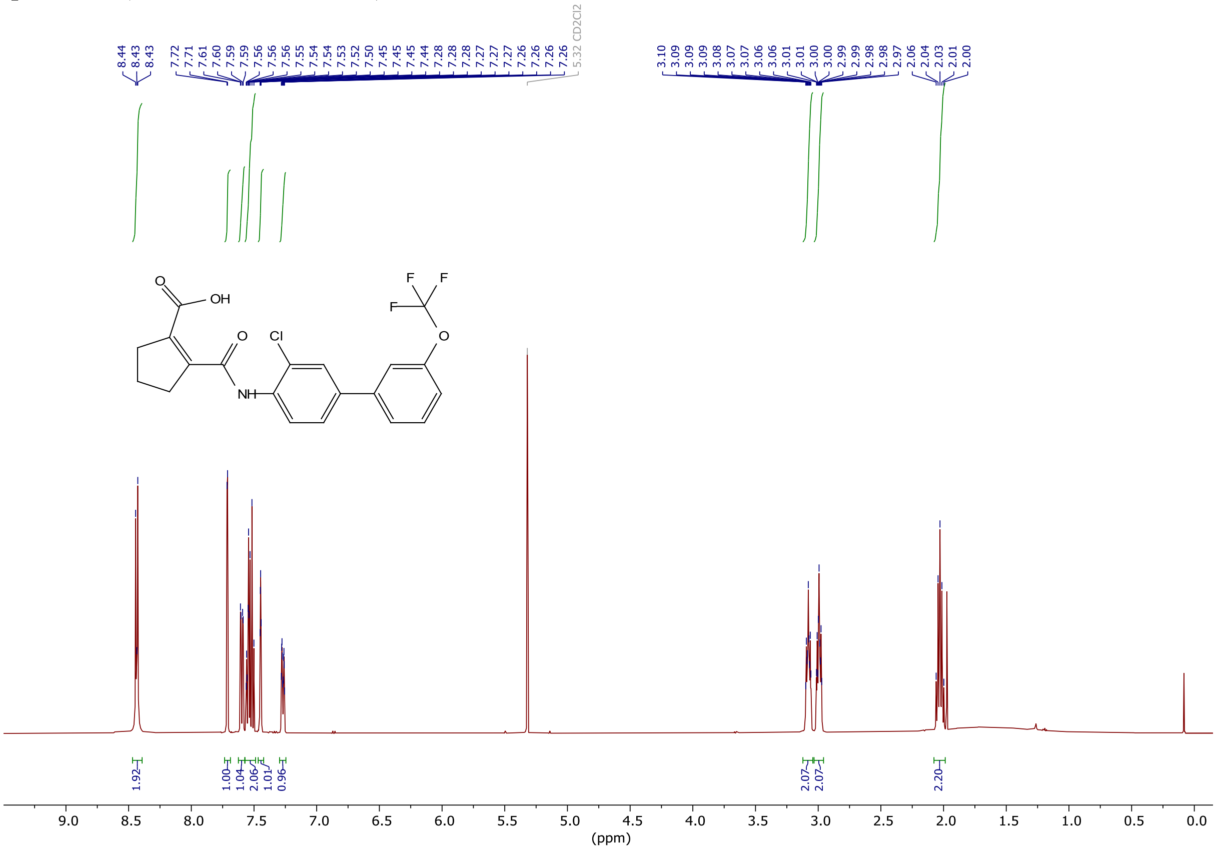

^{1}H NMR (500 MHz, CD_2Cl_2) of **51**

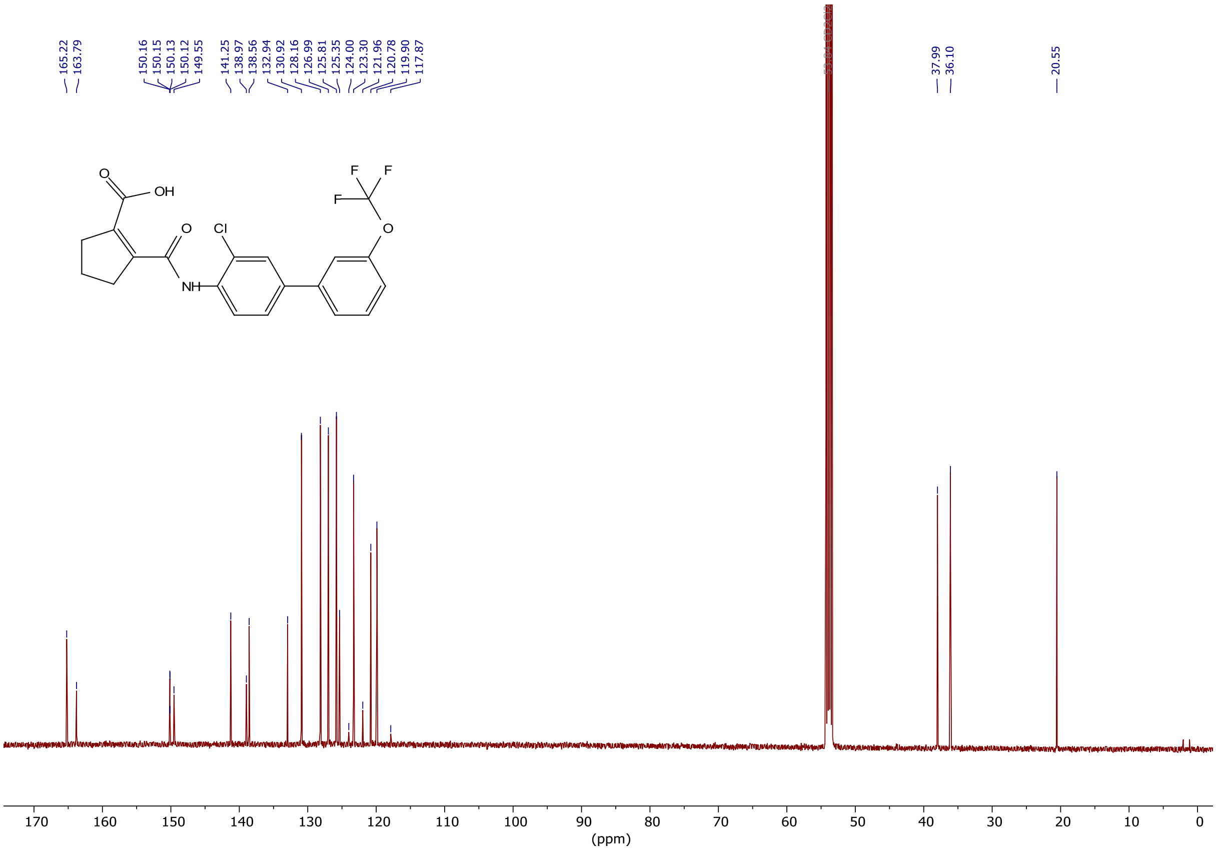

^{13}C NMR (126 MHz, CD_2Cl_2) of **51**

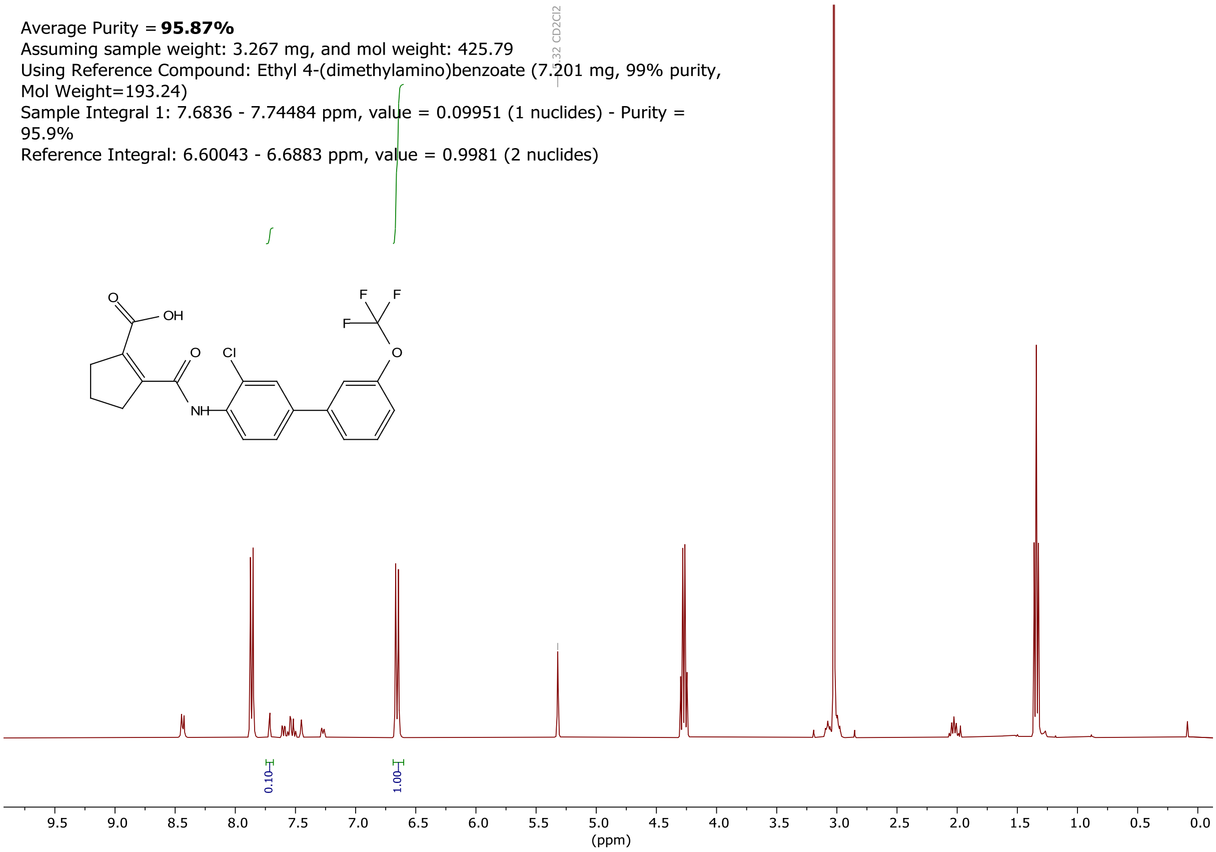

qH NMR (400 MHz, DMSO-d_6) of **51**

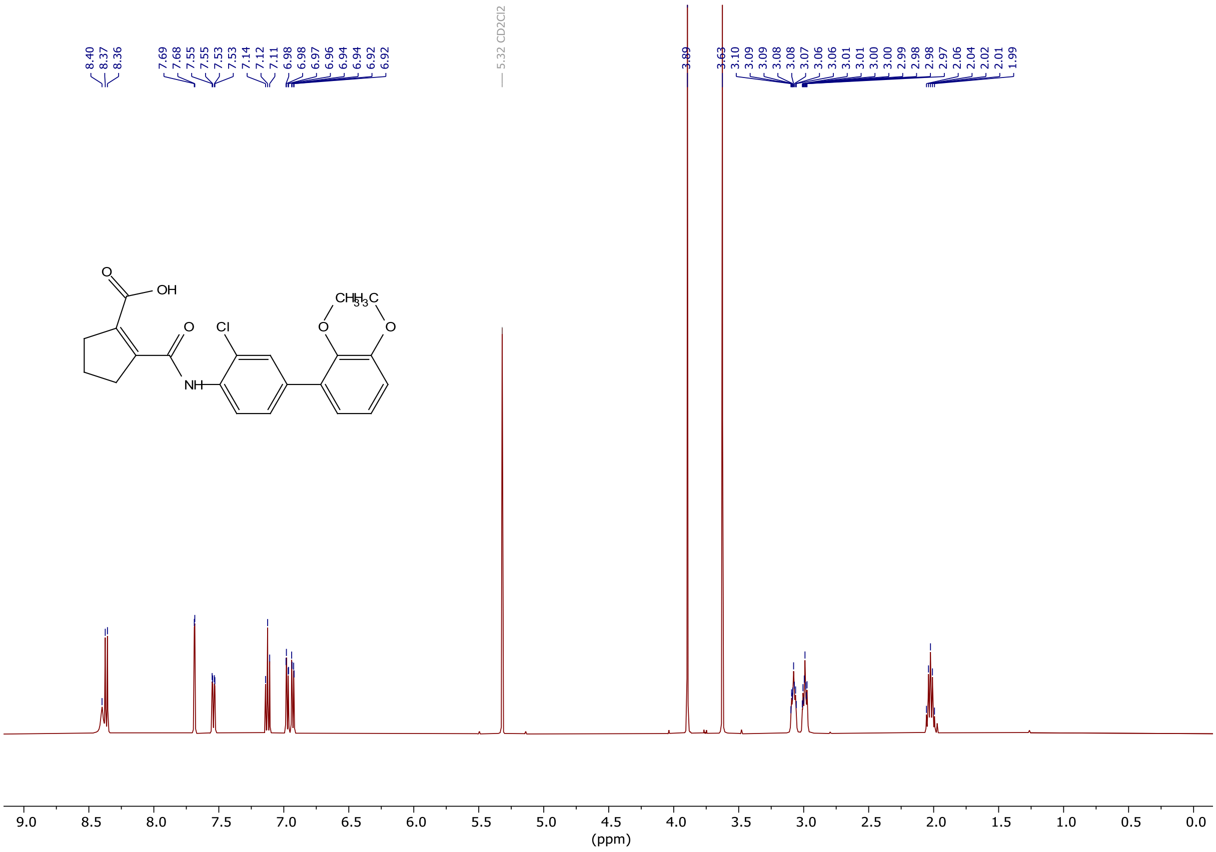

^{1}H NMR (500 MHz, CD_2Cl_2) of **52**

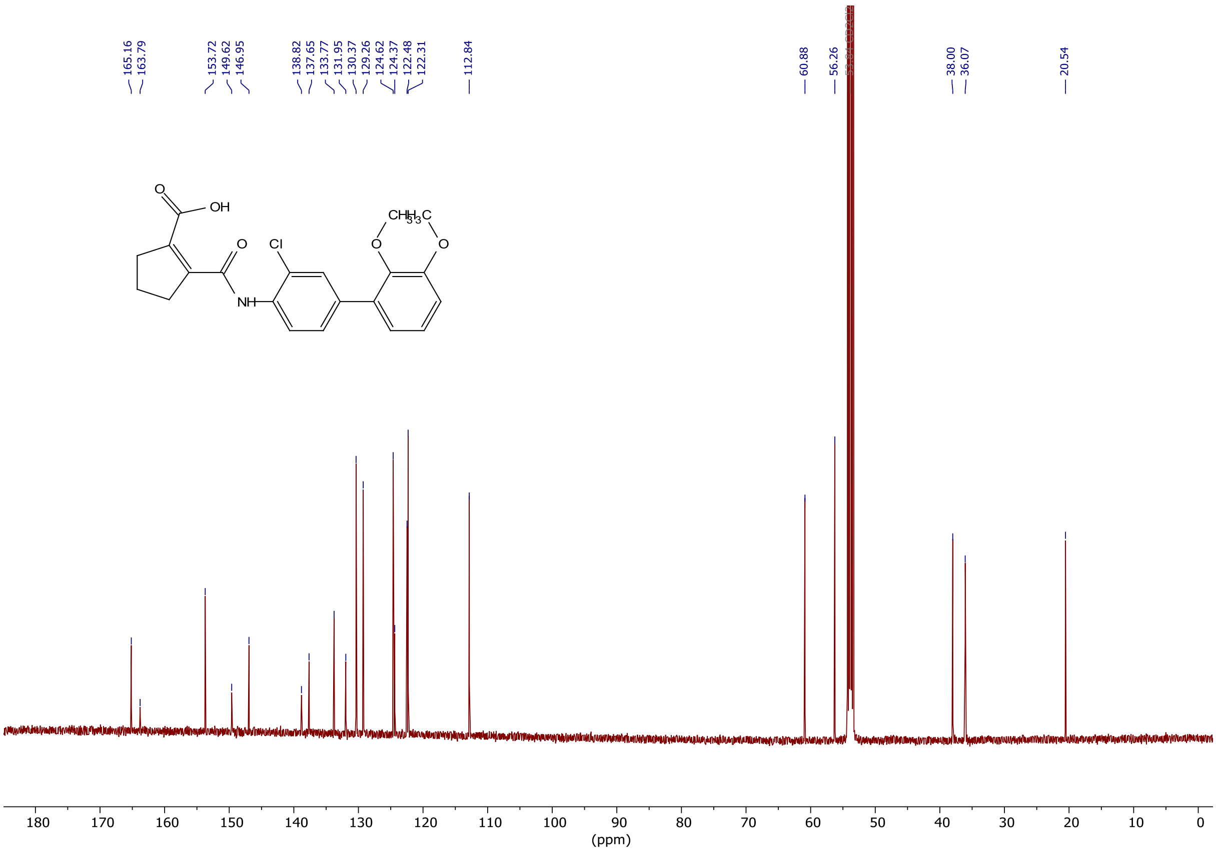

^{13}C NMR (126 MHz, CD_2Cl_2) of **52**

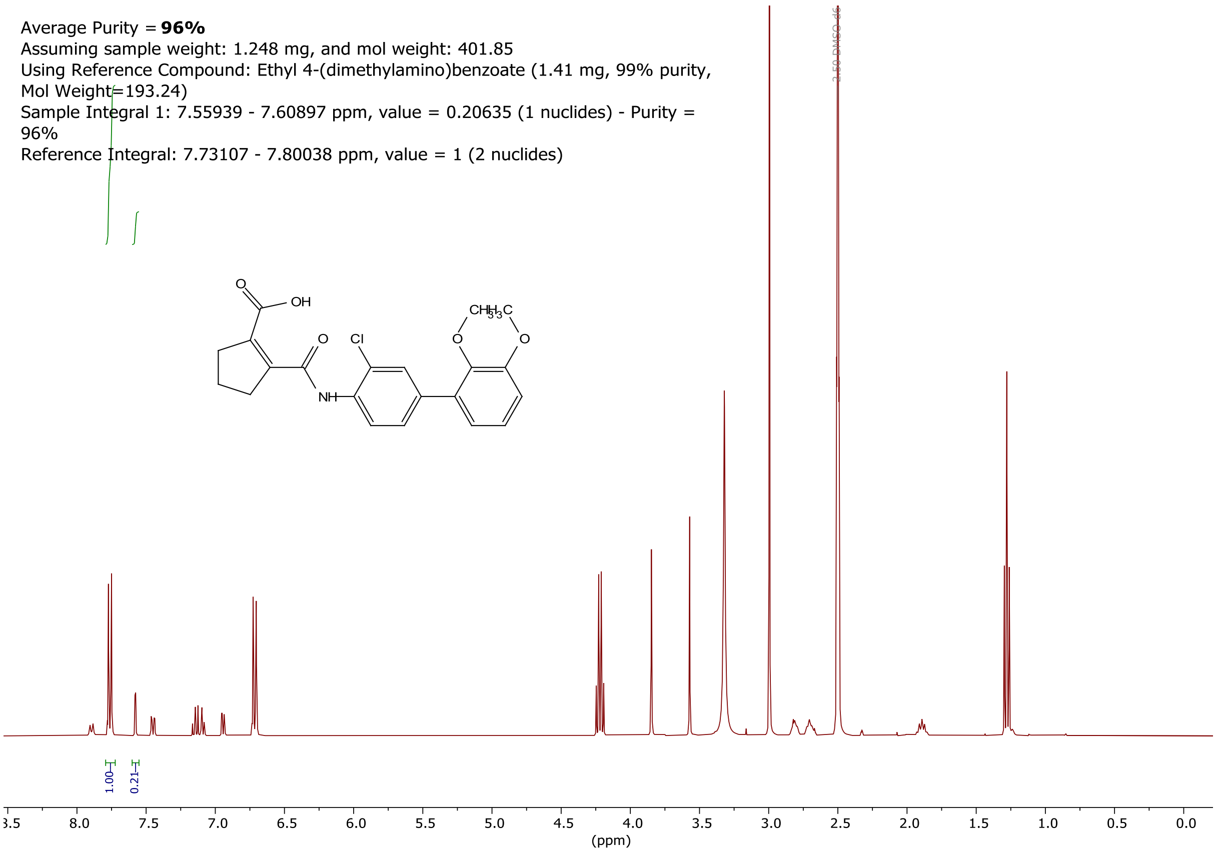

qH NMR (400 MHz, DMSO-d_6) of **52**

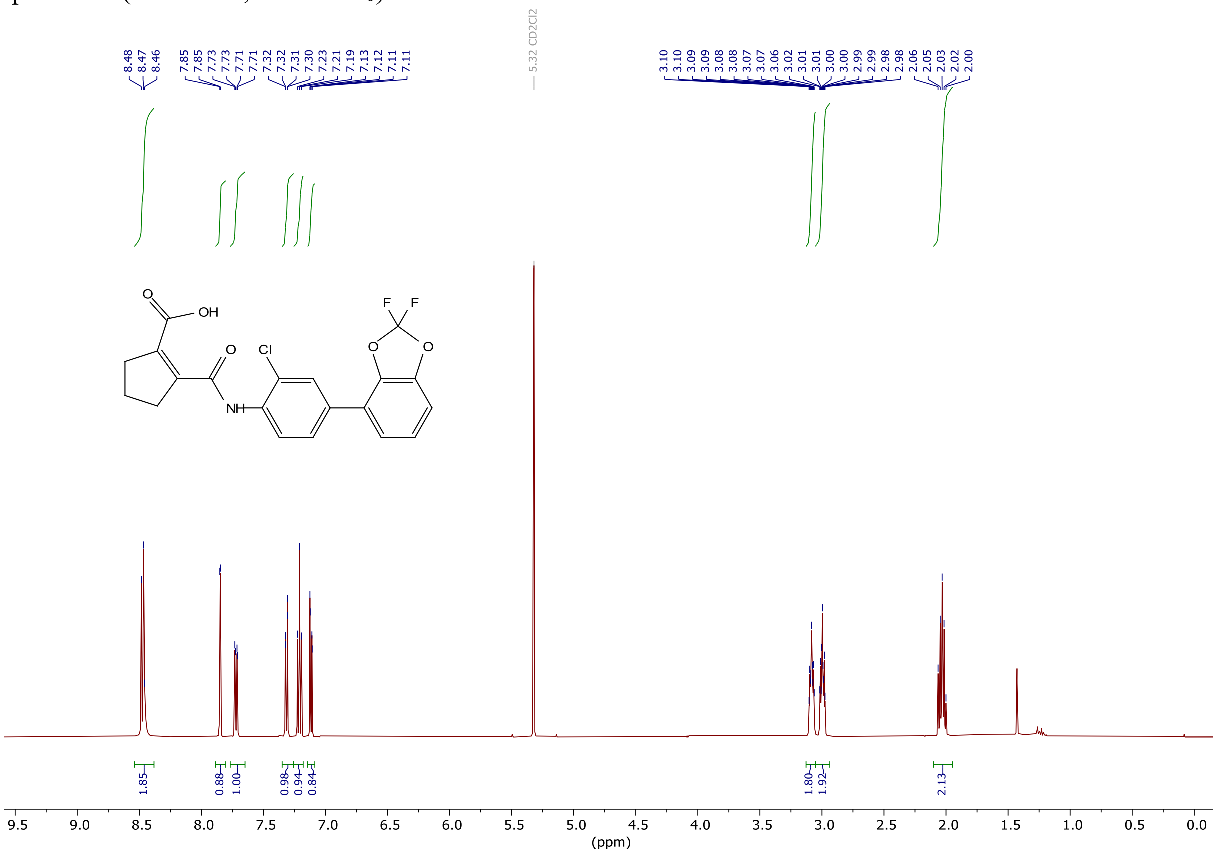

^{1}H NMR (500 MHz, CD_2Cl_2) of **53**

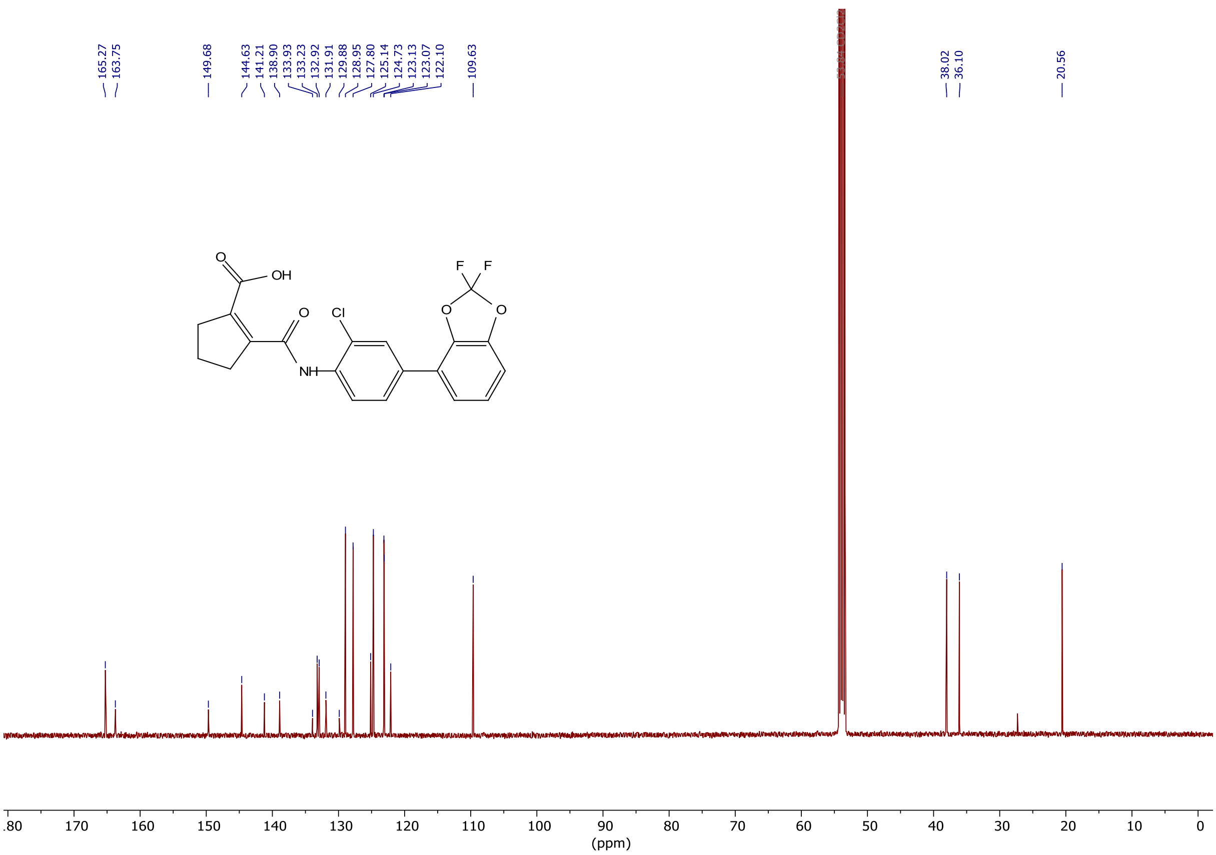

^{13}C NMR (126 MHz, CD_2Cl_2) of **53**

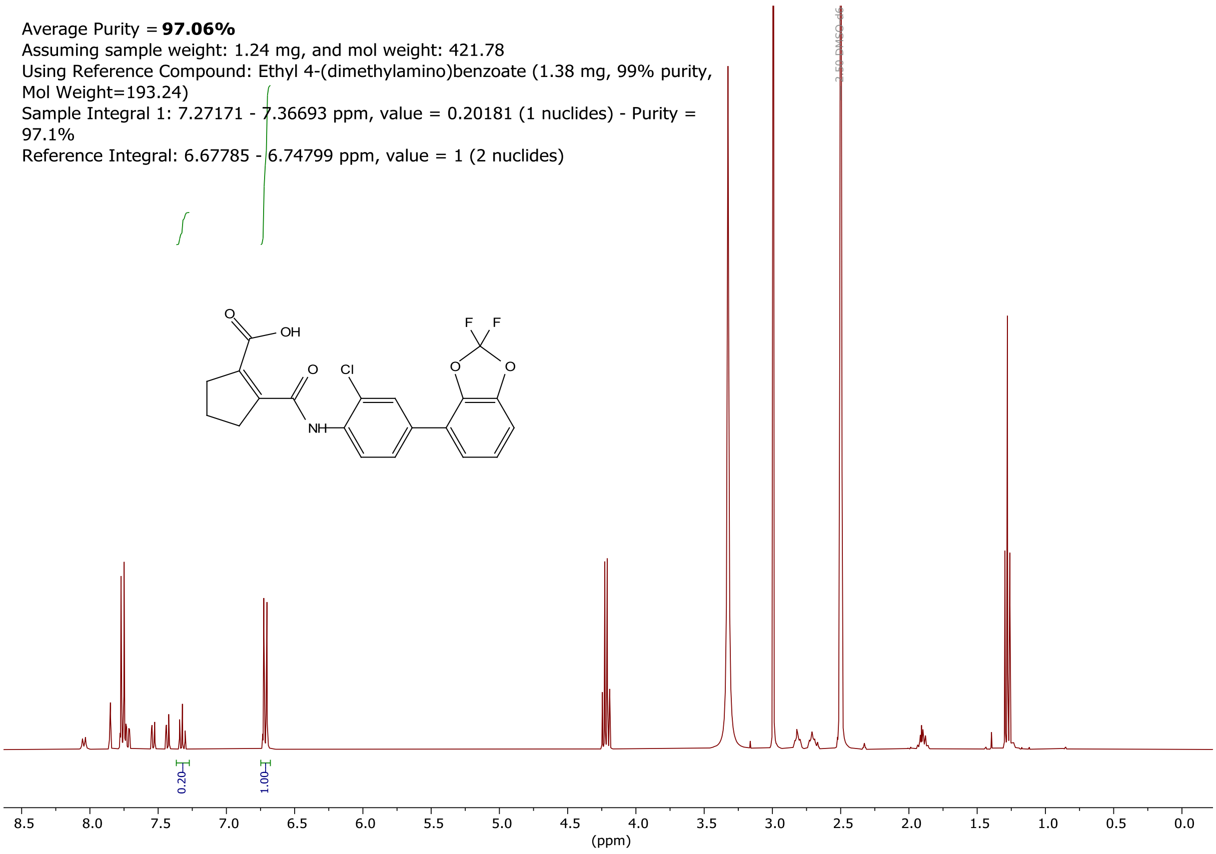

qH NMR (400 MHz, DMSO-d_6) of **53**

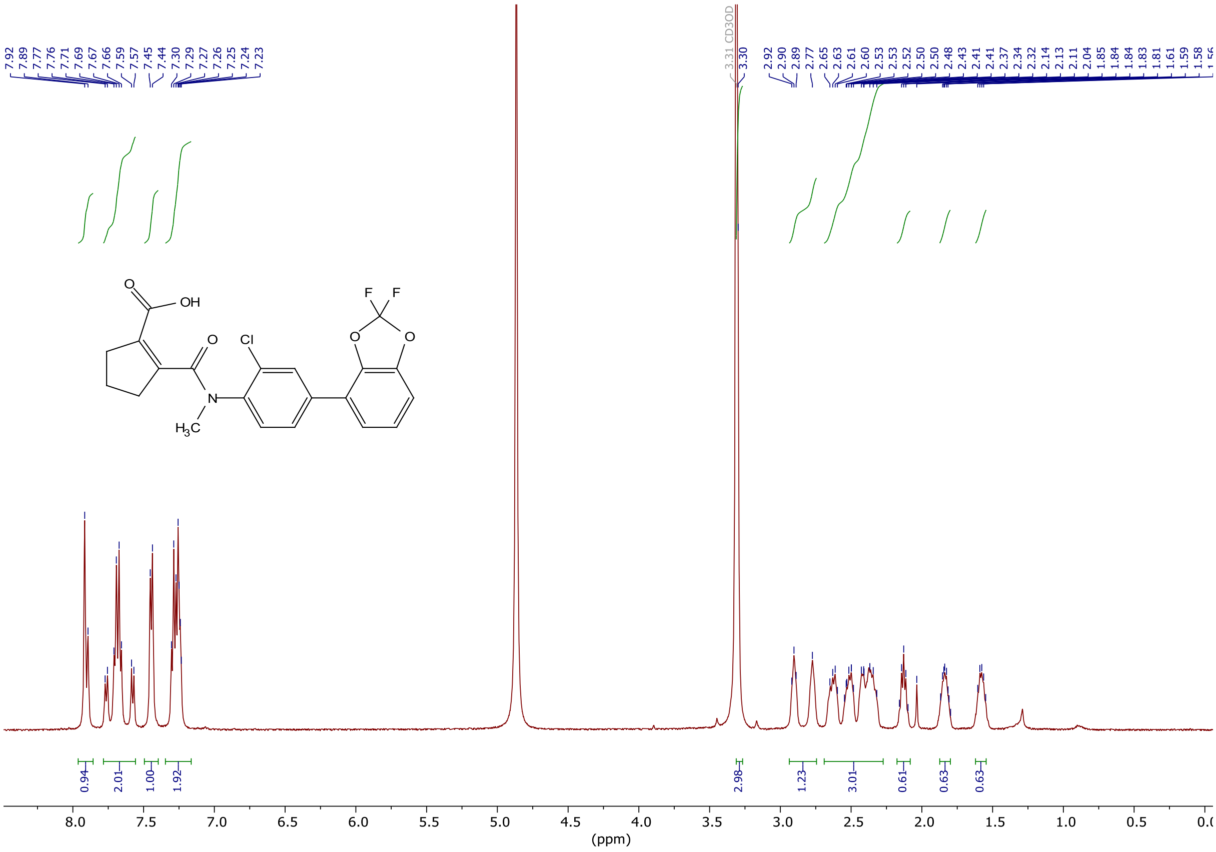

^{1}H NMR (500 MHz, MeOD-d_4) of **54**

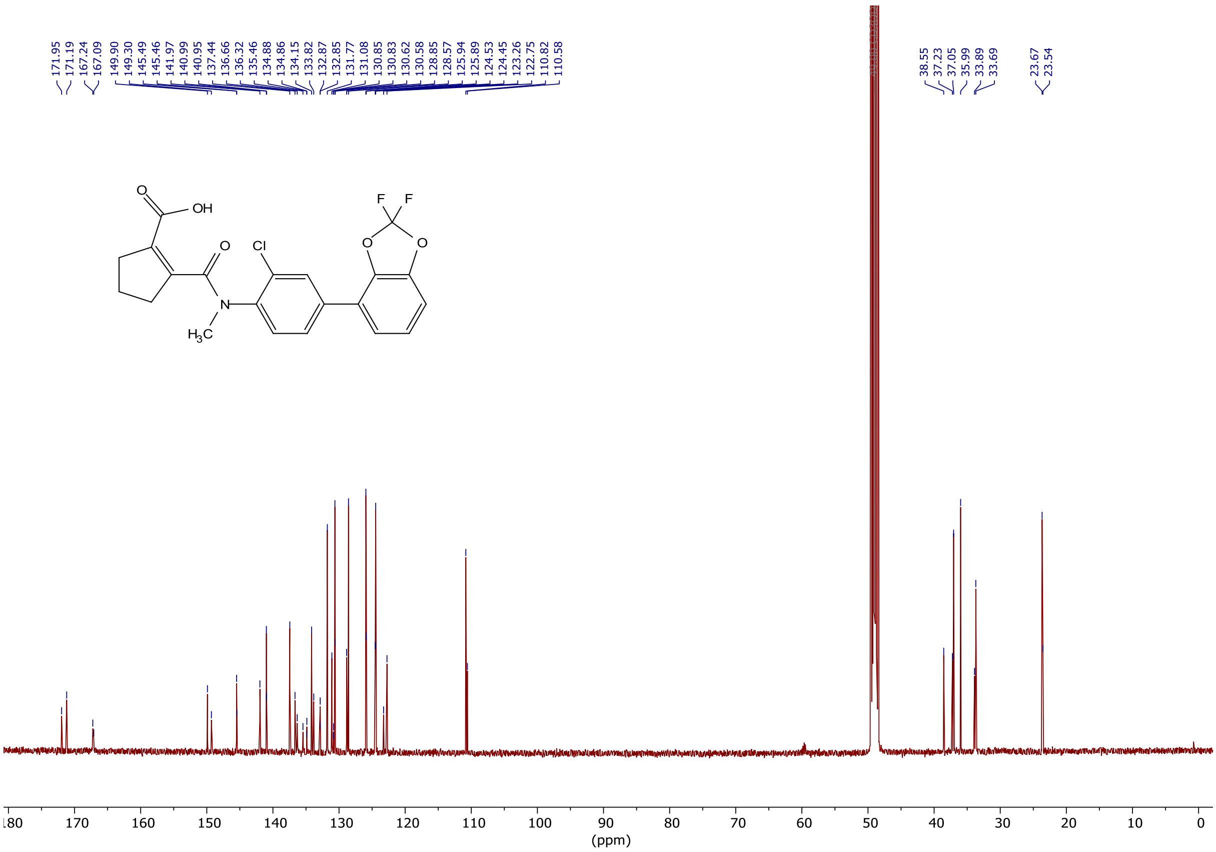

^{13}C NMR (126 MHz, MeOD-d_4) of **54**

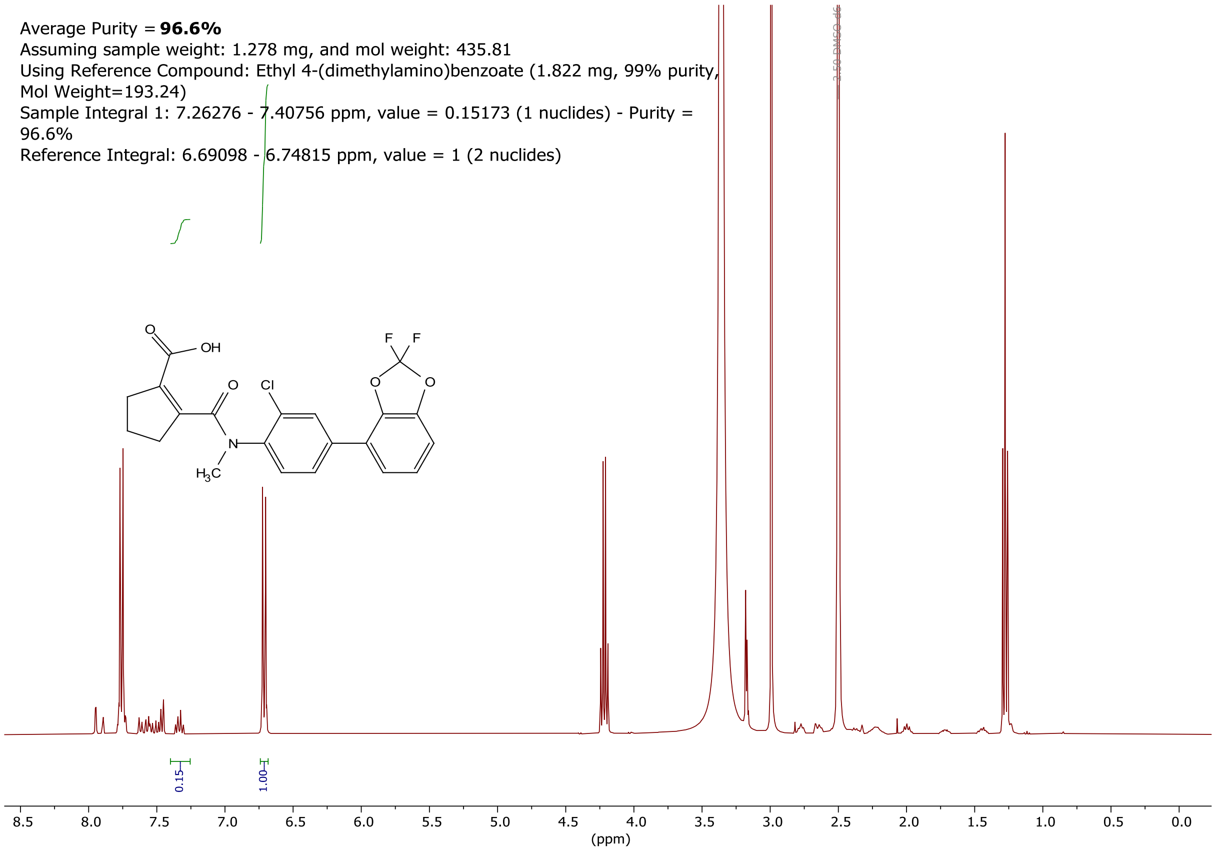

qH NMR (400 MHz, DMSO-d_6) of **54**

Supplementary References

(1) Vietor, J.; Gege, C.; Stiller, T.; Busch, R.; Schallmayer, E.; Kohlhof, H.; Höfner, G.; Pabel, J.; Marschner, J. A.; Merk, D. Development of a Potent Nurr1 Agonist Tool for in Vivo Applications. *J. Med. Chem.* **2023**, *66* (9), 6391–6402.

(2) Kim, W.; Tripathi, M.; Kim, C.; Vardhineni, S.; Cha, Y.; Kandi, S. K.; Feitosa, M.; Kholiya, R.; Sah, E.; Thakur, A.; Kim, Y.; Ko, S.; Bhatia, K.; Manohar, S.; Kong, Y.-B.; Sindhu, G.; Kim, Y.-S.; Cohen, B.; Rawat, D. S. et al. An Optimized Nurr1 Agonist Provides Disease-Modifying Effects in Parkinson's Disease Models. *Nat. Commun.* **2023**, *14* (1), 4283.

(3) Sai, M.; Hank, E. C.; Tai, H.-M.; Kasch, T.; Lewandowski, M.; Vincendeau, M.; Marschner, J. A.; Merk, D. Development of Nurr1 Agonists from Amodiaquine by Scaffold Hopping and Fragment Growing. *Commun. Chem.* **2024**, *7* (1), 149.

(4) Egner, M.; Busch, R.; López-García, Ú.; Lewandowski, M.; Höfner, G.; Wein, T.; Marschner, J. A.; Merk, D. A Nurr1 Agonist Derived from the Natural Ligand DHI Induces Neuroprotective Gene Expression. *J. Med. Chem.* **2025**, *68* (4), 4829–4847.

(5) Willems, S.; Morozov, V.; Marschner, J. A.; Merk, D. Comparative Profiling and Chemogenomics Application of Chemical Tools for NR4A Nuclear Receptors. *J. Med. Chem.* **2025**, *68* (19), 19955–19970.
